# Supplementary material for: Strategies for optimising early detection and obstetric first response management of postpartum haemorrhage at caesarean birth: a modified Delphi-based international expert consensus
Source: BMJ Open. 2024 May 8;14(5):e079713. doi: 10.1136/bmjopen-2023-079713 (PMC11086283; doi:10.1136/bmjopen-2023-079713)
Supplement: Supplementary data [file bmjopen-2023-079713supp001.pdf]

Strategies for optimising early detection and obstetric first response management of postpartum haemorrhage at caesarean birth: A modified Delphi-based international expert consensus

SUPPLEMENTARY MATERIALS

Supplementary Tables 2

Table S1. Definitions of PPH and severe PPH used in clinical guidelines 2

Table S2. Synthesis of evidence and considerations regarding approaches to detect intraoperative and postoperative caesarean PPH and haemodynamic instability 3

Table S3. Synthesis of evidence on thresholds for triggering action on PPH during and after caesarean birth 5

Table S4. Medical Interventions, manoeuvres, and procedures for PPH recommended by WHO compared to recommendations in other PPH guidelines and systematic reviews 7

Table S5. First and second round ratings and agreement for early detection methods for intraoperative and postoperative CB-PPH 11

Table S6. First round ratings and agreement on threshold to initiate treatment for intraoperative CB PPH 12

Table S7. First round ratings and agreement for first response interventions for managing intraoperative and postoperative CB-PPH 14

Table S8. Second round ratings and agreement for first response interventions for managing intraoperative and postoperative CB-PPH 15

Supplementary Figures 16

Figure S1. Interpretation of DI and RAND/UCLA Appropriateness scale 16

Figure S2. PRISMA Flowchart 17

Figure S3. Second round ranking of one-step thresholds to initiate treatment for intraoperative and postoperative CB PPH 18

Supplementary Files 19

Supplementary File S1. Search strategies for each database 19

Supplementary File S2. Systematic literature review methods 24

Supplementary File S3. In-person meeting agenda 43

Supplementary File S4. In-person meeting discussion question guide 46

Supplementary File S5. List of contributors 52

Supplementary File S6. Good Surgical Practices 54

Pingray V, *et al. BMJ Open* 2024; 14:e079713. doi: 10.1136/bmjopen-2023-079713

Supplementary Tables

Table S1. Definitions of PPH and severe PPH used in clinical guidelines

Definitions	Number of guidelines using the definition	Specific guidelines using the definition	Specifications regarding mode of birth and timeframe in which bleeding occurs	Additional considerations
**DEFINITIONS OF PPH**				
Blood loss at least 500 mL	6	(1, 2, 3, 4, 5, 6)	Explicitly specified that this definition applied regardless of the mode of birth: (3, 6)<br>Indicated that blood loss must occur within 24 h of birth: (1, 2, 6)	Two guidelines targeting high-income countries (HICs) specified that for caesarean birth the threshold could be set at a higher blood loss if clinically tolerated: (3, 4)
Blood loss at least 1000 mL	2	(7, 8)	Definition applied for caesarean only: all<br>No timeframe indicated: all	---
Blood loss at least 1000 mL OR signs of haemodynamic instability	5	(9, 10, 11, 12, 13)	Definition applied for caesarean only: (9, 10)<br>Indicated that blood loss must occur within 24 h of birth: all	One guideline recommended that cumulative blood loss of 500-999 mL alone should trigger increased supervision and potential interventions as clinically indicated: (13)
Any bleeding that causes haemodynamic instability	1	(14)	Explicitly specified that definition was applicable regardless of mode of birth<br>Indicated that bleeding must occur within 24 h of birth	For clinical purposes, any blood loss that had the potential to produce hemodynamic instability should be considered PPH. The amount of blood loss required to cause hemodynamic instability would depend on pre-existing conditions (e.g., anaemia, dehydration, gestational hypertension with proteinuria)
**DEFINITIONS OF SEVERE PPH**				
Blood loss at least 1000 mL	4	(1, 2, 3, 5)	Explicitly specified that this definition applied regardless of the mode of birth: (3)<br>Indicated that bleeding must occur within 24 h of birth: (1, 2)	RCOG defined major PPH as blood loss greater than 1000 mL. Major PPH could be further subdivided into moderate (1001–2000 ml) and severe (more than 2000 ml).
Blood loss at least 1000 mL OR signs of haemodynamic instability	2	(6, 12)	Explicitly specified that this definition applied regardless of the mode of birth: (6)<br>Indicated that bleeding must occur within 24 h of birth: all	---

Note: No definition of PPH mentioned: (15, 16, 17). No definition of severe PPH mentioned (4, 8, 11, 13, 16, 17, 18)

Table S2. Synthesis of evidence and considerations regarding approaches to detect intraoperative and postoperative caesarean PPH and haemodynamic instability

Detection methods	Paired with any of the following blood collection devices	Sources discussing this method	Summary of the evidence
**BLOOD LOSS ASSESSMENT METHODS WITH SPECIFIC BLOOD COLLECTION DEVICES**			
Visual estimation of blood loss	• Suction canister<br>• Blood-soaked materials and clots<br>• Calibrated drape<br>• Non-calibrated blood loss collectors placed under the buttocks<br>• Non-calibrated blood loss collectors attached to the abdomen during surgery	4 Guidelines: (1, 8, 11, 14)<br><br>3 Systematic reviews: (19, 20, 21)<br><br>Additional relevant references: (22)	While four guidelines stated that visual estimation was used in practice, none actually recommended this method. Rather, they described its limitations. Visual estimation was described as subjective, imprecise, and known to underestimate actual blood loss (5, 17). Clinicians were advised to be aware that visual estimation of peripartum blood loss is inaccurate (2). The systematic reviews noted that some trialists used visual estimation to determine amount of blood lost and detect PPH. No further comment was provided. Although widely used for the detection of PPH, visual estimation of blood loss was consistently reported as inaccurate (22). While both underestimation and overestimation occur, the extent of underestimation increased as the volume of blood loss increased (17, 22).
Volumetric	• Calibrated suction canister<br>• Calibrated drape	6 Guidelines: (2, 3, 4, 6, 13, 17)<br><br>3 Systematic reviews: (19, 20, 21)<br><br>Additional relevant references: (23, 24, 25, 26, 27)	Guidelines described the use of volumetric methods. Some guidelines proposed using a combination of volumetric and gravimetric methods for assessing blood loss (3, 17). Some guidelines noted that while quantitative methods are more accurate than visual estimation in determining maternal blood loss, their effect on clinical outcomes has not been demonstrated (2, 17). The systematic reviews noted that some trialists used this method to determine amount of blood lost and detect PPH. Some trialists used a combination of gravimetric and volumetric methods. No further comment was provided. Volumetric techniques appeared to be more accurate than the visual estimation of blood loss, irrespective of provider experience, level of training, or specialty (23, 24). Mean measured blood loss was found to be 30% more accurate than estimated blood loss in vaginal births(25). The discrepancy between volumetric methods and visual estimation was found to be higher with increasing blood volume (26). The effectiveness of volumetric methods on clinical outcomes has not been demonstrated (17). A large multicentre multi-country cluster randomized trial comparing calibrated drapes vs. visual estimation failed to show a reduction in severe PPH (27)
Gravimetric	• Non-calibrated blood loss collectors placed under the buttocks<br>• Non-calibrated blood loss collectors attached to the abdomen during surgery<br>• Blood-soaked materials and clots: either intra- or postoperative	8 Guidelines: (2, 3, 4, 5, 6, 12, 13, 17)<br><br>2 Systematic reviews: (19, 20)<br><br>Additional relevant references: (28, 29)	Guidelines described the use of gravimetric methods. Some guidelines proposed using a combination of volumetric and gravimetric methods for assessing blood loss (3, 12, 17). Some guidelines noted that while quantitative methods are more accurate than visual estimation in determining maternal blood loss, their effect on clinical outcomes has not been demonstrated (2, 12, 17). Two guidelines stated that weighing of swabs *may* be used, but did not directly suggest their use (2, 4). The systematic reviews noted that some trialists used this method to determine amount of blood lost and detect PPH. Some trialists used a combination of gravimetric and volumetric methods. No further comment was provided. A 2014 randomized controlled trial including nine hundred women presenting for vaginal delivery found that blood loss recorded using a non-calibrated collector followed by gravimetric assessment was lower than blood loss recorded using the calibrated drape for

Detection methods	Paired with any of the following blood collection devices	Sources discussing this method	Summary of the evidence
			blood collection followed by volumetric assessment, with a mean difference in recorded blood loss of 58.6ml (28). One study of 228 women with PPH following vaginal delivery found weighing blood loss compared to Hgb drop (of 10%) had a sensitivity of <75% and a specificity of 97%). These findings were modelled at hypothetical high prevalence PPH settings (15%, 30%), where the Positive Predictive Value was >86% (29).
**METHODS OF DETECTING HEMODYNAMIC INSTABILITY SECONDARY TO PPH DURING AND AFTER CAESAREAN BIRTH**			
Clinical signs of haemodynamic instability	• N/A	6 Guidelines: (2, 3, 5, 10, 12, 14) 3 Systematic reviews: (19, 20, 21) Additional relevant references: (30, 31, 32, 33, 34, 35, 36)	Signs of haemodynamic instability reported in guidelines included changes in blood pressure, heart rate, pulse oximetry, urine output, or general status (faintness/dizziness, nausea, thirst, altered level of consciousness, pallor, sweating, poor capillary refill, and cold extremities. The guidelines proposed considering clinical signs and symptoms of haemodynamic instability in combination with other methods (such as volumetric and gravimetric methods for blood loss assessment) for the detection of PPH.(2, 3, 5, 10, 12, 14). Some of these guidelines presented tables correlating clinical signs with blood loss and the degree of shock. However, most of them warned that many clinical signs and symptoms do not occur until the blood loss reaches very high levels due to the physiological increase in circulating blood volume during pregnancy. Some other guidelines (6, 12, 13, 14) explicitly proposed including clinical signs and symptoms of haemodynamic instability or the use of the shock index specifically in assessing PPH severity. None of the trials included in the systematic reviews used this method to detect PPH. The Shock Index (SI) as a predictor of several maternal outcomes has been evaluated in the context of PPH research, including both vaginal and caesarean birth. SI performance was usually reported using the Area under the Curve (AUC) parameter (with estimates between 0.7 - 0.8 in most studies). According to the cut-off value of the SI chosen and the specific outcome analysed, SI sensitivity may range from 30% to 90%(30, 31, 32, 33, 34, 35). A recent stepped-wedge cluster randomized trial showed insufficient evidence to suggest that a significant benefit or harm could be attributed using an automated SI device (in low-resource settings) on a composite outcome of maternal deaths, eclampsia, or emergency (36). However, the rate of emergency hysterectomy was significantly reduced.
Visual charts and early warning scores (EWS)	• N/A	5 Guidelines: (2, 4, 6, 12, 13) 0 Systematic reviews: None reference this method. Additional relevant references: (37)	A few guidelines recommended visual charts and early warning scores to alert caregivers to abnormal trends in haemodynamic measurements. However, these seem to be recommended for follow-up monitoring of diagnosed PPH cases rather than for diagnosis of PPH. None of the trials included in the systematic reviews used this method to detect PPH. A systematic literature review of 17 published obstetric EWS reported that they had very high median sensitivity (89%) and specificity (85%) but low median positive predictive values (41%) for predicting morbidity or ICU admission. Obstetric EWS had high accuracy in predicting death (AUROC >0.80) among critically ill obstetric women (37).

Table S3. Synthesis of evidence on thresholds for triggering action on PPH during and after caesarean birth

Thresholds	Guidelines recommending this threshold	Applicable mode of birth (if thresholds differ by mode of birth)	Comments
**ONE-STEP APPROACH: THRESHOLDS TRIGGER FULL RESPONSE PROTOCOL**			
Blood loss at least 1000 mL OR signs of haemodynamic instability	1. (10)<br>2. (11)<br>3. (12)<br>4. (9)	Three guidelines proposed this threshold as being specific for caesarean birth(9, 10, 12).	In these guidelines the same criteria proposed as the definition of PPH were also used as the threshold for initiating treatment.
Blood loss at least 500 mL	1. (6)	This guideline stated that this threshold applied regardless of the mode of birth.	The guideline noted that clinical signs and symptoms of hypovolaemia should be included in the assessment of PPH severity. However, clinical signs of hypovolaemia are misleading in pregnancy due to plasma volume expansion and might not become evident until blood losses reach 1,000-1,500 mL in healthy women. Thus, the blood loss thresholds should depend on the woman's clinical condition and local resources. In this guideline the same criteria proposed for the definition of PPH were also used as the threshold for initiating treatment.
Blood loss at least 500 mL OR Signs of haemodynamic instability	1. (3)	This guideline stated that this threshold applied regardless of the mode of birth.	This guideline noted that the bleeding rate, PPH etiology, and clinical context should be considered. Further, for caesarean births, thresholds could be set at a higher blood loss if clinically tolerated. In this guideline the same criteria proposed for the definition of PPH were also used as the threshold for initiating treatment.
Blood loss at least 1000 mL	1. (7)	This guideline stated that this threshold is specific for caesarean birth.	In this guideline the same criteria proposed for the definition of PPH were also used as the threshold for initiating treatment.
Any excessive bleeding with signs of hemodynamic instability	1.	This guideline stated that this threshold is applicable to all births.	In this guideline the same criteria proposed for the definition of PPH were also used as the threshold for initiating treatment.
**TWO/STEP APPROACH: THRESHOLDS TRIGGER DIFFERENTIAL ACTIONS**			
Lower threshold: Blood loss at least ***500 mL*** *without clinical shock; Higher threshold: Blood loss at least* ***1000 ml,*** *continued bleeding, OR clinical shock*	1. (2)<br>2. (4)<br>3. (5)<br>4. (13)	Guidelines did not specify a difference in threshold by mode of birth.	According to these guidelines, blood loss of **500–1000 ml** (minor PPH) without clinical shock should trigger: close monitoring, laboratory tests, and the use of crystalloid infusion [(2)] ; prompt basic measures (close monitoring, intravenous access, full blood count, group, and screen, insert urinary catheter) to facilitate resuscitation [(4)] ; enhanced surveillance and early interventions as needed[(13)] . Blood loss ≥ **1000 ml** and continued bleeding or clinical shock should trigger a full protocol to achieve resuscitation and haemostasis. [(2, 4, 13)]Three guidelines aligned their proposed lower thresholds with their proposed PPH definitions. [(2, 4, 5)] A single guideline aligned its proposed higher threshold with its PPH definition. [(13)]

Thresholds	Guidelines recommending this threshold	Applicable mode of birth (if thresholds differ by mode of birth)	Comments
Lower threshold: Blood loss at least 1000 mL; Higher threshold: Blood loss of at least 2000 mL OR SI of ≥1.0	1. (8)	Guideline provided thresholds specific for CB	According to this guideline, blood loss of **1000 mL** should trigger suspicion of PPH and initiation of treatment. Blood loss of *at least* **2000 mL or SI of ≥1.0** should trigger: initiation of IV catheter with a large gauge and replacement of a sufficient volume of fluid; consideration of blood transfusion and the transportation of the patient to a secondary or tertiary hospital; monitoring of blood pressure, pulse rate, bleeding amount, urine output and SpO2. This guideline used the same criteria as thresholds for action as were used for proposed PPH definition.

*Four guidelines (1, 15, 16, 17)did not mention any thresholds for initial assessment or to trigger a full protocol. Note CB= caesarean birth

Table S4. Medical Interventions, manoeuvres, and procedures for PPH recommended by WHO compared to recommendations in other PPH guidelines and systematic reviews

Method	WHO recommendation (PPH 2012,TXA 2017, Carbetocin 2018, and UBT 2021) (1, 9, 38, 39)	Other Reviewed Guidelines	Systematic Reviews
**MEDICAL INTERVENTIONS**			
**Uterotonics**			
Oxytocin	Intravenous oxytocin was the recommended first-line treatment for PPH, including among those who have already received oxytocin for prevention of PPH (no dosing information included; 10 IU IM or IV was the recommended dosing and route of administration provided for prophylactic use).	Recommended as first line drug (2, 3, 4, 5, 6, 7, 10, 12, 13, 14, 17)<br>Two different dosing regimens discussed:<br>• Intravenous oxytocin 5 IU (slow IV injection over 2 minutes). May repeat dose once.<br>• Intravenous infusion 5-10 IU per hour (20-40 IU in 500 ml saline over 4 hours).	Not described.
Carbetocin	Not described in the 2012 WHO treatment guidelines. The 2018 WHO prevention guidelines recommended prophylactic carbetocin (100 µg, IM/IV) for all births when cost was comparable to other effective uterotonics, but did not recommend the use of carbetocin as a treatment for PPH.	Recommended as a first line drug (7, 14)or as a second-line drug (10) for treatment.	Not described.
Ergometrine	Recommended if IV oxytocin unavailable, or bleeding nonresponsive to oxytocin. No dosing amount provided. Intravenous route of administration recommended.	Intravenous or intramuscular routes recommended. Maximum dose of 1000 mcg. 250-500 mcg (IV slow, over 2 minutes, or IM), may repeat every 5 minutes (2, 4, 5, 12)	Ergometrine 200 mcg administered intramuscularly, followed by 250 mcg IM carboprost if needed (21)
Oxytocin-ergometrine fixed dose	Recommended if IV oxytocin unavailable, or bleeding nonresponsive to oxytocin. No dosing amount or route of administration recommended. `	Not described.	Syntometrine® (ergometrine 500 mcg plus oxytocin 5 IU) administered intramuscularly plus oxytocin 10 IU administered by an intravenous infusion (21)

Method	WHO recommendation (PPH 2012,TXA 2017, Carbetocin 2018, and UBT 2021) (1, 9, 38, 39)	Other Reviewed Guidelines	Systematic Reviews
Prostaglandin (including sublingual misoprostol, 800 mcg)	Recommended if IV oxytocin unavailable, or bleeding nonresponsive to oxytocin. No dosing amount or route of administration information provided other than for misoprostol (recommended 800 mcg, administered sublingually). Sublingual misoprostol particularly recommended in settings where IV oxytocin unavailable and IM oxytocin used for prophylaxis.	Carboprost was recommended either: as a general second line drug (12), specifically for use after oxytocin/ergometrine (2), or recommended generally with no specific order cited (5). Most reviewed guidelines recommended a dose of 250 mcg IM, which could be repeated every 15 minutes, up to a maximum dose of 2000 mcg (2, 3, 4, 5, 6, 10, 11, 12, 13, 14). An alternative dosing regimen of 500 mcg intramyometrial route was cited in 6 guidelines (4, 5, 11, 12, 13, 14)Misoprostol was recommended either: as first line drug (5), when other first-line drugs unavailable or contraindicated (12), after oxytocin/ergometrine (2), or if carbroprost contraindicated (4). The recommended dosing regimen was a single dose of 600-1000 mcg, by oral, sublingual, or rectal route (2, 3, 4, 5, 6, 7, 10, 11, 12, 13, 14). Sulprostone IV route, 500 mcg administered over 1 hour (3, 6, 7)	Carboprost 250 mcg administered intramuscularly, followed by 250 mcg IM ergometrine if needed – considered a second line drug (21).<br>Misoprostol 800 mcg (4 tablets of 200 mcg) administered rectally. Considered a first line drug. Data drawn from seven trials, only one of which included some women with caesarean birth (21)
Tranexamic acid with standard care	The 2017 WHO guidelines on tranexamic acid recommended a fixed dose of 1 g (100 mg/mL) administered intravenously at 1 mL per minute (i.e., administered over 10 minutes), with a second 1 g IV dose if bleeding continued after 30 minutes OR restarted within 24 hours of completing the first dose.	Intravenous administration of 1 g over 10 minutes. A second dose may be administered after 30 minutes if bleeding persists (2, 3, 4, 5, 6, 7, 11, 12)	A fixed dose of 1g (100 mg/mL) intravenously at 1 ml per minute, within 3 hours of the time of diagnosis (if unknown, time of birth); a second dose of 1g given if needed 30 minutes from the first dose. Considered a first-line treatment (40).
**MANOEUVRES AND OTHER PROCEDURES**			
**Mechanical interventions**			
Uterine massage	Rubbing of the uterus achieved through manual massage of the abdomen, typically sustained until bleeding ceases or the uterus contracts. (initial rubbing of uterus and expression of clots NOT considered therapeutic uterine massage).<br>Recommended (low cost and relative safety of uterine massage considered in this recommendation).<br>Note: This recommendation was developed considering vaginal birth.	Intervention recommended in other 10 guidelines (2, 3, 4, 5, 7, 10, 11, 12, 14)	Not described.

Method	WHO recommendation (PPH 2012,TXA 2017, Carbetocin 2018, and UBT 2021) (1, 9, 38, 39)	Other Reviewed Guidelines	Systematic Reviews
Intrauterine balloon tamponade	The procedure entails insertion of a deflated/uninflated balloon into the uterine cavity and then inflating it to achieve a tamponade effect. Uterine balloon tamponade was recommended for the treatment of postpartum haemorrhage due to uterine atony after vaginal birth in women who did not respond to standard first-line treatment, provided all required resources for of PPH are available and routinely implemented (39). Only two studies included in the evidence supporting the recommendations included caesarean deliveries, both of which evaluated the effect of UBT in cases of placenta praevia or traumatic bleeding. One study (41)suggests that the use of the Bakri balloon could be more effective than haemostatic sutures in, and the second (42)suggests benefits associated to the use of the Bakri balloon held in place with a traction stitch versus Bakri balloon without traction stitch.	Guidelines described the urological Rusch balloon (left over 4-6 hrs), the Bakri SOS tamponade balloon catheter, the Sengstaken-Blakemore esophageal catheter, the Foley catheter, the polyurethane Ebb double balloon (vaginal and uterine), and the silicone BT-Cath tamponade balloon (2, 4, 6, 7, 10, 11, 13, 14)	One trial (50 women) compared Bakri Balloon with and without traction stitch; another trial (13 women) compared Bakri balloon to compressive suturing to the lower segment of the uterus. Not specified whether a first-line, second-line, or temporising treatment (20)
Uterine packing	Not recommended for PPH due to uterine atony.	One guideline (13) did not recommend the use of uterine packing while two guidelines recommended its use as a temporizing measure (14) or for unresponsive PPH (11).	Not described.
**Uterine-sparing surgical interventions and procedures**			
Compressive sutures	Compression suturing that runs through the full thickness of both uterine walls. When tied, the suture allows tight compression of the uterine walls and stops the bleeding. No specific suturing technique (e.g., B-Lynch, Hayman, Pereira). Recommended as first-line surgical intervention.	The B-Lynch technique was the most common uterine compression technique for atony (2, 3, 4, 5, 7, 10, 11, 12, 13, 14); however, other techniques, such as Cho and Hayman, were also recommended and described (10, 11).	One trial (160 women) compared the standard B-Lynch suture to a modified B-Lynch suture. Considered a second-line treatment (20).
Devascularisation / Artery ligation	Vascular flow to the uterus can be interrupted by uterine devascularization, ligation of the uterine or internal iliac arteries Recommended only if all available conservative measures (uterotonics, uterine massage, balloon tamponade) have failed.	If compression sutures are unsuccessful, bilateral uterine artery ligation, bilateral utero-ovarian artery ligation or -If expertise available- bilateral internal iliac artery ligation must be considered (2, 3, 4, 5, 6, 7, 10, 11, 12, 14). A common first approach is bilateral uterine artery ligation (O'Leary sutures) (11).	One trial (23 women) compared uterine artery embolization to surgical devascularization plus B-Lynch compression sutures. Not specified wither first-response, second-line, or temporizing treatment (20).

Method	WHO recommendation (PPH 2012,TXA 2017, Carbetocin 2018, and UBT 2021) (1, 9, 38, 39)	Other Reviewed Guidelines	Systematic Reviews
Uterine artery embolisation (UAE)	If other measures have failed and if the necessary resources were available, the use of uterine artery embolization was recommended as a treatment for PPH due to uterine atony.	Twelve guidelines recommended UAE (1, 2, 3, 4, 5, 6, 7, 10, 11, 12, 13, 14).<br>Only one describes the intervention in greater detail(3)	One trial (23 women) compared uterine artery embolization to surgical devascularization plus B-Lynch compression sutures. Not specified wither first-line, second-line, or temporizing treatment (20).
**Temporising**			
External aortic compression	Recommended as a temporizing measure until appropriate care is available, in PPH due to uterine atony.	In addition to WHO guidelines, seven guidelines recommended external aortic compression (Queensland, (2, 4, 6, 7, 10, 12, 14)	Not described in Systematic Reviews.
Non-pneumatic anti-shock garment	Recommended as a temporizing measure until appropriate care is available.	Recommended in other three guidelines (6, 10, 13).	One systematic review did not find a reduction in maternal mortality associated to NASG in the one cluster-RCT (880 women) included, However, 5 comparative studies (pre-intervention to intervention) included in this review (2330 women) suggested a clinically important reduction in maternal mortality and severe maternal morbidity(43). No effect was observed on the use of blood products. There were no safety issues in all the trials (43).

*Pileggi-Castro 2015 (43) is a systematic review on the non-pneumatic anti-shock garment as a treatment for severe PPH. This review was not captured as part of the overview review because the word “cesarean”/”caesarean” did not appear in the text. After consultation with Prof. Suellen Miller, who participated in each of the primary studies, confirmed that women with caesarean section were included in the original trials, data from this systematic review was added to the report and is reflected in the following tables.

Table S5. First and second round ratings and agreement for early detection methods for intraoperative and postoperative CB-PPH

Blood loss measurement and other PPH detection methods	**How would you rate each of the methods below for early detection of PPH considering…**							
	**the usefulness in managing patients?** 1=Not at all useful; 9=extremely useful	**feasibility in all settings performing CB?** 1=Hardly feasible; 9=Highly feasible	**its acceptability to key stakeholders?** 1=Not at all useful; 9=extremely useful	**the resources required?*** 1= very small; 9=very large	**the usefulness in managing patients?** 1=Not at all useful; 9=extremely useful	**feasibility in all settings performing CB?** 1=Hardly feasible; 9=Highly feasible	**its acceptability to key stakeholders?** 1=Not at all useful; 9=extremely useful	**the resources required?*** 1= very small; 9=very large
	**FIRST ROUND** Median (DI)				**SECOND ROUND** Median (DI)			
**INTRAOPERATIVE**								
Volumetric/gravimetric + clinical signs of haemodynamic instability	**8.0 (-0.93)**	6.0 (2.35)	NA	NA	NA	**8.0 (-3.08)**	NA	NA
Volumetric	**8.0 (-0.71)**	6.5 (3.50)	7.0 (2.35)	6.5 (1.85)	NA	6.5 (10.00)	7.0 (10.0)	7.0 (2.35)
Clinical signs of haemodynamic instability	6.5 (8.31)	**8.0 (-1.94)**	**8.0 (-0.22)**	**3.5 (0.58)**	**7.0 (-0.71)**	NA	NA	NA
Volumetric + gravimetric	**7.0 (-1.26)**	**5.0 (0.94)**	NA	NA	NA	NA	NA	NA
Clinical judgement such as rate of flow and duration	**5.0 (0.92)**	**7.0 (-21.7)**	6.0 (2.35)	**2.0 (0.49)**	NA	NA	7.0 (10.0)	NA
Visual charts and early warning scores (EWS)	**5.0 (0.88)**	6.0 (2.09)	6.0 (1.96)	5.0 (1.70)	NA	7.0 (10.0)	6.5 (10.0)	6.0 (1.74)
Visual estimation of blood loss	**4.5 (0.97)**	**8.5 (-1.81)**	7.0 (30.0)	**1.0 (0.13)**	NA	NA	**7.0 (-1.94)**	NA
Gravimetric	6.0 (2.35)	**4.5 (0.91)**	**5.0 (0.85)**	6.0 (2.09)	7.0 (10.00)	NA	NA	7.0 (10.00)
Visual estimation + visual charts/EWS	6.0 (1.48)	**7.0 (-0.71)**	NA	NA	6.5 (2.35)	NA	NA	NA
**POSTOPERATIVE**								
Clinical signs of haemodynamic instability	**8.0 (-3.08)**	**8.0 (-1.27)**	**8.0 (-1.94)**	**3.5 (0.58)**	NA	NA	NA	NA
Volumetric/gravimetric + clinical signs of haemodynamic instability	**7.0 (-0.93)**	6.0 (1.74)	NA	NA	NA	7.0 (30.00)	NA	NA
Clinical judgement such as rate of flow and duration	**5.0 (0.41)**	6.0 (2.35)	6.0 (2.35)	**2.0 (0.49)**	NA	6.0 (2.35)	7.0 (10.00)	NA
Gravimetric	5.5 (2.35)	**3.0 (0.65)**	**5.0 (0.85)**	6.0 (2.09)	**5.0 (0.63)**	NA	NA	7.0 (10.00)
Volumetric	5.0 (2.09)	**4.5 (0.58)**	7.0 (2.35)	6.5 (1.85)	**5.0 (0.63)**	NA	7.0 (10.00)	7.0 (2.35)
Visual estimation of blood loss	**4.0 (0.52)**	**7.0 (-3.08)**	7.0 (30.0)	**1.0 (0.13)**	NA	NA	**7.0 (-1.94)**	NA
Visual charts and early warning scores (EWS)	6.0 (2.09)	7.0 (2.35)	6.0 (1.96)	5.0 (1.70)	7.0 (10.0)	7.0 (10.00)	6.5 (10.00)	6.0 (1.74)
Visual estimation + visual charts/EWS	6.0 (4.96)	**7.0 (-3.08)**	NA	NA	7.0 (10.0)	NA	NA	NA
Volumetric + gravimetric	7.0 (8.31)	4.0 (1.04)	NA	NA	7.0 (10.0)	**5.0 (0.85)**	NA	NA

Note: A DI < 1 represented agreement, while a DI ≥ 1 indicated disagreement. Results in which agreement is reached are highlighted in bold.NA= Not applicable given that this combination of methods was not rated in the first round for acceptability to key stakeholders and the estimate of resources required, or because agreement was obtained in the first round. * The measurement scale for this criterion is the same as for the other criteria (from 1 to 9). However, unlike the other criteria, low values have a positive interpretation (few resources required) while high values have a negative interpretation (substantial resources required).

Table S6. First round ratings and agreement on threshold to initiate treatment for intraoperative CB PPH

Thresholds for triggering action	**How would you rate each of the following thresholds for managing PPH during CB considering…**		
	**the accuracy of each threshold?** 1=Hardly accurate; 9=Highly accurate	**its feasibility to be used in all settings?** 1=Hardly feasible; 9=Highly feasible	**its acceptability to key stakeholders?** 1=Hardly accepted; 9=Highly accepted
**INTRAOPERATIVE**			
**One-step approach (trigger full response protocol)**			
At least 1000 mL blood loss OR signs of haemodynamic instability, whichever comes first	**9 (-0.34)**	**8 (-0.34)**	**8 (-0.54)**
At least 1000 ml (blood loss alone, regardless of signs of haemodynamic instability)	**7 (-0.71)**	**8 (-0.71)**	**7 (-2.30)**
Haemodynamic instability alone, regardless of volume of blood loss	**7 (-3.08)**	**7 (-0.71)**	**7 (-3.08)**
At least 500 mL blood loss OR signs of haemodynamic instability, whichever comes first	7 (2.30)	**7 (-4.00)**	6.5 (8.31)
At least 500 mL (blood loss alone, regardless of signs of haemodynamic instability)	**5 (0.41)**	6.5 (2.14)	**5 (0.88)**
**Two-step approach (Lower threshold triggers further assessment, preparedness, and close monitoring; Higher threshold triggers treatment initiation)**			
Lower threshold of blood loss at least 500 mL (blood loss alone, regardless of signs of haemodynamic instability), and higher threshold of blood loss at least 1000 mL blood loss OR signs of haemodynamic instability, whichever comes first	**8 (-0.71)**	**8 (-0.71)**	**7 (-0.71)**
Lower threshold of blood loss at least 1000 ml (blood loss alone, regardless of signs of haemodynamic instability), and higher threshold of blood loss at least 2000 mL blood loss OR signs of haemodynamic instability whichever comes first	6.5 (4.96)	**8 (-4.23)**	7 (30.00)
**POSTOPERATIVE**			
**One-step approach (trigger full response protocol**			
At least 1000 mL blood loss OR signs of haemodynamic instability, whichever comes first	**8.5 (-0.34)**	**8 (-2.14)**	**8 (-0.88)**
At least 1000 ml (blood loss alone, regardless of signs of haemodynamic instability)	**7 (-0.71)**	**8 (-0.71)**	**7 (-1.27)**
Haemodynamic instability alone, regardless of volume of blood loss	**7 (-3.08)**	**7 (-4.00)**	7 (10.00)
At least 500 mL blood loss OR signs of haemodynamic instability, whichever comes first	7 (8.31)	**7 (-16.8)**	6.5 (30.00)
At least 500 mL (blood loss alone, regardless of signs of haemodynamic instability)	5 (1.47)	6 (2.09)	5 (1.18)
**Two-step approach (Lower threshold triggers further assessment, preparedness, and close monitoring; Higher threshold triggers treatment initiation)**			
Lower threshold of blood loss at least 500 mL (blood loss alone, regardless of signs of haemodynamic instability), and higher threshold of blood loss at least 1000 mL blood loss OR signs of haemodynamic instability, whichever comes first	**8 (-0.71)**	**8 (-0.71)**	**7.5 (-0.71)**

Lower threshold of blood loss at least 1000 ml (blood loss alone, regardless of signs of haemodynamic instability), and higher threshold of blood loss at least 2000 mL blood loss OR signs of haemodynamic instability whichever comes first	6.5 (4.41)	6 (2.35)	6 (1.96)

Note: A DI < 1 represented agreement, while a DI ≥ 1 indicated disagreement. Results in which agreement is reached are highlighted in bold. CB= caesarean birth.

Table S7. First round ratings and agreement for first response interventions for managing intraoperative and postoperative CB-PPH

First response interventions for managing PPH during and after CB	**How would you rate each intervention for first response management of CB-PPH considering…**				
	**the balance of effects?** 1=Weighted towards undesirable effects; 9=Weighted towards desirable effects	**the resources required? *** 1= very few; 9=very many	**its feasibility?** 1=Hardly feasible; 9=Highly feasible	**its acceptability to stakeholders?** 1=Hardly accepted; 9=Highly accepted	**equity?** 1=Likely to exacerbate inequities; 9=Highly likely to reduce inequities
	Median				
INTRAOPERATIVE					
Oxytocin	**9 (-0.34)**	3 (5.86)	**9 (-0.34)**	**9 (0.00)**	**9 (0.00)**
Carbetocin	**8 (-0.93)**	5 (1.81)	**7.5 (-3.79)**	**8 (-0.65)**	6 (6.55)
TXA	**8 (-0.34)**	**4 (0.76)**	**7 (-4.00)**	**8 (-0.93)**	**8 (-3.79)**
Compressive sutures	7 (2.9)	7 (4.64)	6 (4.71)	5.5 (1.76)	6 (4.71)
Bimanual compression	7 (10.15)	**3 (0.64)**	**7.5 (-3.08)**	6.5 (12.8)	**8 (-2.56)**
Uterine massage	**7 (-15.2)**	2 (1.17)	**8.5 (-0.65)**	**8 (-0.93)**	8 (-0.34)
Oxytocin-ergometrine fixed dose	6 (2.35)	4 (1.61)	**7 (-3.08)**	**8 (-3.08)**	**8 (-2.19)**
Prostaglandin (including sublingual misoprostol)	6 (1.37)	**3 (0.87)**	**8 (-3.79)**	**8 (-2.14)**	**8 (-0.93)**
Ergometrine	6 (4.00)	**3 (0.91)**	**8 (-0.92)**	**7 (-0.92)**	**8 (-0.34)**
Non-pneumatic anti-shock garment	5 (1.04)	**6 (0.78)**	**4 (0.52)**	5 (1.08)	5 (1.00)
External aortic compression	**5 (0.56)**	**2 (0.75)**	6 (30.00)	**5 (0.78)**	7 (30.00)
Intrauterine balloon tamponade	4 (1.61)	5 (2.14)	**4 (0.91)**	**5 (0.85)**	6 (4.22)
POSTOPERATIVE					
Oxytocin	**9 (-0.34)**	3 (5.86)	**9 (-0.34)**	**9 (0.00)**	**9 (0.00)**
TXA	**8 (-0.34)**	**4 (0.76)**	**7 (-13.00)**	**8 (-0.93)**	**8 (-6.78)**
Non-pneumatic anti-shock garment	6 (1.35)	6 (1.73)	**4 (0.52)**	**5 (0.99)**	5 (1.00)
Carbetocin	**6 (-10.29)**	5 (1.81)	**7.5 (-8.76)**	**8 (-0.92)**	5.5 (4.22)
Oxytocin-ergometrine fixed dose	6 (2.05)	4 (1.61)	**7 (-3.08)**	**8 (-3.08)**	**8 (-1.54)**
Uterine massage	6 (2.35)	3 (1.17)	**8 (-0.93)**	**8 (-1.68)**	**8 (-3.79)**
Ergometrine	6 (4.00)	**3 (0.97)**	**8 (-0.71)**	**7.5 (-2.19)**	**8 (-0.34)**
Prostaglandin (including sublingual misoprostol)	**6 (0.89)**	3 (1.35)	**8 (-1.53)**	**7.5 (-2.14)**	**8 (-0.93)**
Bimanual compression	**5 (0.63)**	**3 (0.41)**	6 (16.57)	6 (1.73)	**8 (-23.00)**
External aortic compression	**5 (0.68)**	**2 (0.68)**	6 (2.25)	**5.5 (0.52)**	7 (5.6)
Intrauterine balloon tamponade	4 (1.64)	5 (2.14)	**4.5 (0.91)**	**5 (0.85)**	4.5 (4.22)
Compressive sutures	**2 (0.89)**	6 (2.84)	**4 (0.99)**	**3 (0.91)**	4.5 (1.59)

Note: A DI < 1 represented agreement, while a DI ≥ 1 indicated disagreement. Results in which agreement is reached are highlighted in bold. CB= caesarean birth. * The measurement scale for this criterion is the same as for the other criteria (from 1 to 9). However, unlike the other criteria, low values have a positive interpretation (few resources required) while high values have a negative interpretation (substantial resources required)

Pingray V, *et al. BMJ Open* 2024; 14:e079713. doi: 10.1136/bmjopen-2023-079713

Table S8. Second round ratings and agreement for first response interventions for managing intraoperative and postoperative CB-PPH

**Intraoperative**	Median (RAND DI)
Examine and rapidly initiate cause-specific first response (e.g., if trauma: rapid surgical haemostasis; if atony/placental cause: uterotonics and uterine massage)	**9 (-0.34)**
TXA for all women with PPH during CB regardless of aetiology	**8 (-0.43)**
Plasma expansion with crystalloids or all women with PPH during CB regardless of aetiology	**7.5 (-1.94)**
Uterotonics for all women with PPH during CB regardless of aetiology	**7 (-0.71)**
**Postoperative**	
Examine and rapidly initiate cause-specific first response (e.g., if trauma: rapid surgical haemostasis; if atony/placental cause: uterotonics and uterine massage)	**9 (-0.34)**
TXA for all women with PPH during CB regardless of aetiology	**8 (0.00)**
Plasma expansion with crystalloids or all women with PPH during CB regardless of aetiology	**7.5 (-0.71)**
Uterotonics for all women with PPH during CB regardless of aetiology	**7 (-0.71)**

Note: A DI < 1 represented agreement, while a DI ≥ 1 indicated disagreement. Results in which agreement is reached are highlighted in bold. CB= caesarean birth.

Pingray V, *et al. BMJ Open* 2024; 14:e079713. doi: 10.1136/bmjopen-2023-079713

Supplementary Figures

Figure S1. Interpretation of DI and RAND/UCLA Appropriateness scale

DI (Disagreement index)	Experts' median rating								
	1	2	3	4	5	6	7	8	9
	Bottom third (1-3)			Intermediate third (4-6)			Top third (1-3)		
<1 (Agreement)	Inappropriate			Uncertain			Appropriate		
≥1 (Disagreement)	Uncertain								

Figure S2. PRISMA Flowchart

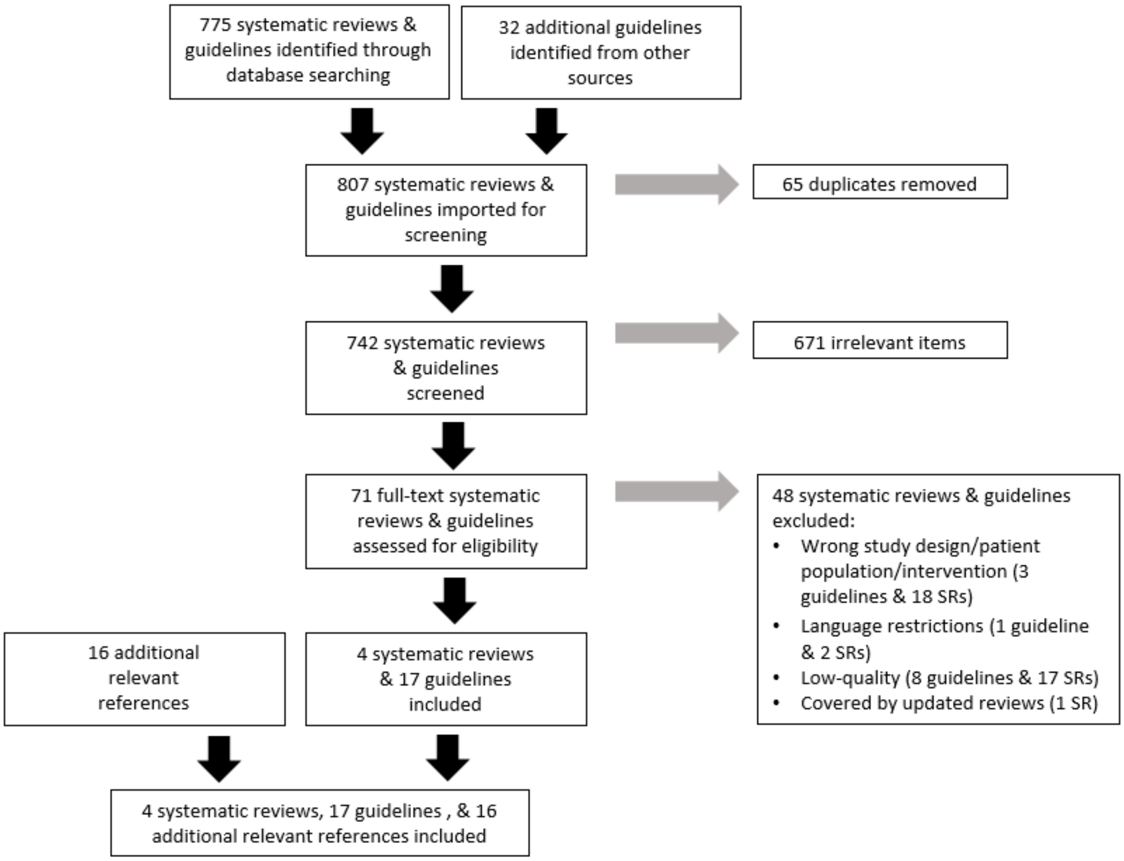

Figure S3. Second round ranking of one-step thresholds to initiate treatment for intraoperative and postoperative CB PPH

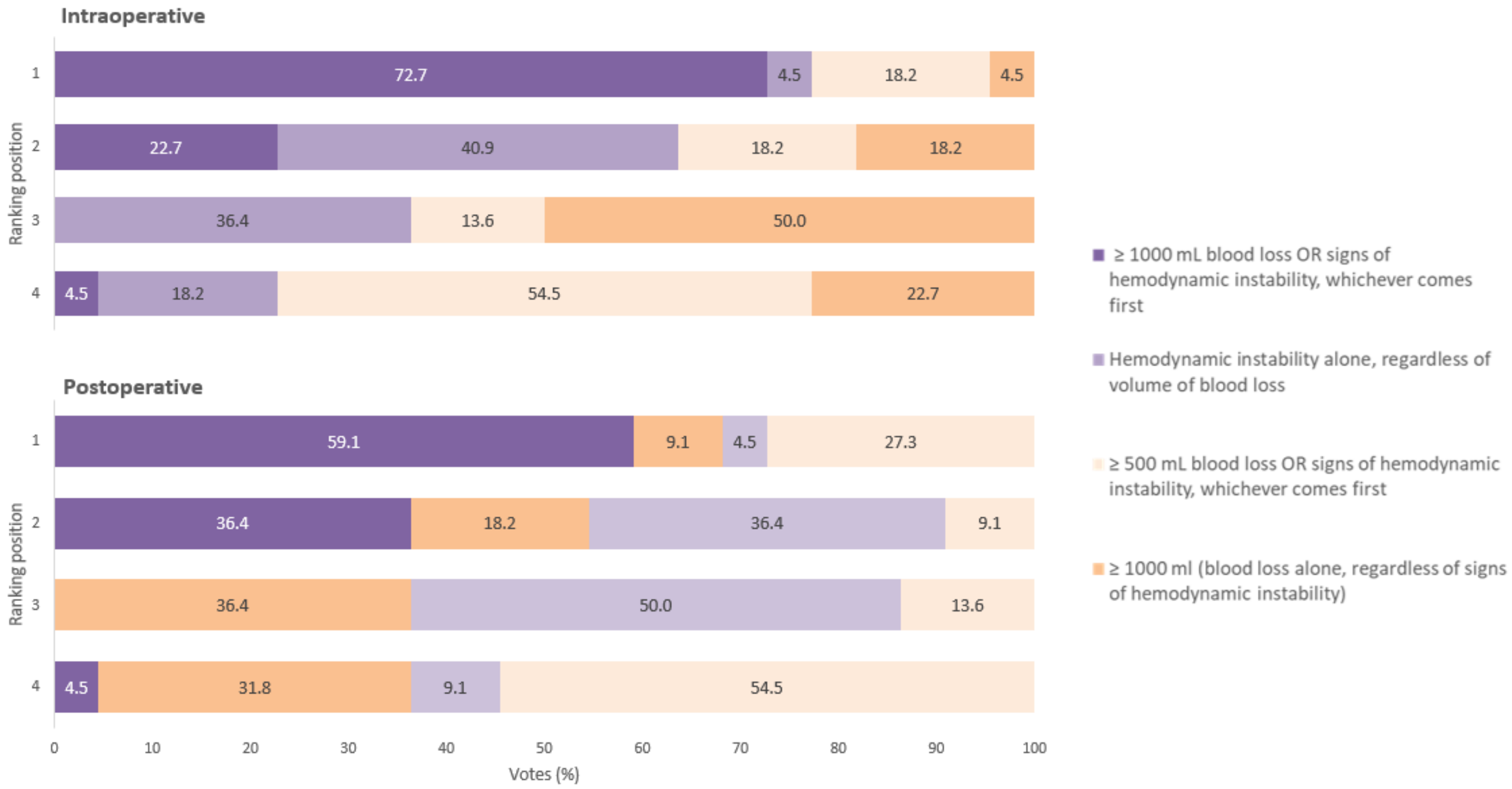

Note: CB= caesarean birth

Supplementary Files

Supplementary File S1. Search strategies for each database

CINAHL (EBSCO) 30-06-2020

#	Query
S23	S20 AND S21<br>Limiters - Published Date: 20120101-20201231
S22	S20 AND S21
S21	TI (Systematic N1 Review) OR AB (Systematic N1 Review) OR TI Meta-Analys* OR AB Meta-Analys* OR AB Cochrane OR TI Metaanalysis OR AB Metaanalysis OR TI Metanalysis OR AB Metanalysis OR (AB MEDLINE AND AB Cochrane) OR Guideline*[ti] OR TI (Guide N1 Line*) OR AB (Guide N1 Line*) OR TI Consensus OR AB Consensus OR TI Recommendation* OR TI (Technology N1 Assessment) OR AB (Technology N1 Assessment) OR TI (Technology N1 Appraisal) OR AB (Technology N1 Appraisal) OR TI HTA OR AB HTA OR TI Overview OR (TI Review AND TI Literature))
S20	S10 AND S19
S19	S11 OR S12 OR S13 OR S14 OR S15 OR S16 OR S17 OR S18
S18	TI Caesarea* OR AB Caesarea*
S17	TI Caesaria* OR AB Caesaria*
S16	TI Cesaria* OR AB Cesaria*
S15	TI Cesarea* OR AB Cesarea*
S14	TI Cesaerea* OR AB Cesaerea*
S13	TI (C N1 Section*) OR AB (C N1 Section*)
S12	TI C-Section* OR AB C-Section*
S11	(MH "Cesarean Section+")
S10	S1 OR S2 OR S3 OR S4 OR S5 OR S6 OR S7 OR S8 OR S9
S9	TI (Blood N1 Collection) OR AB (Blood N1 Collection)
S8	TI (Blood N1 Specimen) OR AB (Blood N1 Specimen)
S7	TI Bleeding OR AB Bleeding
S6	TI (Blood N2 Loss) OR AB (Blood N2 Loss)
S5	(MH "Blood Specimen Collection+")
S4	TI PPH* OR AB PPH*

S3	TI Haemorrhag* OR AB Haemorrhag*
S2	TI Hemorrhag* OR AB Hemorrhag*
S1	(MH "Postpartum Hemorrhage")

COCHRANE LIBRARY 30-04-2020

ID

#1 MeSH descriptor: [Postpartum Hemorrhage] explode all trees

#2 Hemorrhag*:ti,ab,kw

#3 Haemorrhag*:ti,ab,kw

#4 PPH*:ti,ab,kw

#5 MeSH descriptor: [Blood Specimen Collection] explode all trees

#6 (Blood NEAR/1 Loss):ti,ab,kw

#7 Bleeding:ti,ab,kw

#8 (Blood NEAR/1 Specimen):ti,ab,kw

#9 (Blood NEAR/1 Collection):ti,ab,kw

#10 #1 OR #2 OR #3 OR #4 OR #5 OR #6 OR #7 OR #8 OR #9

#11 MeSH descriptor: [Cesarean Section] explode all trees

#12 C-Section*:ti,ab,kw

#13 Cesaerea*:ti,ab,kw

#14 Cesarea*:ti,ab,kw

#15 Cesaria*:ti,ab,kw

#16 Caesaria*:ti,ab,kw

#17 Caesarea*:ti,ab,kw

#18 #12 OR #13 OR #14 OR #15 OR #16 OR #17

#19 #10 AND #18 with Cochrane Library publication date Between Jan 2012 and Apr 2020, in Cochrane Reviews

PUBMED (NLM) 29-04-2020

	Query
#23	**(#20 AND #21)** Filters: **Publication date from 2012/01/01**
#22	**(#20 AND #21)**
#21	**(((Systematic Review[sb] OR Systematic Review[tiab] OR Meta-Analysis[pt] OR Meta-Analys*[tiab] OR "Cochrane Database Syst Rev"[ta] OR Metaanalysis[tiab] OR Metanalysis[tiab] OR Overview[ti] OR (Review[ti] AND Literature[ti]) OR (MEDLINE[tiab] AND Cochrane[tiab]) OR Guideline[pt] OR Practice Guideline[pt] OR Guideline*[ti] OR Guide Line*[tiab] OR Consensus[tiab] OR Recommendation*[ti])))**
#20	**(#11 AND #19)**
#19	**(#12 OR #13 OR #14 OR #15 OR #16 OR #17 OR #18)**
#18	**Caesarea*[tiab]**
#17	**Caesaria*[tiab]**
#16	**Cesaria*[tiab]**
#15	**Cesarea*[tiab]**
#14	**Cesaerea*[tiab]**
#13	**C-Section*[tiab]**
#12	**Cesarean Section[Mesh]**
#11	**((#1 OR #2 OR #3 OR #4 OR #5 OR #6 OR #7 OR #8 OR #9 OR #10))**
#10	**Blood Collection[tiab]**
#9	**Blood Specimen[tiab]**
#8	**Bleeding[tiab]**
#7	**Loss of Blood"[tiab]**
#6	**Blood Loss[tiab]**
#5	**Blood Specimen Collection[Mesh]**
#4	**PPH*[tiab]**
#3	**Haemorrhag*[tiab]**
#2	**Hemorrhag*[tiab]**
#1	**Postpartum Hemorrhage[Mesh]**

EMBASE (ELSEVIER) 29-04-2020

No. Query Results Results Date

#22. #21 AND (2012:py OR 2013:py OR 2014:py OR 2015:py

OR 2016:py OR 2017:py OR 2018:py OR 2019:py OR 2020:py)

#21. #19 AND #20

#20. (('systematic review':ti,ab OR 'meta analysis (topic)':pt OR 'meta analysis':ti,ab OR cochrane:jt OR metaanalysis:ti,ab OR metanalysis.ti,ab. OR (medline:ab AND cochrane:ab) OR practice) AND guideline:pt OR guideline*:ti OR 'guide lines':ti,ab OR consensus:ti,ab OR recommendation*:ti OR biomedical) AND technology AND 'assessment'/exp OR 'technology assessment':ti,ab OR 'technology appraisal':ti,ab OR hta:ti,ab OR overview:ti OR (review:ti AND literature:ti)

#19. #10 AND #18

#18. #11 OR #12 OR #13 OR #14 OR #15 OR #16 OR #17

#17. caesarea*:ti,ab

#16. caesaria*:ti,ab

#15. cesaria*:ti,ab

#14. cesarea*:ti,ab

#13. cesaerea*:ti,ab

#12. (c NEAR/1 section*):ti,ab

#11. 'cesarean section'/exp

#10. #1 OR #2 OR #3 OR #4 OR #5 OR #6 OR #7 OR #8 OR #9

#9. (blood NEAR/1 collection):ti,ab

#8. (blood NEAR/1 specimen):ti,ab

#7. bleeding:ti,ab

#6. (blood NEAR/1 loss):ti,ab

#5. 'blood specimen collection kit'/exp

#4. pph*:ti,ab

#3. haemorrhag*:ti,ab

#2. hemorrhag*:ti,ab

#1. 'postpartum hemorrhage'/exp

LILACS (BVS-EN) 1-05-2020

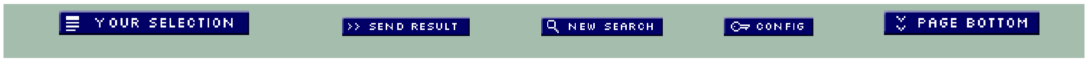

Database : **LILACS**

: **(MH Postpartum Hemorrhage OR Hemorrhag$ OR Hemorragia OR PPH$ OR Haemorrhag$ OR MH Blood Specimen Collection OR Sangrado OR Sangrando) AND (MH Cesarean Section OR C-Section$ OR Cesaerea$ OR Cesarea$ OR Cesaria$ OR Caesaria$ OR Caesarea$) [Words] and 2012 OR 2013 OR 2014 OR 2015 OR 2016 OR 2017 OR 2018 OR 2019 OR 2020 [Country, year publication]**

Supplementary File S2. Systematic literature review methods

Overview

The preceding evidence synthesis includes literature identified through two consecutive processes. First, we conducted an overview review of guidelines and systematic reviews. After synthesizing this evidence, we conducted complementary targeted searches for literature (relevant references) on specific topics to help round out the evidence base. For the purposes of this report, systematic reviews, guidelines, and relevant references were defined as follows:

Overview of Reviews: A systematic method for searching, identifying, and synthesizing systematic reviews

Guideline: A position paper of scientific or professional societies or state-based task groups, with recommendations based on medical evidence and consensus among authors.

Systematic review: A structured review of scientific evidence, with explicit criteria regarding literature search, inclusion and exclusion of articles.

Meta-analysis: A method for systematically combining quantitative data from multiple studies to analyze larger trends.

Other Relevant References: Scientific articles, which were identified via expert recommendation, were added to this evidence base and analyzed. These references were included to gain more insight about PPH detection methods.

Systematic search: A formalized approach to searching the literature with pre-specified search terms and criteria. Often, but not always, used as the first step in a systematic review.

Each of these processes are described in detail below.

Overview of reviews

We conducted an overview of reviews of guidelines, systematic reviews (including rapid, scoping, and umbrella systematic reviews), and meta-analyses reporting detection and first response interventions to suspected PPH during and within the first 24 hours following the caesarean section. As stated above, overview reviews generally are applied to systematic reviews of research questions for the purpose of extracting and analysing their results across important outcomes. In preparing this report, we adapted this methodology to also include guidelines and recommendations.

Search Strategy and Screening Process

A systematic search was conducted for peer-reviewed systematic reviews published from January 2012 to July 2020 in the following electronic databases: PubMed, EMBASE, CINAHL, and Cochrane Library. The

search was complemented by reviewing the grey literature published during the same period to identify guidelines and recommendations in repositories, websites, and national ministries of health from any English-speaking country and/or written in English.

Titles and abstracts of all identified sources were imported into Covidence (Covidence) for initial screening; those that were potentially eligible were selected for full-text review. Pairs of independent reviewers (CRW, FM, AL, AB, and VP) reviewed full texts. Systematic reviews which included analytic non-comparative studies (e.g., case series) were excluded.

Clarification: Whenever possible, definitions, detection methods, thresholds, and conservative treatments specific to PPH that develops during or after caesarean section were extracted from the guidelines. However, some guidelines (Definitions: 11/17 [65%]; Detection Methods: 16/17 [94%]; Thresholds: 9/17 [53%]; and Treatments: 16/17 [94%]) did not specify the mode of birth in PPH-related recommendations. Due to this, the included guidelines' summaries contain both a) recommendations specific to PPH during and after caesarean section and b) recommendations where the mode of birth is not specified. Therefore, some of the detection methods and treatments in this document may seem less suitable for intraoperative PPH than for postoperative PPH.

Quality appraisal

Following selection, pairs of independent reviewers (CRW, FM, AL, AB, and VP) assessed the quality of included full texts using the AGREE reporting checklist for guidelines and recommendations (44) and a modified version of the AMSTAR 2 quality assessment tool for systematic reviews (45). AGREE II is the new (2010) international tool to assess the quality and reporting of practice guidelines (44). AMSTAR 2 is an updated version of the AMSTAR tool initial developed by the Cochrane Collaboration in 2007 to aid in the critical appraisal of systematic reviews. It is one of the most widely-used such tools (45). Reviewers then met to discuss any conflicts. Disagreements were discussed until consensus was reached, and if required, a third reviewer was consulted.

The AGREE II reporting checklist was used as intended. The following modifications were made to the AMSTAR 2 quality assessment tool.

Using the AMSTAR 2 tool, all systematic reviews are assessed on 16 total domains (see Appendix), which together encompass various aspects of systematic review quality, ranging from adequacy of the literature search to management of potential risk of bias in included primary studies. Each included systematic review is assessed individually. Once the overall assessment is complete, the AMSTAR 2 authors propose a scoring system for developing an overall quality rating for the systematic review (45). Under the proposed scoring system, 7 domains are weighted more heavily than the others. These more heavily weighted domains are considered "critical domains" and inadequate responses in these domains are considered "critical flaws."

Under the AMSTAR 2 scoring system, the quality of assessed systematic reviews is determined based on the number of critical and non-critical flaws in the review:

- High: No or one non-critical flaw
- Moderate: More than one non-critical flaw
- Low: One critical flaw
- Critical low: More than one critical flaw

The AMSTAR 2 authors propose seven items (2, 4, 7, 9, 11, 13, and 15) as critical domains. Upon discussion by a panel of experts and the systematic review team, it was decided that only four items (4, 7, 9, and 13) were relevant to our systematic review. These items are:

- Adequacy of the literature search (item 4)
- Justification for excluding individual studies (item 7)
- Risk of bias from individual studies being included in the review (item 9)
- Consideration of risk of bias when interpreting the results of the review (item 13)

Items determined to be irrelevant for our systematic review were item 2 (protocol registered before commencement of the review; since all included systematic reviews were Cochrane Reviews this criterion was met), item 11 (appropriateness of meta-analytical methods; since no meta-analysis was conducted as part of our overview review), and item 15 (assessment of presence and likely impact of publication bias).

Only guidelines with AGREE II scores between 5-7 (AGREE scores range from 0-7) and systematic reviews with modified-AMSTAR quality assessment of Moderate or High were eligible for data extraction.

Outcomes

Main outcome(s)

We will list and describe the frequency and characteristics of reported and recommended interventions for the detection and initial management of caesarean section related PPH.

Additional outcome(s)

In addition, we will describe reported caesarean section PPH definitions, methods for blood loss estimation or measurement, clinical criteria to diagnose PPH and thresholds.

Interventions will be classified according to the time of occurrence: intra or post-operative PPH, and the country and world group (Low income, Lower middle income, Upper middle income or High income) that released the recommendation.

Data Extraction and Synthesis

Each selected systematic review was independently extracted by two reviewers (CRW and FM). Data on PPH definitions, thresholds, detection methods, and treatments were extracted using a common template. Following independent extraction, the reviewers met to discuss any conflicts until consensus was reached, with a third reviewer (VP) joining to resolve conflicts as needed. Final decisions on data extraction were captured in a consolidated database.

Each selected guideline was reviewed by two independent reviewers (AL, AB, and VP). Data on PPH definitions, thresholds, detection methods and treatments were extracted using a common template. Following independent extraction, the reviewers met to discuss any conflicts until consensus was reached. Final decisions on data extraction were captured in a consolidated database. Data were synthesized into descriptive tables based on each of the four topic areas. A brief description of included studies is included in the “Findings” section of this document, with synthesized findings organized by topic area provided in the preceding Evidence Synthesis.

Additional Relevant Resources

Search Strategy and Screening Process

An additional search for peer-reviewed manuscripts was conducted using PubMed with the following search terms: 'Postpartum Hemorrhage' AND 'Detection Methods'. Peer-reviewed RCTs were eligible for inclusion. Subject matter experts were also consulted to add any relevant peer-reviewed articles missed by the systematic search.

Data Extraction and Narrative Synthesis

Each relevant resource article identified through the additional search was reviewed and narratively synthesized by a single member of the team (AL or AB). A second member of the team (AB, VP, or CRW) reviewed the full article, and then reviewed the drafted synthesis. Multiple iterative rounds of revision occurred between the two reviewers until they reached consensus. Narrative syntheses were then added to the corresponding report tables.

Study Flowchart

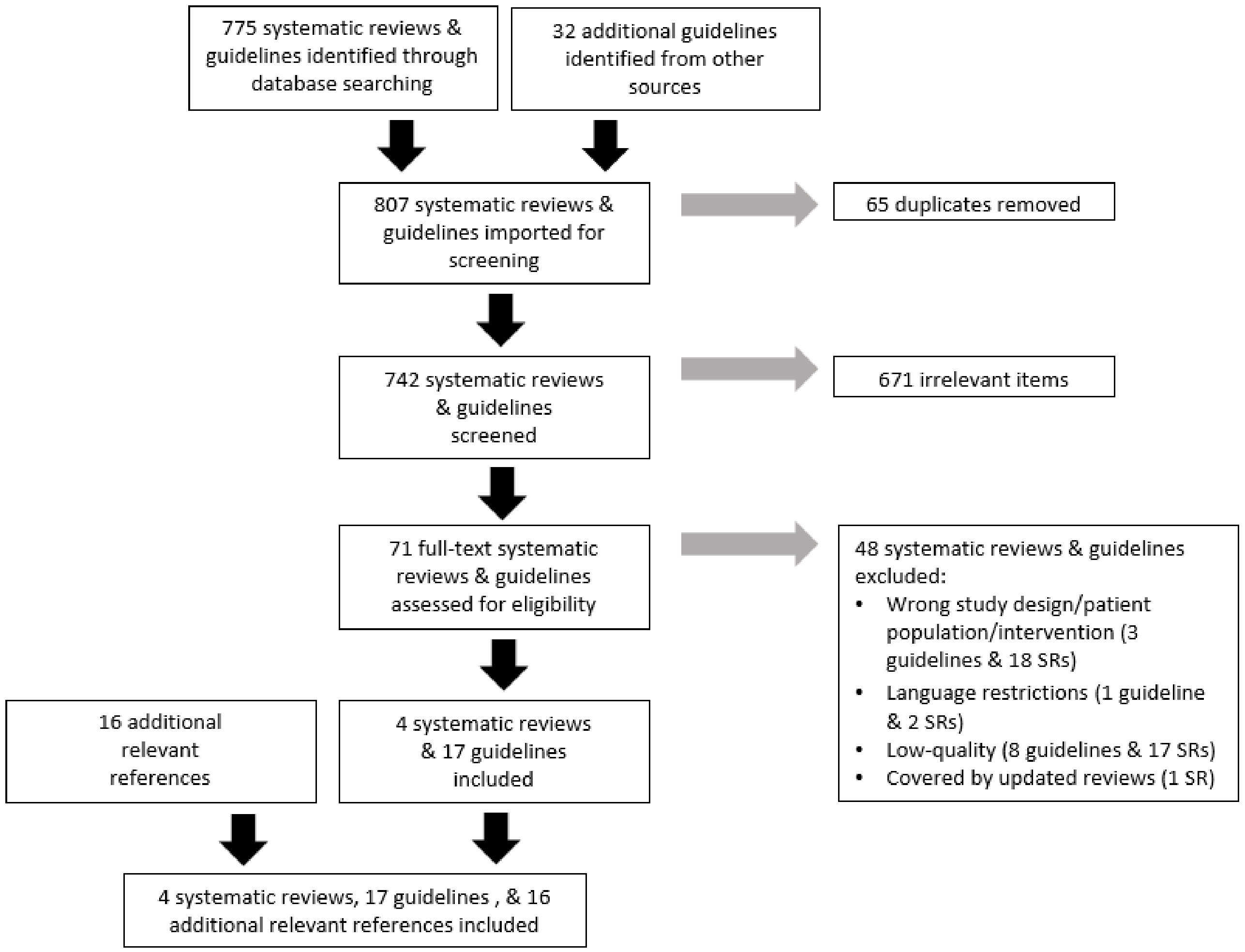

Characteristics of included guidelines and systematic reviews

Guidelines and recommendations

The overview review included 17 guidelines with an overall AGREE II score of 5-7 (scale 0-1) from professional bodies (n=11), the WHO (n=3), government organizations (n=2), and a consortium (n=1). Of the 17 guidelines, 14 were guidelines specific to PPH; two covered general obstetric practice, and one was on caesarean section. Five guidelines were published or updated between 2012-2015 and 12 between 2015-2019. Of the 17, four guidelines were international in scope (WHO, FIGO), while 13 were developed for audiences in high-income countries (HIC). See tables below on Quality appraisal of included guidelines and Main characteristics of included guidelines.

Systematic reviews

The overview review also included four Cochrane systematic reviews, published between 2015 and 2020 See tables below on Quality appraisal of included systematic reviews and Main characteristics of included systematic reviews. All systematic reviews included data on women with PPH following both caesarean section and vaginal birth. The four Cochrane systematic reviews together constitute an update of a single previous Cochrane systematic review from 2014 on the treatment of primary postpartum haemorrhage (46). Although the initial assessment met inclusion criteria, as updated reviews from 2018 and 2020 were available, the initial review was excluded, and the four updated reviews were included instead.

Shakur 2018 was a Cochrane systematic review on antifibrinolytic drugs to treat primary PPH. This study included data across 3 RCTs and included high-, middle-, and low-income countries (40). Gallos 2018 was a Cochrane systematic review and network meta-analysis on uterotonics agents for preventing PPH (19). Although this review was focused on PPH prevention, not first-response management, it is included as one of four Cochrane systematic reviews that together constituted the updating of Mousa 2014 (46). In addition, Gallos 2018 included information on definitions, thresholds, and blood collection and measurement techniques. Gallos 2018 included 196 RCTs from 53 countries. Kellie 2020 was a Cochrane systematic review that compared mechanical and surgical interventions for treating PPH, including comparisons of each technique plus standard care versus standard of care alone, as well as head-to-head comparisons of different techniques. The review included 9 RCTs and data from 7 LMICs (20). The final Cochrane systematic review and network meta-analysis (Parry Smith 2020) surveyed the evidence of uterotonics agents for first-line treatment of PPH (21). This review included 7 RCTs from 10 LMICs. Nearly all data was drawn from women with vaginal births; a small subset of women in one of the included RCTs (Lokugamage 2001) gave birth via caesarean section.

Additional relevant references

Additional relevant references were taken into consideration for Chapter 2 of the Evidence Synthesis: Detection Methods of PPH during and after caesarean birth. Sixteen additional peer-reviewed primary studies were identified through the methodology described above. Given the heterogeneity of these studies, a systematic approach to data extraction was not undertaken;

rather, the studies were reviewed and narratively synthesized by one member of the research team, with a second member of the research team subsequently reviewing the synthesis for accuracy and completeness. Information from these syntheses was then added to Table 2.2 in the Evidence Synthesis.

Quality appraisal of included guidelines

Organization	Title	Year of publication	Percentage of the maximum score							Final Overall Score
			Domain 1	Domain 2	Domain 3	Domain 4	Domain 5	Domain 6	OA1 - Total	
WHO	WHO recommendations for the prevention and treatment of postpartum haemorrhage	2012	100%	100%	95%	100%	81%	88%	100%	7
WHO	WHO recommendation on tranexamic acid for the treatment of postpartum haemorrhage	2017	100%	100%	100%	83%	88%	100%	100%	7
NICE	Caesarean section	2019	100%	86%	100%	100%	96%	75%	100%	7
RCOG	Prevention and Management of Postpartum Haemorrhage	2016	100%	86%	91%	100%	33%	75%	92%	7
WHO	Managing Complications in Pregnancy in Childbirth	2017	94%	100%	49%	100%	96%	100%	75%	6
RCPI; Health Service Executive	Prevention and management of primary postpartum haemorrhage	2014	97%	94%	75%	100%	85%	100%	92%	6
Queensland Health	Queensland Clinical Guidelines: Primary Postpartum Haemorrhage	2019	97%	81%	56%	100%	96%	92%	75%	6
FCNGOF	Postpartum hemorrhage: French College of Gynaecologists and Obstetricians	2016	57%	50%	64%	96%	47%	83%	72%	5

Organization	Title	Year of publication	Percentage of the maximum score							Final Overall Score
			Domain 1	Domain 2	Domain 3	Domain 4	Domain 5	Domain 6	OA1 - Total	
RANZCOG	Management of Postpartum Haemorrhage	2017	100%	89%	54%	100%	10%	81%	67%	5
SOGC	Active Management of the Third Stage of Labour: Prevention and Treatment of Postpartum Hemorrhage	2018	97%	64%	58%	100%	19%	88%	67%	5
DGGG, OEGGG and SGGG	Peripartum Haemorrhage, Diagnosis and Therapy	2016	94%	67%	64%	100%	29%	86%	67%	5
FIGO	Guidelines for Prevention and Treatment of Postpartum Hemorrhage in low resource settings	2012	86%	56%	44%	94%	94%	96%	67%	5
NATA	Patient blood management in obstetrics: prevention and treatment of postpartum haemorrhage	2016	94%	67%	58%	100%	21%	75%	67%	5
ACOG	Postpartum Hemorrhage	2017	98%	54%	60%	87%	58%	50%	67%	5
Standford and others	International consensus statement on the use of uterotonic agents during caesarean section	2019	100%	57%	36%	100%	18%	100%	67%	5

Organization	Title	Year of publication	Percentage of the maximum score							Final Overall Score
			Domain 1	Domain 2	Domain 3	Domain 4	Domain 5	Domain 6	OA1 - Total	
JSOG/JAOG	Guidelines for obstetrical practice in Japan	2014	93%	72%	28%	72%	7%	89%	61%	5
ACOG	Quantitative Blood Loss in Obstetric Hemorrhage	2019	83%	56%	39%	97%	50%	88%	58%	5

Quality appraisal of included systematic reviews

	Gallos 2018	Kellie 2020	Parry-Smith 2020	Shakur 2018
Overall Quality Assessment	HIGH	HIGH	HIGH	MODERATE
Item 1: PICO components described in research questions and inclusion criteria	Yes	Yes	Yes	Yes
Item 2: Evidence of pre-established protocol and explanation of any deviations	Yes	Yes	Yes	Yes
Item 3: Justification of included study designs	No	Yes	Yes	No
Item 4: Comprehensive literature search strategy, including consultation with content experts*	Partial	Partial	Partial	Partial
Item 5: Study selection performed in duplicate	Yes	Yes	Yes	Yes
Item 6: Data extraction performed in duplicate	Yes	Yes	Yes	Yes

	Gallos 2018	Kellie 2020	Parry-Smith 2020	Shakur 2018
Overall Quality Assessment	HIGH	HIGH	HIGH	MODERATE
Item 7: List of excluded studies and justification for exclusion*	Yes	Yes	Yes	Yes
Item 8: Adequate description of included studies	Yes	Yes	Yes	Yes
Item 9: Satisfactory technique used to assess risk of bias in included studies*	Yes	Yes	Yes	Yes
Item 10: Report of sources of funding for included studies	Yes	Yes	Yes	Yes
Item 11: Use of appropriate methods for statistical combination of results	Yes	N/A	No	Yes
Item 12: Assessment of potential impact of risk of bias on results of evidence synthesis	Yes	N/A	Yes	Yes
Item 13: Accounting for risk of bias from included studies in interpretation/discussion of results*	Yes	Yes	Yes	Yes
Item 14: Satisfactory explanation for, and discussion of, any observed heterogeneity	Yes	No	Yes	Yes
Item 15: Adequate investigation of publication bias	Yes	NA	No	No
Item 16: Reporting and management of potential conflicts of interest	Yes	Yes	Yes	Yes

*Considered a critical component of the authors' modified AMSTAR II ranking scale

Main characteristics of included guidelines

Organization1	Name of the Guideline	Year of publication or update	Geographical scope	Main topic
International Agencies				
WHO	WHO recommendation on tranexamic acid for the treatment of postpartum haemorrhage	2017	Global	PPH
WHO	Managing Complications in Pregnancy in Childbirth	2017	Global	Obstetric complications
WHO	WHO recommendations for the prevention and treatment of postpartum haemorrhage	2012	Global	PPH
Government Organizations				
NICE	Caesarean section	2019	UK	CS
Queensland Health	Queensland Clinical Guidelines: Primary Postpartum Haemorrhage	2019	Queensland, Australia	PPH
Professional Body				
ACOG	Quantitative Blood Loss in Obstetric Haemorrhage	2019	US	Quant. blood loss

[1] WHO (World Health Organization), NICE (National Institute for Health and Care Excellence), CMQCC (California Maternal Quality Care Collaborative), ACOG (American College of Obstetricians and Gynecologists), SOGC (Society of Obstetricians and Gynaecologists of Canada), RANZCOG (Royal Australian and New Zealand College of Obstetricians and Gynaecologists), NATA (Network for the Advancement of Patient Blood Management Haemostasis and Thrombosis), FCNGOF (French College of Gynaecologists and Obstetricians, DGGG (German Society of Gynecology and Obstetrics), OEGGG (Austrian Society of Gynecology and Obstetrics), Swiss Society of Obstetrics and Gynecology, RCOG (Royal College of Obstetrics and Gynaecology, HSE (Health and Safety Executive, JSOG/JAOG (Japan Society of Obstetrics and Gynecology, FIGO (The International Federation of Gynecology and Obstetrics)

Organization1	Name of the Guideline	Year of publication or update	Geographical scope	Main topic
SOGC	Prevention and Treatment of Postpartum Haemorrhage	2018	Canada	PPH
RANZCOG	Management of Postpartum Haemorrhage	2017	Australia/New Zealand	PPH
ACOG	Postpartum Haemorrhage	2017	US	PPH
NATA	Patient blood management in obstetrics: prevention and treatment of postpartum haemorrhage	2016	Global	PPH and Blood management
FCNGOF	Postpartum haemorrhage: French College of Gynaecologists and Obstetricians	2016	France	PPH
DGGG, OEGGG and SGGG	Peripartum Haemorrhage, Diagnosis and Therapy	2016	Germany	PPH
RCOG	Prevention and Management of Postpartum Haemorrhage	2016	UK	PPH
Royal College of Physicians of Ireland; HSE	Prevention and management of primary postpartum haemorrhage	2014	Ireland	PPH
JSOG/JAOG	Guidelines for obstetrical practice in Japan	2014	Japan	Obstetrical practice
FIGO	Guidelines for Prevention and Treatment of Postpartum Haemorrhage in low resource settings	2012	Global	PPH
Consortium				
CMQCC	Improving Health Care Response to Obstetric Haemorrhage (v2): A California Quality Improvement Toolkit	2015	US	PPH

Main characteristics of included systematic reviews

Author & Year	Title	Geographical scope	Number of Studies	Sample size
Shakur 2018	Antifibrinolytic drugs for treating primary postpartum haemorrhage	Argentina, Australia, Canada, Denmark, Finland, France, Hong Kong, Korea, Ireland, Israel, Italy, Japan, Netherlands, New Zealand, Sweden, UK, USA	3	20,412
Gallos 2018	Uterotonic agents for preventing postpartum haemorrhage: a network meta-analysis	Argentina, Australia, Austria, Bangladesh, Belgium, Canada, China, Colombia, Egypt, Ecuador, France, Ghana, Guinea Bissau, Hong Kong, Hungary, India, Indonesia, Iran, Ireland, Italy, Jamaica, Kenya, Korea, Kuwait, Libya, Malaysia, Mexico, Mozambique, Nepal, Netherlands, Nigeria, Norway, Panama, Pakistan, Papua, Philippines, Saudi Arabia, Senegal, Singapore, South Africa, Spain, Sweden, Switzerland, Thailand, Turkey, Tunisia, UAE, Uganda, UK, USA, Venezuela, Vietnam, West Indies, Zimbabwe	196	135,559
Kellie 2020	Mechanical and surgical interventions for treating primary postpartum haemorrhage	Benin, Egypt, Mali, Saudi Arabia, Thailand, Turkey, and Pakistan	9	994
Parry-Smith 2020	Uterotonic agents for first-line treatment of postpartum haemorrhage: a network meta-analysis	Argentina, Burkina Faso, Ecuador, Egypt, Gambia, Pakistan, South Africa, Thailand, Turkey, and Vietnam	7	3,738

ADDITIONAL EVIDENCE SYNTHESIS PROCESSES

Detection Methods Pre-Screening Process

The initial list of detection methods developed based on the literature review included several innovative methods that the research team deemed either inapplicable to low-resource settings, or not useful for early detection and triggering immediate treatment. Accordingly, the research team developed a set of criteria to be used to assess the relevance of each method. The criteria were:

- Appropriateness of early detection and triggering immediate treatment
- Feasibility of use in most secondary-level or higher hospitals
- Applicability to intraoperative AND/OR postoperative PPH detection

Three experts (SM, IG, FA) were asked to independently rate each detection method identified in the literature on all criteria. The whole group then met to discuss disagreements until consensus was reached. Detection methods were deemed irrelevant for the purposes of this report if all experts rated them as “No” for all criteria OR if the current cost of the detection method rendered it unaffordable in LMICs. The completed screening tool with decisions on each considered detection method is provided in Supplementary Table 3.

Detection methods deemed relevant for the purposes of this report were then described (descriptions and synthesis are included in the Evidence Synthesis). Detection methods as presented in the Evidence Synthesis were composed of both blood loss assessment methods (which utilized varying blood collection devices to assist with quantification of blood loss) and other methods of assessing PPH. Supplementary Figure 1 provides a useful schematic for how the research team categorized each of the detection methods.

Pre-screening of PPH Detection Methods

PPH detection method -- Description	Is this method appropriate for EARLY detection and triggering immediate treatment?	Is this method likely to be feasible to use in most secondary-level or higher hospitals?	Is this method applicable to intraoperative PPH detection?	Is this method applicable to postoperative PPH detection?	Comments
Visual estimation of blood loss<br>Obstetric or anaesthetic team visually estimates volume of blood loss. It mainly relies on a providers' opinion based on clinical experience and use of visual aids	No	Yes	Yes	Yes	Commonly used to trigger treatment. However, this method grossly underestimates blood loss and thus is not useful for early detection
Clinical signs of PPH<br>Signs of hypovolemia, hypotension (systolic below 80 mmHg), tachycardia (heart rate over 100 bmp), tachypnoea, cardiac output (decreased pulse pressure and respiratory rate) and altered mental state.	No	Yes	Yes	Yes	Commonly used to trigger treatment. However, except in anaemic women, there may be a substantial delay in the onset of clinical signs of PPH, undermining utility for early detection Also, blood pressure may be artificially high due to medications used, LOC affected by medications.
Visual charts and early warning scores<br>Visual charts usually involve scoring each parameter of vital signs monitoring (usually temperature, pulse, blood pressure, respiratory rate, and conscious level), with the aggregate score determining the need for closer monitoring, intervention, and review. Some charts are not validated for use with childbearing women.	Yes	Yes	Yes	Yes	A recent systematic review found that Obstetric Early Warning Systems are effective for early detection and triggering action, particularly when color-coded/shaded.

PPH detection method -- Description	Is this method appropriate for EARLY detection and triggering immediate treatment?	Is this method likely to be feasible to use in most secondary-level or higher hospitals?	Is this method applicable to intraoperative PPH detection?	Is this method applicable to postoperative PPH detection?	Comments
Changes in laboratory values such as haemoglobin and haematocrit Evaluation of haemoglobin concentration through blood sample.	No	Yes	No	Yes	Due to time lag in receiving results, this method is not useful intraoperatively, but could potentially be useful post-operatively if adequate and functioning laboratory facilities are available.
Artificial Intelligence (Triton) Image recognition algorithms are used with pictures of blood-soaked items to perform colorimetric analysis which quantifies haemoglobin and blood loss.	Yes	No	Yes	Yes	Not currently accessible for LMIC.
Dye Dilution Assesses blood flow through venous system by injecting a known quantity of dye into a vein and monitoring its plasmatic concentration after uterine bleeding stops.	No	No	No	No	Due to time lag in receiving results, this method is not useful intraoperatively, but could potentially be useful post-operatively.
Spectrophotometry This method is based on collected blood being mixed with a standardized solution which converts haemoglobin to acid haematin or cyanmethemoglobin. This in turn can be measured by a spectrophotometer or colorimeter.	No	No	No	No	Rare and not currently accessible for LMIC.

PPH detection method -- Description	Is this method appropriate for EARLY detection and triggering immediate treatment?	Is this method likely to be feasible to use in most secondary-level or higher hospitals?	Is this method applicable to intraoperative PPH detection?	Is this method applicable to postoperative PPH detection?	Comments
Initial Imaging<br>This technique involves US duplex Doppler pelvis, US pelvis transabdominal, US pelvis transvaginal imaging to detect uterine atony or lack of uterine contraction.	No	No	No	No	Unclear if available in LMICs.

Blood loss collection devices, blood loss assessment methods and other methods of PPH detection

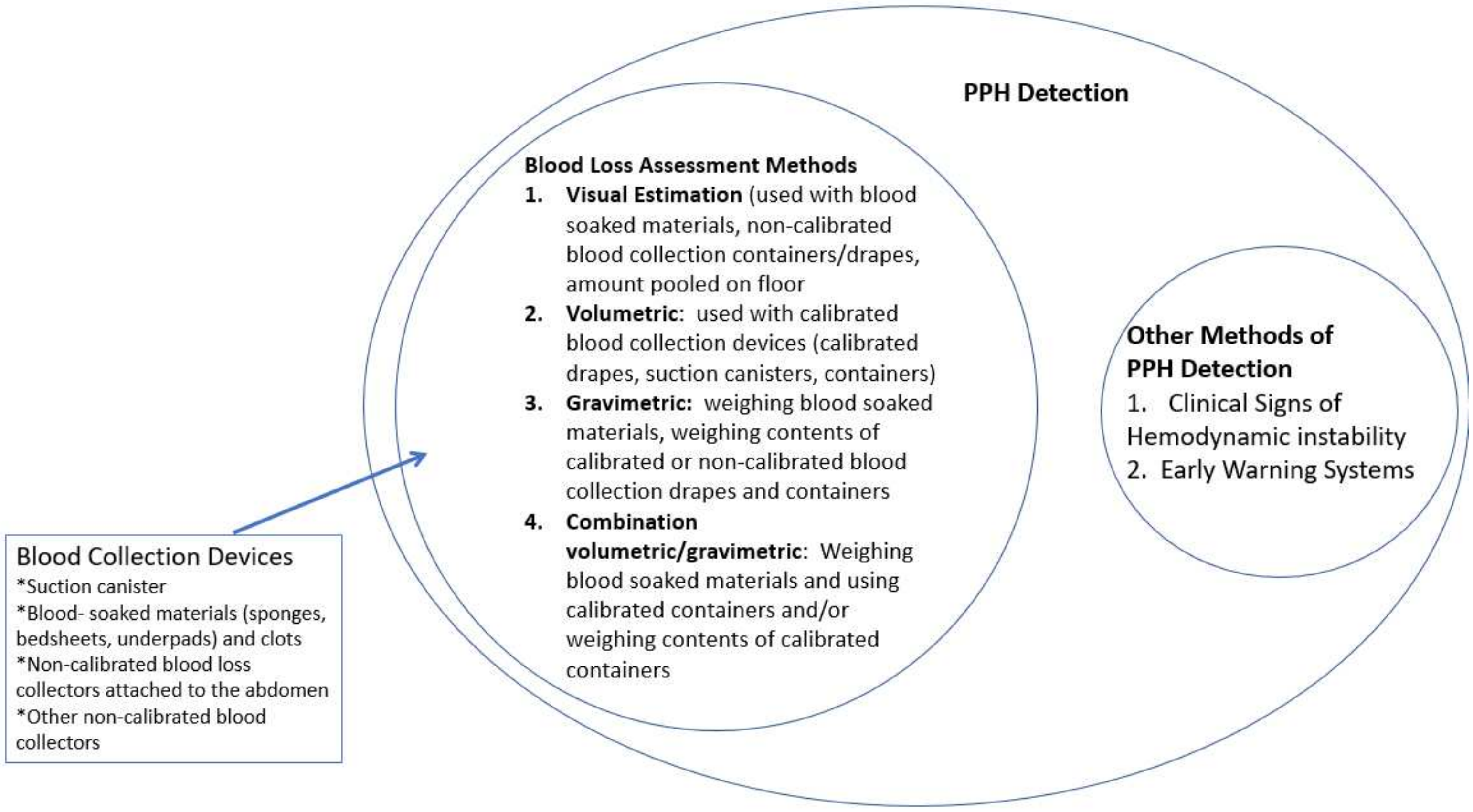

Supplementary File S3. In-person meeting agenda

WHO Technical Consultation on early detection and first response to PPH during and after caesarean birth

Meeting Agenda
27-28 September 2022
Av. Appia 20, Geneva, Salle U2
Zoom: https://who.zoom.us/j/91683929578
(Meeting ID: 916 8392 9578; Password: &uqmG2dD)

Introduction

Thank you for your participation in the **WHO Technical Consultation on early detection and first response to PPH during and after caesarean birth**. This consultation is part of the ongoing EMOTIVE project (Early detection of Postpartum Haemorrhage and treatment using the WHO MOTIVE 'first response' bundle), coordinated by the University of Birmingham.

As a part of the EMOTIVE project, WHO and some EMOTIVE team members are conducting a three-stage modified Delphi process to generate consensus on the optimal approach for early detection and first-response treatment for postpartum haemorrhage occurring during (intraoperative) and after (postoperative) caesarean birth. The first two rounds of this consultation were informed by an overview of reviews of the literature and conducted via an anonymous online platform. This third, in-person round will serve to conclude the consultation.

Experts' Meeting Objectives

This meeting has two primary objectives:

To agree on an optimal CS-PPH detection strategy that would include blood loss measurement methods and thresholds for action during and after caesarean birth.

To develop a first response approach to manage PPH during and after caesarean birth. This objective includes both the selection of the strategy components and the identification of the optimal approach (bundle, algorithm, checklist, or a combination of them)

Organization of the Meeting

This meeting will be conducted in-person and via Zoom over the course of two (2) days. Please use this link to access the meeting virtually: https://who.zoom.us/j/91683929578 (Meeting ID: 916 8392 9578; Password: &uqmG2dD)

The first day of the meeting, we will present and discuss the results of the overview of reviews and first two rounds of the Delphi study. You will have the opportunity to ask questions regarding the findings. Later in the day, we will pose specific questions to the panel regarding issues raised by the results to-date. You will be asked to provide your opinion on each of these topics. Discussions will be focused first on the intraoperative period, and then the postoperative period. The objective is to present different points of view on methods for early detection of CS-PPH, thresholds for triggering action, and first-response treatments by the close of day 1.

The second day of the meeting, we will present proposed strategies for implementing early detection and first-response for CS-PPH in the intraoperative and postoperative periods. You will be asked to provide your opinions on the proposed strategies. Finally, you will be asked to rate the proposed strategies. The final results—including whether consensus was reached—and conclusion will be presented before the end of day 2.

Dates and time

Date: 27-28 September 2022	
Day 1	*Day 2*
Africa<br>• *Kenya — 10:00-18:05*<br>• *Nigeria — 08:00-16:05*<br>• *Republic of Congo —08:00-16:05*<br>• *South Africa — 09:00-17:05*<br><br>Americas<br>• *Argentina — 04:00-12:05*<br>• *Uruguay — 04:00-12:05*<br><br>Europe<br>• *Denmark — 09:00-17:05*<br>• *France (Paris) — 09:00-17:05*<br>• *Switzerland — 09:00-17:05*<br>• *United Kingdom — 08:00-16:05*<br><br>South-East Asia<br>• *Australia (Melbourne) — 17:00-01:05*<br>• *Egypt — 09:00-17:05*<br>• *Vietnam — 14:00-22:05*<br><br>Western Pacific<br>• *India — 12:30-20:35*<br>• *Philippines —15:00-23:05*<br>• *Thailand — 14:00-22:05*	U. Africa<br>• *Kenya — 11:00-14:05*<br>• *Nigeria — 09:00-12:05*<br>• *Republic of Congo — 09:00-12:05*<br>• *South Africa — 10:00-13:05*<br><br>Americas<br>• *Argentina — 05:00-08:05*<br>• *Uruguay — 05:00-08:05*<br><br>Europe<br>• *Denmark — 09:00-12:05*<br>• *France — 10:00-13:05*<br>• *Switzerland — 10:00-13:05*<br>• *United Kingdom — 09:00-12:05*<br><br>South-East Asia<br>• *Australia — 18:00-21:05*<br>• *Egypt — 10:00-13:05*<br>• *Vietnam — 15:00-18:05*<br><br>Western Pacific<br>• *India — 13:30-16:35*<br>• *Philippines — 16:00-19:05*<br>• *Thailand — 15:00-18:05*

AGENDA

**DAY 1: 27 September 2022**		**Presenter**
9:00 – 9:30<br>*30 min*	*Opening session*<br>— Welcome and introductions<br>— Meeting objectives<br>— Meeting logistics	Dr. Olufemi Oladapo<br>Dr. Ioannis Gallos<br>Dr. Fernando Althabe<br>Ms. Caitlin Williams
9:30 – 10:30<br>*60 min*	*Session 1*<br>— Brief review of the objectives and methods of the Delphi study<br>— Briefly summarize the state of the literature<br>— Findings from the first and second rounds of the Delphi study<br>— Questions	Ms. Verónica Pingray<br>Ms. Caitlin Williams<br>Ms. Verónica Pingray<br>Prof. Suellen Miller
10:30 – 10:45<br>*15 min*	*Coffee/Tea Break*	
10:45 – 12:45<br>*120 min*	*Session 2*<br>— Briefly review summary of findings on **intraoperative** period<br>— Guided discussions by topic	Ms. Verónica Pingray<br>Prof. Suellen Miller
12:45 – 13:30<br>*45 min*	*Lunch*	
13:30 – 14:15<br>*45 min*	*Continue Session 2*	Prof. Suellen Miller
14:15 – 15:45<br>*90 min*	*Session 3*<br>— Briefly review summary of findings on **postoperative** period<br>— Guided discussions by topic (detection, thresholds, first-response treatments)	Ms. Verónica Pingray<br>Dr. Fernando Althabe
15:45 – 16:00<br>*15 min*	*Coffee/Tea Break*	
16:00 – 17:00<br>*60 min*	*Continue Session 3*	Dr. Fernando Althabe
17:00 – 17:05<br>*5 min*	*Closing DAY 1*	Dr. Ioannis Gallos
**DAY 2: 28 September 2022**		
10:00 – 10:15<br>*15 min*	*Welcome*<br>— Objective and procedures for Day 2	Dr. Fernando Althabe
10:15 – 11:00<br>*45 min*	*Session 1*<br>— Presentation of a revised list of early detection and first-response treatments for intraoperative and postoperative PPH<br>— Final voting for the intraoperative and postoperative interventions and presentation of results	Prof. Suellen Miller<br>Ms. Caitlin Williams
11:00 – 11:15<br>*15 min*	*Coffee/Tea Break*	
11:15 – 12:35<br>*80 min*	*Session 2*<br>— Organization of an implementation strategy<br>— Guided discussion of possible additional considerations	Dr. Fernando Althabe<br>Prof. Suellen Miller
12:35 – 13:00<br>*25 min*	*Session 3*<br>— Conclusion and next steps	Dr. Fernando Althabe
13:00 – 13:05<br>*5 min*	*Closing DAY 2*	Dr. Arri Coomarasamy<br>Dr. Olufemi Oladapo

Supplementary File S4. In-person meeting discussion question guide

Early detection and first response to postpartum haemorrhage during and after caesarean birth
A Modified-Delphi Study

Pending controversies or disagreements issues to discuss
27-28 September 2022

DAY 1: 27 September 2022

Definitions:
The experts agreed that the same definition of PPH should be used for both vaginal and caesarean births (same regardless of the mode of birth). Currently the WHO defines PPH as blood loss at least 500 mL within 24 hours after birth.

Draft proposed list of intraoperative interventions

Early detection of PPH and thresholds for triggering first-response management

- Aspirated blood volume (+ weighted pads, sponges, gauzes, etc. if feasible) at least 1000 mL, OR
- Haemodynamic instability (blood pressure, heart rate, oximetry) with any blood loss volume

# Register the final amount of intraoperative blood loss and hand over this information to recovery area.

First-response treatment

- TXA (1g in 10 mL IV over 10 min) for all women
- Examine and rapidly initiate cause-specific first response:
  - If trauma: Rapid haemostasis: hysterorraphy, tears, wound.
  - If atony/placental cause: uterotonics and intra-abdominal uterine massage or exteriorize the uterus and massage
- Uterotonics for all women
- IV fluids with crystalloids for all women

Pending issues

<u>**Thresholds**</u>

Background: The experts agreed that blood loss of at least 1000 mL should be used as a threshold to trigger first-response management.

	%	Ranking
**Intraoperative**		
≥1000 mL blood loss OR signs of hemodynamic instability, whichever comes first	72.7	1
Hemodynamic instability alone, regardless of volume of blood loss	40.9	2
≥1000 ml (blood loss alone, regardless of signs of hemodynamic instability)	50.0	3
≥500 mL blood loss OR signs of hemodynamic instability, whichever comes first	50.0	4
**Postoperative**		
≥1000 mL blood loss OR signs of hemodynamic instability, whichever comes first	59.1	1
Hemodynamic instability alone, regardless of volume of blood loss	36.4	2
≥1000 ml (blood loss alone, regardless of signs of hemodynamic instability)	36.7	3
≥500 mL blood loss OR signs of hemodynamic instability, whichever comes first	50.0	4

We believe is important to discuss the overall implications of the different thresholds on clinical practice, in the context of the total blood that women lose during and after CS. At the meeting we will present a summary of what is known so far from PPH prevention trials.

Question 1: Now that we have reviewed this additional data, do we need to revise the top-ranked threshold from the previous rounds (at least 1000 mL OR signs of haemodynamic instability, whichever comes first)?

Question 1.1: If the answer is not to revise, then is it acceptable that the definition (500 mL) and threshold (1000 mL) for triggering action differ?

Treatments

Background: The experts agreed to an aetiology-based treatment approach, but also agreed that the uterotonic of choice should be administered for all women, regardless of presence of atony.

Treatment Options	Median
Examine and rapidly initiate cause-specific first response (e.g., if trauma: rapid surgical haemostasis; if atony/placental cause: uterotonics and uterine massage)	9
Tranexamic acid for all women with PPH during CS regardless of aetiology	8
Plasma expansion with crystalloids for all women with PPH during CS regardless of aetiology	7.5
Uterotonics for all women with PPH during CS regardless of aetiology	7

Question 2: How do we make a recommendation that encompasses these seemingly contradictory statements (i.e., should the first-response uterotonic be administered to all women or just to women with atony?)?

Background: The experts agreed that a first response to intraoperative PPH would be to give oxytocin and TXA. However, many women having a caesarean birth would have already received/or be receiving IV oxytocin, and perhaps TXA, for PPH prevention.

Treatment Options	Median
Examine and rapidly initiate cause-specific first response (e.g., if trauma: rapid surgical haemostasis; if atony/placental cause: uterotonics and uterine massage)	9
Tranexamic acid for all women with PPH during CS regardless of aetiology	8
Plasma expansion with crystalloids for all women with PPH during CS regardless of aetiology	7.5
Uterotonics for all women with PPH during CS regardless of aetiology	7

Question 3: How do we adapt first-response recommendations if women are already receiving oxytocin infusion (common practice and consensus statement) for PPH prevention?

Question 4: In the event that women will routinely be receiving TXA for PPH prevention during CS, what recommendations should we give should they begin to haemorrhage intraoperatively at less than 30 min from the first dose?

Regarding IV fluids

Background: In the survey, the experts agreed that plasma expansion with crystalloids should be used for all women with PPH during CS, regardless of aetiology.

Treatment Options	Median
Examine and rapidly initiate cause-specific first response (e.g., if trauma: rapid surgical haemostasis; if atony/placental cause: uterotonics and uterine massage)	9
Tranexamic acid for all women with PPH during CS regardless of aetiology	8
Plasma expansion with crystalloids for all women with PPH during CS regardless of aetiology	7.5
Uterotonics for all women with PPH during CS regardless of aetiology	7

Question 5: Although we have used “Plasma expansion with crystalloids” in previous surveys, it may not be the best term. Should we use a different term (e.g., increase IV fluids with crystalloids for hemodynamic

maintenance)?<br><br>***Question 6:*** What should our recommendations be about how to continue IV fluids infusion with crystalloids for hemodynamic maintenance?<br><br>***Question 7:*** Do you think that we should be giving more details on type of crystalloids?<br><br>**Additional considerations**<br>***Question 8:*** Are there/should there be additional considerations for any other first-response treatments during the intraoperative period?
**Draft proposed list of postoperative interventions**

Early detection of PPH and thresholds for triggering first-response management

- Haemodynamic instability (blood pressure, heart rate, oxygen saturation) with any blood loss volume, OR
- Accumulated blood loss at least 1000 mL (weighted pads + blood loss during CS)

# If blood loss during CS <500 ml: usual postoperative clinical monitoring.

# If blood loss during CS 500-999 ml: further assessment, preparedness, and close monitoring

First response treatment

- TXA (1g in 10 mL IV over 10 min) for all women
- Examine and rapidly initiate cause-specific first response:
  - If trauma suspected: re-laparotomy for surgical haemostasis
  - If atony: Uterotonics
- Uterotonics for all women
- IV fluids with crystalloids for all women

Pending issues

Background: During the survey, we asked questions about the detection methods, thresholds, and treatments using 2 hours as the postoperative period.

Question 9: Is it valid to extend the results to the first 24 hours, or is a different strategy needed for hours 1-2 hours postoperative versus 3-24 hours postoperative?

For example: Should a woman with cumulative blood loss of 1100 mL in the first 2 hrs have her bleeding managed in the same way as a woman with a cumulative blood loss of 1100 mL in the first 24 hrs?

Detection & thresholds

Background: The panel agreed that using **clinical signs of haemodynamic instability** is a more appropriate option for detecting postoperative PPH due to the risk of internal bleeding postoperative.

Additionally, the panel agreed that deploying volumetric methods (e.g., drapes) would be less feasible than monitoring vital signs. Reasons given were a) the possible low acceptability by women (due to discomfort) and b) the lack of feasibility of continued measurement during women's transfer and normal movements.

Blood loss measurement and other PPH detection methods	How would you rate each of the methods below for early detection of PPH considering… usefulness?	feasibility?	acceptability?	resources required?
Clinical signs of haemodynamic instability	8.0	8.0	8.0	3.5
Volumetric/gravimetric + clinical signs of haemodynamic instability	7.0	7.0	NA	NA
Clinical judgement such as rate of flow and duration	5.0	6.0	7.0	2.0
Gravimetric	5.0	3.0	5.0	7.0
Volumetric	5.0	4.5	7.0	7.0
Visual estimation of blood loss	4.0	7.0	7.0	1.0
Visual charts and early warning scores (EWS)	7.0	7.0	6.5	6.0
Visual estimation + visual charts /EWS	7.0	7.0	NA	NA
Volumetric + gravimetric	7.0	5.0	NA	NA

Given the diverse realities of postoperative monitoring, priority should be given either to frequent monitoring of vital signs or the use of continuous monitoring devices, dependent on the availability of equipment and personnel.

Question 10: Consequently, what kind of guidance should we give, if any? How frequently should the woman's haemodynamic status be monitored? For how long? With what kind of devices?

Background: In addition to monitoring vital signs, providers should be **monitoring blood loss.** The experts agreed that in the postoperative period, providers should continue with the cumulative measurement of blood loss (i.e., adding intraoperative blood loss to postoperative blood loss to calculate total loss).

Pingray V, et al. BMJ Open 2024; 14:e079713. doi: 10.1136/bmjopen-2023-079713

	Median
The volume of intraoperative blood loss should be taken into consideration when determining whether the postoperative threshold had been reached	8

Question 11: What guidance should we give to reflect cumulative intra- and postoperative blood loss?

We propose, **as an example**, the following approach to blood loss monitoring **in situations in which the woman's vital signs are stable**. ***If vital signs indicate haemodynamic instability, the guidance should be to initiate treatment regardless of postoperative blood loss.***

Intraoperative		Postoperative		
**Blood Loss**	**Action Indicated**	**Type of Monitoring**	**Cumulative Blood Loss**	**Action Indicated**
<500	No treatment	Normal monitoring	Cumulative blood loss <1000	Do not initiate treatment. Continue normal monitoring
500-999	No treatment	Further assessment, preparedness, and more frequent postop monitoring	Cumulative blood loss <1000	Do not initiate treatment. Return to normal monitoring
500-999	No treatment	Further assessment, preparedness, and more frequent postop monitoring	Cumulative blood loss >1000	Initiate treatment, and monitor the woman's response to the treatment
≥1000	Initiate treatment and monitor woman's response	Monitor woman's response to treatments	-	-

DAY 2: 28 September 2022

Question 12: How should we frame the first-response strategies? Or bundle?

- Algorithm?
- Checklist?
- Or a hybrid?

Question 13: Should we include preparedness interventions at the facility level?
Prepare for CS PPH

Blood loss measurement

- Calibrated containers
- Pump/aspirator/vacuum suction
- Pads/swabs/lap cloths, etc. with known dry weight
- Scale to weigh the above

Medications

- TXA
- Uterotonics (Oxytocin, ergometrine, sulprostone)
- Crystalloids

Haemodynamic Monitoring

- CRADLE device/ cardiac monitor/ oximeter/sphygmomanometer?

Supplementary File S5. List of contributors

EXPERTS

Fadhlun ALWY AL-BEITY
Senior Lecturer - OBGYN
Tanzania

Nabhan ASHRAF
Professor of Obstetrics and Gynecology
Egypt

Brendan CARVALHO
Professor of Anesthesiology, Perioperative and Pain Medicine
USA

Maria Fernanda ESCOBAR VIDARTE
Professor of Obstetrics and Gynaecology
Colombia

Cherrie EVANS
Senior MNH Advisor
USA

Tippawan LIABSUETRAKUL
Professor of Obstetrics and Gynecology
Thailand

Elliott MAIN
Professor of Obstetrics and Gynecology
USA

Zahida QURESHI
OBGYN and associate professor of Obstetrics and Gynaecology
Kenya

John VARALLO
Global Director Safe Surgery
USA

Edgardo ABALOS
OBGYN and senior researcher
Argentina

Sabaratnam ARULKUMARAN
Emeritus Professor of Obstetrics and Gynaecology
UK

Catherine DENEUX-THARAUX
Perinatal Epidemiologist
France

Sue FAWCUS
Emeritus Professor of Obstetrics and Gynaecology and senior research scholar
South Africa

Hadiza GALADANCI
Professor of Obstetrics and Gynecology
Nigeria

Caroline HOMER
Professor and Co-Program Director Maternal, Child and Adolescent Health
Australia

Justus HOFMEYR
OBGYN and senior researcher
South Africa

Judith Maua ONG'AYI
Senior Technical Officer
Kenya

PHAN NGUYEN Quoc Thuan
OBGYN Clinician and clinical researcher
Vietnam

Alexander DUMONT
Senior Researcher
France

Pisake Lumbiganon
Dean and Professor of Obstetrics and Gynecology Convenor, Thai Cochrane Network Faculty of Medicine Khon Kaen University Khon Kaen
Thailand

OBSERVERS

Arri COOMARASAMY
OBGYN and Professor of Gynaecology
UK

Inês NUNES
OBGYN clinician
Assistant Professor of Obstetrics and Gynecology
Portugal

Andrew WEEKS
Professor of International Maternal Health
UK

STEERING GROUP

Fernando ALTHABE
WHO Consultant
Argentina

Suellen MILLER
Director, Safe Motherhood Program
USA

Veronica PINGRAY
Perinatal epidemiologist and methodologist
Argentina

Caitlin R. WILLIAMS
Researcher
USA

WHO REGIONAL ADVISORS

Claudio SOSA
Consultant
Woman and Reproduction Health Unit
Maternal Health at the Latin American Center of Perinatology,
Women and Reproductive Health,
(CLAP/WR), PAHO

WHO SECRETARIAT

Ioannis GALLOS
Medical Officer, SRH/MPH
Maternal and Perinatal Health
Department of Sexual and Reproductive Health and Research

Olufemi OLADAPO
Unit Head, SRH/MPH
Maternal and Perinatal Health
Department of Sexual and Reproductive Health and Research

Mariana WIDMER
Technical Officer, SRH/MPH
Maternal and Perinatal Health
Department of Sexual and Reproductive Health and Research

Supplementary File S6. Good Surgical Practices

Prevention and early diagnosis of PPH should be applied simultaneously with good surgical practices. One way adherence to these practices can be enhanced is by using Surgical Safety Checklists. WHO has such a Surgical Safety Checklist (SSC), which is generic for any surgical procedure (47). This Checklist was developed with many collaborators. Its purpose is to reduce mistakes, omissions, and adverse outcomes, as well as to improve communication and teamwork during surgery. One study demonstrated that serious morbidity and mortality were reduced from 1.5% to 0.8% by using the WHO SCC in a variety of global settings (48). Several of the expert panel members mentioned using the WHO SSC as an umbrella of good clinical practices for preventing or reducing risks of haemorrhage at CS.

One problem with the generic SSC for caesarean births is the complexity of the specifics of the caesarean surgery and the frequency of PPH at CS. To address this a few organizations have modified the WHO SSC for CS in lower resourced settings or developed surgical checklists specifically for CS (4950). A copy of the Sun *et al*. checklist can be found below (**Figure 1**)

Additional guidance for safety during and after CS can be found in the WHO MCPC (51).

Following good surgical practices are implied as necessary steps for all teams involved in performing CS. There are commonalities among all checklists, but those specifically developed/modified for CS include information about the placenta, risk factors for maternal haemorrhage, including anaemia, and if there are risks present to prepare by having 2 large bore IVs, keeping uterotonics at hand, giving antibiotics before skin incision, foley for urine drainage, anti-microbial skin wash, estimation of blood loss after the surgery and, if PPH occurred, an order to be written for nursing to check Hct, continue IVs, record urine output, and to alert physician if Hct < 20, BP < 80/50, and pulse > 100, and or urine output < 30 cc hr. These more specific parameters and procedures for intra and postop CS management can be useful along with the Hybrid Algorithm-Bundle Strategy we are suggesting.

Figure 1: Sun et al. Caesarean Birth Checklist

NURSE: Name ______________________ Date/Time ______________________

Prior to starting surgery

- ☐ Verify patient name ______________________
- ☐ Allergies ______________
- ☐ Indication ______________________________
- ☐ Fetal presentation: Cephalic / Breech / Transverse – back down / Transverse – back up
- ☐ Placentation: Anterior / Posterior / Lateral / Fundal / Previa
- ☐ High risk for maternal hemorrhage? (multiple prior cesareans, chorioamnionitis, polyhydramnios, twin gestation, magnesium sulfate, prolonged Pitocin infusion)
    - ☐ Yes
        - ☐ 2 large bore IVs in patient with 500cc infusing now
        - ☐ Cytotec in the Salle d'Op
        - ☐ Ergometrine in the Salle d'Op
        - ☐ Oxytocin in the Salle d'Op
    - ☐ No
- ☐ Antibiotics given before skin incision
- ☐ Bladder foley placed
- ☐ Safety belt on patient's leg, above knees
- ☐ Left lateral displacement
- ☐ Skin preparation
    - o Wash
    - o Betadine

DOCTOR: Name______________________ Date/Time ______________________

After completion of surgery

- ☐ Instrument and sponge counts correct? Yes / No
- ☐ Estimation of blood loss______________
    - ☐ Normal blood loss
    - ☐ Large blood loss
        - ☐ Write order for nurse to do each of the following:
            - ☐ Check Hct immediately and 4 hours post-operatively
            - ☐ Give 1000cc Normal Saline Bolus
            - ☐ Record urine output every hour
            - ☐ Call doctor if Hct $\leq$20, BP<80/50 and pulse >100, urine output <30cc/hour
- ☐ Complications
    - ☐ None
    - ☐ Blood transfusion intra-operatively
    - ☐ Maternal death
    - ☐ Perinatal death
    - ☐ Hysterectomy
    - ☐ Uterine artery laceration
    - ☐ Internal organ trauma (bladder, bowel, etc)
    - ☐ Vertical extension of uterine incision

References

1. World Health Organization. WHO recommendations for the prevention and treatment of postpartum haemorrhage. Geneva: World Health Organization; 2012. 41- p.
2. Mavrides E, Allard S, Chandraharan E, Collins P, Green L, Hunt BJ, et al. Prevention and management of postpartum haemorrhage. BJOG: An International Journal of Obstetrics & Gynaecology. 2016;124:e106-e49.
3. Sentilhes L, Vayssière C, Deneux-Tharaux C, Aya AG, Bayoumeu F, Bonnet MP, et al. Postpartum hemorrhage: Guidelines for clinical practice from the French College of Gynaecologists and Obstetricians (CNGOF): In collaboration with the French Society of Anesthesiology and Intensive Care (SFAR). European Journal of Obstetrics and Gynecology and Reproductive Biology. 2016;198(2016):12-21.
4. Institute of O, Gynaecologists Royal College of Physicians of I, Directorate of S, Clinical Programmes Health Service E. Prevention and management of primary postpartum haemorrhage. 2014. p. 12-26.
5. RANZCOG. Management of Postpartum Haemorrhage (PPH). 2017. p. 1-14.
6. Muñoz M, Stensballe J, Ducloy-Bouthors A-S, Bonnet M-P, De Robertis E, Fornet I, et al. Patient blood management in obstetrics: prevention and treatment of postpartum haemorrhage. A NATA consensus statement. Blood transfusion = Trasfusione del sangue. 2019;17(2):112-36.
7. Schlembach D, Helmer H, Henrich W, von Heymann C, Kainer F, Korte W, et al. Peripartum haemorrhage, diagnosis and therapy. Guideline of the DGGG, OEGGG and SGGG (S2k Level, AWMF Registry No. 015/063, March 2016). Geburtshilfe und Frauenheilkunde. 2018;78(04):382-99.
8. Minakami H, Maeda T, Fujii T, Hamada H, Iitsuka Y, Itakura A, et al. Guidelines for obstetrical practice in Japan: Japan Society of Obstetrics and Gynecology (JSOG) and Japan Association of Obstetricians and Gynecologists (JAOG) 2014 edition. Journal of Obstetrics and Gynaecology Research. 2014;40(6):1469-99.
9. World Health Organization. WHO Recommendation on Tranexamic Acid for the Treatment of Postpartum Haemorrhage. Geneva: World Health Organization; 2017. 41- p.
10. Lalonde A, Motherhood FS, Newbon Health C. Prevention and treatment of postpartum hemorrhage in low-resource settings. International Journal of Gynecology & Obstetrics. 2012;117(2):108-18.
11. ACOG. Postpartum Hemorrhage. Obstetrics & Gynecology. 2017;183:e168-e86.
12. Queensland Clinical Guidelines. Maternity and neonatal clinical guideline: Primary postpartum haemorrhage. Queensland Health. 2018:1-39.
13. CMQCC, Health CDoP. Improving Health Care Response to Obstetric Hemorrhage Version 2.0: A California Quality Improvement Toolkit2015.
14. Leduc D, Senikas V, Lalonde AB. No. 235-Active Management of the Third Stage of Labour: Prevention and Treatment of Postpartum Hemorrhage. Journal of Obstetrics and Gynaecology Canada. 2018;40(12):e841-e55.
15. NICE. Caesarean birth 2021 [cited 2023 Apr 30]. Available from: https://www.nice.org.uk/guidance/ng192.
16. World Health Organization. Managing complications in pregnancy and childbirth: A guide for midwives and doctors. 2nd ed. Geneva: World Health Organization; 2017. 492- p.
17. Smith CM, Borders AE, King TL. Quantitative Blood Loss in Obstetric Hemorrhage: ACOG COMMITTEE OPINION SUMMARY, Number 794. Obstetrics and gynecology. 2019;134(6):1368-9.
18. World Health O. WHO Recommendation on Tranexamic Acid for the Treatment of Postpartum Haemorrhage. WHO Recommendation on Tranexamic Acid for the Treatment of Postpartum Haemorrhage. 2017:41-.
19. Gallos ID, Papadopoulou A, Man R, Athanasopoulos N, Tobias A, Price MJ, et al. Uterotonic agents for preventing postpartum haemorrhage: A network meta-analysis. Cochrane Database of Systematic Reviews. 2018;2018(12).
20. Kellie FJ, Wandabwa JN, Mousa HA, Weeks AD. Mechanical and surgical interventions for treating primary postpartum haemorrhage. Cochrane Database of Systematic Reviews. 2020.

21. Parry Smith WR, Papadopoulou A, Thomas E, Tobias A, Price MJ, Meher S, et al. Uterotonic agents for first-line treatment of postpartum haemorrhage: A network meta-analysis. Cochrane Database of Systematic Reviews. 2020;2020(11).
22. Hancock A, Weeks AD, Lavender DT. Is accurate and reliable blood loss estimation the 'crucial step' in early detection of postpartum haemorrhage: an integrative review of the literature. BMC Pregnancy and Childbirth 2015 15:1. 2015;15(1):1-9.
23. Andrikopoulou M, D'Alton ME. Postpartum hemorrhage: Early identification challenges. Seminars in Perinatology. 2019;43(1):11-7.
24. Diaz V, Abalos E, Carroli G. Methods for blood loss estimation after vaginal birth. Cochrane Database of Systematic Reviews. 2018;2018(9).
25. Lertbunnaphong T, Lapthanapat N, Leetheeragul J, Hakularb P, Ownon A. Postpartum blood loss: visual estimation versus objective quantification with a novel birthing drape. Singapore Medical Journal. 2016;57(06):325-8.
26. Patel A, Goudar SS, Geller SE, Kodkany BS, Edlavitch SA, Wagh K, et al. Drape estimation vs. visual assessment for estimating postpartum hemorrhage. International Journal of Gynecology & Obstetrics. 2006;93(3):220-4.
27. Zhang WH, Deneux-Tharaux C, Brocklehurst P, Juszczak E, Joslin M, Alexander S. Effect of a collector bag for measurement of postpartum blood loss after vaginal delivery: cluster randomised trial in 13 European countries. BMJ. 2010;340(feb01 1):c293-c.
28. Ambardekar S, Shochet T, Bracken H, Coyaji K, Winikoff B. Calibrated delivery drape versus indirect gravimetric technique for the measurement of blood loss after delivery: a randomized trial. BMC Pregnancy and Childbirth 2014 14:1. 2014;14(1):1-6.
29. Atukunda EC, Mugyenyi GR, Obua C, Atuhumuza EB, Musinguzi N, Tornes YF, et al. Measuring post-partum haemorrhage in low-resource settings: The diagnostic validity of weighed blood loss versus quantitative changes in hemoglobin. PLOS ONE. 2016;11(4):e0152408-e.
30. Ushida T, Kotani T, Imai K, Nakano-Kobayashi T, Nakamura N, Moriyama Y, et al. Shock index and postpartum hemorrhage in vaginal deliveries: A multicenter retrospective study. Shock. 2021;55(3):332-7.
31. Borovac-Pinheiro A, Ribeiro FM, Morais SS, Pacagnella RC. Shock index and heart rate standard reference values in the immediate postpartum period: A cohort study. PLOS ONE. 2019;14(6):e0217907-e.
32. El Ayadi AM, Nathan HL, Seed PT, Butrick EA, Hezelgrave NL, Shennan AH, et al. Vital sign prediction of adverse maternal outcomes in women with hypovolemic shock: The role of shock index. PLOS ONE. 2016;11(2):e0148729-e.
33. Pacagnella RC, Borovac-Pinheiro A, Silveira C, Siani Morais S, Argenton JLP, Souza JP, et al. The golden hour for postpartum hemorrhage: Results from a prospective cohort study. International Journal of Gynecology & Obstetrics. 2021:ijgo.13823-ijgo.
34. Tanacan A, Fadiloglu E, Unal C, Beksac MS. Importance of shock index in the evaluation of postpartum hemorrhage cases that necessitate blood transfusion. Women & Health. 2020;60(9):1070-8.
35. Lee SY, Kim HY, Cho GJ, Hong SC, Oh MJ, Kim HJ. Use of the shock index to predict maternal outcomes in women referred for postpartum hemorrhage. International Journal of Gynecology & Obstetrics. 2018:ijgo.12714-ijgo.
36. Vousden N, Lawley E, Nathan HL, Seed PT, Gidiri MF, Goudar S, et al. Effect of a novel vital sign device on maternal mortality and morbidity in low-resource settings: a pragmatic, stepped-wedge, cluster-randomised controlled trial. The Lancet Global Health. 2019;7(3):e347-e56.
37. Umar A, Ameh CA, Muriithi F, Mathai M. Early warning systems in obstetrics: A systematic literature review. PLOS ONE. 2019;14(5):e0217864-e.
38. World Health Organization. WHO recommendations: Uterotonics for the prevention of postpartum haemorrhage. Geneva: World Health Organization; 2018. p. 53-.
39. World Health Organization. WHO recommendation on uterine balloon tamponade for the treatment of postpartum haemorrhage. Geneva: World Health Organization; 2021.
40. Shakur H, Beaumont D, Pavord S, Gayet-Ageron A, Ker K, Mousa HA. Antifibrinolytic drugs for treating primary postpartum haemorrhage. Cochrane Database of Systematic Reviews. 2018;2018(2).

Pingray V, *et al. BMJ Open* 2024; 14:e079713. doi: 10.1136/bmjopen-2023-079713

41. Kavak SB, Atilgan R, Demirel İ, Celik E, İlhan R, Sapmaz E. Endouterine hemostatic square suture vs. Bakri balloon tamponade for intractable hemorrhage due to complete placenta previa. Journal of Perinatal Medicine. 2013;41(6).
42. Khalil MI, Al-Dohami H, Aldahish MM. A method to improve the effectiveness of the Bakri balloon for management of postpartum hemorrhage at cesarean. International Journal of Gynecology & Obstetrics. 2011;115(2):198-200.
43. Pileggi-Castro C, Nogueira-Pileggi V, Tunçalp Ö, Oladapo OT, Vogel JP, Souza JP. Non-pneumatic anti-shock garment for improving maternal survival following severe postpartum haemorrhage: a systematic review. Reprod Health. 2015;12:28.
44. Brouwers MC, Kho ME, Browman GP, Burgers JS, Cluzeau F, Feder G, et al. AGREE II: Advancing guideline development, reporting and evaluation in health care. Can Med Assoc J [Internet]. 2010 Dec 14;182(18):E839–42. Available from: http://www.cmaj.ca/cgi/doi/10.1503/cmaj.090449
45. Shea BJ, Reeves BC, Wells G, Thuku M, Hamel C, Moran J, et al. AMSTAR 2: A critical appraisal tool for systematic reviews that include randomised or non-randomised studies of healthcare interventions, or both. BMJ [Internet]. 2017 Sep 21;j4008. Available from: https://www.bmj.com/lookup/doi/10.1136/bmj.j4008
46. Mousa HA, Blum J, Abou El Senoun G, Shakur H, Alfirevic Z. Treatment for primary postpartum haemorrhage. Cochrane Database Syst Rev [Internet]. 2014 Feb 12; Available from: https://doi.wiley.com/10.1002/14651858.CD003249.pub3
47. Tool and resources [Internet]. [cited 2024 Jan 10]. Available from: https://www.who.int/teams/integrated-health-services/patient-safety/research/safe-surgery/tool-and-resources
48. Haynes AB, Weiser TG, Berry WR, Lipsitz SR, Breizat A-HS, Dellinger EP, et al. A surgical safety checklist to reduce morbidity and mortality in a global population. N Engl J Med. 2009 Jan 29;360(5):491–9.
49. Sun M, Patauli D, Bernstein PS, Goffman D, Nathan LM. Use of a cesarean delivery checklist in an African maternity ward to improve management and reduce length of hospital stay. Int J Gynaecol Obstet. 2021 Feb;152(2):236–41.
50. Combs CA, Einerson BD, Toner LE, Patient Safety and Quality Committee, Society for Maternal-Fetal Medicine. Electronic address: smfm@smfm.org. Society for Maternal-Fetal Medicine Special Statement: Surgical safety checklists for cesarean delivery. Am J Obstet Gynecol. 2021 Nov;225(5):B43–9.
51. Managing complications in pregnancy and childbirth: A guide for midwives and doctors - Second Edition [Internet]. World Health Organization; 2017 [cited 2024 Jan 10]. Available from: https://www.who.int/publications/i/item/9789241565493
