# Supplementary material for: Strategies for optimising early detection and obstetric first response management of postpartum haemorrhage at caesarean birth: a modified Delphi-based international expert consensus
Source: BMJ Open. 2024 May 8;14(5):e079713. doi: 10.1136/bmjopen-2023-079713 (PMC11086283; doi:10.1136/bmjopen-2023-079713)
Supplement: Author's manuscript [file bmjopen-2023-079713.draft_revisions.pdf]

BMJ Open

BMJ Open is committed to open peer review. As part of this commitment we make the peer review history of every article we publish publicly available.

When an article is published we post the peer reviewers’ comments and the authors’ responses online. We also post the versions of the paper that were used during peer review. These are the versions that the peer review comments apply to.

The versions of the paper that follow are the versions that were submitted during the peer review process. They are not the versions of record or the final published versions. They should not be cited or distributed as the published version of this manuscript.

BMJ Open is an open access journal and the full, final, typeset and author-corrected version of record of the manuscript is available on our site with no access controls, subscription charges or pay-per-view fees (http://bmjopen.bmj.com).

If you have any questions on BMJ Open’s open peer review process please email info.bmjopen@bmj.com

BMJ Open

Strategies for optimising early detection and first response management of postpartum haemorrhage at caesarean birth: A modified Delphi-based international expert consensus

Journal:	*BMJ Open*
Manuscript ID	bmjopen-2023-079713
Article Type:	Original research
Date Submitted by the Author:	11-Sep-2023
Complete List of Authors:	Pingray, Verónica; Institute for Clinical Effectiveness and Health Policy,<br>Williams, Caitlin R.; University of North Carolina at Chapel Hill Gillings School of Global Public Health, Department of Maternal & Child Health; Institute for Clinical Effectiveness and Health Policy,<br>Al-beity, Fadhlun M; Muhimbili University of Health and Allied Sciences, Department of Obstetrics & Gynaecology ; Karolinska Institute, Department of Obstetrics & Gynaecology<br>Abalos, Edgardo; Secretaría de Salud Pública de Rosario, Maternidad Martin<br>Arulkumaran , Sabaratnam; Department of Obstetrics & Gynaecology, St George's Hospital,<br>Blumenfeld, Alejandro; Institute for Clinical Effectiveness and Health Policy; Universidad de Buenos Aires, Department of Public Health, Faculty of Medicine<br>Carvalho, B; Stanford University School of Medicine,<br>Deneux-Tharaux, Catherine; INSERM<br>Downe, Soo; University of Central Lancashire, Research in childbirth and health; University of Central Lancashire, THRIVE Centre, School of Heath and Community Studies<br>Dumont, Alexandre; Université Paris Cité, CEPED; INSERM<br>Escobar, Maria Fernanda; Fundación Valle del Lili, Departamento de Ginecología y Obstetricia; Universidad Icesi, Facultad de Ciencias de la Salud<br>Evans, Cherrie; Jhpiego/USA, Technical Leadership & Innovations Office<br>Fawcus, Sue ; University of Cape Town, Department of Obstetrics and Gynaecology<br>Galadanci, HS; Aminu Kano Teaching Hospital, Department of Obstetrics and Gynaecology; Bayero University Kano, Africa Center of Excellence for Population Health and Policy<br>Hoang, Diem-Tuyet ; Hung Vuong Hospital, Obstetrics and Gynecology<br>Hofmeyr, G Justus ; University of Botswana, Department of Obstetrics and Gynaecology; University of the Witwatersrand, Effective Care Research Unit,<br>Homer, Caroline ; Burnet Institute<br>Lewis, Ayodele; Amherst College<br>Liabsuetrakul, Tippawan; Prince of Songkla University, Department of Epidemiology; Prince of Songkla University, Department of Obstetrics & Gynecology

	Lumbiganon, Pisake; Khon Kaen University, Department of Obstetrics and Gynaecology<br>Main, Elliot; Stanford University, Department of Obstetrics & Gynecology - Maternal Fetal Medicine; California Maternal Quality Care Collaborative<br>Maua, Judith; Liverpool School of Tropical Medicine<br>Muriithi, Francis; University of Birmingham, Institute of Metabolism and Systems Research; Nottingham University Hospitals NHS Trust, Department of Obstetrics and Gynaecology<br>Nabhan, Ashraf; Ain Shams University Faculty of Medicine, Department of Obstetrics & Gynecology<br>NUNES, Inês; Centro Hospitalar de Vila Nova de Gaia Espinho EPE, Department of Obstetrics and Gynaecology; University of Porto, CINTESIS - Center for Health Technology and Services Research<br>Ortega, Vanesa; Institute for Clinical Effectiveness and Health Policy, Department of Mother and Child Health Research<br>Phan, Thuan; University of Liverpool, Department of Women's and Children's Health; Tu Du Hospital, Department of Delivery<br>Qureshi , Zahida ; University of Nairobi Department of Obstetrics and Gynecology,<br>Sosa, Claudio; Pan American Health Organization, Woman and Reproduction Health Unit at Maternal Health at the Latin American Center of Perinatology (CLAP/WR); Universidad de la República Uruguay, Department of Obstetrics and Gynecology. School of Medicine<br>Varallo, John; Global Surgery Foundation, Women's Health<br>Weeks, Andrew; University of Liverpool, Department of Women's and Children's Health; Liverpool Women's Hospital<br>Widmer, Mariana; WHO, UNDP-UNFPA-UNICEF-WHO-World Bank Special Programme of Research, Development and Research Training in Human Reproduction (HRP), Department of Sexual and Reproductive Health and Research<br>Oladapo, Olufemi ; World Health Organization, UNDP-UNFPA-UNICEF-WHO-World Bank Special Programme of Research, Development and Research Training in Human Reproduction (HRP), Department of Sexual and Reproductive Health and Research<br>Gallos, Ioannis; World Health Organization, UNDP-UNFPA-UNICEF-WHO-World Bank Special Programme of Research, Development and Research Training in Human Reproduction (HRP), Department of Sexual and Reproductive Health and Research<br>Coomarasamy, Arri; University of Birmingham<br>Miller, Suellen; University of California San Francisco, Bixby Center for Global reproductive Health<br>Althabe, Fernando ; World Health Organization, UNDP-UNFPA-UNICEF-WHO-World Bank Special Programme of Research, Development and Research Training in Human Reproduction (HRP), Department of Sexual and Reproductive Health and Research
Keywords:	OBSTETRICS, Maternal medicine < OBSTETRICS, Postpartum Period, Systematic Review, Clinical Decision-Making

SCHOLARONE™
Manuscripts

I, the Submitting Author has the right to grant and does grant on behalf of all authors of the Work (as defined in the below author licence), an exclusive licence and/or a non-exclusive licence for contributions from authors who are: i) UK Crown employees; ii) where BMJ has agreed a CC-BY licence shall apply, and/or iii) in accordance with the terms applicable for US Federal Government officers or employees acting as part of their official duties; on a worldwide, perpetual, irrevocable, royalty-free basis to BMJ Publishing Group Ltd ("BMJ") its licensees and where the relevant Journal is co-owned by BMJ to the co-owners of the Journal, to publish the Work in this journal and any other BMJ products and to exploit all rights, as set out in our licence.

The Submitting Author accepts and understands that any supply made under these terms is made by BMJ to the Submitting Author unless you are acting as an employee on behalf of your employer or a postgraduate student of an affiliated institution which is paying any applicable article publishing charge ("APC") for Open Access articles. Where the Submitting Author wishes to make the Work available on an Open Access basis (and intends to pay the relevant APC), the terms of reuse of such Open Access shall be governed by a Creative Commons licence – details of these licences and which Creative Commons licence will apply to this Work are set out in our licence referred to above.

Other than as permitted in any relevant BMJ Author's Self Archiving Policies, I confirm this Work has not been accepted for publication elsewhere, is not being considered for publication elsewhere and does not duplicate material already published. I confirm all authors consent to publication of this Work and authorise the granting of this licence.

For peer review only

Strategies for optimising early detection and first response management of postpartum haemorrhage at caesarean birth: A modified Delphi-based international expert consensus

Pingray V[†*], Williams CR†, Alwy Al-beity F, Abalos E, Arulkumaran S, Blumenfeld A, Carvalho B, Deneux-Tharaux C, Downe S, Dumont A, Escobar Vidarte MF, Evans C, Fawcus S, Galadanci H, Hoang T, Hofmeyr GJ, Homer C, Lewis A, Liabsuetrakul T, Lumbiganon P, Main E, Maua J, Muriithi F, Nabhan A, Nunes I, Ortega V, Phan T, Qureshi Z, Sosa C, Varallo J, Weeks A, Widmer M, Oladapo OT, Gallos I, Coomarasamy A, Miller S[‡], Althabe F[‡].

[†]These authors contributed equally. [‡]SM and FA share senior co-authorship.

[*]Corresponding author: Veronica Pingray, Institute for Clinical Effectiveness and Health Policy Email: vpingray@iecs.org.ar.

For peer review only - http://bmjopen.bmj.com/site/about/guidelines.xhtml

ABSTRACT

OBJECTIVE: There are no globally agreed upon strategies on early detection and first response management of postpartum haemorrhage during and after caesarean birth. Our study aimed to develop an international expert's consensus on evidence-based approaches for early detection and first response management of PPH intraoperatively and postoperatively in caesarean birth.

DESIGN: Systematic review and three-stage modified-Delphi expert consensus.

SETTING: International.

POPULATION: Panel of 22 global experts in postpartum haemorraghe with diverse backgrounds, and gender, professional, and geographic balance.

OUTCOME MEASURES: Agreement or disagreement on strategies for early detection and first response management of postpartum haemorrhage at caesarean birth.

RESULTS: Experts agreed that the same PPH definition should apply to both vaginal and caesarean birth. For the intraoperative phase, the experts agreed that early detection should be accomplished via quantitative blood loss measurement, complemented by monitoring the woman's haemodynamic status; and that first response should be triggered once the woman loses at least 500 mL of blood with continued bleeding or when she exhibits clinical signs of haemodynamic instability, whichever occurs first. For the first response, experts agreed on immediate administration of uterotonics and tranexamic acid, examination to determine aetiology, and rapid initiation of cause-specific responses. In the postoperative phase, the experts agreed that caesarean birth-related PPH should be detected primarily via frequently monitoring the woman's haemodynamic status and clinical signs and symptoms of internal bleeding, supplemented by cumulative blood loss assessment performed quantitatively or by visual estimation. Postoperative first response was determined to require an individualised approach.

CONCLUSION: These agreed-upon proposed approaches could help improve the detection of PPH in the intra and postoperative phases of caesarean birth and the first response management of intraoperative PPH. Determining how best to implement these strategies is a critical next step.

For peer review only - http://bmjopen.bmj.com/site/about/guidelines.xhtml

KEY WORDS

Postpartum haemorrhage; caesarean birth; early detection; first response management; expert consensus

For peer review only

For peer review only - http://bmjopen.bmj.com/site/about/guidelines.xhtml

STRENGTHS AND LIMITATIONS OF THIS STUDY

Strengths

- Use of a rigorous and systematic process to identify and synthesise high-quality PPH evidence in the literature.
- The selection of the expert panellists ensured a wide range of perspectives to enhance the utility and applicability of this consensus to a wide range of clinical settings.
- There was a very low rate of loss to follow-up and the first two rounds of the modified Delphi process were blinded to avoid social acceptability bias, and the hybrid meeting was facilitated to ensure that all panellists had equal opportunity to contribute to the discussion.

Limitations

- Due to the dearth of quality evidence on PPH related to caesarean birth, experts often had to extrapolate from evidence on interventions recommended for PPH in vaginal birth or make decisions based on their experiences. This sometimes led to omitting interventions that might be useful for early detection or first-response management.
- Given the highly technical content, we did not include recipients of these interventions, or their representatives, among the panellists.
- Since our systematic review, three updated PPH guidelines have been published, with some guidance relevant to PPH during or after caesarean birth. They mostly align with previously published guidance included in our study, with the exception of an increased focus on concealed haemorrhage assessment and one guideline recommending the use of prophylactic tranexamic acid for women at high PPH risk.

For peer review only - http://bmjopen.bmj.com/site/about/guidelines.xhtml

INTRODUCTION

Deaths from postpartum haemorrhage (PPH), the leading direct cause of maternal mortality globally, are potentially preventable with timely diagnosis and management (1,2). The risk of PPH is significantly higher with caesarean birth than vaginal birth, especially in cases of emergency caesarean birth (3). With global caesarean birth rates increasing, PPH during and after caesarean birth is a growing concern (4). The impact is particularly acute in low and middle-income countries (LMICs), where 32% of all maternal deaths after caesarean birth are related to PPH (5). In some LMICs, caesarean births outnumber vaginal births (6). Several factors challenge effective response to PPH in LMICs. These countries have well-documented difficulties accessing surgical services, skilled staff, and blood/blood products (7). Even when access concerns are addressed, use of interventions to detect and manage PPH are often inconsistent (8,9).

A standardised approach to PPH management has been shown to improve outcomes, including significantly reducing severe PPH rates amongst women giving birth vaginally (10). Similarly, studies including women having caesarean birth suggest a reduction in severe morbidity associated with the use of comprehensive haemorrhage protocols (11,12). The World Health Organization (WHO) has published and updated recommendations for the prevention and treatment of PPH (2,13,14). However, these recommendations neither detail methods for early detection of PPH during and after caesarean birth nor clearly indicate when to initiate treatment (i.e., the 'trigger' criteria), both of which may contribute to observed variations in clinical practice (2,7,15). PPH management practices may vary depending on whether the haemorrhage occurs during or after the surgical procedure (16). Proposing standardised and evidence-based global strategies may help to reduce practice variations and improve the quality of care. Our study aimed to develop an international consensus on standardised approaches for PPH detection and first response management during and after caesarean birth.

METHODS

The study involved a systematic review and an expert consensus using a three-stage modified-Delphi process.

Systematic review

A systematic review of published national and international guidelines for PPH prevention and management was conducted to identify interventions for collecting and measuring blood loss, methods for detecting PPH, thresholds for treatment, and first response interventions to manage PPH both during

For peer review only - http://bmjopen.bmj.com/site/about/guidelines.xhtml

surgery (intraoperative) and after surgery (postoperative). To be included, the guidelines needed to include guidance on the detection or management of PPH during or after caesarean birth. The literature search in PubMed, EMBASE, CINAHL, and Cochrane Library databases included papers published from January 2012 to July 2022 (Supplementary File S1). The search was complemented by reviewing the English-language grey literature to identify guidelines.

Since few of these guidelines were focused specifically on the intra- or postoperative phases or described PPH detection methods, an additional systematic search was conducted, focused on PPH detection and management during and after caesarean birth. Peer-reviewed systematic reviews of RCTs were eligible. Subject matter experts were consulted to add any relevant peer-reviewed articles missed by the systematic search.

Titles and abstracts of both guidelines and systematic reviews of RCTs were screened by pairs of independent reviewers who subsequently reviewed full texts, conducted quality appraisals, and extracted data using previously piloted forms. Only guidelines with AGREE II scores between 5 and 7 and systematic reviews with modified-AMSTAR quality assessment of "Moderate" or "High" were eligible for data extraction (17,18). The results of the systematic review were used to inform the development of the Delphi surveys and to provide the experts with summaries of the existing evidence.

Expert consensus

A three-stage modified Delphi process was conducted between December 2021 and September 2022, with two rounds of individual online surveys, followed by a third round: a hybrid (virtual and in-person) meeting with group discussions and final voting. Twenty-five PPH experts with the knowledge and skills to critically assess scientific evidence were invited to participate in all three rounds. They included specialists in nursing, midwifery, obstetrics, surgery, and anaesthesia. The experts were selected to ensure gender, professional, and geographic balance. Most experts were co-authors of recent national and international guidelines or principal or co-investigators of PPH clinical trials. The same experts were invited to participate in all three rounds. In the third round, observers representing professional associations and WHO regional offices, or who were leaders in PPH research were invited to share their views, but were not eligible to vote.

Based on the findings of the systematic review, questionnaires with open- and close-ended questions were developed, piloted, and administered using Survey Monkey™. A summary of the themes and interventions included in the surveys and criteria used to guide judgements are described in Box 1. The

For peer review only - http://bmjopen.bmj.com/site/about/guidelines.xhtml

criteria, methods, interventions, and other items included in the surveys were presented with definitions to facilitate interpretation. The themes were explored separately for the intraoperative and postoperative phases. Experts were asked to consider the postoperative PPH phase as only the first two hours immediately after the operation. Each online survey was available for response for six weeks, and three reminders were sent to participants with incomplete or no responses. In the first round, experts were asked to rate caesarean-related PPH definitions, detection methods, thresholds to trigger treatments, and first response interventions. In the second round, experts received their previous individual ratings and group rating distributions. They were asked to re-rate detection methods with disagreement, rank-order the thresholds and first response treatments that had previously received high ratings, and rate new questions that emerged from experts' comments in open-ended questions from Round 1. In the third round, experts met for a two-day hybrid meeting to discuss areas of divergence between surveys' findings and to rate (anonymously) final sets of interventions. The agenda and questions guide used to facilitate the discussion are available in the Supplementary Materials (Supplementary File S2 and S3). Figure 1 outlines the process of consensus building.

Median group rating and disagreement index (DI) were calculated to summarise experts' ratings and to measure agreement. A DI < 1 indicated agreement, while a DI ≥ 1 indicated disagreement (19). The RAND/UCLA appropriateness scale was used to classify interventions as "appropriate", "inappropriate", or "uncertain" (19). Interventions with median ratings in the top third of the appropriateness scale (7–9) were classified as "appropriate"; those in the bottom third were classified as "inappropriate" (1–3); and those with intermediate median ratings were classified as "uncertain" (4–6). Domains with disagreement among the experts were also classified as "uncertain" (Supplementary Figure S1).

For peer review only - http://bmjopen.bmj.com/site/about/guidelines.xhtml

Box 1. Themes explored, and criteria used to guide assessments

Themes	Criteria and items included in each questionnaire
**PPH definitions**	• Appropriateness of using a single definition for PPH, regardless of mode of birth<br>• Timeframe for postoperative PPH
**Early detection methods**<br>*Intra- and postoperative*	**Criteria:** clinical usefulness, feasibility of use in all settings attending caesarean birth, acceptability to key stakeholders, and estimate of resources required<br>**Items**:<br>• Visual estimation of blood loss<br>• Volumetric assessment of blood loss<br>• Gravimetric assessment of blood loss<br>• Clinical signs of haemodynamic instability<br>• Visual charts and early warning scores<br>• Clinical judgement (e.g., rate of flow, duration)<br>• Volumetric + gravimetric assessment of blood loss<br>• Volumetric/gravimetric assessment of blood loss + clinical signs of haemodynamic instability<br>• Visual estimation + visual charts/early warning systems (EWS)
**Thresholds for action**<br>*Intra- and postoperative*	**Criteria:** accuracy, feasibility of use in all settings attending caesarean birth, and acceptability to key stakeholders<br>**Items:**<br>• ***One step approach*** (Single threshold triggers full response protocol)<br>○ At least 500 mL blood loss alone<br>○ At least 1000 mL blood loss alone<br>○ Haemodynamic instability alone<br>○ At least 500 mL blood loss OR signs of haemodynamic instability<br>○ At least 1000 mL blood loss OR signs of haemodynamic instability<br>• ***Two step approach*** (Lower threshold triggers further assessment, preparedness, and close monitoring; higher threshold triggers initiation of treatment)<br>○ Lower threshold of at least 500 mL, and higher threshold of at least 1000 mL blood loss OR signs of haemodynamic instability<br>○ Lower threshold of at least 1000 ml, and higher threshold of at least 2000 mL blood loss OR signs of haemodynamic instability
**First response interventions**<br>*Intra- and postoperative*	**Criteria**: balance of effects, feasibility of use in all settings attending caesarean birth, acceptability to key stakeholders, estimate of resources required, equity<br>**Items:**<br>• Oxytocin<br>• Carbetocin<br>• Tranexamic acid<br>• Compressive sutures<br>• Bimanual compression<br>• Uterine massage<br>• Oxytocin-ergometrine fixed dose<br>• Prostaglandin<br>• Ergometrine<br>• Non-pneumatic anti-shock garment<br>• External aortic compression<br>• Intrauterine balloon tamponade

Note: NA=not applicable

For peer review only - http://bmjopen.bmj.com/site/about/guidelines.xhtml

RESULTS

The systematic search identified 802 guidelines and systematic reviews. After screening and quality appraisal, 17 guidelines (2,13,15,20–33), four systematic reviews (34–37) and 15 peer-reviewed studies were included (38–53) (Supplementary Figure S2). Included guidelines and systematic reviews identified six PPH definitions, five PPH detection methods, ten blood loss collection devices, seven thresholds to initiate treatment, and 14 interventions to conservatively manage PPH. Results are in Supplementary Tables S1-S4.

Of 25 experts invited, 22 agreed to participate in the Delphi process (Supplementary File S4). All completed the first and second rounds, while 20/22 participated and voted in the third round. The experts who completed all rounds were from 11 countries from all WHO world regions (six from the African Region, one from the Eastern Mediterranean Region, three from the European Region, six from the Region of the Americas, two from the South-East Asian Region, and two from the Western Pacific Region). They had different professional backgrounds (obstetricians and gynaecologists, anaesthetists, surgeons, nurse-midwives, and midwives) and were gender-balanced (12 men and 10 women). In addition, four observers participated in the discussion during the third round but did not vote.

The median ratings and measures of agreement obtained from the first and second rounds of online surveys are given in Supplementary Tables S5-S8 and Figure S3. Experts' ratings and agreements in the third round are given in Table 1. Consensus was reached for (a) using a single definition for PPH, regardless of mode of birth, (b) early detection of PPH at caesarean birth and thresholds to initiate treatment in the intraoperative phase, (c) clinical interventions for first response management of intraoperative PPH, and (d) early detection of PPH after caesarean birth and thresholds to initiate treatment in the postoperative phase. However, the first response treatment in the postoperative phase was determined to require an individualised approach.

For peer review only

For peer review only - http://bmjopen.bmj.com/site/about/guidelines.xhtml

Table 1. Experts' ratings and agreement on early detection and first response to intraoperative and postoperative PPH

Set of clinical interventions	Rating distribution			Agreement			
	# votes in each interval			Median (IQR) in a 1 - 9 differential scale	RAND DI	Qualitative scale	Appropriateness scale
	1 - 3	4 - 6	7 - 9				
**Intraoperative**							
Early detection of PPH in all women having caesarean birth							
Quantitative blood loss measurement and monitoring of haemodynamic status	0	1	19	**8 (1)**	**-0.34**	**Yes**	**Appropriate**
Thresholds for triggering action							
Option 1: At least 500 mL with continued bleeding OR clinical signs of haemodynamic instability	1	3	16	**8 (1)**	**-0.13**	**Yes**	**Appropriate**
Option 2: At least 750 mL with continued bleeding OR clinical signs of haemodynamic instability	7	4	9	6 (5)	3.13	No	Uncertain
**Postoperative**							
Early detection of PPH in all women having caesarean birth							
Monitoring of haemodynamic status and quantitative blood loss measurement if feasible	0	2	18	**8 (1)**	**-0.34**	**Yes**	**Appropriate**
Thresholds for triggering action	Not applicable						

Notes: [a]RAND DI: The disagreement index is a continuous scale used to measure the dispersion of experts' ratings, taken as an indicator of the level of agreement; [b]Agreement: A DI < 1 represents an agreement, while a DI ≥ 1 indicates disagreement; [c]Appropriateness: Items are classified as "appropriate" with median ratings in the top (median between 7-9) third and agreement, and as "uncertain" with intermediate median ratings (median between 4-6) or with disagreement.

For peer review only - http://bmjopen.bmj.com/site/about/guidelines.xhtml

Definition of PPH during and after caesarean birth

The experts agreed that a single definition of PPH, regardless of mode of birth (median rating 7.5; DI -5.23). Specifically, they agreed that the definition of PPH during and after caesarean birth should be the same as the definition of PPH related to vaginal birth, to underscore the importance of rapid action to address excessive bleeding.

Intraoperative phase

Early detection of PPH during caesarean birth and thresholds for triggering action

Experts agreed that during caesarean birth, blood loss should be assessed via quantitative measurement, complemented by ongoing monitoring of the woman's haemodynamic status (median rating 8; DI -0.34). Further, quantitative measurement and monitoring should be conducted for all women having a caesarean birth (Box 2). They noted the importance of distinguishing blood from amniotic fluid. This might be achieved by using separate suction canisters or measuring and recording the amount of amniotic fluid within the canister immediately after the birth and before delivery of the placenta.

The experts agreed that first response treatment should be triggered if the woman has lost at least 500 mL blood and still has continued bleeding or if she exhibits clinical signs of haemodynamic instability, whichever occurs first (median rating 8; DI -0.13). Such early action was considered important to prevent severe PPH and associated morbidity, because measurement of blood loss lags actual blood loss. Rapid response has been identified as a critical component of the effectiveness of an early detection and PPH treatment strategy to prevent severe PPH in vaginal births (40). Experts considered that rapid response is particularly important in settings with high prevalence of anaemia. It was noted that the proposed threshold for triggering action may result in many women receiving first response treatment for PPH. Some experts pointed out that this could diminish providers' responsiveness and recognition of PPH as a serious complication.

For peer review only - http://bmjopen.bmj.com/site/about/guidelines.xhtml

Box 2. Agreed early detection of PPH during caesarean birth and thresholds for triggering first response in the intraoperative phase

- Early detection of PPH during caesarean birth
  - Quantitative measurement of blood loss
    - Volumetric measurement alone if feasible (able to capture all blood)
    - Volumetric measurement + gravimetric measurement
  - Monitor haemodynamic status
- Thresholds for triggering first response
  - At least 500 mL measured blood loss WITH continued active bleeding
    OR
  - Clinical signs of haemodynamic instability

Additional comments

It is important to separate/distinguish amniotic fluid from blood.

To prevent severe PPH, first response management should be triggered early if there is still continued bleeding, particularly in settings with a high prevalence of anaemia or where unavoidable delays implementing treatment are anticipated.

First response management: intraoperative phase

The agreed first response management is summarised in Box 3. Specifically, the experts agreed clinicians should commence an infusion of oxytocin. If a prophylactic or other oxytocin infusion is already in place, the anaesthetist should quickly maximise the oxytocin dose as increasing uterine tone helps to reduce bleeding from the incision. If atony is diagnosed or the bleeding continues, the anaesthetist should rapidly add in a different uterotonic for treatment. The experts noted that this should occur quickly, rather than waiting to see whether the bleeding is responsive to oxytocin. They also agreed that tranexamic acid (TXA) should be administered as first response treatment, unless the woman had already received TXA within the last 30 minutes. Next, the team should carefully examine the woman to determine the source(s) of bleeding and initiate a cause-specific response. If the bleeding is due to trauma, the surgical team should close the uterus, repair any tears, and attend to the wound. If the bleeding is due to uterine atony, the surgical team should control bleeding mechanically with intra-abdominal uterine massage or massage the exteriorised uterus, as the anaesthetic team manages uterotonic administration, as previously described. Some experts noted that the assessment of atonic PPH may require the lifting of surgical drapes to assess vaginal blood loss. Experts highlighted that bleeding may be due to a combination of trauma and uterine atony; in such cases the team should take

For peer review only - http://bmjopen.bmj.com/site/about/guidelines.xhtml

a comprehensive approach. The experts also highlighted the importance of exteriorising and examining the posterior side of the uterus for tears and occult uterine rupture.

Surgical and anaesthetic teams should mobilise to administer the surgical and medical first responses concurrently. Team communication can be challenging and should be practised in drills to develop effective messages that will not alarm women. Teams should immediately call for senior assistance when necessary.

Experts also noted that anaesthetic teams should give IV fluids as needed for haemodynamic maintenance, according to the clinical condition and estimated blood loss. The literature is unclear regarding which specific fluids should be used, the optimal volumes, and how best to monitor haemodynamic status. Until further evidence is available, experts advised each setting to develop or follow local protocols.

Finally, experts acknowledged that this first response approach is intended to be appropriate for most cases of intraoperative PPH. There may be some cases that, due to quantity and rapidity of blood loss, require an individualised approach.

For peer review only

For peer review only - http://bmjopen.bmj.com/site/about/guidelines.xhtml

Box 3. Agreed upon first response treatment for PPH during the intraoperative phase

At least 500 mL measured blood loss WITH ongoing bleeding OR clinical signs of haemodynamic instability:

- If already infusing oxytocin, maximise dose OR add alternative uterotonic. If not already infusing, commence oxytocin infusion.
- TXA (1g in 10 mL IV over 10 min), if not already administered within the last 30 minutes.
- Examine and rapidly initiate cause-specific response:
    - If from incision or surgical trauma: Rapid haemostasis: close uterus, repair tears, attend to the wound.
    - If atony/placental cause: Uterotonics (as above) and control bleeding mechanically with intra-abdominal uterine massage or exteriorise the uterus and massage.

Additional comments

All blood loss may not be immediately obvious. Examine the posterior side of the uterus for cervical tears and occult uterine rupture, and lift the drapes to assess vaginal blood loss.

Medical and surgical first responses should be administered concurrently, and effective team communication is key.

Replace IV fluids as needed for haemodynamic maintenance, according to the clinical condition, estimated blood loss, and local protocols.

TXA should be administered as first response treatment, unless the woman has already received TXA for PPH prevention or treatment within the last 30 minutes. Up to 2 doses of TXA, at least 30 minutes apart may be administered.

If atony is diagnosed or the bleeding continues after the oxytocin dose has been maximised, the anaesthetists should rapidly add in a different uterotonic for treatment.

Due to quantity and rapidity of blood loss, there may be some cases that require an individualised approach.

Postoperative phase

Early detection of PPH after caesarean birth and thresholds for triggering action

Postoperative detection of PPH based on monitoring blood loss can be misleading because of internal bleeding. Thus, during this phase, experts agreed (median rating 8, DI -0.34) that blood loss should be assessed primarily through frequent monitoring of women's haemodynamic status (when possible, at least every 15 minutes for the first 2 hours) and clinical signs and symptoms of internal bleeding (e.g., assessment of fundal height) (Box 4). Some experts noted that postoperative monitoring for at least 30 minutes after caesarean birth should occur in a designated recovery area to ensure the woman's

For peer review only - http://bmjopen.bmj.com/site/about/guidelines.xhtml

safety. If internal bleeding is suspected, experts recommended an urgent ultrasound assessment if available. In addition, if the assessment of postoperative vaginal blood loss is feasible, either by quantitative measurement or estimation (e.g., counting pads), it should be performed. Experts agreed that, when possible, measured postoperative blood loss should be added to the quantified intraoperative blood loss, though they acknowledged that this may be challenging in some settings. Experts noted that cumulative intraoperative and postoperative blood loss, together with a woman's haemodynamic status, can help adjust the frequency and characteristics of postoperative monitoring and thresholds for action. For example, a woman who experienced substantial blood loss intraoperatively may require more frequent monitoring than the baseline every 15 minutes.

The experts acknowledged that haemodynamic parameters for postoperative thresholds to trigger treatment are still unclear. Until further evidence is available, each setting should develop or follow their local protocols. The Obstetric Shock Index (heart rate divided by systolic blood pressure; OSI) has been used in some settings, but there is not yet clear evidence on appropriate cut-off points.

For peer review only - http://bmjopen.bmj.com/site/about/guidelines.xhtml

Box 4. Agreed early detection of postoperative PPH and thresholds for triggering first response

- Early detection of PPH
  - Frequent monitoring of haemodynamic status (at least every 15 minutes for the first 2 hours)
    - Heart rate
    - Blood pressure
    - Shock index
    - Clinical signs/symptoms suspicious of internal bleeding
  - Quantitative blood loss assessment, if feasible
    - Measured or estimated postoperative blood loss (when possible, added to quantified intraoperative blood loss)
- Thresholds for triggering first response management
  - Clinical signs and symptoms of haemodynamic instability, in accordance with local protocols

---

Additional comments

Relying on postoperative blood loss alone can underestimate internal bleeding. Increase vigilance and assess hemodynamic status frequently.

Early detection of postoperative PPH should mainly rely on frequent monitoring of haemodynamic status and clinical signs and symptoms of internal bleeding. If assessment of postoperative vaginal blood loss is feasible, either by quantitative measurement or estimation (e.g., counting pads), it should be performed.

When possible, assessed postoperative blood loss should be added to the quantified intraoperative blood loss.

The cumulative intraoperative and postoperative blood loss, together with a woman's haemodynamic status, may better determine the frequency and characteristics of postoperative monitoring and thresholds for action.

Hemodynamic parameter thresholds for vital signs and Obstetric Shock Index to trigger treatment are not yet agreed upon

Postoperative phase: First response management

The experts noted that the follow-on postoperative treatment approach may vary substantially according to many factors, including the woman's baseline risk, anaemia, whether intraoperative PPH occurred, the woman's postoperative haemodynamic status, and clinical signs and symptoms of internal bleeding (e.g., assessment of fundal height; if available, ultrasound, paracentesis). Until further evidence is available, experts recommended that local protocols be developed that consider these factors, rather than relying on a common postoperative first response approach for all cases and settings.

Experts' final comments

For peer review only - http://bmjopen.bmj.com/site/about/guidelines.xhtml

The experts recognised that detection methods and first response interventions for PPH are essential for the care of all women having a caesarean birth, regardless of their risk status. However, women at high risk of developing PPH may require additional specialised monitoring and care.

In addition, given that PPH can arise intra- or postoperatively for any woman, strategies for early detection of PPH should be incorporated into routine practice alongside PPH prevention and risk assessment.

Finally, experts highlighted two cross-cutting remarks regarding PPH during and after caesarean birth. First, good surgical practices, as recommended by the WHO Guidelines for Safe Surgery, should be followed to prepare for, perform, and follow-up caesarean births (54). The routine use of WHO surgical safety checklists have proven beneficial in reducing perioperative complications (55). Second, it was noted that teamwork, communication, and cooperation are critical. Effectively implementing the early detection and first response interventions described will require training, supportive supervision, monitoring, and evaluation.

DISCUSSION

Main findings

Expert consensus on optimal approaches for detecting and managing PPH during and after caesarean birth was developed among an international panel. Through two systematic reviews and a three-round modified Delphi process, consensus was reached for (a) using a single definition for PPH, regardless of the mode of birth, (b) early detection of PPH during caesarean birth and thresholds to initiate treatment in the intraoperative phase, (c) clinical interventions for first response to intraoperative PPH, and (d) early detection of PPH after caesarean birth and threshold to initiate treatment in the postoperative phase. First response treatment in the postoperative phase was determined to require an individualised approach.

Strengths and limitations

Study strengths include the use of a rigorous and systematic process to identify and synthesise PPH evidence in the literature. We conducted in-depth systematic reviews with detailed quality appraisals to ensure that we used only high-quality evidence to identify approaches for PPH detection and management interventions. The selection of the expert panellists ensured a wide range of perspectives,

For peer review only - http://bmjopen.bmj.com/site/about/guidelines.xhtml

to enhance the utility and applicability of this consensus to a wide range of clinical settings. There was a low rate of loss to follow-up. The first two rounds of the modified-Delphi process were blinded, to avoid social acceptability bias, and the hybrid meeting was facilitated by members of the Steering Group, to ensure that all panellists had equal opportunity to contribute to the discussion. The staged modified-Delphi process allowed ample time for discussion and input, and experts provided additional comments to refine the final statements for clarity and accuracy.

Limitations included a dearth of quality evidence on PPH related to caesarean birth. Despite ample evidence on PPH during and after vaginal birth, there is far less published evidence on caesarean birth. Often, the experts had to extrapolate from evidence on interventions recommended for PPH in vaginal birth and make decisions based on their experiences, expert opinions, and best practices, rather than evidence from comparative research. In some cases, this led to omitting interventions that might be useful for early detection or first-response management because there was no rigorous evidence available. It is also a limitation that, given the highly technical content, we did not include recipients of these interventions, or their representatives, among the panellists.

Additionally, since this systematic review of guidelines was conducted, three updated PPH guidelines have been published (56–58). None of these guidelines are specific to PPH at caesarean birth, though all contain some guidance relevant to PPH during or after caesarean birth. The recommendations within these guidelines generally align with previously published guidance included in our study, with a few notable exceptions. The revised 2023 FIGO PPH guideline recommends the use of the Obstetric Shock Index (with a threshold of ≥0.9 triggering first-response treatment), together with the rule of 30, while acknowledging that “the association between shock parameters and advanced treatment modalities in severe PPH has yet to be reported” (59). In the updated CMQCC Obstetric Hemorrhage Toolkit, greater emphasis is placed on assessing for concealed haemorrhage. The guideline recommends using a combination of clinical signs of hypovolemia, the SI, and Early Warning Score to enable earlier postoperative PPH detection (57). The Royal College of Physicians of Ireland guideline suggests that prophylactic tranexamic acid administration be considered in women at high PPH risk (58). The timing of our study prevented us from incorporating these revisions into our systematic review.

Interpretation

For peer review only - http://bmjopen.bmj.com/site/about/guidelines.xhtml

This expert consensus aligns with the recent expert consensus developed by the African Perioperative Research Group (APORG) Caesarean Delivery Haemorrhage Group (60) for clinicians working in Africa. The APORG expert consensus had a broader scope, encompassing antenatal and perioperative prevention, preparedness, first response, and refractory treatment interventions, as well as community- and health system-level indirect interventions. This present expert consensus focuses only on early detection and first response, including specific thresholds for triggering action.

With rates of caesarean birth rising globally, particularly in middle-income countries (6), this research is timely and crucial. International initiatives are underway to end preventable deaths due to PPH, such as the Roadmap to Combat Postpartum Haemorrhage between 2023 and 2030 (61), and the Pan American Health Organization's Zero Maternal Deaths by Hemorrhage campaign (62). The present expert consensus on early detection and first-response treatment for PPH at caesarean birth adds to existing efforts by clearly delineating how interventions need to be tailored for the context of caesarean birth. This consultation represents an important first step toward developing standardised strategies for reducing morbidity and mortality related to PPH during and after caesarean birth. Determining how best to implement these standardised strategies is a critical next step.

Insights from implementation science suggest that defining evidence-based interventions is a necessary but insufficient step towards changing clinical practice (63). Establishing implementation approaches is believed to increase uptake and fidelity of evidence-based interventions (64). Clinical bundles are one implementation approach that has gained traction in recent years (56,65–68). Global evidence suggests that clinical bundles are a powerful implementation approach for early detection and first response for PPH after vaginal birth (68,69). However, it is unclear whether a bundle is the most appropriate implementation approach for PPH during and after caesarean birth. Bundles require a set of interventions to be administered together, but the administration of some of the clinical interventions outlined here may depend on what occurs during surgery and what other interventions may already have been administered. As such, other implementation approaches, such as algorithms, protocols, or checklists, might be more appropriate (69,70). Defining the optimal implementation approach for early detection and first response management of PPH during and after caesarean birth still remains to be completed. Conducting necessary research to answer this question should be an immediate next step.

In addition, efforts should be pursued to agree on standardised approaches for the management of refractory PPH during and after caesarean birth. These standardised approaches should encompass both

For peer review only

For peer review only - http://bmjopen.bmj.com/site/about/guidelines.xhtml

the specific interventions used to manage refractory PPH and the implementation strategies to support their uptake and sustainability. Standardised approaches will need to be applicable to a variety of settings, including those with limited access to medical specialists.

CONCLUSION

This expert consensus proposes strategies for early detection and first response to PPH during and after caesarean birth. Future research should determine how best to implement these strategies and evaluate the effectiveness of the proposed implementation approach. Such research should be conducted soon, so that the approaches and interventions proposed here can rapidly be operationalised and institutionalized to contribute to the global efforts to reduce maternal death and disability.

CONTRIBUTION TO AUTHORSHIP: SM, FA, AC, IG, and OTO conceived the idea. VP developed the protocol with input from SM and FA. SM, FA, CRW, and VP coordinated the project with input from IG and AC. FM, AL, VO, AB, CRW, and VP conducted the systematic review. OTO, IG, AC, EA, SA, NA, FAA, BC, CDT, SD, AD, MFEV, CE, SF, HG, JH, CH, TL, PL, EM, FM, IN, OJ, QPNP, ZQ, CS, JV, AW, FA, SM, CRW, and VP participated in the consensus and provided expert input at various stages of the project. Data were analysed by CRW and VP with input from SM and FA. SM, FA, CRW, and VP wrote the manuscript with input from all co-authors. This manuscript represents the views of the authors and not the views of the World Health Organization or the UNDP/UNFPA/UNICEF/WHO/World Bank Special Programme of Research, Development and Research Training in Human Reproduction (HRP).

ETHICAL CONSIDERATIONS: Authors confirm that this exercise was conducted according to relevant guidelines and regulations with respect to the Helsinki Declaration. Experts were advised that participation was voluntary and indicated informed consent. Given the consultative nature of the exercise which involved consenting experts in their work capacity, ethical clearance was not required.

FUNDING: This study was funded by the Bill & Melinda Gates Foundation (INV-001393) through a grant to the University of Birmingham, United Kingdom, and the UNDP/UNFPA/UNICEF/WHO/World Bank Special Programme of Research, Development and Research Training in Human Reproduction (HRP), a cosponsored program executed by the WHO.

For peer review only - http://bmjopen.bmj.com/site/about/guidelines.xhtml

DATA AVAILABILITY: Extensive data are presented in the supplementary materials. Given the small sample size, datasets will be available upon reasonable request.

CONSENT FOR PUBLICATION: All authors consented to publication.

PATIENT AND PUBLIC PARTNERSHIP: Given the highly technical content, we did not include recipients of these interventions, or their representatives, among the panellists. This limitation is addressed in the discussion.

COMPETING INTERESTS: Disclosure forms provided by the authors are available with the full text of this article.

ACKNOWLEDGMENTS: We would like to acknowledge the contribution of Bill & Melinda Gates Foundation officers for conceiving the research question and supporting the conduct of this study; the contribution of Zhitong Yu in preparing the consensus building process and the administrative support of Chastine Sebolino and Maria Harmitton Oliveto.

This article reflects the views of the named authors, and not the views of their organisations, or the views of UNDP-UNFPA-UNICEF-WHO-World Bank Special Programme of Research, Development and Research Training in Human Reproduction (HRP) or World Health Organization.

For peer review only

For peer review only - http://bmjopen.bmj.com/site/about/guidelines.xhtml

REFERENCES

1. Say L, Chou D, Gemmill A, Tunçalp Ö, Moller A-B, Daniels J, et al. Global causes of maternal death: a WHO systematic analysis. Lancet Glob Health [Internet]. 2014 Jun;2(6):e323-33. Available from: http://dx.doi.org/10.1016/S2214-109X(14)70227-X

2. World Health Organization(WHO). WHO recommendations for the prevention and treatment of postpartum haemorrhage. Genève, Switzerland: World Health Organization; 2012. 44 p.

3. Butwick AJ, Ramachandran B, Hegde P, Riley ET, El-Sayed YY, Nelson LM. Risk factors for severe postpartum hemorrhage after cesarean delivery: Case-control studies. Anesth Analg [Internet]. 2017 Aug;125(2):523–32. Available from: http://dx.doi.org/10.1213/ANE.0000000000001962

4. Betran AP, Ye J, Moller A-B, Souza JP, Zhang J. Trends and projections of caesarean section rates: global and regional estimates. BMJ Glob Health [Internet]. 2021 Jun;6(6):e005671. Available from: http://dx.doi.org/10.1136/bmjgh-2021-005671

5. Sobhy S, Arroyo-Manzano D, Murugesu N, Karthikeyan G, Kumar V, Kaur I, et al. Maternal and perinatal mortality and complications associated with caesarean section in low-income and middle-income countries: a systematic review and meta-analysis. Lancet [Internet]. 2019 May 11;393(10184):1973–82. Available from: http://dx.doi.org/10.1016/S0140-6736(18)32386-9

6. Al-Zirqi I, Vangen S, Forsén L, Stray-Pedersen B. Effects of onset of labor and mode of delivery on severe postpartum hemorrhage. Obstet Anesth Dig [Internet]. 2010 Sep;30(3):150–1. Available from: http://dx.doi.org/10.1097/01.aoa.0000386819.15311.cb

7. Miller S, Abalos E, Chamillard M, Ciapponi A, Colaci D, Comandé D, et al. Beyond too little, too late and too much, too soon: a pathway towards evidence-based, respectful maternity care worldwide. Lancet [Internet]. 2016 Oct;388(10056):2176–92. Available from: http://dx.doi.org/10.1016/s0140-6736(16)31472-6

8. Chestnut DH. Postpartum hemorrhage – update on problems of definitions and diagnosis. Yearb Anesthesiol Pain Manag [Internet]. 2012 Jan;2012:322–3. Available from: http://dx.doi.org/10.1016/j.yane.2011.07.002

9. Widmer M, Piaggio G, Nguyen TMH, Osoti A, Owa OO, Misra S, et al. Heat-stable carbetocin versus oxytocin to prevent hemorrhage after vaginal birth. N Engl J Med [Internet]. 2018 Jun 27;(NEJMoa1805489). Available from: http://dx.doi.org/10.1056/nejmoa1805489

10. Gallos I, Devall A, Martin J, Middleton L, Beeson L, Galadanci H, et al. Randomized trial of early detection and treatment of postpartum hemorrhage. N Engl J Med [Internet]. 2023 May 9; Available from: http://dx.doi.org/10.1056/NEJMoa2303966

11. Shields LE, Wiesner S, Fulton J, Pelletreau B. Comprehensive maternal hemorrhage protocols reduce the use of blood products and improve patient safety. Am J Obstet Gynecol [Internet]. 2015 Mar;212(3):272–80. Available from: http://dx.doi.org/10.1016/j.ajog.2014.07.012

For peer review only - http://bmjopen.bmj.com/site/about/guidelines.xhtml

12. Main EK, Cape V, Abreo A, Vasher J, Woods A, Carpenter A, et al. Reduction of severe maternal morbidity from hemorrhage using a state perinatal quality collaborative. Am J Obstet Gynecol [Internet]. 2017 Mar;216(3):298.e1-298.e11. Available from: http://dx.doi.org/10.1016/j.ajog.2017.01.017

13. World Health Organization. WHO Recommendation on Tranexamic Acid for the Treatment of Postpartum Haemorrhage. https://www.who.int/publications/i/item/9789241548502; 2017.

14. World Health Organization. WHO recommendation on uterine balloon tamponade for the treatment of postpartum haemorrhage [Internet]. Genève, Switzerland: World Health Organization; 2021 [cited 2023 Apr 30]. 85 p. Available from: https://apps.who.int/iris/handle/10665/340796

15. World Health Organization. Managing complications in pregnancy and childbirth. 2nd ed. Genève, Switzerland: World Health Organization; 2017. 488 p.

16. Althabe F, Therrien MNS, Pingray V, Hermida J, Gülmezoglu AM, Armbruster D, et al. Postpartum hemorrhage care bundles to improve adherence to guidelines: A WHO technical consultation. Int J Gynaecol Obstet [Internet]. 2020 Mar;148(3):290–9. Available from: http://dx.doi.org/10.1002/ijgo.13028

17. Brouwers MC, Kho ME, Browman GP, Burgers JS, Cluzeau F, Feder G, et al. AGREE II: advancing guideline development, reporting and evaluation in health care. CMAJ [Internet]. 2010 Dec 14;182(18):E839-42. Available from: http://dx.doi.org/10.1503/cmaj.090449

18. Shea BJ, Reeves BC, Wells G, Thuku M, Hamel C, Moran J, et al. AMSTAR 2: a critical appraisal tool for systematic reviews that include randomised or non-randomised studies of healthcare interventions, or both. BMJ [Internet]. 2017 Sep 21;j4008. Available from: http://dx.doi.org/10.1136/bmj.j4008

19. Fitch K, Bernstein SJ, Aguilar MD. The rand/UCLA appropriateness method user's manual. Santa Monica, CA: RAND; 1999. 133 p.

20. NICE. Overview | Caesarean birth | Guidance | NICE. [cited 2023 Apr 30]; Available from: https://www.nice.org.uk/guidance/ng192

21. ACOG. Quantitative blood loss in obstetric hemorrhage. Obstet Gynecol [Internet]. 2019 Dec;134(6):e150–6. Available from: http://dx.doi.org/10.1097/aog.0000000000003564

22. Leduc D, Senikas V, Lalonde AB. No. 235-active management of the third stage of labour: Prevention and treatment of postpartum hemorrhage. J Obstet Gynaecol Can [Internet]. 2018 Dec;40(12):e841–55. Available from: http://dx.doi.org/10.1016/j.jogc.2018.09.024

23. RANZCOG Women's Health Committee. Management of Postpartum Haemorrhage [Internet]. RANZCOG; 2016. Available from: chrome-extension://efaidnbmnnnibpcajpcglclefindmkaj/https://ranzcog.edu.au/wp-content/uploads/2022/05/Management-of-Postpartum-Haemorrhage-PPH.pdf

24. Queensland Clinical Guidelines. Postpartum haemorrhage Guideline. chrome-extension://efaidnbmnnnibpcajpcglclefindmkaj/https://www.health.qld.gov.au/__data/assets/pdf_file/0015/140136/g-pph.pdf; 2021.

For peer review only - http://bmjopen.bmj.com/site/about/guidelines.xhtml

25. ACOG. Postpartum Hemorrhage [Internet]. [cited 2023 May 1]. Available from: https://www.acog.org/clinical/clinical-guidance/practice-bulletin/articles/2017/10/postpartum-hemorrhage

26. Muñoz M, Stensballe J, Ducloy-Bouthors A-S, Bonnet M-P, De Robertis E, Fornet I, et al. Patient blood management in obstetrics: prevention and treatment of postpartum haemorrhage. A NATA consensus statement. Blood Transfus [Internet]. 2019 Mar;17(2):112–36. Available from: http://dx.doi.org/10.2450/2019.0245-18

27. Sentilhes L, Vayssière C, Deneux-Tharaux C, Aya AG, Bayoumeu F, Bonnet M-P, et al. Postpartum hemorrhage: guidelines for clinical practice from the French College of Gynaecologists and Obstetricians (CNGOF): in collaboration with the French Society of Anesthesiology and Intensive Care (SFAR). Eur J Obstet Gynecol Reprod Biol [Internet]. 2016 Mar;198:12–21. Available from: http://dx.doi.org/10.1016/j.ejogrb.2015.12.012

28. Schlembach D, Helmer H, Henrich W, von Heymann C, Kainer F, Korte W, et al. Peripartum Haemorrhage, Diagnosis and Therapy. Guideline of the DGGG, OEGGG and SGGG (S2k Level, AWMF Registry No. 015/063, March 2016). Geburtshilfe Frauenheilkd [Internet]. 2018 Apr;78(04):382–99. Available from: http://dx.doi.org/10.1055/a-0582-0122

29. RCOG. Prevention and management of postpartum haemorrhage (green-top guideline no. 52) [Internet]. RCOG. [cited 2023 May 1]. Available from: https://www.rcog.org.uk/guidance/browse-all-guidance/green-top-guidelines/prevention-and-management-of-postpartum-haemorrhage-green-top-guideline-no-52/

30. Institute of Obstetricians and Gynaecologists. Royal College of Physicians of Ireland and Directorate of Strategy and Clinical Programmes. Health Service Executive. CLINICAL PRACTICE GUIDELINE: PREVENTION AND MANAGEMENT OF PRIMARY POSTPARTUM HAEMORRHAGE [Internet]. 2014 Jun. Available from: https://www.hse.ie/eng/about/who/acute-hospitals-division/woman-infants/clinical-guidelines/

31. Lalonde A, International Federation of Gynecology and Obstetrics. Prevention and treatment of postpartum hemorrhage in low-resource settings. Int J Gynaecol Obstet [Internet]. 2012 May;117(2):108–18. Available from: http://dx.doi.org/10.1016/j.ijgo.2012.03.001

32. Lyndon A, Lagrew D, Shields L, Main E, Cape V. Improving Health Care Response to Obstetric Hemorrhage. California Department of Public Health; Maternal, Child and Adolescent Health Division; 2015.

33. Minakami H, Maeda T, Fujii T, Hamada H, Iitsuka Y, Itakura A, et al. Guidelines for obstetrical practice in Japan: Japan Society of Obstetrics and Gynecology (JSOG) and Japan Association of Obstetricians and Gynecologists (JAOG) 2014 edition. J Obstet Gynaecol Res [Internet]. 2014 Jun;40(6):1469–99. Available from: http://dx.doi.org/10.1111/jog.12419

34. Shakur H, Beaumont D, Pavord S, Gayet-Ageron A, Ker K, Mousa HA. Antifibrinolytic drugs for treating primary postpartum haemorrhage. Cochrane Database Syst Rev [Internet]. 2018 Feb 20;2:CD012964. Available from: http://dx.doi.org/10.1002/14651858.CD012964

For peer review only

35. Kellie FJ, Wandabwa JN, Mousa HA, Weeks AD. Mechanical and surgical interventions for treating primary postpartum haemorrhage. Cochrane Database Syst Rev [Internet]. 2020 Jul 1;7(7):CD013663. Available from: http://dx.doi.org/10.1002/14651858.CD013663

36. Parry Smith WR, Papadopoulou A, Thomas E, Tobias A, Price MJ, Meher S, et al. Uterotonic agents for first-line treatment of postpartum haemorrhage: a network meta-analysis. Cochrane Libr [Internet]. 2020 Nov 24;2020(11). Available from: http://dx.doi.org/10.1002/14651858.cd012754.pub2

37. Gallos ID, Williams HM, Price MJ, Merriel A, Gee H, Lissauer D, et al. Uterotonic agents for preventing postpartum haemorrhage: a network meta-analysis. Cochrane Libr [Internet]. 2018 Apr 25; Available from: http://dx.doi.org/10.1002/14651858.cd011689.pub2

38. Andrikopoulou M, D'Alton ME. Postpartum hemorrhage: early identification challenges. Semin Perinatol [Internet]. 2019 Feb;43(1):11–7. Available from: http://dx.doi.org/10.1053/j.semperi.2018.11.003

39. Atukunda EC, Mugyenyi GR, Obua C, Atuhumuza EB, Musinguzi N, Tornes YF, et al. Measuring post-partum haemorrhage in low-resource settings: The diagnostic validity of weighed blood loss versus quantitative changes in hemoglobin. PLoS One [Internet]. 2016 Apr 6;11(4):e0152408. Available from: http://dx.doi.org/10.1371/journal.pone.0152408

40. Bell SF, Watkins A, John M, Macgillivray E, Kitchen TL, James D, et al. Incidence of postpartum haemorrhage defined by quantitative blood loss measurement: a national cohort. BMC Pregnancy Childbirth [Internet]. 2020 May 6;20(1):271. Available from: http://dx.doi.org/10.1186/s12884-020-02971-3

41. Diaz V, Abalos E, Carroli G. Methods for blood loss estimation after vaginal birth. Cochrane Database Syst Rev [Internet]. 2018 Sep 13;9(9):CD010980. Available from: http://dx.doi.org/10.1002/14651858.CD010980.pub2

42. Hancock A, Weeks AD, Lavender DT. Is accurate and reliable blood loss estimation the "crucial step" in early detection of postpartum haemorrhage: an integrative review of the literature. BMC Pregnancy Childbirth [Internet]. 2015 Dec;15(1). Available from: http://dx.doi.org/10.1186/s12884-015-0653-6

43. Larsson C, Saltvedt S, Wiklund I, Pahlen S, Andolf E. Estimation of blood loss after cesarean section and vaginal delivery has low validity with a tendency to exaggeration. Acta Obstet Gynecol Scand [Internet]. 2006;85(12):1448–52. Available from: http://dx.doi.org/10.1080/00016340600985032

44. Rath WH. Postpartum hemorrhage update on problems of definitions and diagnosis. Obstet Anesth Dig [Internet]. 2012 Jun;32(2):76–7. Available from: http://dx.doi.org/10.1097/01.aoa.0000414055.79309.d6

45. Ambardekar S, Shochet T, Bracken H, Coyaji K, Winikoff B. Calibrated delivery drape versus indirect gravimetric technique for the measurement of blood loss after delivery: a randomized trial. BMC Pregnancy Childbirth [Internet]. 2014 Aug 15;14(1):276. Available from: http://dx.doi.org/10.1186/1471-2393-14-276

For peer review only - http://bmjopen.bmj.com/site/about/guidelines.xhtml

46. Buckland SS, Homer CSE. Estimating blood loss after birth: using simulated clinical examples. Women Birth [Internet]. 2007 Jun;20(2):85–8. Available from: http://dx.doi.org/10.1016/j.wombi.2007.01.001

47. Lertbunnaphong T, Lapthanapat N, Leetheeragul J, Hakularb P, Ownon A. Postpartum blood loss: visual estimation versus objective quantification with a novel birthing drape. Singapore Med J [Internet]. 2016 Jun;57(6):325–8. Available from: http://dx.doi.org/10.11622/smedj.2016107

48. Mhyre JM, D'Oria R, Hameed AB, Lappen JR, Holley SL, Hunter SK, et al. The maternal early warning criteria: a proposal from the national partnership for maternal safety. J Obstet Gynecol Neonatal Nurs [Internet]. 2014 Nov;43(6):771–9. Available from: http://dx.doi.org/10.1111/1552-6909.12504

49. Patel A, Goudar SS, Geller SE, Kodkany BS, Edlavitch SA, Wagh K, et al. Drape estimation vs. visual assessment for estimating postpartum hemorrhage. Int J Gynaecol Obstet [Internet]. 2006 Jun;93(3):220–4. Available from: http://dx.doi.org/10.1016/j.ijgo.2006.02.014

50. Schorn MN. Measurement of blood loss: review of the literature. J Midwifery Womens Health [Internet]. 2010 Jan;55(1):20–7. Available from: http://dx.doi.org/10.1016/j.jmwh.2009.02.014

51. Shields LE, Wiesner S, Klein C, Pelletreau B, Hedriana HL. Use of Maternal Early Warning Trigger tool reduces maternal morbidity. Am J Obstet Gynecol [Internet]. 2016 Apr;214(4):527.e1-527.e6. Available from: http://dx.doi.org/10.1016/j.ajog.2016.01.154

52. Umar A, Ameh CA, Muriithi F, Mathai M. Early warning systems in obstetrics: A systematic literature review. PLoS One [Internet]. 2019 May 31;14(5):e0217864. Available from: http://dx.doi.org/10.1371/journal.pone.0217864

53. Zhang W-H, Deneux-Tharaux C, Brocklehurst P, Juszczak E, Joslin M, Alexander S, et al. Effect of a collector bag for measurement of postpartum blood loss after vaginal delivery: cluster randomised trial in 13 European countries. BMJ [Internet]. 2010 Feb 1;340(feb01 1):c293. Available from: http://dx.doi.org/10.1136/bmj.c293

54. World Health Organization. Implementation manual: WHO surgical safety checklist (first edition) [Internet]. World Health Organization; 2008 [cited 2023 May 30]. Report No.: WHO/IER/PSP/2008.05. Available from: https://apps.who.int/iris/handle/10665/70046

55. Bergs J, Hellings J, Cleemput I, Zurel Ö, De Troyer V, Van Hiel M, et al. Systematic review and meta-analysis of the effect of the World Health Organization surgical safety checklist on postoperative complications. Br J Surg [Internet]. 2014 Feb;101(3):150–8. Available from: http://dx.doi.org/10.1002/bjs.9381

56. Escobar MF, Nassar AH, Theron G, Barnea ER, Nicholson W, Ramasauskaite D, et al. FIGO recommendations on the management of postpartum hemorrhage 2022. Int J Gynaecol Obstet [Internet]. 2022 Mar;157 Suppl 1(S1):3–50. Available from: http://dx.doi.org/10.1002/ijgo.14116

57. Lagrew D, McNulty J, Sakowski C, Cape V, McCormick E, Morton CH. Improving Health Care Response to Obstetric Hemorrhage, a California Maternal Quality Care Collaborative Toolkit. 2022; Available from: https://www.cmqcc.org/

For peer review only

58. Byrne B, Spring A, Barrett N, Power J, Mckernan J, Brophy D, et al. Ni Ainle F. National Clinical Practice Guideline: Prevention and Management of Primary Postpartum Haemorrhage. National Women and Infants Health Programme and The Institute of Obstetricians and Gynaecologists. 2022.

59. Morris JL, Winikoff B, Dabash R, Weeks A, Faundes A, Gemzell-Danielsson K, et al. FIGO's updated recommendations for misoprostol used alone in gynecology and obstetrics. Int J Gynaecol Obstet [Internet]. 2017 Sep;138(3):363–6. Available from: http://dx.doi.org/10.1002/ijgo.12181

60. APORG Caesarean Delivery Haemorrhage Group. Identifying interventions to reduce peripartum haemorrhage associated with caesarean delivery in Africa: A Delphi consensus study. PLOS Glob Public Health [Internet]. 2022 Aug 31;2(8):e0000455. Available from: http://dx.doi.org/10.1371/journal.pgph.0000455

61. A Roadmap to combat postpartum haemorrhage between 2023 and 2030. Geneva: World Health Organization. 2023.

62. Zero maternal deaths by hemorrhage [Internet]. [cited 2023 May 15]. Available from: https://www.paho.org/en/zero-maternal-deaths-hemorrhage

63. Bauer MS, Kirchner J. Implementation science: What is it and why should I care? Psychiatry Res [Internet]. 2020 Jan;283(112376):112376. Available from: http://dx.doi.org/10.1016/j.psychres.2019.04.025

64. Carroll C, Patterson M, Wood S, Booth A, Rick J, Balain S. A conceptual framework for implementation fidelity. Implement Sci [Internet]. 2007 Nov 30;2(1):40. Available from: http://dx.doi.org/10.1186/1748-5908-2-40

65. Main EK, Goffman D, Scavone BM, Low LK, Bingham D, Fontaine PL, et al. National Partnership for Maternal Safety consensus bundle on obstetric hemorrhage. J Midwifery Womens Health [Internet]. 2015 Jul;60(4):458–64. Available from: http://dx.doi.org/10.1111/jmwh.12345

66. Main EK, Goffman D, Scavone BM, Low LK, Bingham D, Fontaine PL, et al. National Partnership for Maternal Safety: consensus bundle on obstetric hemorrhage. Anesth Analg [Internet]. 2015 Jul;121(1):142–8. Available from: http://dx.doi.org/10.1097/AOG.0000000000000869

67. D'Alton ME, Friedman AM, Smiley RM, Montgomery DM, Paidas MJ, D'Oria R, et al. National Partnership for Maternal Safety: Consensus bundle on venous thromboembolism. J Obstet Gynecol Neonatal Nurs [Internet]. 2016 Sep;45(5):706–17. Available from: http://dx.doi.org/10.1016/j.jogn.2016.07.001

68. Levy MM, Rhodes A, Phillips GS, Townsend SR, Schorr CA, Beale R, et al. Surviving Sepsis Campaign: association between performance metrics and outcomes in a 7.5-year study. Crit Care Med [Internet]. 2015 Jan;43(1):3–12. Available from: http://dx.doi.org/10.1097/CCM.0000000000000723

69. Margolis CZ. Uses of clinical algorithms. JAMA [Internet]. 1983 Feb 4;249(5):627–32. Available from: http://dx.doi.org/10.1001/jama.249.5.627

For peer review only

For peer review only - http://bmjopen.bmj.com/site/about/guidelines.xhtml

70. Arora KS, Shields LE, Grobman WA, D'Alton ME, Lappen JR, Mercer BM. Triggers, bundles, protocols, and checklists--what every maternal care provider needs to know. Am J Obstet Gynecol [Internet]. 2016 Apr;214(4):444–51. Available from: http://dx.doi.org/10.1016/j.ajog.2015.10.011

For peer review only

For peer review only - http://bmjopen.bmj.com/site/about/guidelines.xhtml

Figure 1. Technical consultation flowchart

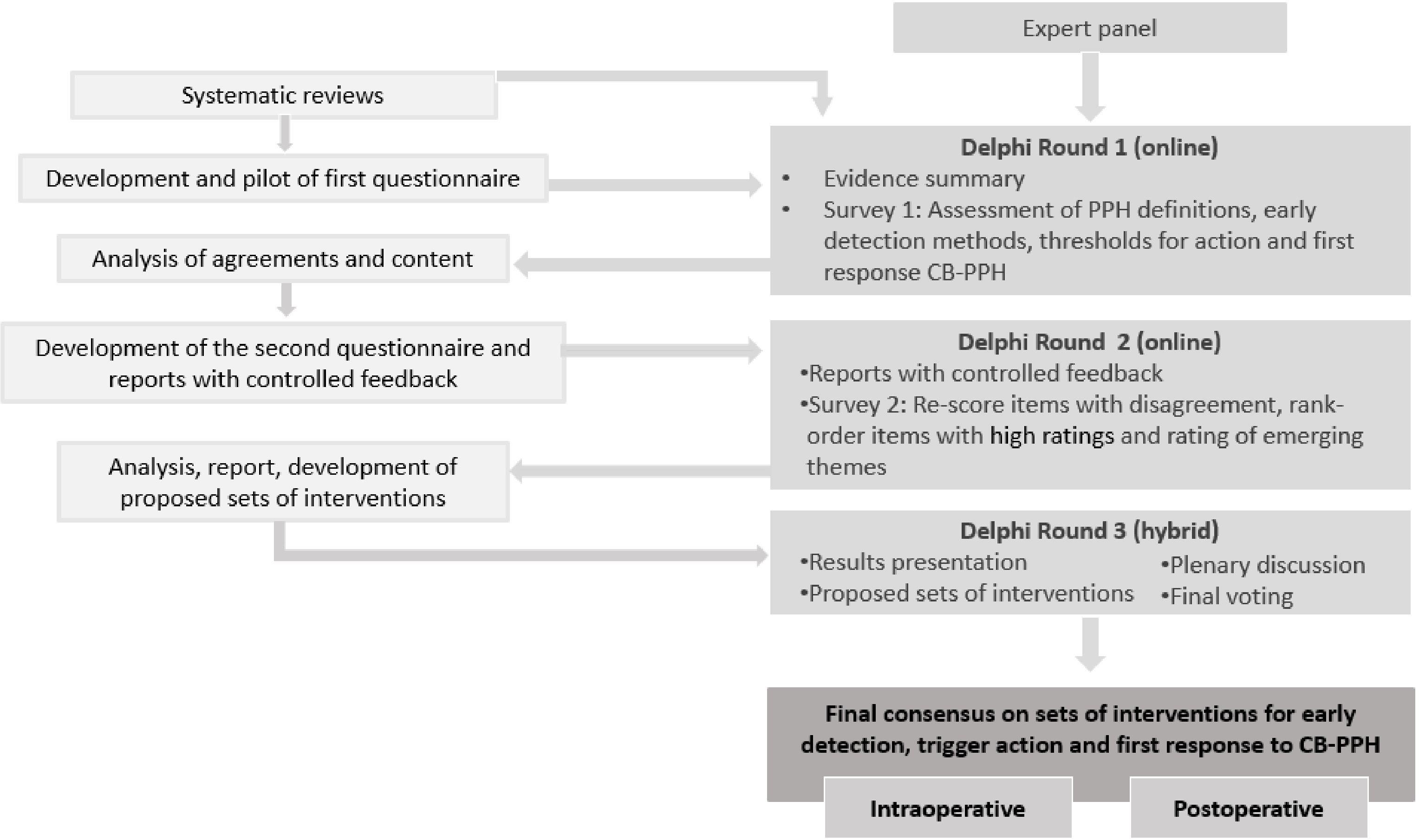

Strategies for optimising early detection and treatment of postpartum haemorrhage at caesarean birth: An international modified Delphi-based expert consensus

SUPPLEMENTARY MATERIALS

Supplementary Tables 2
Table S1. Definitions of PPH and severe PPH used in clinical guidelines 2
Table S2. Synthesis of evidence and considerations regarding approaches to detect intraoperative and postoperative caesarean PPH and haemodynamic instability 3
Table S3. Synthesis of evidence on thresholds for triggering action on PPH during and after caesarean birth 5
Table S4. Medical Interventions, manoeuvres, and procedures for PPH recommended by WHO compared to recommendations in other PPH guidelines and systematic reviews 7
Table S5. First and second round ratings and agreement for early detection methods for intraoperative and postoperative CB-PPH....................11
Table S6. First round ratings and agreement on threshold to initiate treatment for intraoperative CB PPH 12
Table S7. First round ratings and agreement for first response interventions for managing intraoperative and postoperative CB-PPH....................14
Table S8. Second round ratings and agreement for first response interventions for managing intraoperative and postoperative CB-PPH....................15
Supplementary Figures 16
Figure S1. Interpretation of DI and RAND/UCLA Appropriateness scale....................16
Figure S2. PRISMA Flowchart 17
Figure S3. Second round ranking of one-step thresholds to initiate treatment for intraoperative and postoperative CB PPH....................18
Supplementary Files 19
Supplementary File S1. Search strategy 19
Supplementary File S2. In-person meeting agenda 20
Supplementary File S3. In-person meeting discussion question guide 23
Supplementary File S4. List of contributors....................28

For peer review only

Supplementary Tables

Table S1. Definitions of PPH and severe PPH used in clinical guidelines

Definitions	Number of guidelines using the definition	Specific guidelines using the definition	Specifications regarding mode of birth and timeframe in which bleeding occurs	Additional considerations
**DEFINITIONS OF PPH**				
Blood loss at least 500 mL	6	(1, 2, 3, 4, 5, 6)	Explicitly specified that this definition applied regardless of the mode of birth: (3, 6)<br>Indicated that blood loss must occur within 24 h of birth: (1, 2, 6)	Two guidelines targeting high-income countries (HICs) specified that for caesarean birth the threshold could be set at a higher blood loss if clinically tolerated: (3, 4)
Blood loss at least 1000 mL	2	(7, 8)	Definition applied for caesarean only: all<br>No timeframe indicated: all	---
Blood loss at least 1000 mL OR signs of haemodynamic instability	5	(9, 10, 11, 12, 13)	Definition applied for caesarean only: (9, 10)<br>Indicated that blood loss must occur within 24 h of birth: all	One guideline recommended that cumulative blood loss of 500-999 mL alone should trigger increased supervision and potential interventions as clinically indicated: (13)
Any bleeding that causes haemodynamic instability	1	(14)	Explicitly specified that definition was applicable regardless of mode of birth<br>Indicated that bleeding must occur within 24 h of birth	For clinical purposes, any blood loss that had the potential to produce hemodynamic instability should be considered PPH. The amount of blood loss required to cause hemodynamic instability would depend on pre-existing conditions (e.g., anaemia, dehydration, gestational hypertension with proteinuria)
**DEFINITIONS OF SEVERE PPH**				
Blood loss at least 1000 mL	4	(1, 2, 3, 5)	Explicitly specified that this definition applied regardless of the mode of birth: (3)<br>Indicated that bleeding must occur within 24 h of birth: (1, 2)	RCOG defined major PPH as blood loss greater than 1000 mL. Major PPH could be further subdivided into moderate (1001–2000 ml) and severe (more than 2000 ml).
Blood loss at least 1000 mL OR signs of haemodynamic instability	2	(6, 12)	Explicitly specified that this definition applied regardless of the mode of birth: (6)<br>Indicated that bleeding must occur within 24 h of birth: all	---

Note: No definition of PPH mentioned: (15, 16, 17). No definition of severe PPH mentioned (4, 8, 11, 13, 16, 17, 18)

For peer review only - http://bmjopen.bmj.com/site/about/guidelines.xhtml

Table S2. Synthesis of evidence and considerations regarding approaches to detect intraoperative and postoperative caesarean PPH and haemodynamic instability

Detection methods	Paired with any of the following blood collection devices	Sources discussing this method	Summary of the evidence
**BLOOD LOSS ASSESSMENT METHODS WITH SPECIFIC BLOOD COLLECTION DEVICES**			
Visual estimation of blood loss	• Suction canister<br>• Blood-soaked materials and clots<br>• Calibrated drape<br>• Non-calibrated blood loss collectors placed under the buttocks<br>• Non-calibrated blood loss collectors attached to the abdomen during surgery	4 Guidelines: (1, 8, 11, 14)<br><br>3 Systematic reviews: (19, 20, 21)<br><br>Additional relevant references: (22)	While four guidelines stated that visual estimation was used in practice, none actually recommended this method. Rather, they described its limitations. Visual estimation was described as subjective, imprecise, and known to underestimate actual blood loss (5, 17). Clinicians were advised to be aware that visual estimation of peripartum blood loss is inaccurate (2). The systematic reviews noted that some trialists used visual estimation to determine amount of blood lost and detect PPH. No further comment was provided. Although widely used for the detection of PPH, visual estimation of blood loss was consistently reported as inaccurate (22). While both underestimation and overestimation occur, the extent of underestimation increased as the volume of blood loss increased (17, 22).
Volumetric	• Calibrated suction canister<br>• Calibrated drape	6 Guidelines: (2, 3, 4, 6, 13, 17)<br><br>3 Systematic reviews: (19, 20, 21)<br><br>Additional relevant references: (23, 24, 25, 26, 27)	Guidelines described the use of volumetric methods. Some guidelines proposed using a combination of volumetric and gravimetric methods for assessing blood loss (3, 17). Some guidelines noted that while quantitative methods are more accurate than visual estimation in determining maternal blood loss, their effect on clinical outcomes has not been demonstrated (2, 17). The systematic reviews noted that some trialists used this method to determine amount of blood lost and detect PPH. Some trialists used a combination of gravimetric and volumetric methods. No further comment was provided. Volumetric techniques appeared to be more accurate than the visual estimation of blood loss, irrespective of provider experience, level of training, or specialty (23, 24). Mean measured blood loss was found to be 30% more accurate than estimated blood loss in vaginal births(25). The discrepancy between volumetric methods and visual estimation was found to be higher with increasing blood volume (26). The effectiveness of volumetric methods on clinical outcomes has not been demonstrated (17). A large multicentre multi-country cluster randomized trial comparing calibrated drapes vs. visual estimation failed to show a reduction in severe PPH (27)
Gravimetric	• Non-calibrated blood loss collectors placed under the buttocks<br>• Non-calibrated blood loss collectors attached to the abdomen during surgery<br>• Blood-soaked materials and clots: either intra- or postoperative	8 Guidelines: (2, 3, 4, 5, 6, 12, 13, 17)<br><br>2 Systematic reviews: (19, 20)<br><br>Additional relevant references: (28, 29)	Guidelines described the use of gravimetric methods. Some guidelines proposed using a combination of volumetric and gravimetric methods for assessing blood loss (3, 12, 17). Some guidelines noted that while quantitative methods are more accurate than visual estimation in determining maternal blood loss, their effect on clinical outcomes has not been demonstrated (2, 12, 17). Two guidelines stated that weighing of swabs *may* be used, but did not directly suggest their use (2, 4). The systematic reviews noted that some trialists used this method to determine amount of blood lost and detect PPH. Some trialists used a combination of gravimetric and volumetric methods. No further comment was provided. A 2014 randomized controlled trial including nine hundred women presenting for vaginal delivery found that blood loss recorded using a non-calibrated collector followed by gravimetric assessment was lower than blood loss recorded using the calibrated drape for

For peer review only - http://bmjopen.bmj.com/site/about/guidelines.xhtml

Detection methods	Paired with any of the following blood collection devices	Sources discussing this method	Summary of the evidence
			blood collection followed by volumetric assessment, with a mean difference in recorded blood loss of 58.6ml (28). One study of 228 women with PPH following vaginal delivery found weighing blood loss compared to Hgb drop (of 10%) had a sensitivity of <75% and a specificity of 97%). These findings were modelled at hypothetical high prevalence PPH settings (15%, 30%), where the Positive Predictive Value was >86% (29).
**METHODS OF DETECTING HEMODYNAMIC INSTABILITY SECONDARY TO PPH DURING AND AFTER CAESAREAN BIRTH**			
Clinical signs of haemodynamic instability	• N/A	6 Guidelines: (2, 3, 5, 10, 12, 14) 3 Systematic reviews: (19, 20, 21) Additional relevant references: (30, 31, 32, 33, 34, 35, 36)	Signs of haemodynamic instability reported in guidelines included changes in blood pressure, heart rate, pulse oximetry, urine output, or general status (faintness/dizziness, nausea, thirst, altered level of consciousness, pallor, sweating, poor capillary refill, and cold extremities. The guidelines proposed considering clinical signs and symptoms of haemodynamic instability in combination with other methods (such as volumetric and gravimetric methods for blood loss assessment) for the detection of PPH.(2, 3, 5, 10, 12, 14). Some of these guidelines presented tables correlating clinical signs with blood loss and the degree of shock. However, most of them warned that many clinical signs and symptoms do not occur until the blood loss reaches very high levels due to the physiological increase in circulating blood volume during pregnancy. Some other guidelines (6, 12, 13, 14) explicitly proposed including clinical signs and symptoms of haemodynamic instability or the use of the shock index specifically in assessing PPH severity. None of the trials included in the systematic reviews used this method to detect PPH. The Shock Index (SI) as a predictor of several maternal outcomes has been evaluated in the context of PPH research, including both vaginal and caesarean birth. SI performance was usually reported using the Area under the Curve (AUC) parameter (with estimates between 0.7 - 0.8 in most studies). According to the cut-off value of the SI chosen and the specific outcome analysed, SI sensitivity may range from 30% to 90%(30, 31, 32, 33, 34, 35). A recent stepped-wedge cluster randomized trial showed insufficient evidence to suggest that a significant benefit or harm could be attributed using an automated SI device (in low-resource settings) on a composite outcome of maternal deaths, eclampsia, or emergency (36). However, the rate of emergency hysterectomy was significantly reduced.
Visual charts and early warning scores (EWS)	• N/A	5 Guidelines: (2, 4, 6, 12, 13) 0 Systematic reviews: None reference this method. Additional relevant references: (37)	A few guidelines recommended visual charts and early warning scores to alert caregivers to abnormal trends in haemodynamic measurements. However, these seem to be recommended for follow-up monitoring of diagnosed PPH cases rather than for diagnosis of PPH. None of the trials included in the systematic reviews used this method to detect PPH. A systematic literature review of 17 published obstetric EWS reported that they had very high median sensitivity (89%) and specificity (85%) but low median positive predictive values (41%) for predicting morbidity or ICU admission. Obstetric EWS had high accuracy in predicting death (AUROC >0.80) among critically ill obstetric women (37).

For peer review only - http://bmjopen.bmj.com/site/about/guidelines.xhtml

Table S3. Synthesis of evidence on thresholds for triggering action on PPH during and after caesarean birth

Thresholds	Guidelines recommending this threshold	Applicable mode of birth (if thresholds differ by mode of birth)	Comments
**ONE-STEP APPROACH: THRESHOLDS TRIGGER FULL RESPONSE PROTOCOL**			
Blood loss at least 1000 mL OR signs of haemodynamic instability	1. (10)<br>2. (11)<br>3. (12)<br>4. (9)	Three guidelines proposed this threshold as being specific for caesarean birth(9, 10, 12).	In these guidelines the same criteria proposed as the definition of PPH were also used as the threshold for initiating treatment.
Blood loss at least 500 mL	1. (6)	This guideline stated that this threshold applied regardless of the mode of birth.	The guideline noted that clinical signs and symptoms of hypovolaemia should be included in the assessment of PPH severity. However, clinical signs of hypovolaemia are misleading in pregnancy due to plasma volume expansion and might not become evident until blood losses reach 1,000-1,500 mL in healthy women. Thus, the blood loss thresholds should depend on the woman's clinical condition and local resources. In this guideline the same criteria proposed for the definition of PPH were also used as the threshold for initiating treatment.
Blood loss at least 500 mL OR Signs of haemodynamic instability	1. (3)	This guideline stated that this threshold applied regardless of the mode of birth.	This guideline noted that the bleeding rate, PPH etiology, and clinical context should be considered. Further, for caesarean births, thresholds could be set at a higher blood loss if clinically tolerated. In this guideline the same criteria proposed for the definition of PPH were also used as the threshold for initiating treatment.
Blood loss at least 1000 mL	1. (7)	This guideline stated that this threshold is specific for caesarean birth.	In this guideline the same criteria proposed for the definition of PPH were also used as the threshold for initiating treatment.
Any excessive bleeding with signs of hemodynamic instability	1.	This guideline stated that this threshold is applicable to all births.	In this guideline the same criteria proposed for the definition of PPH were also used as the threshold for initiating treatment.
**TWO/STEP APPROACH: THRESHOLDS TRIGGER DIFFERENTIAL ACTIONS**			
Lower threshold: Blood loss at least ***500 mL*** *without clinical shock; Higher threshold: Blood loss at least* ***1000 ml,*** *continued bleeding, OR clinical shock*	1. (2)<br>2. (4)<br>3. (5)<br>4. (13)	Guidelines did not specify a difference in threshold by mode of birth.	According to these guidelines, blood loss of **500–1000 ml** (minor PPH) without clinical shock should trigger: close monitoring, laboratory tests, and the use of crystalloid infusion [(2)] ; prompt basic measures (close monitoring, intravenous access, full blood count, group, and screen, insert urinary catheter) to facilitate resuscitation [(4)] ; enhanced surveillance and early interventions as needed[(13)] . Blood loss ≥ **1000 ml** and continued bleeding or clinical shock should trigger a full protocol to achieve resuscitation and haemostasis. [(2, 4, 13)]Three guidelines aligned their proposed lower thresholds with their proposed PPH definitions. [(2, 4, 5)] A single guideline aligned its proposed higher threshold with its PPH definition. [(13)]

For peer review only - http://bmjopen.bmj.com/site/about/guidelines.xhtml

Thresholds	Guidelines recommending this threshold	Applicable mode of birth (if thresholds differ by mode of birth)	Comments
Lower threshold: Blood loss at least 1000 mL; Higher threshold: Blood loss of at least 2000 mL OR SI of ≥1.0	1. (8)	Guideline provided thresholds specific for CB	According to this guideline, blood loss of **1000 mL** should trigger suspicion of PPH and initiation of treatment. Blood loss of *at least* **2000 mL or SI of ≥1.0** should trigger: initiation of IV catheter with a large gauge and replacement of a sufficient volume of fluid; consideration of blood transfusion and the transportation of the patient to a secondary or tertiary hospital; monitoring of blood pressure, pulse rate, bleeding amount, urine output and SpO2. This guideline used the same criteria as thresholds for action as were used for proposed PPH definition.

*Four guidelines (1, 15, 16, 17)did not mention any thresholds for initial assessment or to trigger a full protocol. Note CB= caesarean birth

For peer review only

Table S4. Medical Interventions, manoeuvres, and procedures for PPH recommended by WHO compared to recommendations in other PPH guidelines and systematic reviews

Method	WHO recommendation (PPH 2012,TXA 2017, Carbetocin 2018, and UBT 2021) (1, 9, 38, 39)	Other Reviewed Guidelines	Systematic Reviews
**MEDICAL INTERVENTIONS**			
**Uterotonics**			
Oxytocin	Intravenous oxytocin was the recommended first-line treatment for PPH, including among those who have already received oxytocin for prevention of PPH (no dosing information included; 10 IU IM or IV was the recommended dosing and route of administration provided for prophylactic use).	Recommended as first line drug ((2, 3, 4, 5, 6, 7, 10, 12, 13, 14, 17)<br>Two different dosing regimens discussed:<br>• Intravenous oxytocin 5 IU (slow IV injection over 2 minutes). May repeat dose once.<br>• Intravenous infusion 5-10 IU per hour (20-40 IU in 500 ml saline over 4 hours).	Not described.
Carbetocin	Not described in the 2012 WHO treatment guidelines. The 2018 WHO prevention guidelines recommended prophylactic carbetocin (100 µg, IM/IV) for all births when cost was comparable to other effective uterotonics, but did not recommend the use of carbetocin as a treatment for PPH.	Recommended as a first line drug (7, 14)or as a second-line drug (10) for treatment.	Not described.
Ergometrine	Recommended if IV oxytocin unavailable, or bleeding nonresponsive to oxytocin. No dosing amount provided. Intravenous route of administration recommended.	Intravenous or intramuscular routes recommended. Maximum dose of 1000 mcg. 250-500 mcg (IV slow, over 2 minutes, or IM), may repeat every 5 minutes (2, 4, 5, 12)	Ergometrine 200 mcg administered intramuscularly, followed by 250 mcg IM carboprost if needed (21)
Oxytocin-ergometrine fixed dose	Recommended if IV oxytocin unavailable, or bleeding nonresponsive to oxytocin. No dosing amount or route of administration recommended. `	Not described.	Syntometrine® (ergometrine 500 mcg plus oxytocin 5 IU) administered intramuscularly plus oxytocin 10 IU administered by an intravenous infusion (21)

For peer review only - http://bmjopen.bmj.com/site/about/guidelines.xhtml

Method	WHO recommendation (PPH 2012,TXA 2017, Carbetocin 2018, and UBT 2021) (1, 9, 38, 39)	Other Reviewed Guidelines	Systematic Reviews
Prostaglandin (including sublingual misoprostol, 800 mcg)	Recommended if IV oxytocin unavailable, or bleeding nonresponsive to oxytocin. No dosing amount or route of administration information provided other than for misoprostol (recommended 800 mcg, administered sublingually). Sublingual misoprostol particularly recommended in settings where IV oxytocin unavailable and IM oxytocin used for prophylaxis.	Carboprost was recommended either: as a general second line drug (12), specifically for use after oxytocin/ergometrine (2), or recommended generally with no specific order cited (5). Most reviewed guidelines recommended a dose of 250 mcg IM, which could be repeated every 15 minutes, up to a maximum dose of 2000 mcg (2, 3, 4, 5, 6, 10, 11, 12, 13, 14). An alternative dosing regimen of 500 mcg intramyometrial route was cited in 6 guidelines (4, 5, 11, 12, 13, 14)Misoprostol was recommended either: as first line drug (5), when other first-line drugs unavailable or contraindicated (12), after oxytocin/ergometrine (2), or if carbroprost contraindicated (4). The recommended dosing regimen was a single dose of 600-1000 mcg, by oral, sublingual, or rectal route (2, 3, 4, 5, 6, 7, 10, 11, 12, 13, 14). Sulprostone IV route, 500 mcg administered over 1 hour (3, 6, 7)	Carboprost 250 mcg administered intramuscularly, followed by 250 mcg IM ergometrine if needed – considered a second line drug (21). Misoprostol 800 mcg (4 tablets of 200 mcg) administered rectally. Considered a first line drug. Data drawn from seven trials, only one of which included some women with caesarean birth (21)
Tranexamic acid with standard care	The 2017 WHO guidelines on tranexamic acid recommended a fixed dose of 1 g (100 mg/mL) administered intravenously at 1 mL per minute (i.e., administered over 10 minutes), with a second 1 g IV dose if bleeding continued after 30 minutes OR restarted within 24 hours of completing the first dose.	Intravenous administration of 1 g over 10 minutes. A second dose may be administered after 30 minutes if bleeding persists (2, 3, 4, 5, 6, 7, 11, 12)	A fixed dose of 1g (100 mg/mL) intravenously at 1 ml per minute, within 3 hours of the time of diagnosis (if unknown, time of birth); a second dose of 1g given if needed 30 minutes from the first dose. Considered a first-line treatment (40).
**MANOEUVRES AND OTHER PROCEDURES**			
**Mechanical interventions**			
Uterine massage	Rubbing of the uterus achieved through manual massage of the abdomen, typically sustained until bleeding ceases or the uterus contracts. (initial rubbing of uterus and expression of clots NOT considered therapeutic uterine massage).<br>Recommended (low cost and relative safety of uterine massage considered in this recommendation).<br>Note: This recommendation was developed considering vaginal birth.	Intervention recommended in other 10 guidelines (2, 3, 4, 5, 7, 10, 11, 12, 14)	Not described.

For peer review only - http://bmjopen.bmj.com/site/about/guidelines.xhtml

Method	WHO recommendation (PPH 2012,TXA 2017, Carbetocin 2018, and UBT 2021) (1, 9, 38, 39)	Other Reviewed Guidelines	Systematic Reviews
Intrauterine balloon tamponade	The procedure entails insertion of a deflated/uninflated balloon into the uterine cavity and then inflating it to achieve a tamponade effect. Uterine balloon tamponade was recommended for the treatment of postpartum haemorrhage due to uterine atony after vaginal birth in women who did not respond to standard first-line treatment, provided all required resources for of PPH are available and routinely implemented (39). Only two studies included in the evidence supporting the recommendations included caesarean deliveries, both of which evaluated the effect of UBT in cases of placenta praevia or traumatic bleeding. One study (41)suggests that the use of the Bakri balloon could be more effective than haemostatic sutures in, and the second (42)suggests benefits associated to the use of the Bakri balloon held in place with a traction stitch versus Bakri balloon without traction stitch.	Guidelines described the urological Rusch balloon (left over 4-6 hrs), the Bakri SOS tamponade balloon catheter, the Sengstaken-Blakemore esophageal catheter, the Foley catheter, the polyurethane Ebb double balloon (vaginal and uterine), and the silicone BT-Cath tamponade balloon (2, 4, 6, 7, 10, 11, 13, 14)	One trial (50 women) compared Bakri Balloon with and without traction stitch; another trial (13 women) compared Bakri balloon to compressive suturing to the lower segment of the uterus. Not specified whether a first-line, second-line, or temporising treatment (20)
Uterine packing	Not recommended for PPH due to uterine atony.	One guideline (13) did not recommend the use of uterine packing while two guidelines recommended its use as a temporizing measure (14) or for unresponsive PPH (11).	Not described.
**Uterine-sparing surgical interventions and procedures**			
Compressive sutures	Compression suturing that runs through the full thickness of both uterine walls. When tied, the suture allows tight compression of the uterine walls and stops the bleeding. No specific suturing technique (e.g., B-Lynch, Hayman, Pereira). Recommended as first-line surgical intervention.	The B-Lynch technique was the most common uterine compression technique for atony (2, 3, 4, 5, 7, 10, 11, 12, 13, 14); however, other techniques, such as Cho and Hayman, were also recommended and described (10, 11).	One trial (160 women) compared the standard B-Lynch suture to a modified B-Lynch suture. Considered a second-line treatment (20).
Devascularisation / Artery ligation	Vascular flow to the uterus can be interrupted by uterine devascularization, ligation of the uterine or internal iliac arteries Recommended only if all available conservative measures (uterotonics, uterine massage, balloon tamponade) have failed.	If compression sutures are unsuccessful, bilateral uterine artery ligation, bilateral utero-ovarian artery ligation or -If expertise available- bilateral internal iliac artery ligation must be considered (2, 3, 4, 5, 6, 7, 10, 11, 12, 14). A common first approach is bilateral uterine artery ligation (O'Leary sutures) (11).	One trial (23 women) compared uterine artery embolization to surgical devascularization plus B-Lynch compression sutures. Not specified wither first-response, second-line, or temporizing treatment (20).

For peer review only - http://bmjopen.bmj.com/site/about/guidelines.xhtml

Method	WHO recommendation (PPH 2012,TXA 2017, Carbetocin 2018, and UBT 2021) (1, 9, 38, 39)	Other Reviewed Guidelines	Systematic Reviews
Uterine artery embolisation (UAE)	If other measures have failed and if the necessary resources were available, the use of uterine artery embolization was recommended as a treatment for PPH due to uterine atony.	Twelve guidelines recommended UAE (1, 2, 3, 4, 5, 6, 7, 10, 11, 12, 13, 14).<br>Only one describes the intervention in greater detail(3)	One trial (23 women) compared uterine artery embolization to surgical devascularization plus B-Lynch compression sutures. Not specified wither first-line, second-line, or temporizing treatment (20).
**Temporising**			
External aortic compression	Recommended as a temporizing measure until appropriate care is available, in PPH due to uterine atony.	In addition to WHO guidelines, seven guidelines recommended external aortic compression (Queensland, (2, 4, 6, 7, 10, 12, 14)	Not described in Systematic Reviews.
Non-pneumatic anti-shock garment	Recommended as a temporizing measure until appropriate care is available.	Recommended in other three guidelines (6, 10, 13).	One systematic review did not find a reduction in maternal mortality associated to NASG in the one cluster-RCT (880 women) included, However, 5 comparative studies (pre-intervention to intervention) included in this review (2330 women) suggested a clinically important reduction in maternal mortality and severe maternal morbidity(43). No effect was observed on the use of blood products. There were no safety issues in all the trials (43).

*Pileggi-Castro 2015 (43) is a systematic review on the non-pneumatic anti-shock garment as a treatment for severe PPH. This review was not captured as part of the overview review because the word “cesarean”/”caesarean” did not appear in the text. After consultation with Prof. Suellen Miller, who participated in each of the primary studies, confirmed that women with caesarean section were included in the original trials, data from this systematic review was added to the report and is reflected in the following tables.

For peer review only

For peer review only - http://bmjopen.bmj.com/site/about/guidelines.xhtml

Table S5. First and second round ratings and agreement for early detection methods for intraoperative and postoperative CB-PPH

Blood loss measurement and other PPH detection methods	How would you rate each of the methods below for early detection of PPH considering…							
	**the usefulness in managing patients?** 1=Not at all useful; 9=extremely useful	**feasibility in all settings performing CB?** 1=Hardly feasible; 9=Highly feasible	**its acceptability to key stakeholders?** 1=Not at all useful; 9=extremely useful	**the resources required?*** 1= very small; 9=very large	**the usefulness in managing patients?** 1=Not at all useful; 9=extremely useful	**feasibility in all settings performing CB?** 1=Hardly feasible; 9=Highly feasible	**its acceptability to key stakeholders?** 1=Not at all useful; 9=extremely useful	**the resources required?*** 1= very small; 9=very large
	**FIRST ROUND** Median (DI)				**SECOND ROUND** Median (DI)			
**INTRAOPERATIVE**								
Volumetric/gravimetric + clinical signs of haemodynamic instability	**8.0 (-0.93)**	6.0 (2.35)	NA	NA	NA	**8.0 (-3.08)**	NA	NA
Volumetric	**8.0 (-0.71)**	6.5 (3.50)	7.0 (2.35)	6.5 (1.85)	NA	6.5 (10.00)	7.0 (10.0)	7.0 (2.35)
Clinical signs of haemodynamic instability	6.5 (8.31)	**8.0 (-1.94)**	**8.0 (-0.22)**	**3.5 (0.58)**	**7.0 (-0.71)**	NA	NA	NA
Volumetric + gravimetric	**7.0 (-1.26)**	**5.0 (0.94)**	NA	NA	NA	NA	NA	NA
Clinical judgement such as rate of flow and duration	**5.0 (0.92)**	**7.0 (-21.7)**	6.0 (2.35)	**2.0 (0.49)**	NA	NA	7.0 (10.0)	NA
Visual charts and early warning scores (EWS)	**5.0 (0.88)**	6.0 (2.09)	6.0 (1.96)	5.0 (1.70)	NA	7.0 (10.0)	6.5 (10.0)	6.0 (1.74)
Visual estimation of blood loss	**4.5 (0.97)**	**8.5 (-1.81)**	7.0 (30.0)	**1.0 (0.13)**	NA	NA	**7.0 (-1.94)**	NA
Gravimetric	6.0 (2.35)	**4.5 (0.91)**	**5.0 (0.85)**	6.0 (2.09)	7.0 (10.00)	NA	NA	7.0 (10.00)
Visual estimation + visual charts/EWS	6.0 (1.48)	**7.0 (-0.71)**	NA	NA	6.5 (2.35)	NA	NA	NA
**POSTOPERATIVE**								
Clinical signs of haemodynamic instability	**8.0 (-3.08)**	**8.0 (-1.27)**	**8.0 (-1.94)**	**3.5 (0.58)**	NA	NA	NA	NA
Volumetric/gravimetric + clinical signs of haemodynamic instability	**7.0 (-0.93)**	6.0 (1.74)	NA	NA	NA	7.0 (30.00)	NA	NA
Clinical judgement such as rate of flow and duration	**5.0 (0.41)**	6.0 (2.35)	6.0 (2.35)	**2.0 (0.49)**	NA	6.0 (2.35)	7.0 (10.00)	NA
Gravimetric	5.5 (2.35)	**3.0 (0.65)**	**5.0 (0.85)**	6.0 (2.09)	**5.0 (0.63)**	NA	NA	7.0 (10.00)
Volumetric	5.0 (2.09)	**4.5 (0.58)**	7.0 (2.35)	6.5 (1.85)	**5.0 (0.63)**	NA	7.0 (10.00)	7.0 (2.35)
Visual estimation of blood loss	**4.0 (0.52)**	**7.0 (-3.08)**	7.0 (30.0)	**1.0 (0.13)**	NA	NA	**7.0 (-1.94)**	NA
Visual charts and early warning scores (EWS)	6.0 (2.09)	7.0 (2.35)	6.0 (1.96)	5.0 (1.70)	7.0 (10.0)	7.0 (10.00)	6.5 (10.00)	6.0 (1.74)
Visual estimation + visual charts/EWS	6.0 (4.96)	**7.0 (-3.08)**	NA	NA	7.0 (10.0)	NA	NA	NA
Volumetric + gravimetric	7.0 (8.31)	4.0 (1.04)	NA	NA	7.0 (10.0)	**5.0 (0.85)**	NA	NA

For peer review only - http://bmjopen.bmj.com/site/about/guidelines.xhtml

For peer review only

Note: A DI < 1 represented agreement, while a DI ≥ 1 indicated disagreement. Results in which agreement is reached are highlighted in bold.NA= Not applicable given that this combination of methods was not rated in the first round for acceptability to key stakeholders and the estimate of resources required, or because agreement was obtained in the first round. * The measurement scale for this criterion is the same as for the other criteria (from 1 to 9). However, unlike the other criteria, low values have a positive interpretation (few resources required) while high values have a negative interpretation (substantial resources required).

For peer review only

For peer review only - http://bmjopen.bmj.com/site/about/guidelines.xhtml

Table S6. First round ratings and agreement on threshold to initiate treatment for intraoperative CB PPH

Thresholds for triggering action	How would you rate each of the following thresholds for managing PPH during CB considering…		
	**the accuracy of each threshold?** 1=Hardly accurate; 9=Highly accurate	**its feasibility to be used in all settings?** 1=Hardly feasible; 9=Highly feasible	**its acceptability to key stakeholders?** 1=Hardly accepted; 9=Highly accepted
**INTRAOPERATIVE**			
**One-step approach (trigger full response protocol)**			
At least 1000 mL blood loss OR signs of haemodynamic instability, whichever comes first	**9 (-0.34)**	**8 (-0.34)**	**8 (-0.54)**
At least 1000 ml (blood loss alone, regardless of signs of haemodynamic instability)	**7 (-0.71)**	**8 (-0.71)**	**7 (-2.30)**
Haemodynamic instability alone, regardless of volume of blood loss	**7 (-3.08)**	**7 (-0.71)**	**7 (-3.08)**
At least 500 mL blood loss OR signs of haemodynamic instability, whichever comes first	7 (2.30)	**7 (-4.00)**	6.5 (8.31)
At least 500 mL (blood loss alone, regardless of signs of haemodynamic instability)	**5 (0.41)**	6.5 (2.14)	**5 (0.88)**
**Two-step approach (Lower threshold triggers further assessment, preparedness, and close monitoring; Higher threshold triggers treatment initiation)**			
Lower threshold of blood loss at least 500 mL (blood loss alone, regardless of signs of haemodynamic instability), and higher threshold of blood loss at least 1000 mL blood loss OR signs of haemodynamic instability, whichever comes first	**8 (-0.71)**	**8 (-0.71)**	**7 (-0.71)**
Lower threshold of blood loss at least 1000 ml (blood loss alone, regardless of signs of haemodynamic instability), and higher threshold of blood loss at least 2000 mL blood loss OR signs of haemodynamic instability whichever comes first	6.5 (4.96)	**8 (-4.23)**	7 (30.00)
**POSTOPERATIVE**			
**One-step approach (trigger full response protocol)**			
At least 1000 mL blood loss OR signs of haemodynamic instability, whichever comes first	**8.5 (-0.34)**	**8 (-2.14)**	**8 (-0.88)**
At least 1000 ml (blood loss alone, regardless of signs of haemodynamic instability)	**7 (-0.71)**	**8 (-0.71)**	**7 (-1.27)**
Haemodynamic instability alone, regardless of volume of blood loss	**7 (-3.08)**	**7 (-4.00)**	7 (10.00)
At least 500 mL blood loss OR signs of haemodynamic instability, whichever comes first	7 (8.31)	**7 (-16.8)**	6.5 (30.00)
At least 500 mL (blood loss alone, regardless of signs of haemodynamic instability)	5 (1.47)	6 (2.09)	5 (1.18)
**Two-step approach (Lower threshold triggers further assessment, preparedness, and close monitoring; Higher threshold triggers treatment initiation)**			
Lower threshold of blood loss at least 500 mL (blood loss alone, regardless of signs of haemodynamic instability), and higher threshold of blood loss at least 1000 mL blood loss OR signs of haemodynamic instability, whichever comes first	**8 (-0.71)**	**8 (-0.71)**	**7.5 (-0.71)**

Lower threshold of blood loss at least 1000 ml (blood loss alone, regardless of signs of haemodynamic instability), and higher threshold of blood loss at least 2000 mL blood loss OR signs of haemodynamic instability whichever comes first	6.5 (4.41)	6 (2.35)	6 (1.96)

Note: A DI < 1 represented agreement, while a DI ≥ 1 indicated disagreement. Results in which agreement is reached are highlighted in bold. CB= caesarean birth.

For peer review only

For peer review only - http://bmjopen.bmj.com/site/about/guidelines.xhtml

Table S7. First round ratings and agreement for first response interventions for managing intraoperative and postoperative CB-PPH

First response interventions for managing PPH during and after CB	**How would you rate each intervention for first response management of CB-PPH considering…**				
	**the balance of effects?** 1=Weighted towards undesirable effects; 9=Weighted towards desirable effects	**the resources required? *** 1= very few; 9=very many	**its feasibility?** 1=Hardly feasible; 9=Highly feasible	**its acceptability to stakeholders?** 1=Hardly accepted; 9=Highly accepted	**equity?** 1=Likely to exacerbate inequities; 9=Highly likely to reduce inequities
	Median				
INTRAOPERATIVE					
Oxytocin	**9 (-0.34)**	3 (5.86)	**9 (-0.34)**	**9 (0.00)**	**9 (0.00)**
Carbetocin	**8 (-0.93)**	5 (1.81)	**7.5 (-3.79)**	**8 (-0.65)**	6 (6.55)
TXA	**8 (-0.34)**	**4 (0.76)**	**7 (-4.00)**	**8 (-0.93)**	**8 (-3.79)**
Compressive sutures	7 (2.9)	7 (4.64)	6 (4.71)	5.5 (1.76)	6 (4.71)
Bimanual compression	7 (10.15)	**3 (0.64)**	**7.5 (-3.08)**	6.5 (12.8)	**8 (-2.56)**
Uterine massage	**7 (-15.2)**	2 (1.17)	**8.5 (-0.65)**	**8 (-0.93)**	8 (-0.34)
Oxytocin-ergometrine fixed dose	6 (2.35)	4 (1.61)	**7 (-3.08)**	**8 (-3.08)**	**8 (-2.19)**
Prostaglandin (including sublingual misoprostol)	6 (1.37)	**3 (0.87)**	**8 (-3.79)**	**8 (-2.14)**	**8 (-0.93)**
Ergometrine	6 (4.00)	**3 (0.91)**	**8 (-0.92)**	**7 (-0.92)**	**8 (-0.34)**
Non-pneumatic anti-shock garment	5 (1.04)	**6 (0.78)**	**4 (0.52)**	5 (1.08)	5 (1.00)
External aortic compression	**5 (0.56)**	**2 (0.75)**	6 (30.00)	**5 (0.78)**	7 (30.00)
Intrauterine balloon tamponade	4 (1.61)	5 (2.14)	**4 (0.91)**	**5 (0.85)**	6 (4.22)
POSTOPERATIVE					
Oxytocin	**9 (-0.34)**	3 (5.86)	**9 (-0.34)**	**9 (0.00)**	**9 (0.00)**
TXA	**8 (-0.34)**	**4 (0.76)**	**7 (-13.00)**	**8 (-0.93)**	**8 (-6.78)**
Non-pneumatic anti-shock garment	6 (1.35)	6 (1.73)	**4 (0.52)**	**5 (0.99)**	5 (1.00)
Carbetocin	**6 (-10.29)**	5 (1.81)	**7.5 (-8.76)**	**8 (-0.92)**	5.5 (4.22)
Oxytocin-ergometrine fixed dose	6 (2.05)	4 (1.61)	**7 (-3.08)**	**8 (-3.08)**	**8 (-1.54)**
Uterine massage	6 (2.35)	3 (1.17)	**8 (-0.93)**	**8 (-1.68)**	**8 (-3.79)**
Ergometrine	6 (4.00)	**3 (0.97)**	**8 (-0.71)**	**7.5 (-2.19)**	**8 (-0.34)**
Prostaglandin (including sublingual misoprostol)	**6 (0.89)**	3 (1.35)	**8 (-1.53)**	**7.5 (-2.14)**	**8 (-0.93)**
Bimanual compression	**5 (0.63)**	**3 (0.41)**	6 (16.57)	6 (1.73)	**8 (-23.00)**
External aortic compression	**5 (0.68)**	**2 (0.68)**	6 (2.25)	**5.5 (0.52)**	7 (5.6)
Intrauterine balloon tamponade	4 (1.64)	5 (2.14)	**4.5 (0.91)**	**5 (0.85)**	4.5 (4.22)
Compressive sutures	**2 (0.89)**	6 (2.84)	**4 (0.99)**	**3 (0.91)**	4.5 (1.59)

Note: A DI < 1 represented agreement, while a DI ≥ 1 indicated disagreement. Results in which agreement is reached are highlighted in bold. CB= caesarean birth. * The measurement scale for this criterion is the same as for the other criteria (from 1 to 9). However, unlike the other criteria, low values have a positive interpretation (few resources required) while high values have a negative interpretation (substantial resources required)

Table S8. Second round ratings and agreement for first response interventions for managing intraoperative and postoperative CB-PPH

**Intraoperative**	Median (RAND DI)
Examine and rapidly initiate cause-specific first response (e.g., if trauma: rapid surgical haemostasis; if atony/placental cause: uterotonics and uterine massage)	**9 (-0.34)**
TXA for all women with PPH during CB regardless of aetiology	**8 (-0.43)**
Plasma expansion with crystalloids or all women with PPH during CB regardless of aetiology	**7.5 (-1.94)**
Uterotonics for all women with PPH during CB regardless of aetiology	**7 (-0.71)**
**Postoperative**	
Examine and rapidly initiate cause-specific first response (e.g., if trauma: rapid surgical haemostasis; if atony/placental cause: uterotonics and uterine massage)	**9 (-0.34)**
TXA for all women with PPH during CB regardless of aetiology	**8 (0.00)**
Plasma expansion with crystalloids or all women with PPH during CB regardless of aetiology	**7.5 (-0.71)**
Uterotonics for all women with PPH during CB regardless of aetiology	**7 (-0.71)**

Note: A DI < 1 represented agreement, while a DI ≥ 1 indicated disagreement. Results in which agreement is reached are highlighted in bold. CB= caesarean birth.

For peer review only - http://bmjopen.bmj.com/site/about/guidelines.xhtml

Supplementary Figures

Figure S1. Interpretation of DI and RAND/UCLA Appropriateness scale

<table>
<tr><td rowspan="3">DI (Disagreement index)</td><td colspan="9">Experts’ median rating</td></tr>
<tr><td>1</td><td>2</td><td>3</td><td>4</td><td>5</td><td>6</td><td>7</td><td>8</td><td>9</td></tr>
<tr><td colspan="3">Bottom third (1-3)</td><td colspan="3">Intermediate third (4-6)</td><td colspan="3">Top third (1-3)</td></tr>
<tr><td><1 (Agreement)</td><td colspan="3">Inappropriate</td><td colspan="3" rowspan="2">Uncertain</td><td colspan="3">Appropriate</td></tr>
<tr><td>≥1 (Disagreement)</td><td colspan="3"></td><td colspan="3"></td></tr>
</table>

For peer review only

Figure S2. PRISMA Flowchart

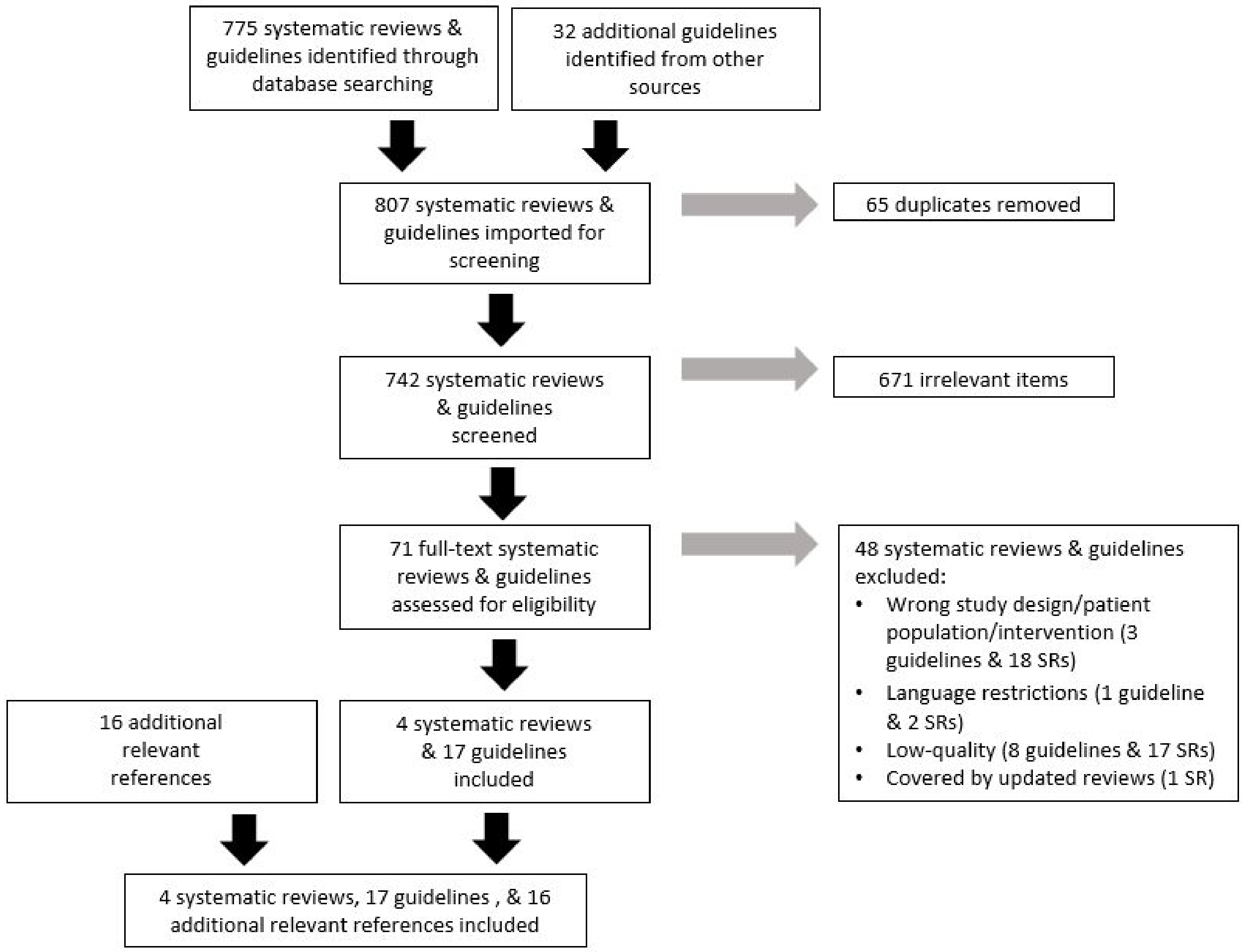

only

For peer review only - http://bmjopen.bmj.com/site/about/guidelines.xhtml

Figure S3. Second round ranking of one-step thresholds to initiate treatment for intraoperative and postoperative CB PPH

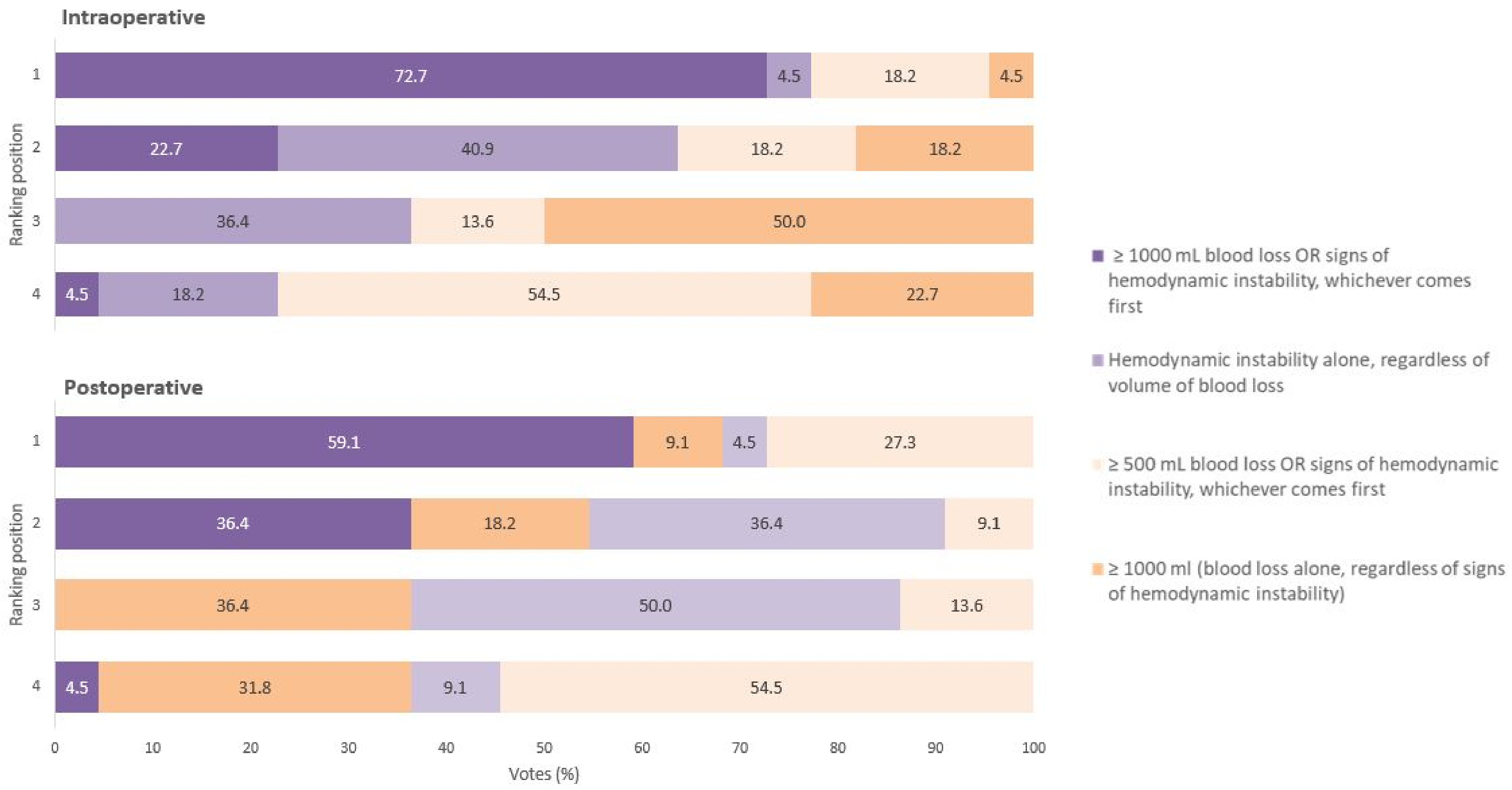

Note: CB= caesarean birth

Supplementary Files

Supplementary File S1. Search strategy

(Postpartum Hemorrhage[Mesh] OR Hemorrhag*[tiab] OR Haemorrhag*[tiab] OR PPH*[tiab] OR **Blood Specimen Collection[Mesh]** OR Blood Loss[tiab] OR “Loss of Blood”[tiab] OR Bleeding[tiab] OR Blood Specimen[tiab] OR Blood Collection[tiab]) AND **(Cesarean Section[Mesh]** OR C-Section*[tiab] OR Cesaerea*[tiab] OR Cesarea*[tiab] OR Cesaria*[tiab] OR Caesaria*[tiab] OR Caesarea*[tiab]) **AND (Systematic Review[sb] OR** Systematic Review[tiab] OR **Meta-Analysis[pt]** OR Meta-Analys*[tiab] OR **"Cochrane Database Syst Rev"[ta]** OR Metaanalysis[tiab] OR Metanalysis[tiab] OR Overview[ti] OR (Review[ti] AND Literature[ti]) OR (MEDLINE[tiab] AND Cochrane[tiab]) OR **Guideline[pt]** OR **Practice Guideline[pt]** OR Guideline*[ti] OR Guide Line*[tiab] OR Consensus[tiab] OR Recommendation*[ti])

(MH Postpartum Hemorrhage OR Hemorrhag$ OR Hemorragia OR PPH$) AND **(MH Cesarean Section** OR C-Section$ OR Cesaerea$ OR Cesarea$ OR Cesaria$ OR Caesaria$ OR Caesarea$)

For peer review only

For peer review only - http://bmjopen.bmj.com/site/about/guidelines.xhtml

Supplementary File S2. In-person meeting agenda

WHO Technical Consultation on early detection and first response to PPH during and after caesarean birth

Meeting Agenda
27-28 September 2022
Av. Appia 20, Geneva, Salle U2
Zoom: https://who.zoom.us/j/91683929578
(Meeting ID: 916 8392 9578; Password: &uqmG2dD)

Introduction

Thank you for your participation in the **WHO Technical Consultation on early detection and first response to PPH during and after caesarean birth**. This consultation is part of the ongoing EMOTIVE project (Early detection of Postpartum Haemorrhage and treatment using the WHO MOTIVE 'first response' bundle), coordinated by the University of Birmingham.

As a part of the EMOTIVE project, WHO and some EMOTIVE team members are conducting a three-stage modified Delphi process to generate consensus on the optimal approach for early detection and first-response treatment for postpartum haemorrhage occurring during (intraoperative) and after (postoperative) caesarean birth. The first two rounds of this consultation were informed by an overview of reviews of the literature and conducted via an anonymous online platform. This third, in-person round will serve to conclude the consultation.

Experts' Meeting Objectives

This meeting has two primary objectives:

To agree on an optimal CS-PPH detection strategy that would include blood loss measurement methods and thresholds for action during and after caesarean birth.

To develop a first response approach to manage PPH during and after caesarean birth. This objective includes both the selection of the strategy components and the identification of the optimal approach (bundle, algorithm, checklist, or a combination of them)

For peer review only - http://bmjopen.bmj.com/site/about/guidelines.xhtml

Organization of the Meeting

This meeting will be conducted in-person and via Zoom over the course of two (2) days. Please use this link to access the meeting virtually: https://who.zoom.us/j/91683929578 (Meeting ID: 916 8392 9578; Password: &uqmG2dD)

The first day of the meeting, we will present and discuss the results of the overview of reviews and first two rounds of the Delphi study. You will have the opportunity to ask questions regarding the findings. Later in the day, we will pose specific questions to the panel regarding issues raised by the results to-date. You will be asked to provide your opinion on each of these topics. Discussions will be focused first on the intraoperative period, and then the postoperative period. The objective is to present different points of view on methods for early detection of CS-PPH, thresholds for triggering action, and first-response treatments by the close of day 1.

The second day of the meeting, we will present proposed strategies for implementing early detection and first-response for CS-PPH in the intraoperative and postoperative periods. You will be asked to provide your opinions on the proposed strategies. Finally, you will be asked to rate the proposed strategies. The final results—including whether consensus was reached—and conclusion will be presented before the end of day 2.

Dates and time

Date: 27-28 September 2022	
Day 1	*Day 2*
Africa<br>• *Kenya — 10:00-18:05*<br>• *Nigeria — 08:00-16:05*<br>• *Republic of Congo —08:00-16:05*<br>• *South Africa — 09:00-17:05*	U. Africa<br>• *Kenya — 11:00-14:05*<br>• *Nigeria — 09:00-12:05*<br>• *Republic of Congo — 09:00-12:05*<br>• *South Africa — 10:00-13:05*
Americas<br>• *Argentina — 04:00-12:05*<br>• *Uruguay — 04:00-12:05*	Americas<br>• *Argentina — 05:00-08:05*<br>• *Uruguay — 05:00-08:05*
Europe<br>• *Denmark — 09:00-17:05*<br>• *France (Paris) — 09:00-17:05*<br>• *Switzerland — 09:00-17:05*<br>• *United Kingdom — 08:00-16:05*	Europe<br>• *Denmark — 09:00-12:05*<br>• *France — 10:00-13:05*<br>• *Switzerland — 10:00-13:05*<br>• *United Kingdom — 09:00-12:05*
South-East Asia<br>• *Australia (Melbourne) — 17:00-01:05*<br>• *Egypt — 09:00-17:05*<br>• *Vietnam — 14:00-22:05*	South-East Asia<br>• *Australia — 18:00-21:05*<br>• *Egypt — 10:00-13:05*<br>• *Vietnam — 15:00-18:05*
Western Pacific<br>• *India — 12:30-20:35*<br>• *Philippines —15:00-23:05*<br>• *Thailand — 14:00-22:05*	Western Pacific<br>• *India — 13:30-16:35*<br>• *Philippines — 16:00-19:05*<br>• *Thailand — 15:00-18:05*

For peer review only - http://bmjopen.bmj.com/site/about/guidelines.xhtml

AGENDA

**DAY 1: 27 September 2022**		**Presenter**
9:00 – 9:30<br>*30 min*	*Opening session*<br>— Welcome and introductions<br>— Meeting objectives<br>— Meeting logistics	Dr. Olufemi Oladapo<br>Dr. Ioannis Gallos<br>Dr. Fernando Althabe<br>Ms. Caitlin Williams
9:30 – 10:30<br>*60 min*	*Session 1*<br>— Brief review of the objectives and methods of the Delphi study<br>— Briefly summarize the state of the literature<br>— Findings from the first and second rounds of the Delphi study<br>— Questions	Ms. Verónica Pingray<br>Ms. Caitlin Williams<br>Ms. Verónica Pingray<br>Prof. Suellen Miller
10:30 – 10:45<br>*15 min*	*Coffee/Tea Break*	
10:45 – 12:45<br>*120 min*	*Session 2*<br>— Briefly review summary of findings on **intraoperative** period<br>— Guided discussions by topic	Ms. Verónica Pingray<br>Prof. Suellen Miller
12:45 – 13:30<br>*45 min*	*Lunch*	
13:30 – 14:15<br>*45 min*	*Continue Session 2*	Prof. Suellen Miller
14:15 – 15:45<br>*90 min*	*Session 3*<br>— Briefly review summary of findings on **postoperative** period<br>— Guided discussions by topic (detection, thresholds, first-response treatments)	Ms. Verónica Pingray<br>Dr. Fernando Althabe
15:45 – 16:00<br>*15 min*	*Coffee/Tea Break*	
16:00 – 17:00<br>*60 min*	*Continue Session 3*	Dr. Fernando Althabe
17:00 – 17:05<br>*5 min*	*Closing DAY 1*	Dr. Ioannis Gallos
**DAY 2: 28 September 2022**		
10:00 – 10:15<br>*15 min*	*Welcome*<br>— Objective and procedures for Day 2	Dr. Fernando Althabe
10:15 – 11:00<br>*45 min*	*Session 1*<br>— Presentation of a revised list of early detection and first-response treatments for intraoperative and postoperative PPH<br>— Final voting for the intraoperative and postoperative interventions and presentation of results	Prof. Suellen Miller<br>Ms. Caitlin Williams
11:00 – 11:15<br>*15 min*	*Coffee/Tea Break*	
11:15 – 12:35<br>*80 min*	*Session 2*<br>— Organization of an implementation strategy<br>— Guided discussion of possible additional considerations	Dr. Fernando Althabe<br>Prof. Suellen Miller
12:35 – 13:00<br>*25 min*	*Session 3*<br>— Conclusion and next steps	Dr. Fernando Althabe
13:00 – 13:05<br>*5 min*	*Closing DAY 2*	Dr. Arri Coomarasamy<br>Dr. Olufemi Oladapo

For peer review only - http://bmjopen.bmj.com/site/about/guidelines.xhtml

Supplementary File S3. In-person meeting discussion question guide

Early detection and first response to postpartum haemorrhage during and after caesarean birth
A Modified-Delphi Study

Pending controversies or disagreements issues to discuss
27-28 September 2022

DAY 1: 27 September 2022

Definitions:
The experts agreed that the same definition of PPH should be used for both vaginal and caesarean births (same regardless of the mode of birth). Currently the WHO defines PPH as blood loss at least 500 mL within 24 hours after birth.

Draft proposed list of intraoperative interventions

Early detection of PPH and thresholds for triggering first-response management

- Aspirated blood volume (+ weighted pads, sponges, gauzes, etc. if feasible) at least 1000 mL, OR
- Haemodynamic instability (blood pressure, heart rate, oximetry) with any blood loss volume

# Register the final amount of intraoperative blood loss and hand over this information to recovery area.

First-response treatment

- TXA (1g in 10 mL IV over 10 min) for all women
- Examine and rapidly initiate cause-specific first response:
    - If trauma: Rapid haemostasis: hysterorraphy, tears, wound.
    - If atony/placental cause: uterotonics and intra-abdominal uterine massage or exteriorize the uterus and massage
- Uterotonics for all women
- IV fluids with crystalloids for all women

Pending issues

<u>Thresholds</u>

Background: The experts agreed that blood loss of at least 1000 mL should be used as a threshold to trigger first-response management.

	%	Ranking
**Intraoperative**		
≥1000 mL blood loss OR signs of hemodynamic instability, whichever comes first	72.7	1
Hemodynamic instability alone, regardless of volume of blood loss	40.9	2
≥1000 ml (blood loss alone, regardless of signs of hemodynamic instability)	50.0	3
≥500 mL blood loss OR signs of hemodynamic instability, whichever comes first	50.0	4
**Postoperative**		
≥1000 mL blood loss OR signs of hemodynamic instability, whichever comes first	59.1	1
Hemodynamic instability alone, regardless of volume of blood loss	36.4	2
≥1000 ml (blood loss alone, regardless of signs of hemodynamic instability)	36.7	3
≥500 mL blood loss OR signs of hemodynamic instability, whichever comes first	50.0	4

We believe is important to discuss the overall implications of the different thresholds on clinical practice, in the context of the total blood that women lose during and after CS. At the meeting we will present a summary of what is known so far from PPH prevention trials.

For peer review only

Question 1: Now that we have reviewed this additional data, do we need to revise the top-ranked threshold from the previous rounds (at least 1000 mL OR signs of haemodynamic instability, whichever comes first)?

Question 1.1: If the answer is not to revise, then is it acceptable that the definition (500 mL) and threshold (1000 mL) for triggering action differ?

Treatments

Background: The experts agreed to an aetiology-based treatment approach, but also agreed that the uterotonic of choice should be administered for all women, regardless of presence of atony.

Treatment Options	Median
Examine and rapidly initiate cause-specific first response (e.g., if trauma: rapid surgical haemostasis; if atony/placental cause: uterotonics and uterine massage)	9
Tranexamic acid for all women with PPH during CS regardless of aetiology	8
Plasma expansion with crystalloids for all women with PPH during CS regardless of aetiology	7.5
Uterotonics for all women with PPH during CS regardless of aetiology	7

Question 2: How do we make a recommendation that encompasses these seemingly contradictory statements (i.e., should the first-response uterotonic be administered to all women or just to women with atony?)?

Background: The experts agreed that a first response to intraoperative PPH would be to give oxytocin and TXA. However, many women having a caesarean birth would have already received/or be receiving IV oxytocin, and perhaps TXA, for PPH prevention.

Treatment Options	Median
Examine and rapidly initiate cause-specific first response (e.g., if trauma: rapid surgical haemostasis; if atony/placental cause: uterotonics and uterine massage)	9
Tranexamic acid for all women with PPH during CS regardless of aetiology	8
Plasma expansion with crystalloids for all women with PPH during CS regardless of aetiology	7.5
Uterotonics for all women with PPH during CS regardless of aetiology	7

Question 3: How do we adapt first-response recommendations if women are already receiving oxytocin infusion (common practice and consensus statement) for PPH prevention?

Question 4: In the event that women will routinely be receiving TXA for PPH prevention during CS, what recommendations should we give should they begin to haemorrhage intraoperatively at less than 30 min from the first dose?

Regarding IV fluids

Background: In the survey, the experts agreed that plasma expansion with crystalloids should be used for all women with PPH during CS, regardless of aetiology.

Treatment Options	Median
Examine and rapidly initiate cause-specific first response (e.g., if trauma: rapid surgical haemostasis; if atony/placental cause: uterotonics and uterine massage)	9
Tranexamic acid for all women with PPH during CS regardless of aetiology	8
Plasma expansion with crystalloids for all women with PPH during CS regardless of aetiology	7.5
Uterotonics for all women with PPH during CS regardless of aetiology	7

Question 5: Although we have used “Plasma expansion with crystalloids” in previous surveys, it may not be the best term. Should we use a different term (e.g., increase IV fluids with crystalloids for hemodynamic maintenance)?

For peer review only

Question 6: What should our recommendations be about how to continue IV fluids infusion with crystalloids for hemodynamic maintenance?

Question 7: Do you think that we should be giving more details on type of crystalloids?

Additional considerations

Question 8: Are there/should there be additional considerations for any other first-response treatments during the intraoperative period?

Draft proposed list of postoperative interventions

Early detection of PPH and thresholds for triggering first-response management

- Haemodynamic instability (blood pressure, heart rate, oxygen saturation) with any blood loss volume, OR
- Accumulated blood loss at least 1000 mL (weighted pads + blood loss during CS)

# If blood loss during CS <500 ml: usual postoperative clinical monitoring.

# If blood loss during CS 500-999 ml: further assessment, preparedness, and close monitoring

First response treatment

- TXA (1g in 10 mL IV over 10 min) for all women
- Examine and rapidly initiate cause-specific first response:
  - If trauma suspected: re-laparotomy for surgical haemostasis
  - If atony: Uterotonics
- Uterotonics for all women
- IV fluids with crystalloids for all women

Pending issues

Background: During the survey, we asked questions about the detection methods, thresholds, and treatments using 2 hours as the postoperative period.

Question 9: Is it valid to extend the results to the first 24 hours, or is a different strategy needed for hours 1-2 hours postoperative versus 3-24 hours postoperative?

For example: Should a woman with cumulative blood loss of 1100 mL in the first 2 hrs have her bleeding managed in the same way as a woman with a cumulative blood loss of 1100 mL in the first 24 hrs?

Detection & thresholds

Background: The panel agreed that using **clinical signs of haemodynamic instability** is a more appropriate option for detecting postoperative PPH due to the risk of internal bleeding postoperative.

Additionally, the panel agreed that deploying volumetric methods (e.g., drapes) would be less feasible than monitoring vital signs. Reasons given were a) the possible low acceptability by women (due to discomfort) and b) the lack of feasibility of continued measurement during women's transfer and normal movements.

	How would you rate each of the methods below for early detection of PPH considering...			
Blood loss measurement and other PPH detection methods	usefulness?	feasibility?	acceptability?	resources required?
Clinical signs of haemodynamic instability	8.0	8.0	8.0	3.5
Volumetric/gravimetric + clinical signs of haemodynamic instability	7.0	7.0	NA	NA
Clinical judgement such as rate of flow and duration	5.0	6.0	7.0	2.0
Gravimetric	5.0	3.0	5.0	7.0
Volumetric	5.0	4.5	7.0	7.0
Visual estimation of blood loss	4.0	7.0	7.0	1.0
Visual charts and early warning scores (EWS)	7.0	7.0	6.5	6.0
Visual estimation + visual charts /EWS	7.0	7.0	NA	NA
Volumetric + gravimetric	7.0	5.0	NA	NA

Given the diverse realities of postoperative monitoring, priority should be given either to frequent monitoring of vital signs or the use of continuous monitoring devices, dependent on the availability of equipment and personnel.

Question 10: Consequently, what kind of guidance should we give, if any? How frequently should the woman's haemodynamic status be monitored? For how long? With what kind of devices?

Background: In addition to monitoring vital signs, providers should be **monitoring blood loss.** The experts agreed that in the postoperative period, providers should continue with the cumulative measurement of blood loss (i.e., adding intraoperative blood loss to postoperative blood loss to calculate total loss).

	**Median**
The volume of intraoperative blood loss should be taken into consideration when determining whether the postoperative threshold had been reached	8

Question 11: What guidance should we give to reflect cumulative intra- and postoperative blood loss?

We propose, **as an example**, the following approach to blood loss monitoring **in situations in which the woman's vital signs are stable**. ***If vital signs indicate haemodynamic instability, the guidance should be to initiate treatment regardless of postoperative blood loss.***

**Intraoperative**		**Postoperative**		
**Blood Loss**	**Action Indicated**	**Type of Monitoring**	**Cumulative Blood Loss**	**Action Indicated**
<500	No treatment	Normal monitoring	Cumulative blood loss <1000	Do not initiate treatment. Continue normal monitoring
500-999	No treatment	Further assessment, preparedness, and more frequent postop monitoring	Cumulative blood loss <1000	Do not initiate treatment. Return to normal monitoring
500-999	No treatment	Further assessment, preparedness, and more frequent postop monitoring	Cumulative blood loss >1000	Initiate treatment, and monitor the woman's response to the treatment
≥1000	Initiate treatment and monitor woman's response	Monitor woman's response to treatments	-	-

DAY 2: 28 September 2022

Question 12: How should we frame the first-response strategies? Or bundle?

- Algorithm?
- Checklist?
- Or a hybrid?

Question 13: Should we include preparedness interventions at the facility level?
Prepare for CS PPH

For peer review only - http://bmjopen.bmj.com/site/about/guidelines.xhtml

Blood loss measurement

- Calibrated containers
- Pump/aspirator/vacuum suction
- Pads/swabs/lap cloths, etc. with known dry weight
- Scale to weigh the above

Medications

- TXA
- Uterotonics (Oxytocin, ergometrine, sulprostone)
- Crystalloids

Haemodynamic Monitoring

- CRADLE device/ cardiac monitor/ oximeter/sphygmomanometer?

For peer review only

Supplementary File S4. List of contributors

EXPERTS

Fadhlun ALWY AL-BEITY - Tanzania
Edgardo ABALOS - Argentina
Sabaratnam ARULKUMARAN - UK
Nabhan ASHRAF - Egypt
Brendan CARVALHO - USA
Catherine DENEUX-THARAUX - France
Alexander DUMONT – France
Maria Fernanda ESCOBAR VIDARTE - Colombia
Cherrie EVANS - USA
Sue FAWCUS - South Africa
Hadiza GALADANCI - Nigeria
Justus HOFMEYR - South Africa
Caroline HOMER - Australia
Tippawan LIABSUETRAKUL - Thailand
Pisake Lumbiganon - Thailand
Elliott MAIN - USA
Judith Maua ONG'AYI - Kenya
PHAN NGUYEN Quoc Thuan - Vietnam
Zahida QURESHI - Kenya
John VARALLO - USA

OBSERVERS

Arri COOMARASAMY - UK
Inês NUNES - Portugal
Andrew WEEKS - UK
Claudio SOSA - Uruguay

STEERING GROUP

Fernando ALTHABE - Argentina
Suellen MILLER - USA
Veronica PINGRAY - Argentina
Caitlin R. WILLIAMS - USA

WHO SECRETARIAT

Ioannis GALLOS
Olufemi OLADAPO
Mariana WIDMER

For peer review only

References

1. World Health Organization. WHO recommendations for the prevention and treatment of postpartum haemorrhage. Geneva: World Health Organization; 2012. 41- p.
2. Mavrides E, Allard S, Chandraharan E, Collins P, Green L, Hunt BJ, et al. Prevention and management of postpartum haemorrhage. BJOG: An International Journal of Obstetrics & Gynaecology. 2016;124:e106-e49.
3. Sentilhes L, Vayssière C, Deneux-Tharaux C, Aya AG, Bayoumeu F, Bonnet MP, et al. Postpartum hemorrhage: Guidelines for clinical practice from the French College of Gynaecologists and Obstetricians (CNGOF): In collaboration with the French Society of Anesthesiology and Intensive Care (SFAR). European Journal of Obstetrics and Gynecology and Reproductive Biology. 2016;198(2016):12-21.
4. Institute of O, Gynaecologists Royal College of Physicians of I, Directorate of S, Clinical Programmes Health Service E. Prevention and management of primary postpartum haemorrhage. 2014. p. 12-26.
5. RANZCOG. Management of Postpartum Haemorrhage (PPH). 2017. p. 1-14.
6. Muñoz M, Stensballe J, Ducloy-Bouthors A-S, Bonnet M-P, De Robertis E, Fornet I, et al. Patient blood management in obstetrics: prevention and treatment of postpartum haemorrhage. A NATA consensus statement. Blood transfusion = Trasfusione del sangue. 2019;17(2):112-36.
7. Schlembach D, Helmer H, Henrich W, von Heymann C, Kainer F, Korte W, et al. Peripartum haemorrhage, diagnosis and therapy. Guideline of the DGGG, OEGGG and SGGG (S2k Level, AWMF Registry No. 015/063, March 2016). Geburtshilfe und Frauenheilkunde. 2018;78(04):382-99.
8. Minakami H, Maeda T, Fujii T, Hamada H, Iitsuka Y, Itakura A, et al. Guidelines for obstetrical practice in Japan: Japan Society of Obstetrics and Gynecology (JSOG) and Japan Association of Obstetricians and Gynecologists (JAOG) 2014 edition. Journal of Obstetrics and Gynaecology Research. 2014;40(6):1469-99.
9. World Health Organization. WHO Recommendation on Tranexamic Acid for the Treatment of Postpartum Haemorrhage. Geneva: World Health Organization; 2017. 41- p.
10. Lalonde A, Motherhood FS, Newbon Health C. Prevention and treatment of postpartum hemorrhage in low-resource settings. International Journal of Gynecology & Obstetrics. 2012;117(2):108-18.
11. ACOG. Postpartum Hemorrhage. Obstetrics & Gynecology. 2017;183:e168-e86.
12. Queensland Clinical Guidelines. Maternity and neonatal clinical guideline: Primary postpartum haemorrhage. Queensland Health. 2018:1-39.
13. CMQCC, Health CDoP. Improving Health Care Response to Obstetric Hemorrhage Version 2.0: A California Quality Improvement Toolkit2015.
14. Leduc D, Senikas V, Lalonde AB. No. 235-Active Management of the Third Stage of Labour: Prevention and Treatment of Postpartum Hemorrhage. Journal of Obstetrics and Gynaecology Canada. 2018;40(12):e841-e55.
15. NICE. Caesarean birth 2021 [cited 2023 Apr 30]. Available from: https://www.nice.org.uk/guidance/ng192.
16. World Health Organization. Managing complications in pregnancy and childbirth: A guide for midwives and doctors. 2nd ed. Geneva: World Health Organization; 2017. 492- p.
17. Smith CM, Borders AE, King TL. Quantitative Blood Loss in Obstetric Hemorrhage: ACOG COMMITTEE OPINION SUMMARY, Number 794. Obstetrics and gynecology. 2019;134(6):1368-9.
18. World Health O. WHO Recommendation on Tranexamic Acid for the Treatment of Postpartum Haemorrhage. WHO Recommendation on Tranexamic Acid for the Treatment of Postpartum Haemorrhage. 2017:41-.
19. Gallos ID, Papadopoulou A, Man R, Athanasopoulos N, Tobias A, Price MJ, et al. Uterotonic agents for preventing postpartum haemorrhage: A network meta-analysis. Cochrane Database of Systematic Reviews. 2018;2018(12).
20. Kellie FJ, Wandabwa JN, Mousa HA, Weeks AD. Mechanical and surgical interventions for treating primary postpartum haemorrhage. Cochrane Database of Systematic Reviews. 2020.

For peer review only

21. Parry Smith WR, Papadopoulou A, Thomas E, Tobias A, Price MJ, Meher S, et al. Uterotonic agents for first-line treatment of postpartum haemorrhage: A network meta-analysis. Cochrane Database of Systematic Reviews. 2020;2020(11).
22. Hancock A, Weeks AD, Lavender DT. Is accurate and reliable blood loss estimation the 'crucial step' in early detection of postpartum haemorrhage: an integrative review of the literature. BMC Pregnancy and Childbirth 2015 15:1. 2015;15(1):1-9.
23. Andrikopoulou M, D'Alton ME. Postpartum hemorrhage: Early identification challenges. Seminars in Perinatology. 2019;43(1):11-7.
24. Diaz V, Abalos E, Carroli G. Methods for blood loss estimation after vaginal birth. Cochrane Database of Systematic Reviews. 2018;2018(9).
25. Lertbunnaphong T, Lapthanapat N, Leetheeragul J, Hakularb P, Ownon A. Postpartum blood loss: visual estimation versus objective quantification with a novel birthing drape. Singapore Medical Journal. 2016;57(06):325-8.
26. Patel A, Goudar SS, Geller SE, Kodkany BS, Edlavitch SA, Wagh K, et al. Drape estimation vs. visual assessment for estimating postpartum hemorrhage. International Journal of Gynecology & Obstetrics. 2006;93(3):220-4.
27. Zhang WH, Deneux-Tharaux C, Brocklehurst P, Juszczak E, Joslin M, Alexander S. Effect of a collector bag for measurement of postpartum blood loss after vaginal delivery: cluster randomised trial in 13 European countries. BMJ. 2010;340(feb01 1):c293-c.
28. Ambardekar S, Shochet T, Bracken H, Coyaji K, Winikoff B. Calibrated delivery drape versus indirect gravimetric technique for the measurement of blood loss after delivery: a randomized trial. BMC Pregnancy and Childbirth 2014 14:1. 2014;14(1):1-6.
29. Atukunda EC, Mugyenyi GR, Obua C, Atuhumuza EB, Musinguzi N, Tornes YF, et al. Measuring post-partum haemorrhage in low-resource settings: The diagnostic validity of weighed blood loss versus quantitative changes in hemoglobin. PLOS ONE. 2016;11(4):e0152408-e.
30. Ushida T, Kotani T, Imai K, Nakano-Kobayashi T, Nakamura N, Moriyama Y, et al. Shock index and postpartum hemorrhage in vaginal deliveries: A multicenter retrospective study. Shock. 2021;55(3):332-7.
31. Borovac-Pinheiro A, Ribeiro FM, Morais SS, Pacagnella RC. Shock index and heart rate standard reference values in the immediate postpartum period: A cohort study. PLOS ONE. 2019;14(6):e0217907-e.
32. El Ayadi AM, Nathan HL, Seed PT, Butrick EA, Hezelgrave NL, Shennan AH, et al. Vital sign prediction of adverse maternal outcomes in women with hypovolemic shock: The role of shock index. PLOS ONE. 2016;11(2):e0148729-e.
33. Pacagnella RC, Borovac-Pinheiro A, Silveira C, Siani Morais S, Argenton JLP, Souza JP, et al. The golden hour for postpartum hemorrhage: Results from a prospective cohort study. International Journal of Gynecology & Obstetrics. 2021:ijgo.13823-ijgo.
34. Tanacan A, Fadiloglu E, Unal C, Beksac MS. Importance of shock index in the evaluation of postpartum hemorrhage cases that necessitate blood transfusion. Women & Health. 2020;60(9):1070-8.
35. Lee SY, Kim HY, Cho GJ, Hong SC, Oh MJ, Kim HJ. Use of the shock index to predict maternal outcomes in women referred for postpartum hemorrhage. International Journal of Gynecology & Obstetrics. 2018:ijgo.12714-ijgo.
36. Vousden N, Lawley E, Nathan HL, Seed PT, Gidiri MF, Goudar S, et al. Effect of a novel vital sign device on maternal mortality and morbidity in low-resource settings: a pragmatic, stepped-wedge, cluster-randomised controlled trial. The Lancet Global Health. 2019;7(3):e347-e56.
37. Umar A, Ameh CA, Muriithi F, Mathai M. Early warning systems in obstetrics: A systematic literature review. PLOS ONE. 2019;14(5):e0217864-e.
38. World Health Organization. WHO recommendations: Uterotonics for the prevention of postpartum haemorrhage. Geneva: World Health Organization; 2018. p. 53-.
39. World Health Organization. WHO recommendation on uterine balloon tamponade for the treatment of postpartum haemorrhage. Geneva: World Health Organization; 2021.
40. Shakur H, Beaumont D, Pavord S, Gayet-Ageron A, Ker K, Mousa HA. Antifibrinolytic drugs for treating primary postpartum haemorrhage. Cochrane Database of Systematic Reviews. 2018;2018(2).

For peer review only

41. Kavak SB, Atilgan R, Demirel İ, Celik E, İlhan R, Sapmaz E. Endouterine hemostatic square suture vs. Bakri balloon tamponade for intractable hemorrhage due to complete placenta previa. Journal of Perinatal Medicine. 2013;41(6).
42. Khalil MI, Al-Dohami H, Aldahish MM. A method to improve the effectiveness of the Bakri balloon for management of postpartum hemorrhage at cesarean. International Journal of Gynecology & Obstetrics. 2011;115(2):198-200.
43. Pileggi-Castro C, Nogueira-Pileggi V, Tunçalp Ö, Oladapo OT, Vogel JP, Souza JP. Non-pneumatic anti-shock garment for improving maternal survival following severe postpartum haemorrhage: a systematic review. Reprod Health. 2015;12:28.

For peer review only

S1 CREDES Checklist:

Recommendations for the Conducting and REporting of DElphi Studies (CREDES)[1]

Strategies for optimising early detection and first response management of postpartum haemorrhage at caesarean birth: A modified Delphi-based international expert consensus

**Items of reporting**	**Reported on page**
Purpose and rationale. The purpose of the study should be clearly defined and demonstrate the appropriateness of the use of the Delphi technique as a method to achieve the research aim. A rationale for the choice of the Delphi technique as the most suitable method needs to be provided.	5
Expert panel. Criteria for the selection of experts and transparent information on recruitment of the expert panel, sociodemographic details including information on expertise regarding the topic in question, (non)response and response rates over the ongoing iterations should be reported.	6, SF 4
Description of the methods. The methods employed need to be comprehensible; this includes information on preparatory steps (How was available evidence on the topic in question synthesised?), piloting of material and survey instruments, design of the survey instrument(s), the number and design of survey rounds, methods of data analysis, processing and synthesis of experts' responses to inform the subsequent survey round and methodological decisions taken by the research team throughout the process.	5-7
Procedure. Flow chart to illustrate the stages of the Delphi process, including a preparatory phase, the actual 'Delphi rounds', interim steps of data processing and analysis, and concluding steps.	Figure 1
Definition and attainment of consensus. It needs to be comprehensible to the reader how consensus was achieved throughout the process, including strategies to deal with non-consensus.	7
Results. Reporting of results for each round separately is highly advisable in order to make the evolving of consensus over the rounds transparent. This includes figures showing the average group response, changes between rounds, as well as any modifications of the survey instrument such as deletion, addition or modification of survey items based on previous rounds.	9-17
Discussion of limitations. Reporting should include a critical reflection of potential limitations and their impact of the resulting guidance.	4 (Box), 19
Adequacy of conclusions. The conclusions should adequately reflect the outcomes of the Delphi study with a view to the scope and applicability of the resulting practice guidance.	21
Publication and dissemination. The resulting guidance on good practice in palliative care should be clearly identifiable from the publication, including recommendations for transfer into practice and implementation. If the publication does not allow for a detailed presentation of either the resulting practice guidance or the methodological features of the applied Delphi technique, or both, reference to a more detailed presentation elsewhere should be made (e.g. availability of the full guideline from the authors or online; publication of a separate paper reporting on methodological details and particularities of the process (e.g. persistent disagreement and controversy on certain issues)). A dissemination plan should include endorsement of the guidance by professional associations and health care authorities to facilitate implementation.	19 - 20

1. Jünger S, Payne SA, Brine J, Radbruch L, Brearley SG. Guidance on Conducting and REporting DElphi Studies (CREDES) in palliative care: Recommendations based on a methodological systematic review. Palliat Med. 2017;31: 684–706. doi:10.1177/0269216317690685

For peer review only

BMJ Open

Strategies for optimising early detection and obstetric first response management of postpartum haemorrhage at caesarean birth: A modified Delphi-based international expert consensus

Journal:	*BMJ Open*
Manuscript ID	bmjopen-2023-079713.R1
Article Type:	Original research
Date Submitted by the Author:	05-Feb-2024

Complete List of Authors:	Pingray, Verónica; Institute for Clinical Effectiveness and Health Policy,<br>Williams, Caitlin R.; University of North Carolina at Chapel Hill Gillings School of Global Public Health, Department of Maternal & Child Health; Institute for Clinical Effectiveness and Health Policy,<br>Al-beity, Fadhlun M; Muhimbili University of Health and Allied Sciences, Department of Obstetrics & Gynaecology ; Karolinska Institute, Department of Obstetrics & Gynaecology<br>Abalos, Edgardo; Secretaría de Salud Pública de Rosario, Maternidad Martin<br>Arulkumaran , Sabaratnam; Department of Obstetrics & Gynaecology, St George’s Hospital,<br>Blumenfeld, Alejandro; Institute for Clinical Effectiveness and Health Policy; Universidad de Buenos Aires, Department of Public Health, Faculty of Medicine<br>Carvalho, B; Stanford University School of Medicine,<br>Deneux-Tharaux, Catherine; INSERM<br>Downe, Soo; University of Central Lancashire, Research in childbirth and health; University of Central Lancashire, THRIVE Centre, School of Heath and Community Studies<br>Dumont, Alexandre; Université Paris Cité, CEPED; INSERM<br>Escobar, Maria Fernanda; Fundación Valle del Lili, Departamento de Ginecología y Obstetricia; Universidad Icesi, Facultad de Ciencias de la Salud<br>Evans, Cherrie; Jhpiego/USA, Technical Leadership & Innovations Office<br>Fawcus, Sue ; University of Cape Town, Department of Obstetrics and Gynaecology<br>Galadanci, HS; Aminu Kano Teaching Hospital, Department of Obstetrics and Gynaecology; Bayero University Kano, Africa Center of Excellence for Population Health and Policy<br>Hoang, Diem-Tuyet ; Hung Vuong Hospital, Obstetrics and Gynecology<br>Hofmeyr, G Justus ; University of Botswana, Department of Obstetrics and Gynaecology; University of the Witwatersrand, Effective Care Research Unit,<br>Homer, Caroline ; Burnet Institute<br>Lewis, Ayodele; Amherst College<br>Liabsuetrakul, Tippawan; Prince of Songkla University, Department of Epidemiology; Prince of Songkla University, Department of Obstetrics & Gynecology

Lumbiganon, Pisake; Khon Kaen University, Department of Obstetrics and Gynaecology
Main, Elliot; Stanford University, Department of Obstetrics & Gynecology - Maternal Fetal Medicine; California Maternal Quality Care Collaborative
Maua, Judith; Liverpool School of Tropical Medicine
MURIITHI, FRANCIS GITHAE; University of Birmingham, Institute of Metabolism and Systems Research; Nottingham University Hospitals NHS Trust, Department of Obstetrics and Gynaecology
Nabhan, Ashraf; Ain Shams University Faculty of Medicine, Department of Obstetrics & Gynecology
NUNES, Inês; Centro Hospitalar de Vila Nova de Gaia Espinho EPE, Department of Obstetrics and Gynaecology; University of Porto, CINTESIS - Center for Health Technology and Services Research
Ortega, Vanesa; Institute for Clinical Effectiveness and Health Policy, Department of Mother and Child Health Research
Phan, Thuan; University of Liverpool, Department of Women's and Children's Health; Tu Du Hospital, Department of Delivery
Qureshi , Zahida ; University of Nairobi Department of Obstetrics and Gynecology,
Sosa, Claudio; Pan American Health Organization, Woman and Reproduction Health Unit at Maternal Health at the Latin American Center of Perinatology (CLAP/WR); Universidad de la República Uruguay, Department of Obstetrics and Gynecology. School of Medicine
Varallo, John; Global Surgery Foundation, Women's Health
Weeks, Andrew; University of Liverpool, Department of Women's and Children's Health; Liverpool Women's Hospital
Widmer, Mariana; WHO, UNDP-UNFPA-UNICEF-WHO-World Bank Special Programme of Research, Development and Research Training in Human Reproduction (HRP), Department of Sexual and Reproductive Health and Research
Oladapo, Olufemi ; World Health Organization, UNDP-UNFPA-UNICEF-WHO-World Bank Special Programme of Research, Development and Research Training in Human Reproduction (HRP), Department of Sexual and Reproductive Health and Research
Gallos, Ioannis; World Health Organization, UNDP-UNFPA-UNICEF-WHO-World Bank Special Programme of Research, Development and Research Training in Human Reproduction (HRP), Department of Sexual and Reproductive Health and Research
Coomarasamy, Arri; University of Birmingham
Miller, Suellen; University of California San Francisco, Bixby Center for Global reproductive Health
Althabe, Fernando ; World Health Organization, UNDP-UNFPA-UNICEF-WHO-World Bank Special Programme of Research, Development and Research Training in Human Reproduction (HRP), Department of Sexual and Reproductive Health and Research

**Primary Subject Heading**:	Obstetrics and gynaecology
Secondary Subject Heading:	Global health
Keywords:	OBSTETRICS, Maternal medicine < OBSTETRICS, Postpartum Period, Systematic Review, Clinical Decision-Making

SCHOLARONE™
Manuscripts

I, the Submitting Author has the right to grant and does grant on behalf of all authors of the Work (as defined in the below author licence), an exclusive licence and/or a non-exclusive licence for contributions from authors who are: i) UK Crown employees; ii) where BMJ has agreed a CC-BY licence shall apply, and/or iii) in accordance with the terms applicable for US Federal Government officers or employees acting as part of their official duties; on a worldwide, perpetual, irrevocable, royalty-free basis to BMJ Publishing Group Ltd ("BMJ") its licensees and where the relevant Journal is co-owned by BMJ to the co-owners of the Journal, to publish the Work in this journal and any other BMJ products and to exploit all rights, as set out in our licence.

The Submitting Author accepts and understands that any supply made under these terms is made by BMJ to the Submitting Author unless you are acting as an employee on behalf of your employer or a postgraduate student of an affiliated institution which is paying any applicable article publishing charge ("APC") for Open Access articles. Where the Submitting Author wishes to make the Work available on an Open Access basis (and intends to pay the relevant APC), the terms of reuse of such Open Access shall be governed by a Creative Commons licence – details of these licences and which Creative Commons licence will apply to this Work are set out in our licence referred to above.

Other than as permitted in any relevant BMJ Author's Self Archiving Policies, I confirm this Work has not been accepted for publication elsewhere, is not being considered for publication elsewhere and does not duplicate material already published. I confirm all authors consent to publication of this Work and authorise the granting of this licence.

For peer review only

Strategies for optimising early detection and obstetric first response management of postpartum haemorrhage at caesarean birth: A modified Delphi-based international expert consensus

Pingray V[†*], Williams CR†, Alwy Al-beity F, Abalos E, Arulkumaran S, Blumenfeld A, Carvalho B, Deneux-Tharaux C, Downe S, Dumont A, Escobar MF, Evans C, Fawcus S, Galadanci H, Hoang T, Hofmeyr GJ, Homer C, Lewis A, Liabsuetrakul T, Lumbiganon P, Main E, Maua J, Muriithi F, Nabhan A, Nunes I, Ortega V, Phan T, Qureshi Z, Sosa C, Varallo J, Weeks A, Widmer M, Oladapo OT, Gallos I, Coomarasamy A, Miller S[‡], Althabe F[‡].

[†]These authors contributed equally. [‡]SM and FA share senior co-authorship.

[*]Corresponding author: Veronica Pingray, Institute for Clinical Effectiveness and Health Policy Email: vpingray@iecs.org.ar.

For peer review only - http://bmjopen.bmj.com/site/about/guidelines.xhtml

ABSTRACT

OBJECTIVE: There are no globally agreed upon strategies on early detection and first response management of postpartum haemorrhage during and after caesarean birth. Our study aimed to develop an international expert's consensus on evidence-based approaches for early detection and obstetric first response management of PPH intraoperatively and postoperatively in caesarean birth.

DESIGN: Systematic review and three-stage modified-Delphi expert consensus.

SETTING: International.

POPULATION: Panel of 22 global experts in postpartum haemorrhage with diverse backgrounds, and gender, professional, and geographic balance.

OUTCOME MEASURES: Agreement or disagreement on strategies for early detection and first response management of postpartum haemorrhage at caesarean birth.

RESULTS: Experts agreed that the same PPH definition should apply to both vaginal and caesarean birth. For the intraoperative phase, the experts agreed that early detection should be accomplished via quantitative blood loss measurement, complemented by monitoring the woman's haemodynamic status; and that first response should be triggered once the woman loses at least 500 mL of blood with continued bleeding or when she exhibits clinical signs of haemodynamic instability, whichever occurs first. For the first response, experts agreed on immediate administration of uterotonics and tranexamic acid, examination to determine aetiology, and rapid initiation of cause-specific responses. In the postoperative phase, the experts agreed that caesarean birth-related PPH should be detected primarily via frequently monitoring the woman's haemodynamic status and clinical signs and symptoms of internal bleeding, supplemented by cumulative blood loss assessment performed quantitatively or by visual estimation. Postoperative first response was determined to require an individualised approach.

CONCLUSION: These agreed-upon proposed approaches could help improve the detection of PPH in the intra and postoperative phases of caesarean birth and the first response management of intraoperative PPH. Determining how best to implement these strategies is a critical next step.

For peer review only - http://bmjopen.bmj.com/site/about/guidelines.xhtml

KEY WORDS

Postpartum haemorrhage; caesarean birth; early detection; first response management; tranexamic acid; expert consensus

For peer review only

For peer review only - http://bmjopen.bmj.com/site/about/guidelines.xhtml

STRENGTHS AND LIMITATIONS OF THIS STUDY

Strengths

- Use of a rigorous and systematic process to identify and synthesise high-quality PPH evidence in the literature.
- The selection of the expert panellists ensured a wide range of perspectives to enhance the utility and applicability of this consensus to a wide range of clinical settings.
- There was a very low rate of loss to follow-up and the first two rounds of the modified Delphi process were blinded to avoid social acceptability bias, and the hybrid meeting was facilitated to ensure that all panellists had equal opportunity to contribute to the discussion.

Limitations

- Due to the dearth of quality evidence on PPH related to caesarean birth, experts often had to extrapolate from evidence on interventions recommended for PPH in vaginal birth or make decisions based on their experiences.
- Given the highly technical content, we did not include recipients of these interventions, or their representatives, among the panellists.

For peer review only - http://bmjopen.bmj.com/site/about/guidelines.xhtml

INTRODUCTION

Deaths from postpartum haemorrhage (PPH), the leading direct cause of maternal mortality globally, are potentially preventable with timely diagnosis and management (1,2). The risk of PPH is significantly higher with caesarean birth than vaginal birth, especially in cases of emergency caesarean birth (3). With global caesarean birth rates increasing, PPH during and after caesarean birth is a growing concern (4). The impact is particularly acute in low and middle-income countries (LMICs), where 32% of all maternal deaths after caesarean birth are related to PPH (5). In some LMICs, caesarean births outnumber vaginal births (6). Several factors challenge effective response to PPH in LMICs. These countries have well-documented difficulties accessing surgical services, skilled staff, and blood/blood products (7). Even when access concerns are addressed, the use of interventions to detect and manage PPH is often inconsistent (8,9).

A standardised approach to PPH management has been shown to improve outcomes, including significantly reducing severe PPH rates amongst women giving birth vaginally (10). Similarly, studies including women having caesarean birth suggest a reduction in severe morbidity associated with the use of comprehensive haemorrhage protocols (11,12). The World Health Organization (WHO) has published and updated recommendations for the prevention and treatment of PPH (2,13,14). However, these recommendations neither detail methods for early detection of PPH during and after caesarean birth nor clearly indicate when to initiate treatment (i.e., the 'trigger' criteria), both of which may contribute to observed variations in clinical practice (2,7,15). PPH management practices may vary depending on whether the haemorrhage occurs during or after the surgical procedure (16). Proposing standardised and evidence-based global strategies may help to reduce practice variations and improve the quality of care. Our study aimed to develop an international consensus on standardised approaches for PPH detection and obstetric first response management for women who develop primary PPH during and after caesarean birth, and at the time of initiating treatment, the suspected aetiology is uterine atony, traumatic PPH, or unknown.

METHODS

For peer review only - http://bmjopen.bmj.com/site/about/guidelines.xhtml

The study involved a systematic review and an expert consensus using a three-stage modified-Delphi process.

Systematic review

A systematic review of published national and international guidelines for PPH prevention and management was conducted to identify interventions for collecting and measuring blood loss, methods for detecting PPH, thresholds for treatment, and first response conservative obstetric interventions to manage PPH both during surgery (intraoperative) and after surgery (postoperative). The evidence summarised involved treatments options for women who develop primary PPH during or after CS, and at the time of initiating treatment, the suspected aetiology is either uterine atony, traumatic PPH, or unknown. Treatments for managing women with a diagnosis of antepartum haemorrhage, coagulopathy, placenta praevia, or placenta accreta were not included, given that treatments are usually specific to each aetiology. To be included, the guidelines needed to include guidance on the detection or management of PPH during or after caesarean birth. The literature search in PubMed, EMBASE, CINAHL, and Cochrane Library databases included papers published from January 2012 to July 2022 (Supplementary File S1). The search was complemented by reviewing the English-language grey literature to identify guidelines.

Since few of these guidelines were focused specifically on the intra- or postoperative phases or described PPH detection methods, an additional systematic search was conducted, focused on PPH detection and conservative obstetric first response management during and after caesarean birth. Peer-reviewed systematic reviews of RCTs were eligible. Subject matter experts were consulted to add any relevant peer-reviewed articles missed by the systematic search.

Titles and abstracts of both guidelines and systematic reviews of RCTs were screened by pairs of independent reviewers who subsequently reviewed full texts, conducted quality appraisals, and extracted data using previously piloted forms. Only guidelines with AGREE II scores between 5 and 7 and systematic reviews with modified-AMSTAR quality assessment of “Moderate” or “High” were eligible for data extraction (17,18). The results of the systematic review were used to inform the development of the Delphi surveys and to provide the experts with summaries of the existing evidence. Additional methodological details con be found in the Supplementary Materials (Supplementary File S2).

Expert consensus

For peer review only - http://bmjopen.bmj.com/site/about/guidelines.xhtml

A three-stage modified Delphi process was conducted between December 2021 and September 2022, with two rounds of individual online surveys, followed by a third round: a hybrid (virtual and in-person) meeting with group discussions and final voting. Twenty-five PPH experts with the knowledge and skills to critically assess scientific evidence were invited to participate in all three rounds. They included specialists in nursing, midwifery, obstetrics, surgery, and anaesthesia. The experts were selected to ensure gender, professional, and geographic balance. Most experts were co-authors of recent national and international guidelines or principal or co-investigators of PPH clinical trials. The same experts were invited to participate in all three rounds. In the third round, observers representing professional associations and WHO regional offices, or who were leaders in PPH research were invited to share their views, but were not eligible to vote.

Based on the findings of the systematic review, questionnaires with open- and close-ended questions were developed, piloted, and administered using Survey Monkey™. A summary of the themes and interventions included in the surveys and criteria used to guide judgements are described in Box 1. The criteria, methods, interventions, and other items included in the surveys were presented with definitions to facilitate interpretation. The themes were explored separately for the intraoperative and postoperative phases. Experts had to consider PPH detection methods and first response obstetric interventions to be applied in any type of CEmONC (Comprehensive Emergency Obstetric and Newborn Care services) facility, and applicable for primary PPH. In line with the scope of the systematic review, the consensus targeted conservative first-response obstetric interventions applicable for women with any cause of PPH until the main cause of PPH is identified. Consequently, it did not target the first response to women with PPH due to placenta praevia, placenta accreta, coagulopathies, and retained tissue. However, it will apply to women with atonic and traumatic PPH. Although most cases of PPH are controlled by the simultaneous application of obstetric interventions and haemostatic support (19), this consensus focused mainly on obstetric interventions and not haemostatic resuscitation and treatment of anaemia and coagulopathy.

Experts were asked to consider the postoperative PPH phase as only the first two hours immediately after the operation. Each online survey was available for response for six weeks, and three reminders were sent to participants with incomplete or no responses. In the first round, experts were asked to rate caesarean-related PPH definitions, detection methods, thresholds to trigger treatments, and first response conservative obstetric interventions. In the second round, experts received their previous individual ratings and group rating distributions. They were asked to re-rate detection methods with disagreement, rank-order the thresholds and first response treatments that had previously received

For peer review only - http://bmjopen.bmj.com/site/about/guidelines.xhtml

high ratings, and rate new questions that emerged from experts' comments in open-ended questions from Round 1. In the third round, experts met for a two-day hybrid meeting to discuss areas of divergence between surveys' findings and to rate (anonymously) the final sets of interventions. The agenda and questions guide used to facilitate the discussion are available in the Supplementary Materials (Supplementary File S3 and S4). Figure 1 outlines the process of consensus building.

Median group rating and disagreement index (DI) were calculated to summarise experts' ratings and to measure agreement. A DI < 1 indicated agreement, while a DI ≥ 1 indicated disagreement (20). The RAND/UCLA appropriateness scale was used to classify interventions as "appropriate", "inappropriate", or "uncertain" (20). Interventions with median ratings in the top third of the appropriateness scale (7–9) were classified as "appropriate"; those in the bottom third were classified as "inappropriate" (1–3); and those with intermediate median ratings were classified as "uncertain" (4–6). Domains with disagreement among the experts were also classified as "uncertain" (Supplementary Figure S1).

Patient and public involvement: Given the highly technical content, we did not include recipients of these interventions, or their representatives, among the panellists. This limitation is addressed in the discussion.

For peer review only - http://bmjopen.bmj.com/site/about/guidelines.xhtml

Box 1. Themes explored and criteria used to guide assessments

**Themes**	**Criteria and items included in each questionnaire**
**PPH definitions**	• Appropriateness of using a single definition for PPH, regardless of mode of birth<br>• Timeframe for postoperative PPH
**Early detection methods**<br>*Intra- and postoperative*	**Criteria:** clinical usefulness, feasibility of use in all settings attending caesarean birth, acceptability to key stakeholders, and estimate of resources required<br>**Items**:<br>• Visual estimation of blood loss<br>• Volumetric assessment of blood loss<br>• Gravimetric assessment of blood loss<br>• Clinical signs of haemodynamic instability<br>• Visual charts and early warning scores<br>• Clinical judgement (e.g., rate of flow, duration)<br>• Volumetric + gravimetric assessment of blood loss<br>• Volumetric/gravimetric assessment of blood loss + clinical signs of haemodynamic instability<br>• Visual estimation + visual charts/early warning systems (EWS)
**Thresholds for action**<br>*Intra- and postoperative*	**Criteria:** accuracy, feasibility of use in all settings attending caesarean birth, and acceptability to key stakeholders<br>**Items:**<br>• ***One step approach*** (Single threshold triggers full response protocol)<br>○ At least 500 mL blood loss alone<br>○ At least 1000 mL blood loss alone<br>○ Haemodynamic instability alone<br>○ At least 500 mL blood loss OR signs of haemodynamic instability<br>○ At least 1000 mL blood loss OR signs of haemodynamic instability<br>• ***Two step approach*** (Lower threshold triggers further assessment, preparedness, and close monitoring; higher threshold triggers initiation of treatment)<br>○ Lower threshold of at least 500 mL, and higher threshold of at least 1000 mL blood loss OR signs of haemodynamic instability<br>○ Lower threshold of at least 1000 ml, and higher threshold of at least 2000 mL blood loss OR signs of haemodynamic instability
**First response conservative obstetric interventions**<br>*Intra- and postoperative*	**Criteria**: balance of effects, feasibility of use in all settings attending caesarean birth, acceptability to key stakeholders, estimate of resources required, equity<br>**Items:**<br>• Oxytocin<br>• Carbetocin<br>• Tranexamic acid<br>• Compressive sutures<br>• Bimanual compression<br>• Uterine massage<br>• Oxytocin-ergometrine fixed dose<br>• Prostaglandin<br>• Ergometrine<br>• Non-pneumatic anti-shock garment<br>• External aortic compression<br>• Intrauterine balloon tamponade

Note: NA=not applicable

For peer review only

For peer review only - http://bmjopen.bmj.com/site/about/guidelines.xhtml

RESULTS

The systematic search identified 802 guidelines and systematic reviews. After screening and quality appraisal, 17 guidelines (2,13,15,21–34), four systematic reviews (35–38), and 15 peer-reviewed studies (39–54) were included (Supplementary Figure S2). Included guidelines and systematic reviews identified six PPH definitions, five PPH detection methods, ten blood loss collection devices, seven thresholds to initiate treatment, and 14 obstetric interventions to manage PPH conservatively. Results are in Supplementary Tables S1-S4.

Of the 25 experts invited, 22 agreed to participate in the Delphi process (Supplementary File S5). All completed the first and second rounds, while 20/22 participated and voted in the third round. The experts who completed all rounds were from 11 countries from all WHO world regions (six from the African Region, one from the Eastern Mediterranean Region, three from the European Region, six from the Region of the Americas, two from the South-East Asian Region, and two from the Western Pacific Region). They had different professional backgrounds (obstetricians and gynaecologists, anaesthetists, surgeons, nurse-midwives, and midwives) and were gender-balanced (12 men and 10 women). In addition, four observers participated in the discussion during the third round but did not vote.

The median ratings and measures of agreement obtained from the first and second rounds of online surveys are given in Supplementary Tables S5-S8 and Figure S3. Experts' ratings and agreements in the third round are given in Table 1. Consensus was reached for (a) using a single definition for PPH, regardless of mode of birth, (b) early detection of PPH at caesarean birth and thresholds to initiate treatment in the intraoperative phase, (c) clinical interventions for conservative obstetric first response management of intraoperative PPH, and (d) early detection of PPH after caesarean birth and thresholds to initiate treatment in the postoperative phase. However, the first response treatment in the postoperative phase was determined to require an individualised approach.

For peer review only - http://bmjopen.bmj.com/site/about/guidelines.xhtml

Table 1. Experts' ratings and agreement on early detection and first response to intraoperative and postoperative PPH

Set of clinical interventions	Rating distribution			Agreement			
	# votes in each interval			Median (IQR) in a 1 - 9 differential scale	RAND DI	Qualitative scale	Appropriateness scale
	1 - 3	4 - 6	7 - 9				
**Intraoperative**							
Early detection of PPH in all women having caesarean birth							
Quantitative blood loss measurement and monitoring of haemodynamic status	0	1	19	**8 (1)**	**-0.34**	**Yes**	**Appropriate**
Thresholds for triggering action							
Option 1: At least 500 mL with continued bleeding OR clinical signs of haemodynamic instability	1	3	16	**8 (1)**	**-0.13**	**Yes**	**Appropriate**
Option 2: At least 750 mL with continued bleeding OR clinical signs of haemodynamic instability	7	4	9	6 (5)	3.13	No	Uncertain
**Postoperative**							
Early detection of PPH in all women having caesarean birth							
Monitoring of haemodynamic status and quantitative blood loss measurement if feasible	0	2	18	**8 (1)**	**-0.34**	**Yes**	**Appropriate**
Thresholds for triggering action	Not applicable						

Notes: [a]RAND DI: The disagreement index is a continuous scale used to measure the dispersion of experts' ratings, taken as an indicator of the level of agreement; [b]Agreement: A DI < 1 represents an agreement, while a DI ≥ 1 indicates disagreement; [c]Appropriateness: Items are classified as "appropriate" with median ratings in the top (median between 7-9) third and agreement, and as "uncertain" with intermediate median ratings (median between 4-6) or with disagreement.

For peer review only - http://bmjopen.bmj.com/site/about/guidelines.xhtml

Definition of PPH during and after caesarean birth

The experts agreed that a single definition of PPH, regardless of mode of birth (median rating 7.5; DI -5.23). Specifically, they agreed that the definition of PPH during and after caesarean birth should be the same as the definition of PPH related to vaginal birth, to underscore the importance of rapid action to address excessive bleeding.

Intraoperative phase

Early detection of PPH during caesarean birth and thresholds for triggering action

Experts agreed that during caesarean birth, blood loss should be assessed via quantitative measurement, complemented by ongoing monitoring of the woman's haemodynamic status (median rating 8; DI -0.34). Further, quantitative measurement and monitoring should be incorporated into routine practice alongside strategies to prevent PPH. (Box 2). They noted the importance of distinguishing blood from amniotic fluid. This might be achieved by using separate suction canisters or measuring and recording the amount of amniotic fluid within the canister immediately after birth and before delivery of the placenta.

The experts agreed that first response treatment should be triggered if the woman has lost at least 500 mL of blood and still has continued bleeding or if she exhibits clinical signs of haemodynamic instability, whichever occurs first (median rating 8; DI -0.13). Such early action was considered important to prevent severe PPH and associated morbidity, because measurement of blood loss lags actual blood loss. Rapid response has been identified as a critical component of the effectiveness of an early detection and PPH treatment strategy to prevent severe PPH in vaginal births (40). Experts considered that rapid response is particularly important in settings with a high prevalence of anaemia. It was noted that the proposed threshold for triggering action may result in many women receiving first response treatment for PPH. Some experts pointed out that this could diminish providers' responsiveness and recognition of PPH as a serious complication.

Several experts flagged the need for guidance in determining when haemodynamic instability occurs. They noted that physicians are often diligent in recording vital signs, but may not know when to escalate care. Although beyond the scope of this consensus, the provision of guidance to clinicians was acknowledged.

For peer review only - http://bmjopen.bmj.com/site/about/guidelines.xhtml

Box 2. Agreed early detection of PPH during caesarean birth and thresholds for triggering first response in the intraoperative phase

- Early detection of PPH during caesarean birth
  - Quantitative measurement of blood loss
    - Volumetric measurement alone if feasible (able to capture all blood)
    - Volumetric measurement + gravimetric measurement
  - Monitor haemodynamic status
- Thresholds for triggering first response
  - At least 500 mL measured blood loss <u>WITH</u> continued active bleeding
    OR
  - Clinical signs of haemodynamic instability

Additional comments

It is important to separate/distinguish amniotic fluid from blood.

To prevent severe PPH, first response management should be triggered early if there is still continued bleeding, particularly in settings with a high prevalence of anaemia or where unavoidable delays implementing treatment are anticipated.

First response management: intraoperative phase

The agreed first response management is summarised in Box 3. Specifically, the experts agreed clinicians should commence an infusion of oxytocin. If a prophylactic or other oxytocin infusion is already in place, the anaesthetist should quickly maximise the oxytocin dose as increasing uterine tone helps to reduce bleeding from the incision. If atony is diagnosed or the bleeding continues, the anaesthetist should rapidly add in a different uterotonic for treatment. The experts noted that this should occur quickly, rather than waiting to see whether the bleeding is responsive to oxytocin. They also agreed that tranexamic acid (TXA) should be administered as first response treatment, unless the woman had already received TXA within the last 30 minutes. Next, the team should carefully examine the woman to determine the source(s) of bleeding and initiate a cause-specific response. If the bleeding is due to trauma, the surgical team should close the uterus, repair any tears, and attend to the wound. If the bleeding is due to uterine atony, the surgical team should control bleeding mechanically with intra-abdominal uterine massage or massage the exteriorised uterus, as the anaesthetic team manages uterotonic administration, as previously described. Some experts noted that the assessment of atonic PPH may require installing and monitoring under-buttock drapes to assess vaginal blood loss. Experts highlighted that bleeding may be due to a combination of trauma and uterine atony; in such cases, the

For peer review only - http://bmjopen.bmj.com/site/about/guidelines.xhtml

team should take a comprehensive approach. The experts also highlighted the importance of exteriorising and examining the posterior side of the uterus for tears and occult uterine rupture.

Surgical and anaesthetic teams should mobilise to administer the surgical and medical first responses concurrently. Team communication can be challenging and should be practised in drills to develop effective messages that will not alarm women. Teams should immediately call for senior assistance when necessary.

Experts also noted that anaesthetic teams should replace fluids as needed for haemodynamic maintenance, according to the clinical condition and estimated blood loss. Some experts noted that providing guidance on amounts of fluids was too case-specific. However, others stressed that inexperienced clinicians needed concrete guidance to avoid adding too many fluids and inducing fluid overload. Although this type of guidance is beyond the scope of this study, it is a relevant issue that should be addressed.

Experts stressed the importance of ensuring adequate intravenous access (via a wide-bore cannula or a second cannula) early on to enable escalation, given that it can be difficult to establish as a woman loses greater blood volume.

Although outside the scope of this consensus, one expert noted that clinicians need to be thinking about coagulopathy: both as a possible cause of bleeding and as a side-effect of resuscitation efforts, and that it requires specific guidance on appropriate blood products in all settings.

Finally, experts acknowledged that this first response approach is intended to be appropriate for most cases of intraoperative PPH. There may be some cases that, due to quantity and rapidity of blood loss, require an individualised approach. Placental aetiologies, such as placenta previa or accreta, may require specific first response surgical (e.g., lower uterine compression sutures) or mechanical (e.g., internal aortic compression) procedures. While these aetiologies were not specifically targeted in this Delphi process, some first response actions were suggested by experts.

For peer review only - http://bmjopen.bmj.com/site/about/guidelines.xhtml

For peer review only

Box 3. Agreed upon first response treatment for PPH during the intraoperative phase

At least 500 mL measured blood loss WITH ongoing bleeding OR clinical signs of haemodynamic instability:

- If already infusing oxytocin, maximise dose OR add alternative uterotonic. If not already infusing, commence oxytocin infusion.
- TXA (1g in 10 mL IV over 10 min), if not already administered within the last 30 minutes.
- Examine and rapidly initiate cause-specific response:
  - If from incision or surgical trauma: Rapid haemostasis: close uterus, repair tears, attend to the wound.
  - If atony/placental cause: Uterotonics (as above) and control bleeding mechanically with intra-abdominal uterine massage or exteriorise the uterus and massage.

Additional comments

All blood loss may not be immediately obvious. Examine the posterior side of the uterus for cervical tears and occult uterine rupture, and install and monitor an under-buttock drape to assess vaginal blood loss.

Medical and surgical first responses should be administered concurrently, and effective team communication is key.

Replace IV fluids as needed for haemodynamic maintenance, according to the clinical condition, estimated blood loss, and local protocols.

TXA should be administered as first response treatment, unless the woman has already received TXA for PPH prevention or treatment within the last 30 minutes. Up to 2 doses of TXA, at least 30 minutes apart may be administered.

If atony is diagnosed or the bleeding continues after the oxytocin dose has been maximised, the anaesthetists should rapidly add in a different uterotonic for treatment.

Due to quantity and rapidity of blood loss, there may be some cases that require an individualised approach.

Postoperative phase

Early detection of PPH after caesarean birth and thresholds for triggering action

Postoperative detection of PPH based on monitoring blood loss can be misleading because of internal bleeding. Thus, during this phase, experts agreed (median rating 8, DI -0.34) that blood loss should be assessed primarily through frequent monitoring of women's haemodynamic status (when possible, at least every 15 minutes for the first 2 hours) and clinical signs and symptoms of internal bleeding (e.g., assessment of fundal height) (Box 4). In addition, if the assessment of postoperative vaginal blood loss is feasible, either by quantitative measurement or estimation (e.g., counting and weighting pads), it should

For peer review only - http://bmjopen.bmj.com/site/about/guidelines.xhtml

be performed. Here, the experts noted that clinical teams should not rely on vital signs alone, as vital signs' disturbances can lag behind other clinical indications of haemorrhage. Some experts noted that postoperative monitoring for at least 30 minutes after caesarean birth should occur in a designated recovery area to ensure the woman's safety. If internal bleeding is suspected, experts recommended an urgent ultrasound assessment if available. Experts agreed that, when possible, measured postoperative blood loss should be added to the quantified intraoperative blood loss, though they acknowledged that this may be challenging in some settings. Experts noted that cumulative intraoperative and postoperative blood loss, together with a woman's haemodynamic status, can help adjust the frequency and characteristics of postoperative monitoring and thresholds for action. For example, a woman who experienced substantial blood loss intraoperatively may require more frequent monitoring than the baseline every 15 minutes.

Although it is beyond the scope of this study, the experts acknowledged that providing guidance on haemodynamic parameters cut-off points for postoperative thresholds to trigger treatment will help clinicians act more quickly. Several experts raised the possibility of using the Shock Index (heart rate divided by systolic blood pressure; OSI) as a clinical decision support tool to simplify the decision of when to act, given that it has been used in some settings, including low-resource settings.

For peer review only - http://bmjopen.bmj.com/site/about/guidelines.xhtml

Box 4. Agreed early detection of postoperative PPH and thresholds for triggering first response

- Early detection of PPH
  - Frequent monitoring of haemodynamic status (at least every 15 minutes for the first 2 hours)
    - Heart rate
    - Blood pressure
    - Shock index
    - Clinical signs/symptoms suspicious of internal bleeding
  - Quantitative blood loss assessment, if feasible
    - Measured or estimated postoperative blood loss (when possible, added to quantified intraoperative blood loss)
- Thresholds for triggering first response management
  - Clinical signs and symptoms of haemodynamic instability, in accordance with local protocols

---

Additional comments

Relying on postoperative blood loss alone can underestimate internal bleeding. Increase vigilance and assess hemodynamic status frequently.

Early detection of postoperative PPH should mainly rely on frequent monitoring of haemodynamic status and clinical signs and symptoms of internal bleeding. If assessment of postoperative vaginal blood loss is feasible, either by quantitative measurement or estimation (e.g., counting pads), it should be performed.

When possible, assessed postoperative blood loss should be added to the quantified intraoperative blood loss.

The cumulative intraoperative and postoperative blood loss, together with a woman's haemodynamic status, may better determine the frequency and characteristics of postoperative monitoring and thresholds for action.

Hemodynamic parameter thresholds for vital signs and Obstetric Shock Index to trigger treatment are not yet agreed upon

Postoperative phase: First response management

The experts noted that the follow-on postoperative treatment approach may vary substantially according to many factors, including the woman's baseline risk, anaemia, whether intraoperative PPH occurred, the woman's postoperative haemodynamic status, and clinical signs and symptoms of internal bleeding (e.g., assessment of fundal height; if available, ultrasound, paracentesis). Until further evidence is available, experts recommended that local protocols be developed that consider these factors, rather than relying on a common postoperative first response approach for all cases and settings.

Experts' final comments

For peer review only - http://bmjopen.bmj.com/site/about/guidelines.xhtml

The experts recognised that detection methods and first response interventions for PPH are essential for the care of all women having a caesarean birth, regardless of their risk status. However, women at high risk of developing PPH may require additional specialised monitoring and care.

In addition, given that PPH can arise intra- or postoperatively for any woman, strategies for early detection of PPH should be incorporated into routine practice alongside PPH prevention and risk assessment.

Finally, experts highlighted two cross-cutting remarks regarding PPH during and after caesarean birth. First, good surgical practices, as recommended by the WHO Guidelines for Safe Surgery, should be followed to prepare for, perform, and follow-up caesarean births (55). The routine use of WHO surgical safety checklists has proven beneficial in reducing perioperative complications (56). See Supplementary File S6 for more details. Second, it was noted that teamwork, communication, and cooperation are critical. Effectively implementing the early detection and first response interventions described will require training, supportive supervision, monitoring, and evaluation.

DISCUSSION

Main findings

Expert consensus on optimal approaches for detecting and managing PPH during and after caesarean birth was developed among an international panel. Through two systematic reviews and a three-round modified Delphi process, consensus was reached for (a) using a single definition for PPH, regardless of the mode of birth, (b) early detection of PPH during caesarean birth and thresholds to initiate treatment in the intraoperative phase, (c) clinical interventions for first response to intraoperative PPH, and (d) early detection of PPH after caesarean birth and threshold to initiate treatment in the postoperative phase. First response treatment in the postoperative phase was determined to require an individualised approach.

Strengths and limitations

Study strengths include the use of a rigorous and systematic process to identify and synthesise PPH evidence in the literature. We conducted in-depth systematic reviews with detailed quality appraisals to ensure that we used only high-quality evidence to identify approaches for PPH detection and management interventions. The selection of the expert panellists ensured a wide range of perspectives,

For peer review only - http://bmjopen.bmj.com/site/about/guidelines.xhtml

to enhance the utility and applicability of this consensus to a wide range of clinical settings. There was a low rate of loss to follow-up. The first two rounds of the modified-Delphi process were blinded, to avoid social acceptability bias, and the hybrid meeting was facilitated by members of the Steering Group, to ensure that all panellists had equal opportunity to contribute to the discussion. The staged modified-Delphi process allowed ample time for discussion and input, and experts provided additional comments to refine the final statements for clarity and accuracy.

Limitations included a dearth of quality evidence on PPH related to caesarean birth. Despite ample evidence on PPH during and after vaginal birth, there is far less published evidence on caesarean birth. Often, the experts had to extrapolate from evidence on interventions recommended for PPH in vaginal birth and make decisions based on their experiences, expert opinions, and best practices, rather than evidence from comparative research. In some cases, this led to omitting interventions that might be useful for early detection or first-response management because there was no rigorous evidence available. It is also a limitation that, given the highly technical content, we did not include recipients of these interventions, or their representatives, among the panellists.

Additionally, since this systematic review of guidelines was conducted, three updated PPH guidelines have been published (57–59). None of these guidelines are specific to PPH at caesarean birth, though all contain some guidance relevant to PPH during or after caesarean birth. The recommendations within these guidelines generally align with previously published guidance included in our study, with a few notable exceptions. The revised 2023 FIGO PPH guideline recommends the use of the Obstetric Shock Index (with a threshold of ≥0.9 triggering first-response treatment), together with the rule of 30, while acknowledging that “the association between shock parameters and advanced treatment modalities in severe PPH has yet to be reported” (60). In the updated CMQCC Obstetric Hemorrhage Toolkit, greater emphasis is placed on assessing for concealed haemorrhage. The guideline recommends using a combination of clinical signs of hypovolemia, the SI, and Early Warning Score to enable earlier postoperative PPH detection (58). The Royal College of Physicians of Ireland guideline suggests that prophylactic tranexamic acid administration be considered in women at high PPH risk (59). The timing of our study prevented us from incorporating these revisions into our systematic review.

Interpretation

For peer review only - http://bmjopen.bmj.com/site/about/guidelines.xhtml

This expert consensus aligns with the recent expert consensus developed by the African Perioperative Research Group (APORG) Caesarean Delivery Haemorrhage Group (61) for clinicians working in Africa. The APORG expert consensus had a broader scope, encompassing antenatal and perioperative prevention, preparedness, first response, refractory treatment interventions, and community- and health system-level indirect interventions. This present expert consensus focuses only on early detection and first response, including specific thresholds for triggering action.

With rates of caesarean birth rising globally, particularly in middle-income countries (6), this research is timely and crucial. International initiatives are underway to end preventable deaths due to PPH, such as the Roadmap to Combat Postpartum Haemorrhage between 2023 and 2030 (62), and the Pan American Health Organization's Zero Maternal Deaths by Hemorrhage campaign (63). The present expert consensus on early detection and first-response treatment for PPH at caesarean birth adds to existing efforts by clearly delineating how interventions need to be tailored for the context of caesarean birth. This consultation represents an important first step toward developing standardised strategies for reducing morbidity and mortality related to PPH during and after caesarean birth. Determining how best to implement these standardised strategies is a critical next step.

Insights from implementation science suggest that defining evidence-based interventions is a necessary but insufficient step towards changing clinical practice (64). Establishing implementation approaches is believed to increase uptake and fidelity of evidence-based interventions (65). Clinical bundles are one implementation approach that has gained traction in recent years (57,66–69). Global evidence suggests that clinical bundles are a powerful implementation approach for early detection and first response for PPH after vaginal birth (68,69). However, it is unclear whether a bundle is the most appropriate implementation approach for PPH during and after caesarean birth. Bundles require a set of interventions to be administered together, but the administration of some of the clinical interventions outlined here may depend on what occurs during surgery and what other interventions may already have been administered. As such, other implementation approaches, such as algorithms, protocols, checklists, or activation of haemorrhage codes, might be more appropriate (70,71). Defining the optimal implementation approach for early detection and first response management of PPH during and after caesarean birth still remains to be completed. Conducting the necessary research to answer this question should be an immediate next step.

For peer review only - http://bmjopen.bmj.com/site/about/guidelines.xhtml

In addition, efforts should be pursued to agree on standardised approaches for the management of refractory PPH during and after caesarean birth. Importantly, these standardised approaches should encompass both the specific interventions used to manage refractory PPH, appropriate fluid and blood product management protocols, and the implementation strategies to support their uptake and sustainability. Standardised approaches will need to be applicable to various settings, including those with limited access to laboratories, crossmatched blood and blood products, expensive devices, and medical specialists. This consensus focuses mainly on obstetric interventions, although haemodynamic resuscitation and obstetric measures to stop haemorrhage should be applied simultaneously. Recommendations for haemostatic resuscitation, including hemodynamic, coagulopathy, transfusion, and intraoperative cell salvage (72), will be part of the forthcoming WHO/FIGO/ICM consolidated PPH guidelines in 2024 (F Althabe personal communication).

CONCLUSION

This expert consensus proposes strategies for early detection and first response to PPH during and after caesarean birth. Future research should determine how best to implement these strategies and evaluate the effectiveness of the proposed implementation approach. Such research should be conducted soon, so that the approaches and interventions proposed here can rapidly be operationalised and institutionalised to contribute to the global efforts to reduce maternal death and disability.

CONTRIBUTION TO AUTHORSHIP: SM, FA, AC, IG, and OTO conceived the idea. VP developed the protocol with input from SM and FA. SM, FA, CRW, and VP coordinated the project with input from IG and AC. FM, AL, VO, AB, CRW, and VP conducted the systematic review. OTO, IG, AC, EA, SA, NA, FAA, BC, CDT, SD, AD, MFE, CE, SF, HG, HGJ, CH, TL, PL, EM, IN, MJ, HT, PT, ZQ, CS, JV, AW, FA, SM, CRW, and VP participated in the consensus and provided expert input at various stages of the project. Data were analysed by CRW and VP with input from SM and FA. SM, FA, CRW, and VP wrote the manuscript with input from MW and all co-authors. This manuscript represents the views of the authors and not the views of the World Health Organization or the UNDP/UNFPA/UNICEF/WHO/World Bank Special Programme of Research, Development and Research Training in Human Reproduction (HRP).

ETHICAL CONSIDERATIONS: Authors confirm that this exercise was conducted according to relevant guidelines and regulations with respect to the Helsinki Declaration. Experts were advised that

For peer review only - http://bmjopen.bmj.com/site/about/guidelines.xhtml

participation was voluntary and indicated informed consent. Given the consultative nature of the exercise which involved consenting experts in their work capacity, ethical clearance was not required.

FUNDING: This study was funded by the Bill & Melinda Gates Foundation (INV-001393) through a grant to the University of Birmingham, United Kingdom, and the UNDP/UNFPA/UNICEF/WHO/World Bank Special Programme of Research, Development and Research Training in Human Reproduction (HRP), a cosponsored program executed by the WHO.

DATA AVAILABILITY: Extensive data are presented in the supplementary materials. Given the small sample size, datasets will be available upon reasonable request.

CONSENT FOR PUBLICATION: All authors consented to publication.

COMPETING INTERESTS: Disclosure forms provided by the authors are available with the full text of this article.

ACKNOWLEDGMENTS: We would like to acknowledge the contribution of Bill & Melinda Gates Foundation officers for conceiving the research question and supporting the conduct of this study; the contribution of Zhitong Yu in preparing the consensus building process and the administrative support of Chastine Sebolino and Maria Harmitton Oliveto.

This article reflects the views of the named authors, and not the views of their organisations, or the views of UNDP-UNFPA-UNICEF-WHO-World Bank Special Programme of Research, Development and Research Training in Human Reproduction (HRP) or World Health Organization.

For peer review only - http://bmjopen.bmj.com/site/about/guidelines.xhtml

REFERENCES

1. Say L, Chou D, Gemmill A, Tunçalp Ö, Moller A-B, Daniels J, et al. Global causes of maternal death: a WHO systematic analysis. Lancet Glob Health [Internet]. 2014 Jun;2(6):e323-33. Available from: http://dx.doi.org/10.1016/S2214-109X(14)70227-X

2. World Health Organization(WHO). WHO recommendations for the prevention and treatment of postpartum haemorrhage. Genève, Switzerland: World Health Organization; 2012. 44 p.

3. Butwick AJ, Ramachandran B, Hegde P, Riley ET, El-Sayed YY, Nelson LM. Risk factors for severe postpartum hemorrhage after cesarean delivery: Case-control studies. Anesth Analg [Internet]. 2017 Aug;125(2):523–32. Available from: http://dx.doi.org/10.1213/ANE.0000000000001962

4. Betran AP, Ye J, Moller A-B, Souza JP, Zhang J. Trends and projections of caesarean section rates: global and regional estimates. BMJ Glob Health [Internet]. 2021 Jun;6(6):e005671. Available from: http://dx.doi.org/10.1136/bmjgh-2021-005671

5. Sobhy S, Arroyo-Manzano D, Murugesu N, Karthikeyan G, Kumar V, Kaur I, et al. Maternal and perinatal mortality and complications associated with caesarean section in low-income and middle-income countries: a systematic review and meta-analysis. Lancet [Internet]. 2019 May 11;393(10184):1973–82. Available from: http://dx.doi.org/10.1016/S0140-6736(18)32386-9

6. Al-Zirqi I, Vangen S, Forsén L, Stray-Pedersen B. Effects of onset of labor and mode of delivery on severe postpartum hemorrhage. Obstet Anesth Dig [Internet]. 2010 Sep;30(3):150–1. Available from: http://dx.doi.org/10.1097/01.aoa.0000386819.15311.cb

7. Miller S, Abalos E, Chamillard M, Ciapponi A, Colaci D, Comandé D, et al. Beyond too little, too late and too much, too soon: a pathway towards evidence-based, respectful maternity care worldwide. Lancet [Internet]. 2016 Oct;388(10056):2176–92. Available from: http://dx.doi.org/10.1016/s0140-6736(16)31472-6

8. Chestnut DH. Postpartum hemorrhage – update on problems of definitions and diagnosis. Yearb Anesthesiol Pain Manag [Internet]. 2012 Jan;2012:322–3. Available from: http://dx.doi.org/10.1016/j.yane.2011.07.002

9. Widmer M, Piaggio G, Nguyen TMH, Osoti A, Owa OO, Misra S, et al. Heat-stable carbetocin versus oxytocin to prevent hemorrhage after vaginal birth. N Engl J Med [Internet]. 2018 Jun 27;(NEJMoa1805489). Available from: http://dx.doi.org/10.1056/nejmoa1805489

10. Gallos I, Devall A, Martin J, Middleton L, Beeson L, Galadanci H, et al. Randomised trial of early detection and treatment of postpartum hemorrhage. N Engl J Med [Internet]. 2023 May 9; Available from: http://dx.doi.org/10.1056/NEJMoa2303966

11. Shields LE, Wiesner S, Fulton J, Pelletreau B. Comprehensive maternal hemorrhage protocols reduce the use of blood products and improve patient safety. Am J Obstet Gynecol [Internet]. 2015 Mar;212(3):272–80. Available from: http://dx.doi.org/10.1016/j.ajog.2014.07.012

For peer review only - http://bmjopen.bmj.com/site/about/guidelines.xhtml

12. Main EK, Cape V, Abreo A, Vasher J, Woods A, Carpenter A, et al. Reduction of severe maternal morbidity from hemorrhage using a state perinatal quality collaborative. Am J Obstet Gynecol [Internet]. 2017 Mar;216(3):298.e1-298.e11. Available from: http://dx.doi.org/10.1016/j.ajog.2017.01.017

13. World Health Organization. WHO Recommendation on Tranexamic Acid for the Treatment of Postpartum Haemorrhage. https://www.who.int/publications/i/item/9789241548502; 2017.

14. World Health Organization. WHO recommendation on uterine balloon tamponade for the treatment of postpartum haemorrhage [Internet]. Genève, Switzerland: World Health Organization; 2021 [cited 2023 Apr 30]. 85 p. Available from: https://apps.who.int/iris/handle/10665/340796

15. World Health Organization. Managing complications in pregnancy and childbirth. 2nd ed. Genève, Switzerland: World Health Organization; 2017. 488 p.

16. Althabe F, Therrien MNS, Pingray V, Hermida J, Gülmezoglu AM, Armbruster D, et al. Postpartum hemorrhage care bundles to improve adherence to guidelines: A WHO technical consultation. Int J Gynaecol Obstet [Internet]. 2020 Mar;148(3):290–9. Available from: http://dx.doi.org/10.1002/ijgo.13028

17. Brouwers MC, Kho ME, Browman GP, Burgers JS, Cluzeau F, Feder G, et al. AGREE II: advancing guideline development, reporting and evaluation in health care. CMAJ [Internet]. 2010 Dec 14;182(18):E839-42. Available from: http://dx.doi.org/10.1503/cmaj.090449

18. Shea BJ, Reeves BC, Wells G, Thuku M, Hamel C, Moran J, et al. AMSTAR 2: a critical appraisal tool for systematic reviews that include randomised or non-randomised studies of healthcare interventions, or both. BMJ [Internet]. 2017 Sep 21;j4008. Available from: http://dx.doi.org/10.1136/bmj.j4008

19. Hofer S, Blaha J, Collins PW, Ducloy-Bouthors A-S, Guasch E, Labate F, et al. Haemostatic support in postpartum haemorrhage. Eur J Anaesthesiol [Internet]. 2023 Jan [cited 2024 Jan 10];40(1):29–38. Available from: https://journals.lww.com/ejanaesthesiology/fulltext/2023/01000/haemostatic_support_in_postpartum_haemorrhage__a.5.aspx

20. Fitch K, Bernstein SJ, Aguilar MD. The rand/UCLA appropriateness method user's manual. Santa Monica, CA: RAND; 1999. 133 p.

21. NICE. Overview | Caesarean birth | Guidance | NICE. [cited 2023 Apr 30]; Available from: https://www.nice.org.uk/guidance/ng192

22. ACOG. Quantitative blood loss in obstetric hemorrhage. Obstet Gynecol [Internet]. 2019 Dec;134(6):e150–6. Available from: http://dx.doi.org/10.1097/aog.0000000000003564

23. Leduc D, Senikas V, Lalonde AB. No. 235-active management of the third stage of labour: Prevention and treatment of postpartum hemorrhage. J Obstet Gynaecol Can [Internet]. 2018 Dec;40(12):e841–55. Available from: http://dx.doi.org/10.1016/j.jogc.2018.09.024

24. RANZCOG Women's Health Committee. Management of Postpartum Haemorrhage [Internet]. RANZCOG; 2016. Available from: chrome-

For peer review only - http://bmjopen.bmj.com/site/about/guidelines.xhtml

extension://efaidnbmnnnibpcajpcglclefindmkaj/https://ranzcog.edu.au/wp-content/uploads/2022/05/Management-of-Postpartum-Haemorrhage-PPH.pdf

25. Queensland Clinical Guidelines. Postpartum haemorrhage Guideline. chrome-extension://efaidnbmnnnibpcajpcglclefindmkaj/https://www.health.qld.gov.au/__data/assets/pdf_file/0015/140136/g-pph.pdf; 2021.

26. ACOG. Postpartum Hemorrhage [Internet]. [cited 2023 May 1]. Available from: https://www.acog.org/clinical/clinical-guidance/practice-bulletin/articles/2017/10/postpartum-hemorrhage

27. Muñoz M, Stensballe J, Ducloy-Bouthors A-S, Bonnet M-P, De Robertis E, Fornet I, et al. Patient blood management in obstetrics: prevention and treatment of postpartum haemorrhage. A NATA consensus statement. Blood Transfus [Internet]. 2019 Mar;17(2):112–36. Available from: http://dx.doi.org/10.2450/2019.0245-18

28. Sentilhes L, Vayssière C, Deneux-Tharaux C, Aya AG, Bayoumeu F, Bonnet M-P, et al. Postpartum hemorrhage: guidelines for clinical practice from the French College of Gynaecologists and Obstetricians (CNGOF): in collaboration with the French Society of Anesthesiology and Intensive Care (SFAR). Eur J Obstet Gynecol Reprod Biol [Internet]. 2016 Mar;198:12–21. Available from: http://dx.doi.org/10.1016/j.ejogrb.2015.12.012

29. Schlembach D, Helmer H, Henrich W, von Heymann C, Kainer F, Korte W, et al. Peripartum Haemorrhage, Diagnosis and Therapy. Guideline of the DGGG, OEGGG and SGGG (S2k Level, AWMF Registry No. 015/063, March 2016). Geburtshilfe Frauenheilkd [Internet]. 2018 Apr;78(04):382–99. Available from: http://dx.doi.org/10.1055/a-0582-0122

30. RCOG. Prevention and management of postpartum haemorrhage (green-top guideline no. 52) [Internet]. RCOG. [cited 2023 May 1]. Available from: https://www.rcog.org.uk/guidance/browse-all-guidance/green-top-guidelines/prevention-and-management-of-postpartum-haemorrhage-green-top-guideline-no-52/

31. Institute of Obstetricians and Gynaecologists. Royal College of Physicians of Ireland and Directorate of Strategy and Clinical Programmes. Health Service Executive. CLINICAL PRACTICE GUIDELINE: PREVENTION AND MANAGEMENT OF PRIMARY POSTPARTUM HAEMORRHAGE [Internet]. 2014 Jun. Available from: https://www.hse.ie/eng/about/who/acute-hospitals-division/woman-infants/clinical-guidelines/

32. Lalonde A, International Federation of Gynecology and Obstetrics. Prevention and treatment of postpartum hemorrhage in low-resource settings. Int J Gynaecol Obstet [Internet]. 2012 May;117(2):108–18. Available from: http://dx.doi.org/10.1016/j.ijgo.2012.03.001

33. Lyndon A, Lagrew D, Shields L, Main E, Cape V. Improving Health Care Response to Obstetric Hemorrhage. California Department of Public Health; Maternal, Child and Adolescent Health Division; 2015.

34. Minakami H, Maeda T, Fujii T, Hamada H, Iitsuka Y, Itakura A, et al. Guidelines for obstetrical practice in Japan: Japan Society of Obstetrics and Gynecology (JSOG) and Japan Association of Obstetricians and Gynecologists (JAOG) 2014 edition. J Obstet Gynaecol Res [Internet]. 2014 Jun;40(6):1469–99. Available from: http://dx.doi.org/10.1111/jog.12419

For peer review only

For peer review only - http://bmjopen.bmj.com/site/about/guidelines.xhtml

35. Shakur H, Beaumont D, Pavord S, Gayet-Ageron A, Ker K, Mousa HA. Antifibrinolytic drugs for treating primary postpartum haemorrhage. Cochrane Database Syst Rev [Internet]. 2018 Feb 20;2:CD012964. Available from: http://dx.doi.org/10.1002/14651858.CD012964

36. Kellie FJ, Wandabwa JN, Mousa HA, Weeks AD. Mechanical and surgical interventions for treating primary postpartum haemorrhage. Cochrane Database Syst Rev [Internet]. 2020 Jul 1;7(7):CD013663. Available from: http://dx.doi.org/10.1002/14651858.CD013663

37. Parry Smith WR, Papadopoulou A, Thomas E, Tobias A, Price MJ, Meher S, et al. Uterotonic agents for first-line treatment of postpartum haemorrhage: a network meta-analysis. Cochrane Libr [Internet]. 2020 Nov 24;2020(11). Available from: http://dx.doi.org/10.1002/14651858.cd012754.pub2

38. Gallos ID, Williams HM, Price MJ, Merriel A, Gee H, Lissauer D, et al. Uterotonic agents for preventing postpartum haemorrhage: a network meta-analysis. Cochrane Libr [Internet]. 2018 Apr 25; Available from: http://dx.doi.org/10.1002/14651858.cd011689.pub2

39. Andrikopoulou M, D'Alton ME. Postpartum hemorrhage: early identification challenges. Semin Perinatol [Internet]. 2019 Feb;43(1):11–7. Available from: http://dx.doi.org/10.1053/j.semperi.2018.11.003

40. Atukunda EC, Mugyenyi GR, Obua C, Atuhumuza EB, Musinguzi N, Tornes YF, et al. Measuring post-partum haemorrhage in low-resource settings: The diagnostic validity of weighed blood loss versus quantitative changes in hemoglobin. PLoS One [Internet]. 2016 Apr 6;11(4):e0152408. Available from: http://dx.doi.org/10.1371/journal.pone.0152408

41. Bell SF, Watkins A, John M, Macgillivray E, Kitchen TL, James D, et al. Incidence of postpartum haemorrhage defined by quantitative blood loss measurement: a national cohort. BMC Pregnancy Childbirth [Internet]. 2020 May 6;20(1):271. Available from: http://dx.doi.org/10.1186/s12884-020-02971-3

42. Diaz V, Abalos E, Carroli G. Methods for blood loss estimation after vaginal birth. Cochrane Database Syst Rev [Internet]. 2018 Sep 13;9(9):CD010980. Available from: http://dx.doi.org/10.1002/14651858.CD010980.pub2

43. Hancock A, Weeks AD, Lavender DT. Is accurate and reliable blood loss estimation the "crucial step" in early detection of postpartum haemorrhage: an integrative review of the literature. BMC Pregnancy Childbirth [Internet]. 2015 Dec;15(1). Available from: http://dx.doi.org/10.1186/s12884-015-0653-6

44. Larsson C, Saltvedt S, Wiklund I, Pahlen S, Andolf E. Estimation of blood loss after cesarean section and vaginal delivery has low validity with a tendency to exaggeration. Acta Obstet Gynecol Scand [Internet]. 2006;85(12):1448–52. Available from: http://dx.doi.org/10.1080/00016340600985032

45. Rath WH. Postpartum hemorrhage update on problems of definitions and diagnosis. Obstet Anesth Dig [Internet]. 2012 Jun;32(2):76–7. Available from: http://dx.doi.org/10.1097/01.aoa.0000414055.79309.d6

46. Ambardekar S, Shochet T, Bracken H, Coyaji K, Winikoff B. Calibrated delivery drape versus indirect gravimetric technique for the measurement of blood loss after delivery: a randomised trial. BMC

For peer review only - http://bmjopen.bmj.com/site/about/guidelines.xhtml

Pregnancy Childbirth [Internet]. 2014 Aug 15;14(1):276. Available from: http://dx.doi.org/10.1186/1471-2393-14-276

47. Buckland SS, Homer CSE. Estimating blood loss after birth: using simulated clinical examples. Women Birth [Internet]. 2007 Jun;20(2):85–8. Available from: http://dx.doi.org/10.1016/j.wombi.2007.01.001

48. Lertbunnaphong T, Lapthanapat N, Leetheeragul J, Hakularb P, Ownon A. Postpartum blood loss: visual estimation versus objective quantification with a novel birthing drape. Singapore Med J [Internet]. 2016 Jun;57(6):325–8. Available from: http://dx.doi.org/10.11622/smedj.2016107

49. Mhyre JM, D'Oria R, Hameed AB, Lappen JR, Holley SL, Hunter SK, et al. The maternal early warning criteria: a proposal from the national partnership for maternal safety. J Obstet Gynecol Neonatal Nurs [Internet]. 2014 Nov;43(6):771–9. Available from: http://dx.doi.org/10.1111/1552-6909.12504

50. Patel A, Goudar SS, Geller SE, Kodkany BS, Edlavitch SA, Wagh K, et al. Drape estimation vs. visual assessment for estimating postpartum hemorrhage. Int J Gynaecol Obstet [Internet]. 2006 Jun;93(3):220–4. Available from: http://dx.doi.org/10.1016/j.ijgo.2006.02.014

51. Schorn MN. Measurement of blood loss: review of the literature. J Midwifery Womens Health [Internet]. 2010 Jan;55(1):20–7. Available from: http://dx.doi.org/10.1016/j.jmwh.2009.02.014

52. Shields LE, Wiesner S, Klein C, Pelletreau B, Hedriana HL. Use of Maternal Early Warning Trigger tool reduces maternal morbidity. Am J Obstet Gynecol [Internet]. 2016 Apr;214(4):527.e1-527.e6. Available from: http://dx.doi.org/10.1016/j.ajog.2016.01.154

53. Umar A, Ameh CA, Muriithi F, Mathai M. Early warning systems in obstetrics: A systematic literature review. PLoS One [Internet]. 2019 May 31;14(5):e0217864. Available from: http://dx.doi.org/10.1371/journal.pone.0217864

54. Zhang W-H, Deneux-Tharaux C, Brocklehurst P, Juszczak E, Joslin M, Alexander S, et al. Effect of a collector bag for measurement of postpartum blood loss after vaginal delivery: cluster randomised trial in 13 European countries. BMJ [Internet]. 2010 Feb 1;340(feb01 1):c293. Available from: http://dx.doi.org/10.1136/bmj.c293

55. World Health Organization. Implementation manual: WHO surgical safety checklist (first edition) [Internet]. World Health Organization; 2008 [cited 2023 May 30]. Report No.: WHO/IER/PSP/2008.05. Available from: https://apps.who.int/iris/handle/10665/70046

56. Bergs J, Hellings J, Cleemput I, Zurel Ö, De Troyer V, Van Hiel M, et al. Systematic review and meta-analysis of the effect of the World Health Organization surgical safety checklist on postoperative complications. Br J Surg [Internet]. 2014 Feb;101(3):150–8. Available from: http://dx.doi.org/10.1002/bjs.9381

57. Escobar MF, Nassar AH, Theron G, Barnea ER, Nicholson W, Ramasauskaite D, et al. FIGO recommendations on the management of postpartum hemorrhage 2022. Int J Gynaecol Obstet [Internet]. 2022 Mar;157 Suppl 1(S1):3–50. Available from: http://dx.doi.org/10.1002/ijgo.14116

For peer review only - http://bmjopen.bmj.com/site/about/guidelines.xhtml

58. Lagrew D, McNulty J, Sakowski C, Cape V, McCormick E, Morton CH. Improving Health Care Response to Obstetric Hemorrhage, a California Maternal Quality Care Collaborative Toolkit. 2022; Available from: https://www.cmqcc.org/

59. Byrne B, Spring A, Barrett N, Power J, Mckernan J, Brophy D, et al. Ni Ainle F. National Clinical Practice Guideline: Prevention and Management of Primary Postpartum Haemorrhage. National Women and Infants Health Programme and The Institute of Obstetricians and Gynaecologists. 2022.

60. Morris JL, Winikoff B, Dabash R, Weeks A, Faundes A, Gemzell-Danielsson K, et al. FIGO's updated recommendations for misoprostol used alone in gynecology and obstetrics. Int J Gynaecol Obstet [Internet]. 2017 Sep;138(3):363–6. Available from: http://dx.doi.org/10.1002/ijgo.12181

61. APORG Caesarean Delivery Haemorrhage Group. Identifying interventions to reduce peripartum haemorrhage associated with caesarean delivery in Africa: A Delphi consensus study. PLOS Glob Public Health [Internet]. 2022 Aug 31;2(8):e0000455. Available from: http://dx.doi.org/10.1371/journal.pgph.0000455

62. A Roadmap to combat postpartum haemorrhage between 2023 and 2030. Geneva: World Health Organization. 2023.

63. Zero maternal deaths by hemorrhage [Internet]. [cited 2023 May 15]. Available from: https://www.paho.org/en/zero-maternal-deaths-hemorrhage

64. Bauer MS, Kirchner J. Implementation science: What is it and why should I care? Psychiatry Res [Internet]. 2020 Jan;283(112376):112376. Available from: http://dx.doi.org/10.1016/j.psychres.2019.04.025

65. Carroll C, Patterson M, Wood S, Booth A, Rick J, Balain S. A conceptual framework for implementation fidelity. Implement Sci [Internet]. 2007 Nov 30;2(1):40. Available from: http://dx.doi.org/10.1186/1748-5908-2-40

66. Main EK, Goffman D, Scavone BM, Low LK, Bingham D, Fontaine PL, et al. National Partnership for Maternal Safety consensus bundle on obstetric hemorrhage. J Midwifery Womens Health [Internet]. 2015 Jul;60(4):458–64. Available from: http://dx.doi.org/10.1111/jmwh.12345

67. Main EK, Goffman D, Scavone BM, Low LK, Bingham D, Fontaine PL, et al. National Partnership for Maternal Safety: consensus bundle on obstetric hemorrhage. Anesth Analg [Internet]. 2015 Jul;121(1):142–8. Available from: http://dx.doi.org/10.1097/AOG.0000000000000869

68. D'Alton ME, Friedman AM, Smiley RM, Montgomery DM, Paidas MJ, D'Oria R, et al. National Partnership for Maternal Safety: Consensus bundle on venous thromboembolism. J Obstet Gynecol Neonatal Nurs [Internet]. 2016 Sep;45(5):706–17. Available from: http://dx.doi.org/10.1016/j.jogn.2016.07.001

69. Levy MM, Rhodes A, Phillips GS, Townsend SR, Schorr CA, Beale R, et al. Surviving Sepsis Campaign: association between performance metrics and outcomes in a 7.5-year study. Crit Care Med [Internet]. 2015 Jan;43(1):3–12. Available from: http://dx.doi.org/10.1097/CCM.0000000000000723

For peer review only - http://bmjopen.bmj.com/site/about/guidelines.xhtml

70. Margolis CZ. Uses of clinical algorithms. JAMA [Internet]. 1983 Feb 4;249(5):627–32. Available from: http://dx.doi.org/10.1001/jama.249.5.627

71. Arora KS, Shields LE, Grobman WA, D'Alton ME, Lappen JR, Mercer BM. Triggers, bundles, protocols, and checklists--what every maternal care provider needs to know. Am J Obstet Gynecol [Internet]. 2016 Apr;214(4):444–51. Available from: http://dx.doi.org/10.1016/j.ajog.2015.10.011

72. Iyer NS, Khanuja K, Roman A, Al-Kouatly HB. Use of cell salvage at the time of cesarean delivery: A meta-analysis of randomised controlled trials. Am J Obstet Gynecol MFM [Internet]. 2023 Dec 16;(101257):101257. Available from: https://www.sciencedirect.com/science/article/pii/S2589933323003993

For peer review only

Figure Legends

Figure 1 - Technical consultation flowchart

For peer review only - http://bmjopen.bmj.com/site/about/guidelines.xhtml

Figure 1. Technical consultation flowchart

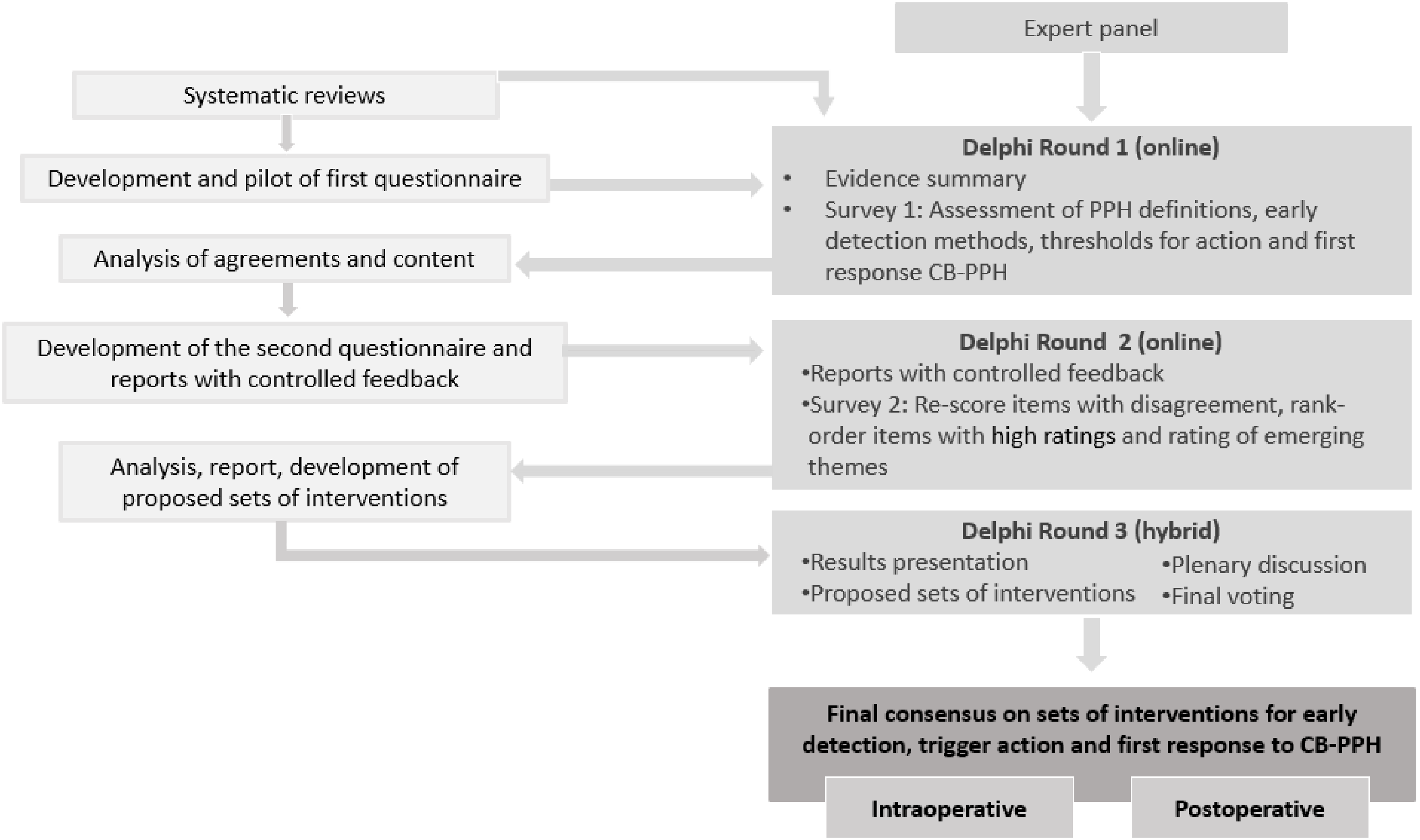

Strategies for optimising early detection and obstetric first response management of postpartum haemorrhage at caesarean birth: A modified Delphi-based international expert consensus

SUPPLEMENTARY MATERIALS

Supplementary Tables ... 2

Table S1. Definitions of PPH and severe PPH used in clinical guidelines ... 2

Table S2. Synthesis of evidence and considerations regarding approaches to detect intraoperative and postoperative caesarean PPH and haemodynamic instability ... 3

Table S3. Synthesis of evidence on thresholds for triggering action on PPH during and after caesarean birth ... 5

Table S4. Medical Interventions, manoeuvres, and procedures for PPH recommended by WHO compared to recommendations in other PPH guidelines and systematic reviews ... 7

Table S5. First and second round ratings and agreement for early detection methods for intraoperative and postoperative CB-PPH ... 11

Table S6. First round ratings and agreement on threshold to initiate treatment for intraoperative CB PPH ... 12

Table S7. First round ratings and agreement for first response interventions for managing intraoperative and postoperative CB-PPH ... 14

Table S8. Second round ratings and agreement for first response interventions for managing intraoperative and postoperative CB-PPH ... 15

Supplementary Figures ... 16

Figure S1. Interpretation of DI and RAND/UCLA Appropriateness scale ... 16

Figure S2. PRISMA Flowchart ... 17

Figure S3. Second round ranking of one-step thresholds to initiate treatment for intraoperative and postoperative CB PPH ... 18

Supplementary Files ... 19

Supplementary File S1. Search strategies for each database ... 19

Supplementary File S2. Systematic literature review methods ... 24

Supplementary File S3. In-person meeting agenda ... 43

Supplementary File S4. In-person meeting discussion question guide ... 46

Supplementary File S5. List of contributors ... 52

Supplementary File S6. Good Surgical Practices ... 54

For peer review only - http://bmjopen.bmj.com/site/about/guidelines.xhtml

Supplementary Tables

Table S1. Definitions of PPH and severe PPH used in clinical guidelines

Definitions	Number of guidelines using the definition	Specific guidelines using the definition	Specifications regarding mode of birth and timeframe in which bleeding occurs	Additional considerations
**DEFINITIONS OF PPH**				
Blood loss at least 500 mL	6	(1, 2, 3, 4, 5, 6)	Explicitly specified that this definition applied regardless of the mode of birth: (3, 6)<br>Indicated that blood loss must occur within 24 h of birth: (1, 2, 6)	Two guidelines targeting high-income countries (HICs) specified that for caesarean birth the threshold could be set at a higher blood loss if clinically tolerated: (3, 4)
Blood loss at least 1000 mL	2	(7, 8)	Definition applied for caesarean only: all<br>No timeframe indicated: all	---
Blood loss at least 1000 mL OR signs of haemodynamic instability	5	(9, 10, 11, 12, 13)	Definition applied for caesarean only: (9, 10)<br>Indicated that blood loss must occur within 24 h of birth: all	One guideline recommended that cumulative blood loss of 500-999 mL alone should trigger increased supervision and potential interventions as clinically indicated: (13)
Any bleeding that causes haemodynamic instability	1	(14)	Explicitly specified that definition was applicable regardless of mode of birth<br>Indicated that bleeding must occur within 24 h of birth	For clinical purposes, any blood loss that had the potential to produce hemodynamic instability should be considered PPH. The amount of blood loss required to cause hemodynamic instability would depend on pre-existing conditions (e.g., anaemia, dehydration, gestational hypertension with proteinuria)
**DEFINITIONS OF SEVERE PPH**				
Blood loss at least 1000 mL	4	(1, 2, 3, 5)	Explicitly specified that this definition applied regardless of the mode of birth: (3)<br>Indicated that bleeding must occur within 24 h of birth: (1, 2)	RCOG defined major PPH as blood loss greater than 1000 mL. Major PPH could be further subdivided into moderate (1001–2000 ml) and severe (more than 2000 ml).
Blood loss at least 1000 mL OR signs of haemodynamic instability	2	(6, 12)	Explicitly specified that this definition applied regardless of the mode of birth: (6)<br>Indicated that bleeding must occur within 24 h of birth: all	---

Note: No definition of PPH mentioned: (15, 16, 17). No definition of severe PPH mentioned (4, 8, 11, 13, 16, 17, 18)

For peer review only - http://bmjopen.bmj.com/site/about/guidelines.xhtml

Table S2. Synthesis of evidence and considerations regarding approaches to detect intraoperative and postoperative caesarean PPH and haemodynamic instability

Detection methods	Paired with any of the following blood collection devices	Sources discussing this method	Summary of the evidence
**BLOOD LOSS ASSESSMENT METHODS WITH SPECIFIC BLOOD COLLECTION DEVICES**			
Visual estimation of blood loss	• Suction canister<br>• Blood-soaked materials and clots<br>• Calibrated drape<br>• Non-calibrated blood loss collectors placed under the buttocks<br>• Non-calibrated blood loss collectors attached to the abdomen during surgery	4 Guidelines: (1, 8, 11, 14)<br><br>3 Systematic reviews: (19, 20, 21)<br><br>Additional relevant references: (22)	While four guidelines stated that visual estimation was used in practice, none actually recommended this method. Rather, they described its limitations. Visual estimation was described as subjective, imprecise, and known to underestimate actual blood loss (5, 17). Clinicians were advised to be aware that visual estimation of peripartum blood loss is inaccurate (2). The systematic reviews noted that some trialists used visual estimation to determine amount of blood lost and detect PPH. No further comment was provided. Although widely used for the detection of PPH, visual estimation of blood loss was consistently reported as inaccurate (22). While both underestimation and overestimation occur, the extent of underestimation increased as the volume of blood loss increased (17, 22).
Volumetric	• Calibrated suction canister<br>• Calibrated drape	6 Guidelines: (2, 3, 4, 6, 13, 17)<br><br>3 Systematic reviews: (19, 20, 21)<br><br>Additional relevant references: (23, 24, 25, 26, 27)	Guidelines described the use of volumetric methods. Some guidelines proposed using a combination of volumetric and gravimetric methods for assessing blood loss (3, 17). Some guidelines noted that while quantitative methods are more accurate than visual estimation in determining maternal blood loss, their effect on clinical outcomes has not been demonstrated (2, 17). The systematic reviews noted that some trialists used this method to determine amount of blood lost and detect PPH. Some trialists used a combination of gravimetric and volumetric methods. No further comment was provided. Volumetric techniques appeared to be more accurate than the visual estimation of blood loss, irrespective of provider experience, level of training, or specialty (23, 24). Mean measured blood loss was found to be 30% more accurate than estimated blood loss in vaginal births(25). The discrepancy between volumetric methods and visual estimation was found to be higher with increasing blood volume (26). The effectiveness of volumetric methods on clinical outcomes has not been demonstrated (17). A large multicentre multi-country cluster randomized trial comparing calibrated drapes vs. visual estimation failed to show a reduction in severe PPH (27)
Gravimetric	• Non-calibrated blood loss collectors placed under the buttocks<br>• Non-calibrated blood loss collectors attached to the abdomen during surgery<br>• Blood-soaked materials and clots: either intra- or postoperative	8 Guidelines: (2, 3, 4, 5, 6, 12, 13, 17)<br><br>2 Systematic reviews: (19, 20)<br><br>Additional relevant references: (28, 29)	Guidelines described the use of gravimetric methods. Some guidelines proposed using a combination of volumetric and gravimetric methods for assessing blood loss (3, 12, 17). Some guidelines noted that while quantitative methods are more accurate than visual estimation in determining maternal blood loss, their effect on clinical outcomes has not been demonstrated (2, 12, 17). Two guidelines stated that weighing of swabs *may* be used, but did not directly suggest their use (2, 4). The systematic reviews noted that some trialists used this method to determine amount of blood lost and detect PPH. Some trialists used a combination of gravimetric and volumetric methods. No further comment was provided. A 2014 randomized controlled trial including nine hundred women presenting for vaginal delivery found that blood loss recorded using a non-calibrated collector followed by gravimetric assessment was lower than blood loss recorded using the calibrated drape for

For peer review only - http://bmjopen.bmj.com/site/about/guidelines.xhtml

Detection methods	Paired with any of the following blood collection devices	Sources discussing this method	Summary of the evidence
			blood collection followed by volumetric assessment, with a mean difference in recorded blood loss of 58.6ml (28). One study of 228 women with PPH following vaginal delivery found weighing blood loss compared to Hgb drop (of 10%) had a sensitivity of <75% and a specificity of 97%). These findings were modelled at hypothetical high prevalence PPH settings (15%, 30%), where the Positive Predictive Value was >86% (29).
**METHODS OF DETECTING HEMODYNAMIC INSTABILITY SECONDARY TO PPH DURING AND AFTER CAESAREAN BIRTH**			
Clinical signs of haemodynamic instability	• N/A	6 Guidelines: (2, 3, 5, 10, 12, 14)<br>3 Systematic reviews: (19, 20, 21)<br>Additional relevant references: (30, 31, 32, 33, 34, 35, 36)	Signs of haemodynamic instability reported in guidelines included changes in blood pressure, heart rate, pulse oximetry, urine output, or general status (faintness/dizziness, nausea, thirst, altered level of consciousness, pallor, sweating, poor capillary refill, and cold extremities. The guidelines proposed considering clinical signs and symptoms of haemodynamic instability in combination with other methods (such as volumetric and gravimetric methods for blood loss assessment) for the detection of PPH.(2, 3, 5, 10, 12, 14). Some of these guidelines presented tables correlating clinical signs with blood loss and the degree of shock. However, most of them warned that many clinical signs and symptoms do not occur until the blood loss reaches very high levels due to the physiological increase in circulating blood volume during pregnancy. Some other guidelines (6, 12, 13, 14) explicitly proposed including clinical signs and symptoms of haemodynamic instability or the use of the shock index specifically in assessing PPH severity. None of the trials included in the systematic reviews used this method to detect PPH. The Shock Index (SI) as a predictor of several maternal outcomes has been evaluated in the context of PPH research, including both vaginal and caesarean birth. SI performance was usually reported using the Area under the Curve (AUC) parameter (with estimates between 0.7 - 0.8 in most studies). According to the cut-off value of the SI chosen and the specific outcome analysed, SI sensitivity may range from 30% to 90%(30, 31, 32, 33, 34, 35). A recent stepped-wedge cluster randomized trial showed insufficient evidence to suggest that a significant benefit or harm could be attributed using an automated SI device (in low-resource settings) on a composite outcome of maternal deaths, eclampsia, or emergency (36). However, the rate of emergency hysterectomy was significantly reduced.
Visual charts and early warning scores (EWS)	• N/A	5 Guidelines: (2, 4, 6, 12, 13)<br>0 Systematic reviews: None reference this method.<br>Additional relevant references: (37)	A few guidelines recommended visual charts and early warning scores to alert caregivers to abnormal trends in haemodynamic measurements. However, these seem to be recommended for follow-up monitoring of diagnosed PPH cases rather than for diagnosis of PPH. None of the trials included in the systematic reviews used this method to detect PPH. A systematic literature review of 17 published obstetric EWS reported that they had very high median sensitivity (89%) and specificity (85%) but low median positive predictive values (41%) for predicting morbidity or ICU admission. Obstetric EWS had high accuracy in predicting death (AUROC >0.80) among critically ill obstetric women (37).

For peer review only - http://bmjopen.bmj.com/site/about/guidelines.xhtml

Table S3. Synthesis of evidence on thresholds for triggering action on PPH during and after caesarean birth

Thresholds	Guidelines recommending this threshold	Applicable mode of birth (if thresholds differ by mode of birth)	Comments
**ONE-STEP APPROACH: THRESHOLDS TRIGGER FULL RESPONSE PROTOCOL**			
Blood loss at least 1000 mL OR signs of haemodynamic instability	1. (10)<br>2. (11)<br>3. (12)<br>4. (9)	Three guidelines proposed this threshold as being specific for caesarean birth(9, 10, 12).	In these guidelines the same criteria proposed as the definition of PPH were also used as the threshold for initiating treatment.
Blood loss at least 500 mL	1. (6)	This guideline stated that this threshold applied regardless of the mode of birth.	The guideline noted that clinical signs and symptoms of hypovolaemia should be included in the assessment of PPH severity. However, clinical signs of hypovolaemia are misleading in pregnancy due to plasma volume expansion and might not become evident until blood losses reach 1,000-1,500 mL in healthy women. Thus, the blood loss thresholds should depend on the woman's clinical condition and local resources. In this guideline the same criteria proposed for the definition of PPH were also used as the threshold for initiating treatment.
Blood loss at least 500 mL OR Signs of haemodynamic instability	1. (3)	This guideline stated that this threshold applied regardless of the mode of birth.	This guideline noted that the bleeding rate, PPH etiology, and clinical context should be considered. Further, for caesarean births, thresholds could be set at a higher blood loss if clinically tolerated. In this guideline the same criteria proposed for the definition of PPH were also used as the threshold for initiating treatment.
Blood loss at least 1000 mL	1. (7)	This guideline stated that this threshold is specific for caesarean birth.	In this guideline the same criteria proposed for the definition of PPH were also used as the threshold for initiating treatment.
Any excessive bleeding with signs of hemodynamic instability	1.	This guideline stated that this threshold is applicable to all births.	In this guideline the same criteria proposed for the definition of PPH were also used as the threshold for initiating treatment.
**TWO/STEP APPROACH: THRESHOLDS TRIGGER DIFFERENTIAL ACTIONS**			
*Lower threshold: Blood loss at least **500 mL** without clinical shock; Higher threshold: Blood loss at least **1000 ml,** continued bleeding, OR clinical shock*	1. (2)<br>2. (4)<br>3. (5)<br>4. (13)	Guidelines did not specify a difference in threshold by mode of birth.	According to these guidelines, blood loss of **500–1000 ml** (minor PPH) without clinical shock should trigger: close monitoring, laboratory tests, and the use of crystalloid infusion [2] ; prompt basic measures (close monitoring, intravenous access, full blood count, group, and screen, insert urinary catheter) to facilitate resuscitation [4] ; enhanced surveillance and early interventions as needed[13] . Blood loss ≥ **1000 ml** and continued bleeding or clinical shock should trigger a full protocol to achieve resuscitation and haemostasis. [2, 4, 13]Three guidelines aligned their proposed lower thresholds with their proposed PPH definitions. [2, 4, 5] A single guideline aligned its proposed higher threshold with its PPH definition. [13]

Thresholds	Guidelines recommending this threshold	Applicable mode of birth (if thresholds differ by mode of birth)	Comments
Lower threshold: Blood loss at least 1000 mL; Higher threshold: Blood loss of at least 2000 mL OR SI of ≥1.0	1. (8)	Guideline provided thresholds specific for CB	According to this guideline, blood loss of **1000 mL** should trigger suspicion of PPH and initiation of treatment. Blood loss of *at least* **2000 mL or SI of ≥1.0** should trigger: initiation of IV catheter with a large gauge and replacement of a sufficient volume of fluid; consideration of blood transfusion and the transportation of the patient to a secondary or tertiary hospital; monitoring of blood pressure, pulse rate, bleeding amount, urine output and SpO2. This guideline used the same criteria as thresholds for action as were used for proposed PPH definition.

*Four guidelines (1, 15, 16, 17)did not mention any thresholds for initial assessment or to trigger a full protocol. Note CB= caesarean birth

For peer review only

Table S4. Medical Interventions, manoeuvres, and procedures for PPH recommended by WHO compared to recommendations in other PPH guidelines and systematic reviews

Method	WHO recommendation (PPH 2012,TXA 2017, Carbetocin 2018, and UBT 2021) (1, 9, 38, 39)	Other Reviewed Guidelines	Systematic Reviews
**MEDICAL INTERVENTIONS**			
**Uterotonics**			
Oxytocin	Intravenous oxytocin was the recommended first-line treatment for PPH, including among those who have already received oxytocin for prevention of PPH (no dosing information included; 10 IU IM or IV was the recommended dosing and route of administration provided for prophylactic use).	Recommended as first line drug (2, 3, 4, 5, 6, 7, 10, 12, 13, 14, 17)<br>Two different dosing regimens discussed:<br>• Intravenous oxytocin 5 IU (slow IV injection over 2 minutes). May repeat dose once.<br>• Intravenous infusion 5-10 IU per hour (20-40 IU in 500 ml saline over 4 hours).	Not described.
Carbetocin	Not described in the 2012 WHO treatment guidelines. The 2018 WHO prevention guidelines recommended prophylactic carbetocin (100 µg, IM/IV) for all births when cost was comparable to other effective uterotonics, but did not recommend the use of carbetocin as a treatment for PPH.	Recommended as a first line drug (7, 14)or as a second-line drug (10) for treatment.	Not described.
Ergometrine	Recommended if IV oxytocin unavailable, or bleeding nonresponsive to oxytocin. No dosing amount provided. Intravenous route of administration recommended.	Intravenous or intramuscular routes recommended. Maximum dose of 1000 mcg. 250-500 mcg (IV slow, over 2 minutes, or IM), may repeat every 5 minutes (2, 4, 5, 12)	Ergometrine 200 mcg administered intramuscularly, followed by 250 mcg IM carboprost if needed (21)
Oxytocin-ergometrine fixed dose	Recommended if IV oxytocin unavailable, or bleeding nonresponsive to oxytocin. No dosing amount or route of administration recommended. `	Not described.	Syntometrine® (ergometrine 500 mcg plus oxytocin 5 IU) administered intramuscularly plus oxytocin 10 IU administered by an intravenous infusion (21)

For peer review only - http://bmjopen.bmj.com/site/about/guidelines.xhtml

Method	WHO recommendation (PPH 2012,TXA 2017, Carbetocin 2018, and UBT 2021) (1, 9, 38, 39)	Other Reviewed Guidelines	Systematic Reviews
Prostaglandin (including sublingual misoprostol, 800 mcg)	Recommended if IV oxytocin unavailable, or bleeding nonresponsive to oxytocin. No dosing amount or route of administration information provided other than for misoprostol (recommended 800 mcg, administered sublingually). Sublingual misoprostol particularly recommended in settings where IV oxytocin unavailable and IM oxytocin used for prophylaxis.	Carboprost was recommended either: as a general second line drug (12), specifically for use after oxytocin/ergometrine (2), or recommended generally with no specific order cited (5). Most reviewed guidelines recommended a dose of 250 mcg IM, which could be repeated every 15 minutes, up to a maximum dose of 2000 mcg (2, 3, 4, 5, 6, 10, 11, 12, 13, 14). An alternative dosing regimen of 500 mcg intramyometrial route was cited in 6 guidelines (4, 5, 11, 12, 13, 14)Misoprostol was recommended either: as first line drug (5), when other first-line drugs unavailable or contraindicated (12), after oxytocin/ergometrine (2), or if carbroprost contraindicated (4). The recommended dosing regimen was a single dose of 600-1000 mcg, by oral, sublingual, or rectal route (2, 3, 4, 5, 6, 7, 10, 11, 12, 13, 14). Sulprostone IV route, 500 mcg administered over 1 hour (3, 6, 7)	Carboprost 250 mcg administered intramuscularly, followed by 250 mcg IM ergometrine if needed – considered a second line drug (21). Misoprostol 800 mcg (4 tablets of 200 mcg) administered rectally. Considered a first line drug. Data drawn from seven trials, only one of which included some women with caesarean birth (21)
Tranexamic acid with standard care	The 2017 WHO guidelines on tranexamic acid recommended a fixed dose of 1 g (100 mg/mL) administered intravenously at 1 mL per minute (i.e., administered over 10 minutes), with a second 1 g IV dose if bleeding continued after 30 minutes OR restarted within 24 hours of completing the first dose.	Intravenous administration of 1 g over 10 minutes. A second dose may be administered after 30 minutes if bleeding persists (2, 3, 4, 5, 6, 7, 11, 12)	A fixed dose of 1g (100 mg/mL) intravenously at 1 ml per minute, within 3 hours of the time of diagnosis (if unknown, time of birth); a second dose of 1g given if needed 30 minutes from the first dose. Considered a first-line treatment (40).
**MANOEUVRES AND OTHER PROCEDURES**			
**Mechanical interventions**			
Uterine massage	Rubbing of the uterus achieved through manual massage of the abdomen, typically sustained until bleeding ceases or the uterus contracts. (initial rubbing of uterus and expression of clots NOT considered therapeutic uterine massage).<br>Recommended (low cost and relative safety of uterine massage considered in this recommendation).<br>Note: This recommendation was developed considering vaginal birth.	Intervention recommended in other 10 guidelines (2, 3, 4, 5, 7, 10, 11, 12, 14)	Not described.

For peer review only - http://bmjopen.bmj.com/site/about/guidelines.xhtml

Method	WHO recommendation (PPH 2012,TXA 2017, Carbetocin 2018, and UBT 2021) (1, 9, 38, 39)	Other Reviewed Guidelines	Systematic Reviews
Intrauterine balloon tamponade	The procedure entails insertion of a deflated/uninflated balloon into the uterine cavity and then inflating it to achieve a tamponade effect. Uterine balloon tamponade was recommended for the treatment of postpartum haemorrhage due to uterine atony after vaginal birth in women who did not respond to standard first-line treatment, provided all required resources for of PPH are available and routinely implemented (39). Only two studies included in the evidence supporting the recommendations included caesarean deliveries, both of which evaluated the effect of UBT in cases of placenta praevia or traumatic bleeding. One study (41)suggests that the use of the Bakri balloon could be more effective than haemostatic sutures in, and the second (42)suggests benefits associated to the use of the Bakri balloon held in place with a traction stitch versus Bakri balloon without traction stitch.	Guidelines described the urological Rusch balloon (left over 4-6 hrs), the Bakri SOS tamponade balloon catheter, the Sengstaken-Blakemore esophageal catheter, the Foley catheter, the polyurethane Ebb double balloon (vaginal and uterine), and the silicone BT-Cath tamponade balloon (2, 4, 6, 7, 10, 11, 13, 14)	One trial (50 women) compared Bakri Balloon with and without traction stitch; another trial (13 women) compared Bakri balloon to compressive suturing to the lower segment of the uterus. Not specified whether a first-line, second-line, or temporising treatment (20)
Uterine packing	Not recommended for PPH due to uterine atony.	One guideline (13) did not recommend the use of uterine packing while two guidelines recommended its use as a temporizing measure (14) or for unresponsive PPH (11).	Not described.
**Uterine-sparing surgical interventions and procedures**			
Compressive sutures	Compression suturing that runs through the full thickness of both uterine walls. When tied, the suture allows tight compression of the uterine walls and stops the bleeding. No specific suturing technique (e.g., B-Lynch, Hayman, Pereira). Recommended as first-line surgical intervention.	The B-Lynch technique was the most common uterine compression technique for atony (2, 3, 4, 5, 7, 10, 11, 12, 13, 14); however, other techniques, such as Cho and Hayman, were also recommended and described (10, 11).	One trial (160 women) compared the standard B-Lynch suture to a modified B-Lynch suture. Considered a second-line treatment (20).
Devascularisation / Artery ligation	Vascular flow to the uterus can be interrupted by uterine devascularization, ligation of the uterine or internal iliac arteries Recommended only if all available conservative measures (uterotonics, uterine massage, balloon tamponade) have failed.	If compression sutures are unsuccessful, bilateral uterine artery ligation, bilateral utero-ovarian artery ligation or -If expertise available- bilateral internal iliac artery ligation must be considered (2, 3, 4, 5, 6, 7, 10, 11, 12, 14). A common first approach is bilateral uterine artery ligation (O'Leary sutures) (11).	One trial (23 women) compared uterine artery embolization to surgical devascularization plus B-Lynch compression sutures. Not specified wither first-response, second-line, or temporizing treatment (20).

For peer review only - http://bmjopen.bmj.com/site/about/guidelines.xhtml

Method	WHO recommendation (PPH 2012,TXA 2017, Carbetocin 2018, and UBT 2021) (1, 9, 38, 39)	Other Reviewed Guidelines	Systematic Reviews
Uterine artery embolisation (UAE)	If other measures have failed and if the necessary resources were available, the use of uterine artery embolization was recommended as a treatment for PPH due to uterine atony.	Twelve guidelines recommended UAE (1, 2, 3, 4, 5, 6, 7, 10, 11, 12, 13, 14).<br>Only one describes the intervention in greater detail(3)	One trial (23 women) compared uterine artery embolization to surgical devascularization plus B-Lynch compression sutures. Not specified wither first-line, second-line, or temporizing treatment (20).
**Temporising**			
External aortic compression	Recommended as a temporizing measure until appropriate care is available, in PPH due to uterine atony.	In addition to WHO guidelines, seven guidelines recommended external aortic compression (Queensland, (2, 4, 6, 7, 10, 12, 14)	Not described in Systematic Reviews.
Non-pneumatic anti-shock garment	Recommended as a temporizing measure until appropriate care is available.	Recommended in other three guidelines (6, 10, 13).	One systematic review did not find a reduction in maternal mortality associated to NASG in the one cluster-RCT (880 women) included, However, 5 comparative studies (pre-intervention to intervention) included in this review (2330 women) suggested a clinically important reduction in maternal mortality and severe maternal morbidity(43). No effect was observed on the use of blood products. There were no safety issues in all the trials (43).

*Pileggi-Castro 2015 (43) is a systematic review on the non-pneumatic anti-shock garment as a treatment for severe PPH. This review was not captured as part of the overview review because the word "cesarean"/"caesarean" did not appear in the text. After consultation with Prof. Suellen Miller, who participated in each of the primary studies, confirmed that women with caesarean section were included in the original trials, data from this systematic review was added to the report and is reflected in the following tables.

For peer review only - http://bmjopen.bmj.com/site/about/guidelines.xhtml

Table S5. First and second round ratings and agreement for early detection methods for intraoperative and postoperative CB-PPH

Blood loss measurement and other PPH detection methods	How would you rate each of the methods below for early detection of PPH considering…							
	**the usefulness in managing patients?**	**feasibility in all settings performing CB?**	**its acceptability to key stakeholders?**	**the resources required?***	**the usefulness in managing patients?**	**feasibility in all settings performing CB?**	**its acceptability to key stakeholders?**	**the resources required?***
	1=Not at all useful; 9=extremely useful	1=Hardly feasible; 9=Highly feasible	1=Not at all useful; 9=extremely useful	1= very small; 9=very large	1=Not at all useful; 9=extremely useful	1=Hardly feasible; 9=Highly feasible	1=Not at all useful; 9=extremely useful	1= very small; 9=very large
	**FIRST ROUND** Median (DI)				**SECOND ROUND** Median (DI)			
**INTRAOPERATIVE**								
Volumetric/gravimetric + clinical signs of haemodynamic instability	**8.0 (-0.93)**	6.0 (2.35)	NA	NA	NA	**8.0 (-3.08)**	NA	NA
Volumetric	**8.0 (-0.71)**	6.5 (3.50)	7.0 (2.35)	6.5 (1.85)	NA	6.5 (10.00)	7.0 (10.0)	7.0 (2.35)
Clinical signs of haemodynamic instability	6.5 (8.31)	**8.0 (-1.94)**	**8.0 (-0.22)**	**3.5 (0.58)**	**7.0 (-0.71)**	NA	NA	NA
Volumetric + gravimetric	**7.0 (-1.26)**	**5.0 (0.94)**	NA	NA	NA	NA	NA	NA
Clinical judgement such as rate of flow and duration	**5.0 (0.92)**	**7.0 (-21.7)**	6.0 (2.35)	**2.0 (0.49)**	NA	NA	7.0 (10.0)	NA
Visual charts and early warning scores (EWS)	**5.0 (0.88)**	6.0 (2.09)	6.0 (1.96)	5.0 (1.70)	NA	7.0 (10.0)	6.5 (10.0)	6.0 (1.74)
Visual estimation of blood loss	**4.5 (0.97)**	**8.5 (-1.81)**	7.0 (30.0)	**1.0 (0.13)**	NA	NA	**7.0 (-1.94)**	NA
Gravimetric	6.0 (2.35)	**4.5 (0.91)**	**5.0 (0.85)**	6.0 (2.09)	7.0 (10.00)	NA	NA	7.0 (10.00)
Visual estimation + visual charts/EWS	6.0 (1.48)	**7.0 (-0.71)**	NA	NA	6.5 (2.35)	NA	NA	NA
**POSTOPERATIVE**								
Clinical signs of haemodynamic instability	**8.0 (-3.08)**	**8.0 (-1.27)**	**8.0 (-1.94)**	**3.5 (0.58)**	NA	NA	NA	NA
Volumetric/gravimetric + clinical signs of haemodynamic instability	**7.0 (-0.93)**	6.0 (1.74)	NA	NA	NA	7.0 (30.00)	NA	NA
Clinical judgement such as rate of flow and duration	**5.0 (0.41)**	6.0 (2.35)	6.0 (2.35)	**2.0 (0.49)**	NA	6.0 (2.35)	7.0 (10.00)	NA
Gravimetric	5.5 (2.35)	**3.0 (0.65)**	**5.0 (0.85)**	6.0 (2.09)	**5.0 (0.63)**	NA	NA	7.0 (10.00)
Volumetric	5.0 (2.09)	**4.5 (0.58)**	7.0 (2.35)	6.5 (1.85)	**5.0 (0.63)**	NA	7.0 (10.00)	7.0 (2.35)
Visual estimation of blood loss	**4.0 (0.52)**	**7.0 (-3.08)**	7.0 (30.0)	**1.0 (0.13)**	NA	NA	**7.0 (-1.94)**	NA
Visual charts and early warning scores (EWS)	6.0 (2.09)	7.0 (2.35)	6.0 (1.96)	5.0 (1.70)	7.0 (10.0)	7.0 (10.00)	6.5 (10.00)	6.0 (1.74)
Visual estimation + visual charts/EWS	6.0 (4.96)	**7.0 (-3.08)**	NA	NA	7.0 (10.0)	NA	NA	NA
Volumetric + gravimetric	7.0 (8.31)	4.0 (1.04)	NA	NA	7.0 (10.0)	**5.0 (0.85)**	NA	NA

Note: A DI < 1 represented agreement, while a DI ≥ 1 indicated disagreement. Results in which agreement is reached are highlighted in bold.NA= Not applicable given that this combination of methods was not rated in the first round for acceptability to key stakeholders and the estimate of resources required, or because agreement was obtained in the first round. * The measurement scale for this criterion is the same as for the other criteria (from 1 to 9). However, unlike the other criteria, low values have a positive interpretation (few resources required) while high values have a negative interpretation (substantial resources required).

For peer review only - http://bmjopen.bmj.com/site/about/guidelines.xhtml

Table S6. First round ratings and agreement on threshold to initiate treatment for intraoperative CB PPH

Thresholds for triggering action	How would you rate each of the following thresholds for managing PPH during CB considering…		
	**the accuracy of each threshold?** 1=Hardly accurate; 9=Highly accurate	**its feasibility to be used in all settings?** 1=Hardly feasible; 9=Highly feasible	**its acceptability to key stakeholders?** 1=Hardly accepted; 9=Highly accepted
**INTRAOPERATIVE**			
**One-step approach (trigger full response protocol)**			
At least 1000 mL blood loss OR signs of haemodynamic instability, whichever comes first	**9 (-0.34)**	**8 (-0.34)**	**8 (-0.54)**
At least 1000 ml (blood loss alone, regardless of signs of haemodynamic instability)	**7 (-0.71)**	**8 (-0.71)**	**7 (-2.30)**
Haemodynamic instability alone, regardless of volume of blood loss	**7 (-3.08)**	**7 (-0.71)**	**7 (-3.08)**
At least 500 mL blood loss OR signs of haemodynamic instability, whichever comes first	7 (2.30)	**7 (-4.00)**	6.5 (8.31)
At least 500 mL (blood loss alone, regardless of signs of haemodynamic instability)	**5 (0.41)**	6.5 (2.14)	**5 (0.88)**
**Two-step approach (Lower threshold triggers further assessment, preparedness, and close monitoring; Higher threshold triggers treatment initiation)**			
Lower threshold of blood loss at least 500 mL (blood loss alone, regardless of signs of haemodynamic instability), and higher threshold of blood loss at least 1000 mL blood loss OR signs of haemodynamic instability, whichever comes first	**8 (-0.71)**	**8 (-0.71)**	**7 (-0.71)**
Lower threshold of blood loss at least 1000 ml (blood loss alone, regardless of signs of haemodynamic instability), and higher threshold of blood loss at least 2000 mL blood loss OR signs of haemodynamic instability whichever comes first	6.5 (4.96)	**8 (-4.23)**	7 (30.00)
**POSTOPERATIVE**			
**One-step approach (trigger full response protocol**			
At least 1000 mL blood loss OR signs of haemodynamic instability, whichever comes first	**8.5 (-0.34)**	**8 (-2.14)**	**8 (-0.88)**
At least 1000 ml (blood loss alone, regardless of signs of haemodynamic instability)	**7 (-0.71)**	**8 (-0.71)**	**7 (-1.27)**
Haemodynamic instability alone, regardless of volume of blood loss	**7 (-3.08)**	**7 (-4.00)**	7 (10.00)
At least 500 mL blood loss OR signs of haemodynamic instability, whichever comes first	7 (8.31)	**7 (-16.8)**	6.5 (30.00)
At least 500 mL (blood loss alone, regardless of signs of haemodynamic instability)	5 (1.47)	6 (2.09)	5 (1.18)
**Two-step approach (Lower threshold triggers further assessment, preparedness, and close monitoring; Higher threshold triggers treatment initiation)**			
Lower threshold of blood loss at least 500 mL (blood loss alone, regardless of signs of haemodynamic instability), and higher threshold of blood loss at least 1000 mL blood loss OR signs of haemodynamic instability, whichever comes first	**8 (-0.71)**	**8 (-0.71)**	**7.5 (-0.71)**

For peer review only - http://bmjopen.bmj.com/site/about/guidelines.xhtml

Lower threshold of blood loss at least 1000 ml (blood loss alone, regardless of signs of haemodynamic instability), and higher threshold of blood loss at least 2000 mL blood loss OR signs of haemodynamic instability whichever comes first	6.5 (4.41)	6 (2.35)	6 (1.96)

Note: A DI < 1 represented agreement, while a DI ≥ 1 indicated disagreement. Results in which agreement is reached are highlighted in bold. CB= caesarean birth.

For peer review only

For peer review only - http://bmjopen.bmj.com/site/about/guidelines.xhtml

Table S7. First round ratings and agreement for first response interventions for managing intraoperative and postoperative CB-PPH

First response interventions for managing PPH during and after CB	**How would you rate each intervention for first response management of CB-PPH considering…**				
	**the balance of effects?** 1=Weighted towards undesirable effects; 9=Weighted towards desirable effects	**the resources required? *** 1= very few; 9=very many	**its feasibility?** 1=Hardly feasible; 9=Highly feasible	**its acceptability to stakeholders?** 1=Hardly accepted; 9=Highly accepted	**equity?** 1=Likely to exacerbate inequities; 9=Highly likely to reduce inequities
	Median				
INTRAOPERATIVE					
Oxytocin	**9 (-0.34)**	3 (5.86)	**9 (-0.34)**	**9 (0.00)**	**9 (0.00)**
Carbetocin	**8 (-0.93)**	5 (1.81)	**7.5 (-3.79)**	**8 (-0.65)**	6 (6.55)
TXA	**8 (-0.34)**	**4 (0.76)**	**7 (-4.00)**	**8 (-0.93)**	**8 (-3.79)**
Compressive sutures	7 (2.9)	7 (4.64)	6 (4.71)	5.5 (1.76)	6 (4.71)
Bimanual compression	7 (10.15)	**3 (0.64)**	**7.5 (-3.08)**	6.5 (12.8)	**8 (-2.56)**
Uterine massage	**7 (-15.2)**	2 (1.17)	**8.5 (-0.65)**	**8 (-0.93)**	8 (-0.34)
Oxytocin-ergometrine fixed dose	6 (2.35)	4 (1.61)	**7 (-3.08)**	**8 (-3.08)**	**8 (-2.19)**
Prostaglandin (including sublingual misoprostol)	6 (1.37)	**3 (0.87)**	**8 (-3.79)**	**8 (-2.14)**	**8 (-0.93)**
Ergometrine	6 (4.00)	**3 (0.91)**	**8 (-0.92)**	**7 (-0.92)**	**8 (-0.34)**
Non-pneumatic anti-shock garment	5 (1.04)	**6 (0.78)**	**4 (0.52)**	5 (1.08)	5 (1.00)
External aortic compression	**5 (0.56)**	**2 (0.75)**	6 (30.00)	**5 (0.78)**	7 (30.00)
Intrauterine balloon tamponade	4 (1.61)	5 (2.14)	**4 (0.91)**	**5 (0.85)**	6 (4.22)
POSTOPERATIVE					
Oxytocin	**9 (-0.34)**	3 (5.86)	**9 (-0.34)**	**9 (0.00)**	**9 (0.00)**
TXA	**8 (-0.34)**	**4 (0.76)**	**7 (-13.00)**	**8 (-0.93)**	**8 (-6.78)**
Non-pneumatic anti-shock garment	6 (1.35)	6 (1.73)	**4 (0.52)**	**5 (0.99)**	5 (1.00)
Carbetocin	**6 (-10.29)**	5 (1.81)	**7.5 (-8.76)**	**8 (-0.92)**	5.5 (4.22)
Oxytocin-ergometrine fixed dose	6 (2.05)	4 (1.61)	**7 (-3.08)**	**8 (-3.08)**	**8 (-1.54)**
Uterine massage	6 (2.35)	3 (1.17)	**8 (-0.93)**	**8 (-1.68)**	**8 (-3.79)**
Ergometrine	6 (4.00)	**3 (0.97)**	**8 (-0.71)**	**7.5 (-2.19)**	**8 (-0.34)**
Prostaglandin (including sublingual misoprostol)	**6 (0.89)**	3 (1.35)	**8 (-1.53)**	**7.5 (-2.14)**	**8 (-0.93)**
Bimanual compression	**5 (0.63)**	**3 (0.41)**	6 (16.57)	6 (1.73)	**8 (-23.00)**
External aortic compression	**5 (0.68)**	**2 (0.68)**	6 (2.25)	**5.5 (0.52)**	7 (5.6)
Intrauterine balloon tamponade	4 (1.64)	5 (2.14)	**4.5 (0.91)**	**5 (0.85)**	4.5 (4.22)
Compressive sutures	**2 (0.89)**	6 (2.84)	**4 (0.99)**	**3 (0.91)**	4.5 (1.59)

Note: A DI < 1 represented agreement, while a DI ≥ 1 indicated disagreement. Results in which agreement is reached are highlighted in bold. CB= caesarean birth. * The measurement scale for this criterion is the same as for the other criteria (from 1 to 9). However, unlike the other criteria, low values have a positive interpretation (few resources required) while high values have a negative interpretation (substantial resources required)

Table S8. Second round ratings and agreement for first response interventions for managing intraoperative and postoperative CB-PPH

**Intraoperative**	Median (RAND DI)
Examine and rapidly initiate cause-specific first response (e.g., if trauma: rapid surgical haemostasis; if atony/placental cause: uterotonics and uterine massage)	**9 (-0.34)**
TXA for all women with PPH during CB regardless of aetiology	**8 (-0.43)**
Plasma expansion with crystalloids or all women with PPH during CB regardless of aetiology	**7.5 (-1.94)**
Uterotonics for all women with PPH during CB regardless of aetiology	**7 (-0.71)**
**Postoperative**	
Examine and rapidly initiate cause-specific first response (e.g., if trauma: rapid surgical haemostasis; if atony/placental cause: uterotonics and uterine massage)	**9 (-0.34)**
TXA for all women with PPH during CB regardless of aetiology	**8 (0.00)**
Plasma expansion with crystalloids or all women with PPH during CB regardless of aetiology	**7.5 (-0.71)**
Uterotonics for all women with PPH during CB regardless of aetiology	**7 (-0.71)**

Note: A DI < 1 represented agreement, while a DI ≥ 1 indicated disagreement. Results in which agreement is reached are highlighted in bold. CB= caesarean birth.

For peer review only - http://bmjopen.bmj.com/site/about/guidelines.xhtml

Supplementary Figures

Figure S1. Interpretation of DI and RAND/UCLA Appropriateness scale

DI (Disagreement index)	Experts' median rating								
	1	2	3	4	5	6	7	8	9
	Bottom third (1-3)			Intermediate third (4-6)			Top third (1-3)		
<1 (Agreement)	Inappropriate			Uncertain			Appropriate		
≥1 (Disagreement)	Uncertain								

For peer review only

For peer review only - http://bmjopen.bmj.com/site/about/guidelines.xhtml

Figure S2. PRISMA Flowchart

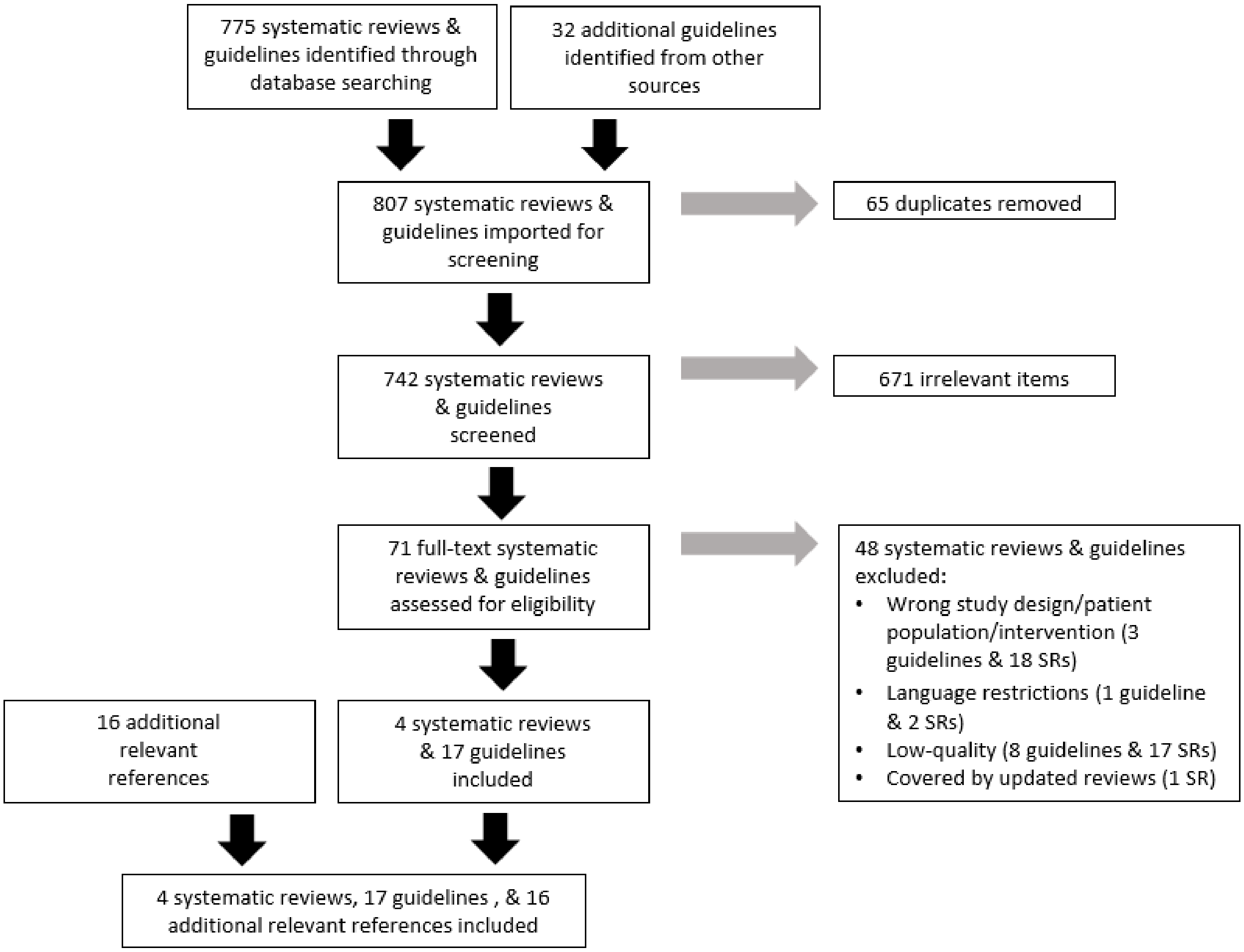

only

For peer review only - http://bmjopen.bmj.com/site/about/guidelines.xhtml

Figure S3. Second round ranking of one-step thresholds to initiate treatment for intraoperative and postoperative CB PPH

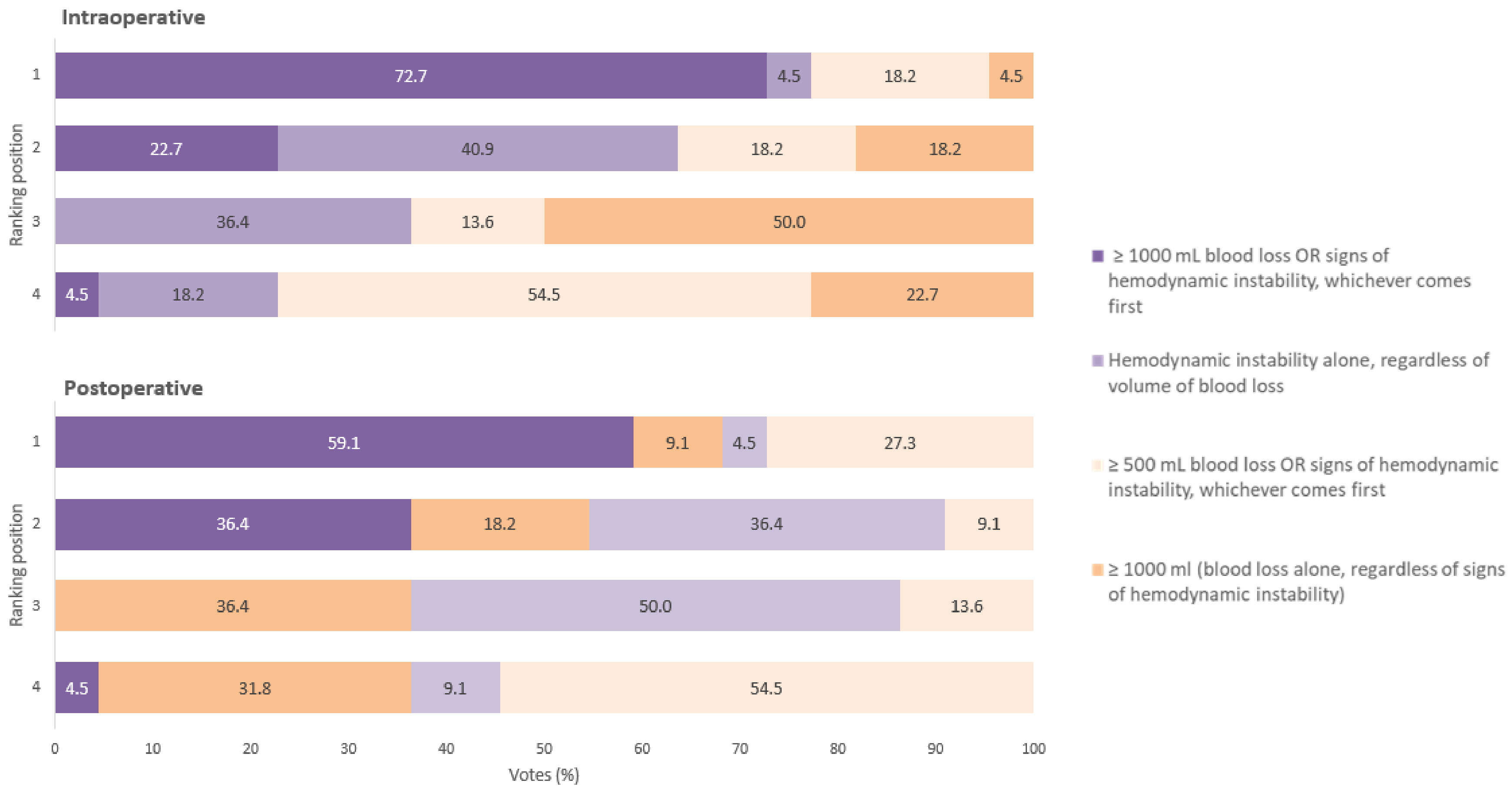

Note: CB= caesarean birth

Supplementary Files

Supplementary File S1. Search strategies for each database

CINAHL (EBSCO) 30-06-2020

#	Query
S23	S20 AND S21 Limiters - Published Date: 20120101-20201231
S22	S20 AND S21
S21	TI (Systematic N1 Review) OR AB (Systematic N1 Review) OR TI Meta-Analys* OR AB Meta-Analys* OR AB Cochrane OR TI Metaanalysis OR AB Metaanalysis OR TI Metanalysis OR AB Metanalysis OR (AB MEDLINE AND AB Cochrane) OR Guideline*[ti] OR TI (Guide N1 Line*) OR AB (Guide N1 Line*) OR TI Consensus OR AB Consensus OR TI Recommendation* OR TI (Technology N1 Assessment) OR AB (Technology N1 Assessment) OR TI (Technology N1 Appraisal) OR AB (Technology N1 Appraisal) OR TI HTA OR AB HTA OR TI Overview OR (TI Review AND TI Literature))
S20	S10 AND S19
S19	S11 OR S12 OR S13 OR S14 OR S15 OR S16 OR S17 OR S18
S18	TI Caesarea* OR AB Caesarea*
S17	TI Caesaria* OR AB Caesaria*
S16	TI Cesaria* OR AB Cesaria*
S15	TI Cesarea* OR AB Cesarea*
S14	TI Cesaerea* OR AB Cesaerea*
S13	TI (C N1 Section*) OR AB (C N1 Section*)
S12	TI C-Section* OR AB C-Section*
S11	(MH "Cesarean Section+")
S10	S1 OR S2 OR S3 OR S4 OR S5 OR S6 OR S7 OR S8 OR S9
S9	TI (Blood N1 Collection) OR AB (Blood N1 Collection)
S8	TI (Blood N1 Specimen) OR AB (Blood N1 Specimen)
S7	TI Bleeding OR AB Bleeding
S6	TI (Blood N2 Loss) OR AB (Blood N2 Loss)
S5	(MH "Blood Specimen Collection+")
S4	TI PPH* OR AB PPH*

For peer review only - http://bmjopen.bmj.com/site/about/guidelines.xhtml

S3	TI Haemorrhag* OR AB Haemorrhag*
S2	TI Hemorrhag* OR AB Hemorrhag*
S1	(MH "Postpartum Hemorrhage")

COCHRANE LIBRARY 30-04-2020

ID

#1 MeSH descriptor: [Postpartum Hemorrhage] explode all trees

#2 Hemorrhag*:ti,ab,kw

#3 Haemorrhag*:ti,ab,kw

#4 PPH*:ti,ab,kw

#5 MeSH descriptor: [Blood Specimen Collection] explode all trees

#6 (Blood NEAR/1 Loss):ti,ab,kw

#7 Bleeding:ti,ab,kw

#8 (Blood NEAR/1 Specimen):ti,ab,kw

#9 (Blood NEAR/1 Collection):ti,ab,kw

#10 #1 OR #2 OR #3 OR #4 OR #5 OR #6 OR #7 OR #8 OR #9

#11 MeSH descriptor: [Cesarean Section] explode all trees

#12 C-Section*:ti,ab,kw

#13 Cesaerea*:ti,ab,kw

#14 Cesarea*:ti,ab,kw

#15 Cesaria*:ti,ab,kw

#16 Caesaria*:ti,ab,kw

#17 Caesarea*:ti,ab,kw

#18 #12 OR #13 OR #14 OR #15 OR #16 OR #17

#19 #10 AND #18 with Cochrane Library publication date Between Jan 2012 and Apr 2020, in Cochrane Reviews

For peer review only - http://bmjopen.bmj.com/site/about/guidelines.xhtml

For peer review only

PUBMED (NLM) 29-04-2020

	Query
#23	**(#20 AND #21)** Filters: **Publication date from 2012/01/01**
#22	**(#20 AND #21)**
#21	**(((Systematic Review[sb] OR Systematic Review[tiab] OR Meta-Analysis[pt] OR Meta-Analys*[tiab] OR "Cochrane Database Syst Rev"[ta] OR Metaanalysis[tiab] OR Metanalysis[tiab] OR Overview[ti] OR (Review[ti] AND Literature[ti]) OR (MEDLINE[tiab] AND Cochrane[tiab]) OR Guideline[pt] OR Practice Guideline[pt] OR Guideline*[ti] OR Guide Line*[tiab] OR Consensus[tiab] OR Recommendation*[ti])))**
#20	**(#11 AND #19)**
#19	**(#12 OR #13 OR #14 OR #15 OR #16 OR #17 OR #18)**
#18	**Caesarea*[tiab]**
#17	**Caesaria*[tiab]**
#16	**Cesaria*[tiab]**
#15	**Cesarea*[tiab]**
#14	**Cesaerea*[tiab]**
#13	**C-Section*[tiab]**
#12	**Cesarean Section[Mesh]**
#11	**((#1 OR #2 OR #3 OR #4 OR #5 OR #6 OR #7 OR #8 OR #9 OR #10))**
#10	**Blood Collection[tiab]**
#9	**Blood Specimen[tiab]**
#8	**Bleeding[tiab]**
#7	**Loss of Blood”[tiab]**
#6	**Blood Loss[tiab]**
#5	**Blood Specimen Collection[Mesh]**
#4	**PPH*[tiab]**
#3	**Haemorrhag*[tiab]**
#2	**Hemorrhag*[tiab]**
#1	**Postpartum Hemorrhage[Mesh]**

EMBASE (ELSEVIER) 29-04-2020

No. Query Results Results Date

#22. #21 AND (2012:py OR 2013:py OR 2014:py OR 2015:py

For peer review only - http://bmjopen.bmj.com/site/about/guidelines.xhtml

OR 2016:py OR 2017:py OR 2018:py OR 2019:py OR 2020:py)

#21. #19 AND #20

#20. (('systematic review':ti,ab OR 'meta analysis (topic)':pt OR 'meta analysis':ti,ab OR cochrane:jt OR metaanalysis:ti,ab OR metanalysis.ti,ab. OR (medline:ab AND cochrane:ab) OR practice) AND guideline:pt OR guideline*:ti OR 'guide lines':ti,ab OR consensus:ti,ab OR recommendation*:ti OR biomedical) AND technology AND 'assessment'/exp OR 'technology assessment':ti,ab OR 'technology appraisal':ti,ab OR hta:ti,ab OR overview:ti OR (review:ti AND literature:ti)

#19. #10 AND #18

#18. #11 OR #12 OR #13 OR #14 OR #15 OR #16 OR #17

#17. caesarea*:ti,ab

#16. caesaria*:ti,ab

#15. cesaria*:ti,ab

#14. cesarea*:ti,ab

#13. cesaerea*:ti,ab

#12. (c NEAR/1 section*):ti,ab

#11. 'cesarean section'/exp

#10. #1 OR #2 OR #3 OR #4 OR #5 OR #6 OR #7 OR #8 OR #9

#9. (blood NEAR/1 collection):ti,ab

#8. (blood NEAR/1 specimen):ti,ab

#7. bleeding:ti,ab

#6. (blood NEAR/1 loss):ti,ab

For peer review only

For peer review only - http://bmjopen.bmj.com/site/about/guidelines.xhtml

#5. 'blood specimen collection kit'/exp

#4. pph*:ti,ab

#3. haemorrhag*:ti,ab

#2. hemorrhag*:ti,ab

#1. 'postpartum hemorrhage'/exp

LILACS (BVS-EN) 1-05-2020

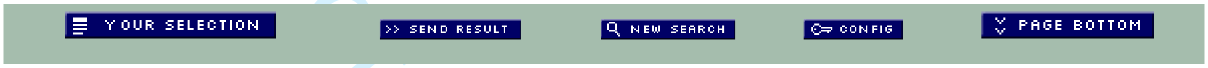

Database : **LILACS**

: **(MH Postpartum Hemorrhage OR Hemorrhag$ OR Hemorragia OR PPH$ OR Haemorrhag$ OR MH Blood Specimen Collection OR Sangrado OR Sangrando) AND (MH Cesarean Section OR C-Section$ OR Cesaerea$ OR Cesarea$ OR Cesaria$ OR Caesaria$ OR Caesarea$) [Words] and 2012 OR 2013 OR 2014 OR 2015 OR 2016 OR 2017 OR 2018 OR 2019 OR 2020 [Country, year publication]**

For peer review only

For peer review only - http://bmjopen.bmj.com/site/about/guidelines.xhtml

Supplementary File S2. Systematic literature review methods

Overview

The preceding evidence synthesis includes literature identified through two consecutive processes. First, we conducted an overview review of guidelines and systematic reviews. After synthesizing this evidence, we conducted complementary targeted searches for literature (relevant references) on specific topics to help round out the evidence base. For the purposes of this report, systematic reviews, guidelines, and relevant references were defined as follows:

Overview of Reviews: A systematic method for searching, identifying, and synthesizing systematic reviews

Guideline: A position paper of scientific or professional societies or state-based task groups, with recommendations based on medical evidence and consensus among authors.

Systematic review: A structured review of scientific evidence, with explicit criteria regarding literature search, inclusion and exclusion of articles.

Meta-analysis: A method for systematically combining quantitative data from multiple studies to analyze larger trends.

Other Relevant References: Scientific articles, which were identified via expert recommendation, were added to this evidence base and analyzed. These references were included to gain more insight about PPH detection methods.

Systematic search: A formalized approach to searching the literature with pre-specified search terms and criteria. Often, but not always, used as the first step in a systematic review.

Each of these processes are described in detail below.

Overview of reviews

We conducted an overview of reviews of guidelines, systematic reviews (including rapid, scoping, and umbrella systematic reviews), and meta-analyses reporting detection and first response interventions to suspected PPH during and within the first 24 hours following the caesarean section. As stated above, overview reviews generally are applied to systematic reviews of research questions for the purpose of extracting and analysing their results across important outcomes. In preparing this report, we adapted this methodology to also include guidelines and recommendations.

Search Strategy and Screening Process

A systematic search was conducted for peer-reviewed systematic reviews published from January 2012 to July 2020 in the following electronic databases: PubMed, EMBASE, CINAHL, and Cochrane Library. The

For peer review only - http://bmjopen.bmj.com/site/about/guidelines.xhtml

For peer review only

search was complemented by reviewing the grey literature published during the same period to identify guidelines and recommendations in repositories, websites, and national ministries of health from any English-speaking country and/or written in English.

Titles and abstracts of all identified sources were imported into Covidence (Covidence) for initial screening; those that were potentially eligible were selected for full-text review. Pairs of independent reviewers (CRW, FM, AL, AB, and VP) reviewed full texts. Systematic reviews which included analytic non-comparative studies (e.g., case series) were excluded.

Clarification: Whenever possible, definitions, detection methods, thresholds, and conservative treatments specific to PPH that develops during or after caesarean section were extracted from the guidelines. However, some guidelines (Definitions: 11/17 [65%]; Detection Methods: 16/17 [94%]; Thresholds: 9/17 [53%]; and Treatments: 16/17 [94%]) did not specify the mode of birth in PPH-related recommendations. Due to this, the included guidelines' summaries contain both a) recommendations specific to PPH during and after caesarean section and b) recommendations where the mode of birth is not specified. Therefore, some of the detection methods and treatments in this document may seem less suitable for intraoperative PPH than for postoperative PPH.

Quality appraisal

Following selection, pairs of independent reviewers (CRW, FM, AL, AB, and VP) assessed the quality of included full texts using the AGREE reporting checklist for guidelines and recommendations (44) and a modified version of the AMSTAR 2 quality assessment tool for systematic reviews (45). AGREE II is the new (2010) international tool to assess the quality and reporting of practice guidelines (44). AMSTAR 2 is an updated version of the AMSTAR tool initial developed by the Cochrane Collaboration in 2007 to aid in the critical appraisal of systematic reviews. It is one of the most widely-used such tools (45). Reviewers then met to discuss any conflicts. Disagreements were discussed until consensus was reached, and if required, a third reviewer was consulted.

The AGREE II reporting checklist was used as intended. The following modifications were made to the AMSTAR 2 quality assessment tool.

Using the AMSTAR 2 tool, all systematic reviews are assessed on 16 total domains (see Appendix), which together encompass various aspects of systematic review quality, ranging from adequacy of the literature search to management of potential risk of bias in included primary studies. Each included systematic review is assessed individually. Once the overall assessment is complete, the AMSTAR 2 authors propose a scoring system for developing an overall quality rating for the systematic review (45). Under the proposed scoring system, 7 domains are weighted more heavily than the others. These more heavily weighted domains are considered "critical domains" and inadequate responses in these domains are considered "critical flaws."

Under the AMSTAR 2 scoring system, the quality of assessed systematic reviews is determined based on the number of critical and non-critical flaws in the review:

For peer review only - http://bmjopen.bmj.com/site/about/guidelines.xhtml

- High: No or one non-critical flaw
- Moderate: More than one non-critical flaw
- Low: One critical flaw
- Critical low: More than one critical flaw

The AMSTAR 2 authors propose seven items (2, 4, 7, 9, 11, 13, and 15) as critical domains. Upon discussion by a panel of experts and the systematic review team, it was decided that only four items (4, 7, 9, and 13) were relevant to our systematic review. These items are:

- Adequacy of the literature search (item 4)
- Justification for excluding individual studies (item 7)
- Risk of bias from individual studies being included in the review (item 9)
- Consideration of risk of bias when interpreting the results of the review (item 13)

Items determined to be irrelevant for our systematic review were item 2 (protocol registered before commencement of the review; since all included systematic reviews were Cochrane Reviews this criterion was met), item 11 (appropriateness of meta-analytical methods; since no meta-analysis was conducted as part of our overview review), and item 15 (assessment of presence and likely impact of publication bias).

Only guidelines with AGREE II scores between 5-7 (AGREE scores range from 0-7) and systematic reviews with modified-AMSTAR quality assessment of Moderate or High were eligible for data extraction.

Outcomes

Main outcome(s)

We will list and describe the frequency and characteristics of reported and recommended interventions for the detection and initial management of caesarean section related PPH.

Additional outcome(s)

In addition, we will describe reported caesarean section PPH definitions, methods for blood loss estimation or measurement, clinical criteria to diagnose PPH and thresholds.

Interventions will be classified according to the time of occurrence: intra or post-operative PPH, and the country and world group (Low income, Lower middle income, Upper middle income or High income) that released the recommendation.

Data Extraction and Synthesis

For peer review only

For peer review only - http://bmjopen.bmj.com/site/about/guidelines.xhtml

Each selected systematic review was independently extracted by two reviewers (CRW and FM). Data on PPH definitions, thresholds, detection methods, and treatments were extracted using a common template. Following independent extraction, the reviewers met to discuss any conflicts until consensus was reached, with a third reviewer (VP) joining to resolve conflicts as needed. Final decisions on data extraction were captured in a consolidated database.

Each selected guideline was reviewed by two independent reviewers (AL, AB, and VP). Data on PPH definitions, thresholds, detection methods and treatments were extracted using a common template. Following independent extraction, the reviewers met to discuss any conflicts until consensus was reached. Final decisions on data extraction were captured in a consolidated database. Data were synthesized into descriptive tables based on each of the four topic areas. A brief description of included studies is included in the "Findings" section of this document, with synthesized findings organized by topic area provided in the preceding Evidence Synthesis.

Additional Relevant Resources

Search Strategy and Screening Process

An additional search for peer-reviewed manuscripts was conducted using PubMed with the following search terms: 'Postpartum Hemorrhage' AND 'Detection Methods'. Peer-reviewed RCTs were eligible for inclusion. Subject matter experts were also consulted to add any relevant peer-reviewed articles missed by the systematic search.

Data Extraction and Narrative Synthesis

Each relevant resource article identified through the additional search was reviewed and narratively synthesized by a single member of the team (AL or AB). A second member of the team (AB, VP, or CRW) reviewed the full article, and then reviewed the drafted synthesis. Multiple iterative rounds of revision occurred between the two reviewers until they reached consensus. Narrative syntheses were then added to the corresponding report tables.

For peer review only - http://bmjopen.bmj.com/site/about/guidelines.xhtml

Study Flowchart

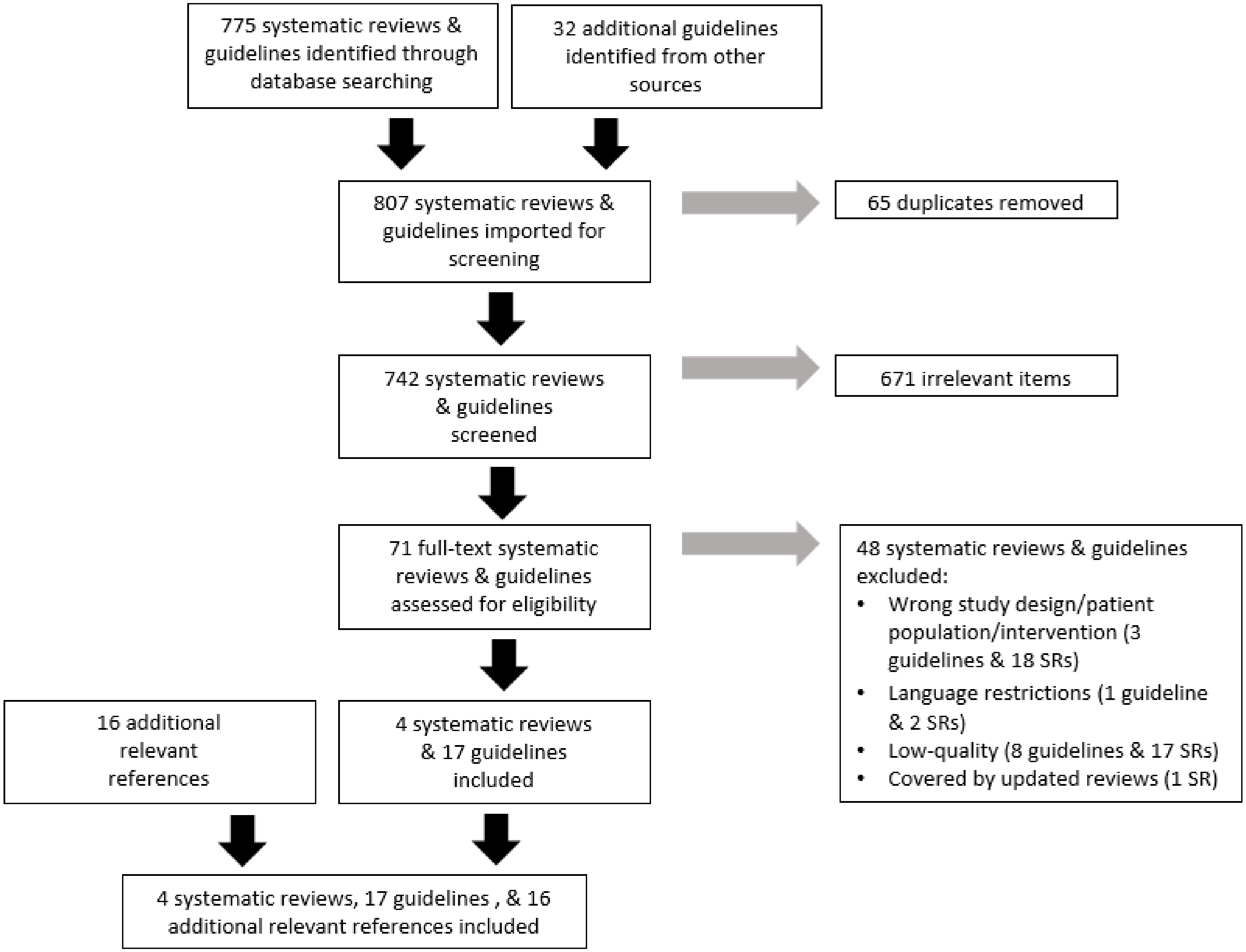

For peer review only - http://bmjopen.bmj.com/site/about/guidelines.xhtml

Characteristics of included guidelines and systematic reviews

Guidelines and recommendations

The overview review included 17 guidelines with an overall AGREE II score of 5-7 (scale 0-1) from professional bodies (n=11), the WHO (n=3), government organizations (n=2), and a consortium (n=1). Of the 17 guidelines, 14 were guidelines specific to PPH; two covered general obstetric practice, and one was on caesarean section. Five guidelines were published or updated between 2012-2015 and 12 between 2015-2019. Of the 17, four guidelines were international in scope (WHO, FIGO), while 13 were developed for audiences in high-income countries (HIC). See tables below on Quality appraisal of included guidelines and Main characteristics of included guidelines.

Systematic reviews

The overview review also included four Cochrane systematic reviews, published between 2015 and 2020 See tables below on Quality appraisal of included systematic reviews and Main characteristics of included systematic reviews. All systematic reviews included data on women with PPH following both caesarean section and vaginal birth. The four Cochrane systematic reviews together constitute an update of a single previous Cochrane systematic review from 2014 on the treatment of primary postpartum haemorrhage (46). Although the initial assessment met inclusion criteria, as updated reviews from 2018 and 2020 were available, the initial review was excluded, and the four updated reviews were included instead.

Shakur 2018 was a Cochrane systematic review on antifibrinolytic drugs to treat primary PPH. This study included data across 3 RCTs and included high-, middle-, and low-income countries (40). Gallos 2018 was a Cochrane systematic review and network meta-analysis on uterotonics agents for preventing PPH (19). Although this review was focused on PPH prevention, not first-response management, it is included as one of four Cochrane systematic reviews that together constituted the updating of Mousa 2014 (46). In addition, Gallos 2018 included information on definitions, thresholds, and blood collection and measurement techniques. Gallos 2018 included 196 RCTs from 53 countries. Kellie 2020 was a Cochrane systematic review that compared mechanical and surgical interventions for treating PPH, including comparisons of each technique plus standard care versus standard of care alone, as well as head-to-head comparisons of different techniques. The review included 9 RCTs and data from 7 LMICs (20). The final Cochrane systematic review and network meta-analysis (Parry Smith 2020) surveyed the evidence of uterotonics agents for first-line treatment of PPH (21). This review included 7 RCTs from 10 LMICs. Nearly all data was drawn from women with vaginal births; a small subset of women in one of the included RCTs (Lokugamage 2001) gave birth via caesarean section.

Additional relevant references

Additional relevant references were taken into consideration for Chapter 2 of the Evidence Synthesis: Detection Methods of PPH during and after caesarean birth. Sixteen additional peer-reviewed primary studies were identified through the methodology described above. Given the heterogeneity of these studies, a systematic approach to data extraction was not undertaken;

For peer review only - http://bmjopen.bmj.com/site/about/guidelines.xhtml

rather, the studies were reviewed and narratively synthesized by one member of the research team, with a second member of the research team subsequently reviewing the synthesis for accuracy and completeness. Information from these syntheses was then added to Table 2.2 in the Evidence Synthesis.

For peer review only

For peer review only - http://bmjopen.bmj.com/site/about/guidelines.xhtml

Quality appraisal of included guidelines

Organization	Title	Year of publication	Percentage of the maximum score							Final Overall Score
			Domain 1	Domain 2	Domain 3	Domain 4	Domain 5	Domain 6	OA1 - Total	
WHO	WHO recommendations for the prevention and treatment of postpartum haemorrhage	2012	100%	100%	95%	100%	81%	88%	100%	7
WHO	WHO recommendation on tranexamic acid for the treatment of postpartum haemorrhage	2017	100%	100%	100%	83%	88%	100%	100%	7
NICE	Caesarean section	2019	100%	86%	100%	100%	96%	75%	100%	7
RCOG	Prevention and Management of Postpartum Haemorrhage	2016	100%	86%	91%	100%	33%	75%	92%	7
WHO	Managing Complications in Pregnancy in Childbirth	2017	94%	100%	49%	100%	96%	100%	75%	6
RCPI; Health Service Executive	Prevention and management of primary postpartum haemorrhage	2014	97%	94%	75%	100%	85%	100%	92%	6
Queensland Health	Queensland Clinical Guidelines: Primary Postpartum Haemorrhage	2019	97%	81%	56%	100%	96%	92%	75%	6
FCNGOF	Postpartum hemorrhage: French College of Gynaecologists and Obstetricians	2016	57%	50%	64%	96%	47%	83%	72%	5

For peer review only - http://bmjopen.bmj.com/site/about/guidelines.xhtml

Organization	Title	Year of publication	Percentage of the maximum score							Final Overall Score
			Domain 1	Domain 2	Domain 3	Domain 4	Domain 5	Domain 6	OA1 - Total	
RANZCOG	Management of Postpartum Haemorrhage	2017	100%	89%	54%	100%	10%	81%	67%	5
SOGC	Active Management of the Third Stage of Labour: Prevention and Treatment of Postpartum Hemorrhage	2018	97%	64%	58%	100%	19%	88%	67%	5
DGGG, OEGGG and SGGG	Peripartum Haemorrhage, Diagnosis and Therapy	2016	94%	67%	64%	100%	29%	86%	67%	5
FIGO	Guidelines for Prevention and Treatment of Postpartum Hemorrhage in low resource settings	2012	86%	56%	44%	94%	94%	96%	67%	5
NATA	Patient blood management in obstetrics: prevention and treatment of postpartum haemorrhage	2016	94%	67%	58%	100%	21%	75%	67%	5
ACOG	Postpartum Hemorrhage	2017	98%	54%	60%	87%	58%	50%	67%	5
Standford and others	International consensus statement on the use of uterotonic agents during caesarean section	2019	100%	57%	36%	100%	18%	100%	67%	5

For peer review only - http://bmjopen.bmj.com/site/about/guidelines.xhtml

Organization	Title	Year of publication	Percentage of the maximum score							Final Overall Score
			Domain 1	Domain 2	Domain 3	Domain 4	Domain 5	Domain 6	OA1 - Total	
JSOG/JAOG	Guidelines for obstetrical practice in Japan	2014	93%	72%	28%	72%	7%	89%	61%	5
ACOG	Quantitative Blood Loss in Obstetric Hemorrhage	2019	83%	56%	39%	97%	50%	88%	58%	5

Quality appraisal of included systematic reviews

	Gallos 2018	Kellie 2020	Parry-Smith 2020	Shakur 2018
Overall Quality Assessment	HIGH	HIGH	HIGH	MODERATE
Item 1: PICO components described in research questions and inclusion criteria	Yes	Yes	Yes	Yes
Item 2: Evidence of pre-established protocol and explanation of any deviations	Yes	Yes	Yes	Yes
Item 3: Justification of included study designs	No	Yes	Yes	No
Item 4: Comprehensive literature search strategy, including consultation with content experts*	Partial	Partial	Partial	Partial
Item 5: Study selection performed in duplicate	Yes	Yes	Yes	Yes
Item 6: Data extraction performed in duplicate	Yes	Yes	Yes	Yes

For peer review only - http://bmjopen.bmj.com/site/about/guidelines.xhtml

	Gallos 2018	Kellie 2020	Parry-Smith 2020	Shakur 2018
Overall Quality Assessment	HIGH	HIGH	HIGH	MODERATE
Item 7: List of excluded studies and justification for exclusion*	Yes	Yes	Yes	Yes
Item 8: Adequate description of included studies	Yes	Yes	Yes	Yes
Item 9: Satisfactory technique used to assess risk of bias in included studies*	Yes	Yes	Yes	Yes
Item 10: Report of sources of funding for included studies	Yes	Yes	Yes	Yes
Item 11: Use of appropriate methods for statistical combination of results	Yes	N/A	No	Yes
Item 12: Assessment of potential impact of risk of bias on results of evidence synthesis	Yes	N/A	Yes	Yes
Item 13: Accounting for risk of bias from included studies in interpretation/discussion of results*	Yes	Yes	Yes	Yes
Item 14: Satisfactory explanation for, and discussion of, any observed heterogeneity	Yes	No	Yes	Yes
Item 15: Adequate investigation of publication bias	Yes	NA	No	No
Item 16: Reporting and management of potential conflicts of interest	Yes	Yes	Yes	Yes

*Considered a critical component of the authors' modified AMSTAR II ranking scale

Main characteristics of included guidelines

For peer review only - http://bmjopen.bmj.com/site/about/guidelines.xhtml

Organization[1]	Name of the Guideline	Year of publication or update	Geographical scope	Main topic
International Agencies				
WHO	WHO recommendation on tranexamic acid for the treatment of postpartum haemorrhage	2017	Global	PPH
WHO	Managing Complications in Pregnancy in Childbirth	2017	Global	Obstetric complications
WHO	WHO recommendations for the prevention and treatment of postpartum haemorrhage	2012	Global	PPH
Government Organizations				
NICE	Caesarean section	2019	UK	CS
Queensland Health	Queensland Clinical Guidelines: Primary Postpartum Haemorrhage	2019	Queensland, Australia	PPH
Professional Body				
ACOG	Quantitative Blood Loss in Obstetric Haemorrhage	2019	US	Quant. blood loss

[1] WHO (World Health Organization), NICE (National Institute for Health and Care Excellence), CMQCC (California Maternal Quality Care Collaborative), ACOG (American College of Obstetricians and Gynecologists), SOGC (Society of Obstetricians and Gynaecologists of Canada), RANZCOG (Royal Australian and New Zealand College of Obstetricians and Gynaecologists), NATA (Network for the Advancement of Patient Blood Management Haemostasis and Thrombosis), FCNGOF (French College of Gynaecologists and Obstetricians, DGGG (German Society of Gynecology and Obstetrics), OEGGG (Austrian Society of Gynecology and Obstetrics), Swiss Society of Obstetrics and Gynecology, RCOG (Royal College of Obstetrics and Gynaecology, HSE (Health and Safety Executive, JSOG/JAOG (Japan Society of Obstetrics and Gynecology, FIGO (The International Federation of Gynecology and Obstetrics)

For peer review only - http://bmjopen.bmj.com/site/about/guidelines.xhtml

Organization1	Name of the Guideline	Year of publication or update	Geographical scope	Main topic
SOGC	Prevention and Treatment of Postpartum Haemorrhage	2018	Canada	PPH
RANZCOG	Management of Postpartum Haemorrhage	2017	Australia/New Zealand	PPH
ACOG	Postpartum Haemorrhage	2017	US	PPH
NATA	Patient blood management in obstetrics: prevention and treatment of postpartum haemorrhage	2016	Global	PPH and Blood management
FCNGOF	Postpartum haemorrhage: French College of Gynaecologists and Obstetricians	2016	France	PPH
DGGG, OEGGG and SGGG	Peripartum Haemorrhage, Diagnosis and Therapy	2016	Germany	PPH
RCOG	Prevention and Management of Postpartum Haemorrhage	2016	UK	PPH
Royal College of Physicians of Ireland; HSE	Prevention and management of primary postpartum haemorrhage	2014	Ireland	PPH
JSOG/JAOG	Guidelines for obstetrical practice in Japan	2014	Japan	Obstetrical practice
FIGO	Guidelines for Prevention and Treatment of Postpartum Haemorrhage in low resource settings	2012	Global	PPH
Consortium				
CMQCC	Improving Health Care Response to Obstetric Haemorrhage (v2): A California Quality Improvement Toolkit	2015	US	PPH

For peer review only - http://bmjopen.bmj.com/site/about/guidelines.xhtml

Main characteristics of included systematic reviews

Author & Year	Title	Geographical scope	Number of Studies	Sample size
Shakur 2018	Antifibrinolytic drugs for treating primary postpartum haemorrhage	Argentina, Australia, Canada, Denmark, Finland, France, Hong Kong, Korea, Ireland, Israel, Italy, Japan, Netherlands, New Zealand, Sweden, UK, USA	3	20,412
Gallos 2018	Uterotonic agents for preventing postpartum haemorrhage: a network meta-analysis	Argentina, Australia, Austria, Bangladesh, Belgium, Canada, China, Colombia, Egypt, Ecuador, France, Ghana, Guinea Bissau, Hong Kong, Hungary, India, Indonesia, Iran, Ireland, Italy, Jamaica, Kenya, Korea, Kuwait, Libya, Malaysia, Mexico, Mozambique, Nepal, Netherlands, Nigeria, Norway, Panama, Pakistan, Papua, Philippines, Saudi Arabia, Senegal, Singapore, South Africa, Spain, Sweden, Switzerland, Thailand, Turkey, Tunisia, UAE, Uganda, UK, USA, Venezuela, Vietnam, West Indies, Zimbabwe	196	135,559
Kellie 2020	Mechanical and surgical interventions for treating primary postpartum haemorrhage	Benin, Egypt, Mali, Saudi Arabia, Thailand, Turkey, and Pakistan	9	994
Parry-Smith 2020	Uterotonic agents for first-line treatment of postpartum haemorrhage: a network meta-analysis	Argentina, Burkina Faso, Ecuador, Egypt, Gambia, Pakistan, South Africa, Thailand, Turkey, and Vietnam	7	3,738

For peer review only

For peer review only - http://bmjopen.bmj.com/site/about/guidelines.xhtml

ADDITIONAL EVIDENCE SYNTHESIS PROCESSES

Detection Methods Pre-Screening Process

The initial list of detection methods developed based on the literature review included several innovative methods that the research team deemed either inapplicable to low-resource settings, or not useful for early detection and triggering immediate treatment. Accordingly, the research team developed a set of criteria to be used to assess the relevance of each method. The criteria were:

- Appropriateness of early detection and triggering immediate treatment
- Feasibility of use in most secondary-level or higher hospitals
- Applicability to intraoperative AND/OR postoperative PPH detection

Three experts (SM, IG, FA) were asked to independently rate each detection method identified in the literature on all criteria. The whole group then met to discuss disagreements until consensus was reached. Detection methods were deemed irrelevant for the purposes of this report if all experts rated them as "No" for all criteria OR if the current cost of the detection method rendered it unaffordable in LMICs. The completed screening tool with decisions on each considered detection method is provided in Supplementary Table 3.

Detection methods deemed relevant for the purposes of this report were then described (descriptions and synthesis are included in the Evidence Synthesis). Detection methods as presented in the Evidence Synthesis were composed of both blood loss assessment methods (which utilized varying blood collection devices to assist with quantification of blood loss) and other methods of assessing PPH. Supplementary Figure 1 provides a useful schematic for how the research team categorized each of the detection methods.

For peer review only - http://bmjopen.bmj.com/site/about/guidelines.xhtml

Pre-screening of PPH Detection Methods

PPH detection method -- Description	Is this method appropriate for EARLY detection and triggering immediate treatment?	Is this method likely to be feasible to use in most secondary-level or higher hospitals?	Is this method applicable to intraoperative PPH detection?	Is this method applicable to postoperative PPH detection?	Comments
Visual estimation of blood loss<br>Obstetric or anaesthetic team visually estimates volume of blood loss. It mainly relies on a providers' opinion based on clinical experience and use of visual aids	No	Yes	Yes	Yes	Commonly used to trigger treatment. However, this method grossly underestimates blood loss and thus is not useful for early detection
Clinical signs of PPH<br>Signs of hypovolemia, hypotension (systolic below 80 mmHg), tachycardia (heart rate over 100 bmp), tachypnoea, cardiac output (decreased pulse pressure and respiratory rate) and altered mental state.	No	Yes	Yes	Yes	Commonly used to trigger treatment. However, except in anaemic women, there may be a substantial delay in the onset of clinical signs of PPH, undermining utility for early detection Also, blood pressure may be artificially high due to medications used, LOC affected by medications.
Visual charts and early warning scores<br>Visual charts usually involve scoring each parameter of vital signs monitoring (usually temperature, pulse, blood pressure, respiratory rate, and conscious level), with the aggregate score determining the need for closer monitoring, intervention, and review. Some charts are not validated for use with childbearing women.	Yes	Yes	Yes	Yes	A recent systematic review found that Obstetric Early Warning Systems are effective for early detection and triggering action, particularly when color-coded/shaded.

For peer review only

PPH detection method -- Description	Is this method appropriate for EARLY detection and triggering immediate treatment?	Is this method likely to be feasible to use in most secondary-level or higher hospitals?	Is this method applicable to intraoperative PPH detection?	Is this method applicable to postoperative PPH detection?	Comments
Changes in laboratory values such as haemoglobin and haematocrit<br>Evaluation of haemoglobin concentration through blood sample.	No	Yes	No	Yes	Due to time lag in receiving results, this method is not useful intraoperatively, but could potentially be useful post-operatively if adequate and functioning laboratory facilities are available.
Artificial Intelligence (Triton)<br>Image recognition algorithms are used with pictures of blood-soaked items to perform colorimetric analysis which quantifies haemoglobin and blood loss.	Yes	No	Yes	Yes	Not currently accessible for LMIC.
Dye Dilution<br>Assesses blood flow through venous system by injecting a known quantity of dye into a vein and monitoring its plasmatic concentration after uterine bleeding stops.	No	No	No	No	Due to time lag in receiving results, this method is not useful intraoperatively, but could potentially be useful post-operatively.
Spectrophotometry<br>This method is based on collected blood being mixed with a standardized solution which converts haemoglobin to acid haematin or cyanmethemoglobin. This in turn can be measured by a spectrophotometer or colorimeter.	No	No	No	No	Rare and not currently accessible for LMIC.

For peer review only

PPH detection method -- Description	Is this method appropriate for EARLY detection and triggering immediate treatment?	Is this method likely to be feasible to use in most secondary-level or higher hospitals?	Is this method applicable to intraoperative PPH detection?	Is this method applicable to postoperative PPH detection?	Comments
Initial Imaging<br>This technique involves US duplex Doppler pelvis, US pelvis transabdominal, US pelvis transvaginal imaging to detect uterine atony or lack of uterine contraction.	No	No	No	No	Unclear if available in LMICs.

For peer review only

Blood loss collection devices, blood loss assessment methods and other methods of PPH detection

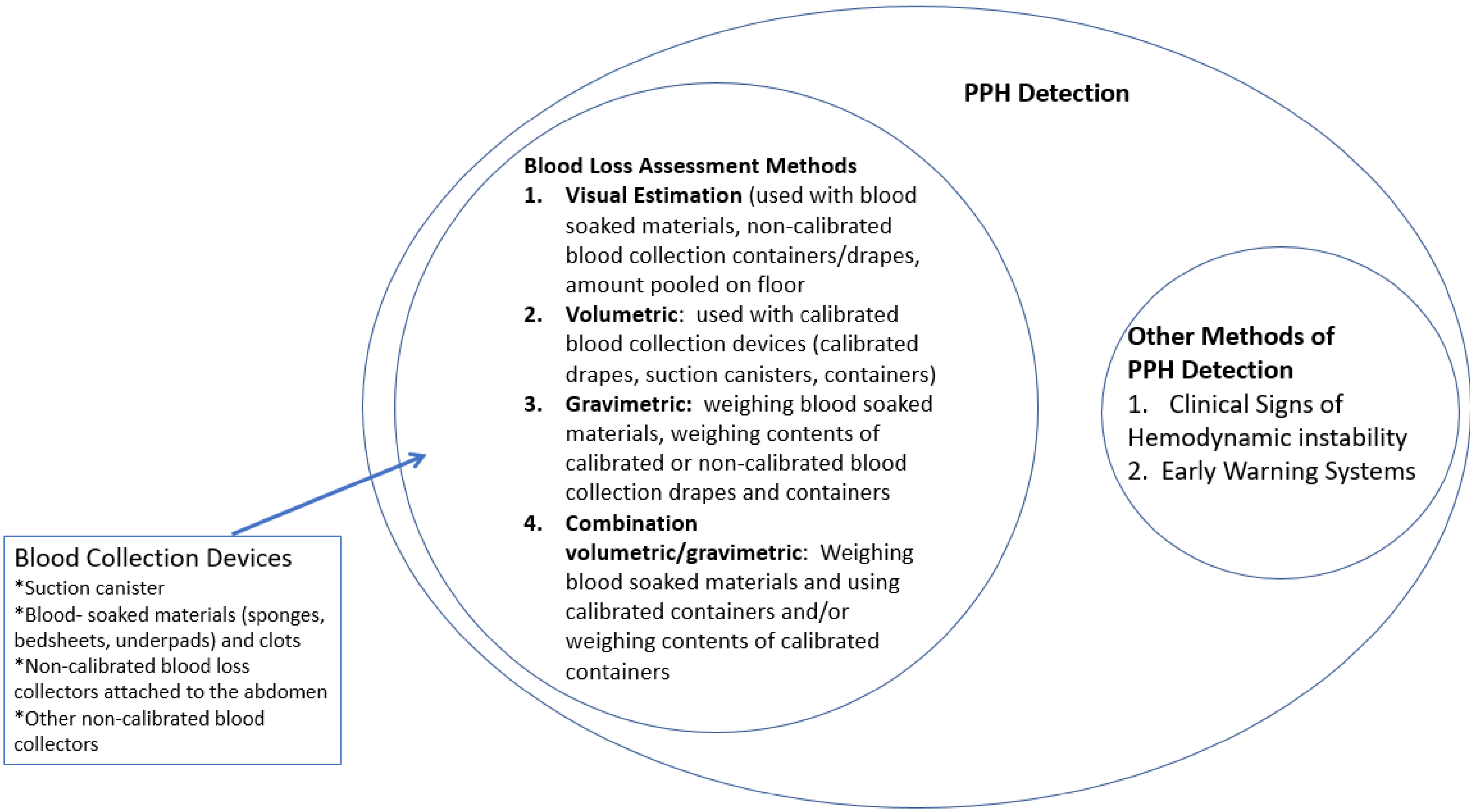

For peer review only - http://bmjopen.bmj.com/site/about/guidelines.xhtml

Supplementary File S3. In-person meeting agenda

WHO Technical Consultation on early detection and first response to PPH during and after caesarean birth

Meeting Agenda
27-28 September 2022
Av. Appia 20, Geneva, Salle U2
Zoom: https://who.zoom.us/j/91683929578
(Meeting ID: 916 8392 9578; Password: &uqmG2dD)

Introduction

Thank you for your participation in the **WHO Technical Consultation on early detection and first response to PPH during and after caesarean birth**. This consultation is part of the ongoing EMOTIVE project (Early detection of Postpartum Haemorrhage and treatment using the WHO MOTIVE 'first response' bundle), coordinated by the University of Birmingham.

As a part of the EMOTIVE project, WHO and some EMOTIVE team members are conducting a three-stage modified Delphi process to generate consensus on the optimal approach for early detection and first-response treatment for postpartum haemorrhage occurring during (intraoperative) and after (postoperative) caesarean birth. The first two rounds of this consultation were informed by an overview of reviews of the literature and conducted via an anonymous online platform. This third, in-person round will serve to conclude the consultation.

Experts' Meeting Objectives

This meeting has two primary objectives:

To agree on an optimal CS-PPH detection strategy that would include blood loss measurement methods and thresholds for action during and after caesarean birth.

To develop a first response approach to manage PPH during and after caesarean birth. This objective includes both the selection of the strategy components and the identification of the optimal approach (bundle, algorithm, checklist, or a combination of them)

For peer review only - http://bmjopen.bmj.com/site/about/guidelines.xhtml

Organization of the Meeting

This meeting will be conducted in-person and via Zoom over the course of two (2) days. Please use this link to access the meeting virtually: https://who.zoom.us/j/91683929578 (Meeting ID: 916 8392 9578; Password: &uqmG2dD)

The first day of the meeting, we will present and discuss the results of the overview of reviews and first two rounds of the Delphi study. You will have the opportunity to ask questions regarding the findings. Later in the day, we will pose specific questions to the panel regarding issues raised by the results to-date. You will be asked to provide your opinion on each of these topics. Discussions will be focused first on the intraoperative period, and then the postoperative period. The objective is to present different points of view on methods for early detection of CS-PPH, thresholds for triggering action, and first-response treatments by the close of day 1.

The second day of the meeting, we will present proposed strategies for implementing early detection and first-response for CS-PPH in the intraoperative and postoperative periods. You will be asked to provide your opinions on the proposed strategies. Finally, you will be asked to rate the proposed strategies. The final results—including whether consensus was reached—and conclusion will be presented before the end of day 2.

Dates and time

Date: 27-28 September 2022	
Day 1	*Day 2*
Africa<br>• *Kenya — 10:00-18:05*<br>• *Nigeria — 08:00-16:05*<br>• *Republic of Congo —08:00-16:05*<br>• *South Africa — 09:00-17:05*	U. Africa<br>• *Kenya — 11:00-14:05*<br>• *Nigeria — 09:00-12:05*<br>• *Republic of Congo — 09:00-12:05*<br>• *South Africa — 10:00-13:05*
Americas<br>• *Argentina — 04:00-12:05*<br>• *Uruguay — 04:00-12:05*	Americas<br>• *Argentina — 05:00-08:05*<br>• *Uruguay — 05:00-08:05*
Europe<br>• *Denmark — 09:00-17:05*<br>• *France (Paris) — 09:00-17:05*<br>• *Switzerland — 09:00-17:05*<br>• *United Kingdom — 08:00-16:05*	Europe<br>• *Denmark — 09:00-12:05*<br>• *France — 10:00-13:05*<br>• *Switzerland — 10:00-13:05*<br>• *United Kingdom — 09:00-12:05*
South-East Asia<br>• *Australia (Melbourne) — 17:00-01:05*<br>• *Egypt — 09:00-17:05*<br>• *Vietnam — 14:00-22:05*	South-East Asia<br>• *Australia — 18:00-21:05*<br>• *Egypt — 10:00-13:05*<br>• *Vietnam — 15:00-18:05*
Western Pacific<br>• *India — 12:30-20:35*<br>• *Philippines —15:00-23:05*<br>• *Thailand — 14:00-22:05*	Western Pacific<br>• *India — 13:30-16:35*<br>• *Philippines — 16:00-19:05*<br>• *Thailand — 15:00-18:05*

For peer review only - http://bmjopen.bmj.com/site/about/guidelines.xhtml

AGENDA

**DAY 1: 27 September 2022**		**Presenter**
9:00 – 9:30<br>*30 min*	*Opening session*<br>— Welcome and introductions<br>— Meeting objectives<br>— Meeting logistics	Dr. Olufemi Oladapo<br>Dr. Ioannis Gallos<br>Dr. Fernando Althabe<br>Ms. Caitlin Williams
9:30 – 10:30<br>*60 min*	*Session 1*<br>— Brief review of the objectives and methods of the Delphi study<br>— Briefly summarize the state of the literature<br>— Findings from the first and second rounds of the Delphi study<br>— Questions	Ms. Verónica Pingray<br>Ms. Caitlin Williams<br>Ms. Verónica Pingray<br>Prof. Suellen Miller
10:30 – 10:45<br>*15 min*	*Coffee/Tea Break*	
10:45 – 12:45<br>*120 min*	*Session 2*<br>— Briefly review summary of findings on **intraoperative** period<br>— Guided discussions by topic	Ms. Verónica Pingray<br>Prof. Suellen Miller
12:45 – 13:30<br>*45 min*	*Lunch*	
13:30 – 14:15<br>*45 min*	*Continue Session 2*	Prof. Suellen Miller
14:15 – 15:45<br>*90 min*	*Session 3*<br>— Briefly review summary of findings on **postoperative** period<br>— Guided discussions by topic (detection, thresholds, first-response treatments)	Ms. Verónica Pingray<br>Dr. Fernando Althabe
15:45 – 16:00<br>*15 min*	*Coffee/Tea Break*	
16:00 – 17:00<br>*60 min*	*Continue Session 3*	Dr. Fernando Althabe
17:00 – 17:05<br>*5 min*	*Closing DAY 1*	Dr. Ioannis Gallos
**DAY 2: 28 September 2022**		
10:00 – 10:15<br>*15 min*	*Welcome*<br>— Objective and procedures for Day 2	Dr. Fernando Althabe
10:15 – 11:00<br>*45 min*	*Session 1*<br>— Presentation of a revised list of early detection and first-response treatments for intraoperative and postoperative PPH<br>— Final voting for the intraoperative and postoperative interventions and presentation of results	Prof. Suellen Miller<br>Ms. Caitlin Williams
11:00 – 11:15<br>*15 min*	*Coffee/Tea Break*	
11:15 – 12:35<br>*80 min*	*Session 2*<br>— Organization of an implementation strategy<br>— Guided discussion of possible additional considerations	Dr. Fernando Althabe<br>Prof. Suellen Miller
12:35 – 13:00<br>*25 min*	*Session 3*<br>— Conclusion and next steps	Dr. Fernando Althabe
13:00 – 13:05<br>*5 min*	*Closing DAY 2*	Dr. Arri Coomarasamy<br>Dr. Olufemi Oladapo

For peer review only - http://bmjopen.bmj.com/site/about/guidelines.xhtml

Supplementary File S4. In-person meeting discussion question guide

Early detection and first response to postpartum haemorrhage during and after caesarean birth A Modified-Delphi Study

Pending controversies or disagreements issues to discuss
27-28 September 2022

DAY 1: 27 September 2022

Definitions:
The experts agreed that the same definition of PPH should be used for both vaginal and caesarean births (same regardless of the mode of birth). Currently the WHO defines PPH as blood loss at least 500 mL within 24 hours after birth.

Draft proposed list of intraoperative interventions

Early detection of PPH and thresholds for triggering first-response management

- Aspirated blood volume (+ weighted pads, sponges, gauzes, etc. if feasible) at least 1000 mL, OR
- Haemodynamic instability (blood pressure, heart rate, oximetry) with any blood loss volume

# Register the final amount of intraoperative blood loss and hand over this information to recovery area.

First-response treatment

- TXA (1g in 10 mL IV over 10 min) for all women
- Examine and rapidly initiate cause-specific first response:
  - If trauma: Rapid haemostasis: hysterorraphy, tears, wound.
  - If atony/placental cause: uterotonics and intra-abdominal uterine massage or exteriorize the uterus and massage
- Uterotonics for all women
- IV fluids with crystalloids for all women

Pending issues

Thresholds

Background: The experts agreed that blood loss of at least 1000 mL should be used as a threshold to trigger first-response management.

	%	Ranking
**Intraoperative**		
≥1000 mL blood loss OR signs of hemodynamic instability, whichever comes first	72.7	1
Hemodynamic instability alone, regardless of volume of blood loss	40.9	2
≥1000 ml (blood loss alone, regardless of signs of hemodynamic instability)	50.0	3
≥500 mL blood loss OR signs of hemodynamic instability, whichever comes first	50.0	4
**Postoperative**		
≥1000 mL blood loss OR signs of hemodynamic instability, whichever comes first	59.1	1
Hemodynamic instability alone, regardless of volume of blood loss	36.4	2
≥1000 ml (blood loss alone, regardless of signs of hemodynamic instability)	36.7	3
≥500 mL blood loss OR signs of hemodynamic instability, whichever comes first	50.0	4

We believe is important to discuss the overall implications of the different thresholds on clinical practice, in the context of the total blood that women lose during and after CS. At the meeting we will present a summary of what is known so far from PPH prevention trials.

For peer review only - http://bmjopen.bmj.com/site/about/guidelines.xhtml

Question 1: Now that we have reviewed this additional data, do we need to revise the top-ranked threshold from the previous rounds (at least 1000 mL OR signs of haemodynamic instability, whichever comes first)?

Question 1.1: If the answer is not to revise, then is it acceptable that the definition (500 mL) and threshold (1000 mL) for triggering action differ?

Treatments

Background: The experts agreed to an aetiology-based treatment approach, but also agreed that the uterotonic of choice should be administered for all women, regardless of presence of atony.

Treatment Options	Median
Examine and rapidly initiate cause-specific first response (e.g., if trauma: rapid surgical haemostasis; if atony/placental cause: uterotonics and uterine massage)	9
Tranexamic acid for all women with PPH during CS regardless of aetiology	8
Plasma expansion with crystalloids for all women with PPH during CS regardless of aetiology	7.5
Uterotonics for all women with PPH during CS regardless of aetiology	7

Question 2: How do we make a recommendation that encompasses these seemingly contradictory statements (i.e., should the first-response uterotonic be administered to all women or just to women with atony?)?

Background: The experts agreed that a first response to intraoperative PPH would be to give oxytocin and TXA. However, many women having a caesarean birth would have already received/or be receiving IV oxytocin, and perhaps TXA, for PPH prevention.

Treatment Options	Median
Examine and rapidly initiate cause-specific first response (e.g., if trauma: rapid surgical haemostasis; if atony/placental cause: uterotonics and uterine massage)	9
Tranexamic acid for all women with PPH during CS regardless of aetiology	8
Plasma expansion with crystalloids for all women with PPH during CS regardless of aetiology	7.5
Uterotonics for all women with PPH during CS regardless of aetiology	7

Question 3: How do we adapt first-response recommendations if women are already receiving oxytocin infusion (common practice and consensus statement) for PPH prevention?

Question 4: In the event that women will routinely be receiving TXA for PPH prevention during CS, what recommendations should we give should they begin to haemorrhage intraoperatively at less than 30 min from the first dose?

Regarding IV fluids

Background: In the survey, the experts agreed that plasma expansion with crystalloids should be used for all women with PPH during CS, regardless of aetiology.

Treatment Options	Median
Examine and rapidly initiate cause-specific first response (e.g., if trauma: rapid surgical haemostasis; if atony/placental cause: uterotonics and uterine massage)	9
Tranexamic acid for all women with PPH during CS regardless of aetiology	8
Plasma expansion with crystalloids for all women with PPH during CS regardless of aetiology	7.5
Uterotonics for all women with PPH during CS regardless of aetiology	7

Question 5: Although we have used “Plasma expansion with crystalloids” in previous surveys, it may not be the best term. Should we use a different term (e.g., increase IV fluids with crystalloids for hemodynamic

For peer review only - http://bmjopen.bmj.com/site/about/guidelines.xhtml

maintenance)?<br><br>***Question 6:*** What should our recommendations be about how to continue IV fluids infusion with crystalloids for hemodynamic maintenance?<br><br>***Question 7:*** Do you think that we should be giving more details on type of crystalloids?<br><br>**Additional considerations**<br>***Question 8:*** Are there/should there be additional considerations for any other first-response treatments during the intraoperative period?
**Draft proposed list of postoperative interventions**

For peer review only

Early detection of PPH and thresholds for triggering first-response management

- Haemodynamic instability (blood pressure, heart rate, oxygen saturation) with any blood loss volume, OR
- Accumulated blood loss at least 1000 mL (weighted pads + blood loss during CS)

# If blood loss during CS <500 ml: usual postoperative clinical monitoring.

# If blood loss during CS 500-999 ml: further assessment, preparedness, and close monitoring

First response treatment

- TXA (1g in 10 mL IV over 10 min) for all women
- Examine and rapidly initiate cause-specific first response:
  - If trauma suspected: re-laparotomy for surgical haemostasis
  - If atony: Uterotonics
- Uterotonics for all women
- IV fluids with crystalloids for all women

Pending issues

Background: During the survey, we asked questions about the detection methods, thresholds, and treatments using 2 hours as the postoperative period.

Question 9: Is it valid to extend the results to the first 24 hours, or is a different strategy needed for hours 1-2 hours postoperative versus 3-24 hours postoperative?

For example: Should a woman with cumulative blood loss of 1100 mL in the first 2 hrs have her bleeding managed in the same way as a woman with a cumulative blood loss of 1100 mL in the first 24 hrs?

Detection & thresholds

Background: The panel agreed that using **clinical signs of haemodynamic instability** is a more appropriate option for detecting postoperative PPH due to the risk of internal bleeding postoperative.

Additionally, the panel agreed that deploying volumetric methods (e.g., drapes) would be less feasible than monitoring vital signs. Reasons given were a) the possible low acceptability by women (due to discomfort) and b) the lack of feasibility of continued measurement during women's transfer and normal movements.

Blood loss measurement and other PPH detection methods	How would you rate each of the methods below for early detection of PPH considering... usefulness?	feasibility?	acceptability?	resources required?
Clinical signs of haemodynamic instability	8.0	8.0	8.0	3.5
Volumetric/gravimetric + clinical signs of haemodynamic instability	7.0	7.0	NA	NA
Clinical judgement such as rate of flow and duration	5.0	6.0	7.0	2.0
Gravimetric	5.0	3.0	5.0	7.0
Volumetric	5.0	4.5	7.0	7.0
Visual estimation of blood loss	4.0	7.0	7.0	1.0
Visual charts and early warning scores (EWS)	7.0	7.0	6.5	6.0
Visual estimation + visual charts /EWS	7.0	7.0	NA	NA
Volumetric + gravimetric	7.0	5.0	NA	NA

Given the diverse realities of postoperative monitoring, priority should be given either to frequent monitoring of vital signs or the use of continuous monitoring devices, dependent on the availability of equipment and personnel.

Question 10: Consequently, what kind of guidance should we give, if any? How frequently should the woman's haemodynamic status be monitored? For how long? With what kind of devices?

Background: In addition to monitoring vital signs, providers should be **monitoring blood loss.** The experts agreed that in the postoperative period, providers should continue with the cumulative measurement of blood loss (i.e., adding intraoperative blood loss to postoperative blood loss to calculate total loss).

For peer review only - http://bmjopen.bmj.com/site/about/guidelines.xhtml

	Median
The volume of intraoperative blood loss should be taken into consideration when determining whether the postoperative threshold had been reached	8

Question 11: What guidance should we give to reflect cumulative intra- and postoperative blood loss?

We propose, **as an example**, the following approach to blood loss monitoring **in situations in which the woman's vital signs are stable**. ***If vital signs indicate haemodynamic instability, the guidance should be to initiate treatment regardless of postoperative blood loss.***

Intraoperative		Postoperative		
**Blood Loss**	**Action Indicated**	**Type of Monitoring**	**Cumulative Blood Loss**	**Action Indicated**
<500	No treatment	Normal monitoring	Cumulative blood loss <1000	Do not initiate treatment. Continue normal monitoring
500-999	No treatment	Further assessment, preparedness, and more frequent postop monitoring	Cumulative blood loss <1000	Do not initiate treatment. Return to normal monitoring
500-999	No treatment	Further assessment, preparedness, and more frequent postop monitoring	Cumulative blood loss >1000	Initiate treatment, and monitor the woman's response to the treatment
≥1000	Initiate treatment and monitor woman's response	Monitor woman's response to treatments	-	-

DAY 2: 28 September 2022

Question 12: How should we frame the first-response strategies? Or bundle?

- Algorithm?
- Checklist?
- Or a hybrid?

Question 13: Should we include preparedness interventions at the facility level?
Prepare for CS PPH

For peer review only - http://bmjopen.bmj.com/site/about/guidelines.xhtml

Blood loss measurement

- Calibrated containers
- Pump/aspirator/vacuum suction
- Pads/swabs/lap cloths, etc. with known dry weight
- Scale to weigh the above

Medications

- TXA
- Uterotonics (Oxytocin, ergometrine, sulprostone)
- Crystalloids

Haemodynamic Monitoring

- CRADLE device/ cardiac monitor/ oximeter/sphygmomanometer?

For peer review only

For peer review only - http://bmjopen.bmj.com/site/about/guidelines.xhtml

Supplementary File S5. List of contributors

EXPERTS

Fadhlun ALWY AL-BEITY
Senior Lecturer - OBGYN
Tanzania

Nabhan ASHRAF
Professor of Obstetrics and Gynecology
Egypt

Brendan CARVALHO
Professor of Anesthesiology, Perioperative and Pain Medicine
USA

Maria Fernanda ESCOBAR VIDARTE
Professor of Obstetrics and Gynaecology
Colombia

Cherrie EVANS
Senior MNH Advisor
USA

Tippawan LIABSUETRAKUL
Professor of Obstetrics and Gynecology
Thailand

Elliott MAIN
Professor of Obstetrics and Gynecology
USA

Zahida QURESHI
OBGYN and associate professor of Obstetrics and Gynaecology
Kenya

John VARALLO
Global Director Safe Surgery
USA

Edgardo ABALOS
OBGYN and senior researcher
Argentina

Sabaratnam ARULKUMARAN
Emeritus Professor of Obstetrics and Gynaecology
UK

Catherine DENEUX-THARAUX
Perinatal Epidemiologist
France

Sue FAWCUS
Emeritus Professor of Obstetrics and Gynaecology and senior research scholar
South Africa

Hadiza GALADANCI
Professor of Obstetrics and Gynecology
Nigeria

Caroline HOMER
Professor and Co-Program Director Maternal, Child and Adolescent Health
Australia

Justus HOFMEYR
OBGYN and senior researcher
South Africa

Judith Maua ONG'AYI
Senior Technical Officer
Kenya

PHAN NGUYEN Quoc Thuan
OBGYN Clinician and clinical researcher
Vietnam

Alexander DUMONT
Senior Researcher
France

Pisake Lumbiganon
Dean and Professor of Obstetrics and Gynecology Convenor, Thai Cochrane Network Faculty of Medicine Khon Kaen University Khon Kaen
Thailand

For peer review only

OBSERVERS

Arri COOMARASAMY
OBGYN and Professor of Gynaecology
UK

Inês NUNES
OBGYN clinician
Assistant Professor of Obstetrics and Gynecology
Portugal

Andrew WEEKS
Professor of International Maternal Health
UK

STEERING GROUP

Fernando ALTHABE
WHO Consultant
Argentina

Suellen MILLER
Director, Safe Motherhood Program
USA

Veronica PINGRAY
Perinatal epidemiologist and methodologist
Argentina

Caitlin R. WILLIAMS
Researcher
USA

WHO REGIONAL ADVISORS

Claudio SOSA
Consultant
Woman and Reproduction Health Unit
Maternal Health at the Latin American Center of Perinatology,
Women and Reproductive Health,
(CLAP/WR), PAHO

WHO SECRETARIAT

Ioannis GALLOS
Medical Officer, SRH/MPH
Maternal and Perinatal Health
Department of Sexual and Reproductive Health and Research

Olufemi OLADAPO
Unit Head, SRH/MPH
Maternal and Perinatal Health
Department of Sexual and Reproductive Health and Research

Mariana WIDMER
Technical Officer, SRH/MPH
Maternal and Perinatal Health
Department of Sexual and Reproductive Health and Research

For peer review only

Supplementary File S6. Good Surgical Practices

Prevention and early diagnosis of PPH should be applied simultaneously with good surgical practices. One way adherence to these practices can be enhanced is by using Surgical Safety Checklists. WHO has such a Surgical Safety Checklist (SSC), which is generic for any surgical procedure (47). This Checklist was developed with many collaborators. Its purpose is to reduce mistakes, omissions, and adverse outcomes, as well as to improve communication and teamwork during surgery. One study demonstrated that serious morbidity and mortality were reduced from 1.5% to 0.8% by using the WHO SCC in a variety of global settings (48). Several of the expert panel members mentioned using the WHO SSC as an umbrella of good clinical practices for preventing or reducing risks of haemorrhage at CS.

One problem with the generic SSC for caesarean births is the complexity of the specifics of the caesarean surgery and the frequency of PPH at CS. To address this a few organizations have modified the WHO SSC for CS in lower resourced settings or developed surgical checklists specifically for CS (4950). A copy of the Sun *et al*. checklist can be found below (**Figure 1**)

Additional guidance for safety during and after CS can be found in the WHO MCPC (51).

Following good surgical practices are implied as necessary steps for all teams involved in performing CS. There are commonalities among all checklists, but those specifically developed/modified for CS include information about the placenta, risk factors for maternal haemorrhage, including anaemia, and if there are risks present to prepare by having 2 large bore IVs, keeping uterotonics at hand, giving antibiotics before skin incision, foley for urine drainage, anti-microbial skin wash, estimation of blood loss after the surgery and, if PPH occurred, an order to be written for nursing to check Hct, continue IVs, record urine output, and to alert physician if Hct < 20, BP < 80/50, and pulse > 100, and or urine output < 30 cc hr. These more specific parameters and procedures for intra and postop CS management can be useful along with the Hybrid Algorithm-Bundle Strategy we are suggesting.

For peer review only - http://bmjopen.bmj.com/site/about/guidelines.xhtml

Figure 1: Sun et al. Caesarean Birth Checklist

NURSE: Name ______________________ Date/Time ________________

Prior to starting surgery

- ☐ Verify patient name ______________________
- ☐ Allergies ______________
- ☐ Indication __________________________________
- ☐ Fetal presentation: Cephalic / Breech / Transverse – back down / Transverse – back up
- ☐ Placentation: Anterior / Posterior / Lateral / Fundal / Previa
- ☐ High risk for maternal hemorrhage? (multiple prior cesareans, chorioamnionitis, polyhydramnios, twin gestation, magnesium sulfate, prolonged Pitocin infusion)
  - ☐ Yes
    - ☐ 2 large bore IVs in patient with 500cc infusing now
    - ☐ Cytotec in the Salle d'Op
    - ☐ Ergometrine in the Salle d'Op
    - ☐ Oxytocin in the Salle d'Op
  - ☐ No
- ☐ Antibiotics given before skin incision
- ☐ Bladder foley placed
- ☐ Safety belt on patient's leg, above knees
- ☐ Left lateral displacement
- ☐ Skin preparation
  - o Wash
  - o Betadine

DOCTOR: Name___________________ Date/Time _________________

After completion of surgery

- ☐ Instrument and sponge counts correct? Yes / No
- ☐ Estimation of blood loss______________
  - ☐ Normal blood loss
  - ☐ Large blood loss
    - ☐ Write order for nurse to do each of the following:
      - ☐ Check Hct immediately and 4 hours post-operatively
      - ☐ Give 1000cc Normal Saline Bolus
      - ☐ Record urine output every hour
      - ☐ Call doctor if Hct ≤20, BP<80/50 and pulse >100, urine output <30cc/hour
- ☐ Complications
  - ☐ None
  - ☐ Blood transfusion intra-operatively
  - ☐ Maternal death
  - ☐ Perinatal death
  - ☐ Hysterectomy
  - ☐ Uterine artery laceration
  - ☐ Internal organ trauma (bladder, bowel, etc)
  - ☐ Vertical extension of uterine incision

For peer review only - http://bmjopen.bmj.com/site/about/guidelines.xhtml

References

1. World Health Organization. WHO recommendations for the prevention and treatment of postpartum haemorrhage. Geneva: World Health Organization; 2012. 41- p.
2. Mavrides E, Allard S, Chandraharan E, Collins P, Green L, Hunt BJ, et al. Prevention and management of postpartum haemorrhage. BJOG: An International Journal of Obstetrics & Gynaecology. 2016;124:e106-e49.
3. Sentilhes L, Vayssière C, Deneux-Tharaux C, Aya AG, Bayoumeu F, Bonnet MP, et al. Postpartum hemorrhage: Guidelines for clinical practice from the French College of Gynaecologists and Obstetricians (CNGOF): In collaboration with the French Society of Anesthesiology and Intensive Care (SFAR). European Journal of Obstetrics and Gynecology and Reproductive Biology. 2016;198(2016):12-21.
4. Institute of O, Gynaecologists Royal College of Physicians of I, Directorate of S, Clinical Programmes Health Service E. Prevention and management of primary postpartum haemorrhage. 2014. p. 12-26.
5. RANZCOG. Management of Postpartum Haemorrhage (PPH). 2017. p. 1-14.
6. Muñoz M, Stensballe J, Ducloy-Bouthors A-S, Bonnet M-P, De Robertis E, Fornet I, et al. Patient blood management in obstetrics: prevention and treatment of postpartum haemorrhage. A NATA consensus statement. Blood transfusion = Trasfusione del sangue. 2019;17(2):112-36.
7. Schlembach D, Helmer H, Henrich W, von Heymann C, Kainer F, Korte W, et al. Peripartum haemorrhage, diagnosis and therapy. Guideline of the DGGG, OEGGG and SGGG (S2k Level, AWMF Registry No. 015/063, March 2016). Geburtshilfe und Frauenheilkunde. 2018;78(04):382-99.
8. Minakami H, Maeda T, Fujii T, Hamada H, Iitsuka Y, Itakura A, et al. Guidelines for obstetrical practice in Japan: Japan Society of Obstetrics and Gynecology (JSOG) and Japan Association of Obstetricians and Gynecologists (JAOG) 2014 edition. Journal of Obstetrics and Gynaecology Research. 2014;40(6):1469-99.
9. World Health Organization. WHO Recommendation on Tranexamic Acid for the Treatment of Postpartum Haemorrhage. Geneva: World Health Organization; 2017. 41- p.
10. Lalonde A, Motherhood FS, Newbon Health C. Prevention and treatment of postpartum hemorrhage in low-resource settings. International Journal of Gynecology & Obstetrics. 2012;117(2):108-18.
11. ACOG. Postpartum Hemorrhage. Obstetrics & Gynecology. 2017;183:e168-e86.
12. Queensland Clinical Guidelines. Maternity and neonatal clinical guideline: Primary postpartum haemorrhage. Queensland Health. 2018:1-39.
13. CMQCC, Health CDoP. Improving Health Care Response to Obstetric Hemorrhage Version 2.0: A California Quality Improvement Toolkit2015.
14. Leduc D, Senikas V, Lalonde AB. No. 235-Active Management of the Third Stage of Labour: Prevention and Treatment of Postpartum Hemorrhage. Journal of Obstetrics and Gynaecology Canada. 2018;40(12):e841-e55.
15. NICE. Caesarean birth 2021 [cited 2023 Apr 30]. Available from: https://www.nice.org.uk/guidance/ng192.
16. World Health Organization. Managing complications in pregnancy and childbirth: A guide for midwives and doctors. 2nd ed. Geneva: World Health Organization; 2017. 492- p.
17. Smith CM, Borders AE, King TL. Quantitative Blood Loss in Obstetric Hemorrhage: ACOG COMMITTEE OPINION SUMMARY, Number 794. Obstetrics and gynecology. 2019;134(6):1368-9.
18. World Health O. WHO Recommendation on Tranexamic Acid for the Treatment of Postpartum Haemorrhage. WHO Recommendation on Tranexamic Acid for the Treatment of Postpartum Haemorrhage. 2017:41-.
19. Gallos ID, Papadopoulou A, Man R, Athanasopoulos N, Tobias A, Price MJ, et al. Uterotonic agents for preventing postpartum haemorrhage: A network meta-analysis. Cochrane Database of Systematic Reviews. 2018;2018(12).
20. Kellie FJ, Wandabwa JN, Mousa HA, Weeks AD. Mechanical and surgical interventions for treating primary postpartum haemorrhage. Cochrane Database of Systematic Reviews. 2020.

For peer review only - http://bmjopen.bmj.com/site/about/guidelines.xhtml

21. Parry Smith WR, Papadopoulou A, Thomas E, Tobias A, Price MJ, Meher S, et al. Uterotonic agents for first-line treatment of postpartum haemorrhage: A network meta-analysis. Cochrane Database of Systematic Reviews. 2020;2020(11).
22. Hancock A, Weeks AD, Lavender DT. Is accurate and reliable blood loss estimation the 'crucial step' in early detection of postpartum haemorrhage: an integrative review of the literature. BMC Pregnancy and Childbirth 2015 15:1. 2015;15(1):1-9.
23. Andrikopoulou M, D'Alton ME. Postpartum hemorrhage: Early identification challenges. Seminars in Perinatology. 2019;43(1):11-7.
24. Diaz V, Abalos E, Carroli G. Methods for blood loss estimation after vaginal birth. Cochrane Database of Systematic Reviews. 2018;2018(9).
25. Lertbunnaphong T, Lapthanapat N, Leetheeragul J, Hakularb P, Ownon A. Postpartum blood loss: visual estimation versus objective quantification with a novel birthing drape. Singapore Medical Journal. 2016;57(06):325-8.
26. Patel A, Goudar SS, Geller SE, Kodkany BS, Edlavitch SA, Wagh K, et al. Drape estimation vs. visual assessment for estimating postpartum hemorrhage. International Journal of Gynecology & Obstetrics. 2006;93(3):220-4.
27. Zhang WH, Deneux-Tharaux C, Brocklehurst P, Juszczak E, Joslin M, Alexander S. Effect of a collector bag for measurement of postpartum blood loss after vaginal delivery: cluster randomised trial in 13 European countries. BMJ. 2010;340(feb01 1):c293-c.
28. Ambardekar S, Shochet T, Bracken H, Coyaji K, Winikoff B. Calibrated delivery drape versus indirect gravimetric technique for the measurement of blood loss after delivery: a randomized trial. BMC Pregnancy and Childbirth 2014 14:1. 2014;14(1):1-6.
29. Atukunda EC, Mugyenyi GR, Obua C, Atuhumuza EB, Musinguzi N, Tornes YF, et al. Measuring post-partum haemorrhage in low-resource settings: The diagnostic validity of weighed blood loss versus quantitative changes in hemoglobin. PLOS ONE. 2016;11(4):e0152408-e.
30. Ushida T, Kotani T, Imai K, Nakano-Kobayashi T, Nakamura N, Moriyama Y, et al. Shock index and postpartum hemorrhage in vaginal deliveries: A multicenter retrospective study. Shock. 2021;55(3):332-7.
31. Borovac-Pinheiro A, Ribeiro FM, Morais SS, Pacagnella RC. Shock index and heart rate standard reference values in the immediate postpartum period: A cohort study. PLOS ONE. 2019;14(6):e0217907-e.
32. El Ayadi AM, Nathan HL, Seed PT, Butrick EA, Hezelgrave NL, Shennan AH, et al. Vital sign prediction of adverse maternal outcomes in women with hypovolemic shock: The role of shock index. PLOS ONE. 2016;11(2):e0148729-e.
33. Pacagnella RC, Borovac-Pinheiro A, Silveira C, Siani Morais S, Argenton JLP, Souza JP, et al. The golden hour for postpartum hemorrhage: Results from a prospective cohort study. International Journal of Gynecology & Obstetrics. 2021:ijgo.13823-ijgo.
34. Tanacan A, Fadiloglu E, Unal C, Beksac MS. Importance of shock index in the evaluation of postpartum hemorrhage cases that necessitate blood transfusion. Women & Health. 2020;60(9):1070-8.
35. Lee SY, Kim HY, Cho GJ, Hong SC, Oh MJ, Kim HJ. Use of the shock index to predict maternal outcomes in women referred for postpartum hemorrhage. International Journal of Gynecology & Obstetrics. 2018:ijgo.12714-ijgo.
36. Vousden N, Lawley E, Nathan HL, Seed PT, Gidiri MF, Goudar S, et al. Effect of a novel vital sign device on maternal mortality and morbidity in low-resource settings: a pragmatic, stepped-wedge, cluster-randomised controlled trial. The Lancet Global Health. 2019;7(3):e347-e56.
37. Umar A, Ameh CA, Muriithi F, Mathai M. Early warning systems in obstetrics: A systematic literature review. PLOS ONE. 2019;14(5):e0217864-e.
38. World Health Organization. WHO recommendations: Uterotonics for the prevention of postpartum haemorrhage. Geneva: World Health Organization; 2018. p. 53-.
39. World Health Organization. WHO recommendation on uterine balloon tamponade for the treatment of postpartum haemorrhage. Geneva: World Health Organization; 2021.
40. Shakur H, Beaumont D, Pavord S, Gayet-Ageron A, Ker K, Mousa HA. Antifibrinolytic drugs for treating primary postpartum haemorrhage. Cochrane Database of Systematic Reviews. 2018;2018(2).

For peer review only - http://bmjopen.bmj.com/site/about/guidelines.xhtml

41. Kavak SB, Atilgan R, Demirel İ, Celik E, İlhan R, Sapmaz E. Endouterine hemostatic square suture vs. Bakri balloon tamponade for intractable hemorrhage due to complete placenta previa. Journal of Perinatal Medicine. 2013;41(6).
42. Khalil MI, Al-Dohami H, Aldahish MM. A method to improve the effectiveness of the Bakri balloon for management of postpartum hemorrhage at cesarean. International Journal of Gynecology & Obstetrics. 2011;115(2):198-200.
43. Pileggi-Castro C, Nogueira-Pileggi V, Tunçalp Ö, Oladapo OT, Vogel JP, Souza JP. Non-pneumatic anti-shock garment for improving maternal survival following severe postpartum haemorrhage: a systematic review. Reprod Health. 2015;12:28.
44. Brouwers MC, Kho ME, Browman GP, Burgers JS, Cluzeau F, Feder G, et al. AGREE II: Advancing guideline development, reporting and evaluation in health care. Can Med Assoc J [Internet]. 2010 Dec 14;182(18):E839–42. Available from: http://www.cmaj.ca/cgi/doi/10.1503/cmaj.090449
45. Shea BJ, Reeves BC, Wells G, Thuku M, Hamel C, Moran J, et al. AMSTAR 2: A critical appraisal tool for systematic reviews that include randomised or non-randomised studies of healthcare interventions, or both. BMJ [Internet]. 2017 Sep 21;j4008. Available from: https://www.bmj.com/lookup/doi/10.1136/bmj.j4008
46. Mousa HA, Blum J, Abou El Senoun G, Shakur H, Alfirevic Z. Treatment for primary postpartum haemorrhage. Cochrane Database Syst Rev [Internet]. 2014 Feb 12; Available from: https://doi.wiley.com/10.1002/14651858.CD003249.pub3
47. Tool and resources [Internet]. [cited 2024 Jan 10]. Available from: https://www.who.int/teams/integrated-health-services/patient-safety/research/safe-surgery/tool-and-resources
48. Haynes AB, Weiser TG, Berry WR, Lipsitz SR, Breizat A-HS, Dellinger EP, et al. A surgical safety checklist to reduce morbidity and mortality in a global population. N Engl J Med. 2009 Jan 29;360(5):491–9.
49. Sun M, Patauli D, Bernstein PS, Goffman D, Nathan LM. Use of a cesarean delivery checklist in an African maternity ward to improve management and reduce length of hospital stay. Int J Gynaecol Obstet. 2021 Feb;152(2):236–41.
50. Combs CA, Einerson BD, Toner LE, Patient Safety and Quality Committee, Society for Maternal-Fetal Medicine. Electronic address: smfm@smfm.org. Society for Maternal-Fetal Medicine Special Statement: Surgical safety checklists for cesarean delivery. Am J Obstet Gynecol. 2021 Nov;225(5):B43–9.
51. Managing complications in pregnancy and childbirth: A guide for midwives and doctors - Second Edition [Internet]. World Health Organization; 2017 [cited 2024 Jan 10]. Available from: https://www.who.int/publications/i/item/9789241565493

For peer review only - http://bmjopen.bmj.com/site/about/guidelines.xhtml

S1 CREDES Checklist:

Recommendations for the Conducting and REporting of DElphi Studies (CREDES)[1]

Strategies for optimising early detection and first response management of postpartum haemorrhage at caesarean birth: A modified Delphi-based international expert consensus

Items of reporting	Reported on page
Purpose and rationale. The purpose of the study should be clearly defined and demonstrate the appropriateness of the use of the Delphi technique as a method to achieve the research aim. A rationale for the choice of the Delphi technique as the most suitable method needs to be provided.	5
Expert panel. Criteria for the selection of experts and transparent information on recruitment of the expert panel, sociodemographic details including information on expertise regarding the topic in question, (non)response and response rates over the ongoing iterations should be reported.	6, SF 4
Description of the methods. The methods employed need to be comprehensible; this includes information on preparatory steps (How was available evidence on the topic in question synthesised?), piloting of material and survey instruments, design of the survey instrument(s), the number and design of survey rounds, methods of data analysis, processing and synthesis of experts' responses to inform the subsequent survey round and methodological decisions taken by the research team throughout the process.	5-7
Procedure. Flow chart to illustrate the stages of the Delphi process, including a preparatory phase, the actual 'Delphi rounds', interim steps of data processing and analysis, and concluding steps.	Figure 1
Definition and attainment of consensus. It needs to be comprehensible to the reader how consensus was achieved throughout the process, including strategies to deal with non-consensus.	7
Results. Reporting of results for each round separately is highly advisable in order to make the evolving of consensus over the rounds transparent. This includes figures showing the average group response, changes between rounds, as well as any modifications of the survey instrument such as deletion, addition or modification of survey items based on previous rounds.	9-17
Discussion of limitations. Reporting should include a critical reflection of potential limitations and their impact of the resulting guidance.	4 (Box), 19
Adequacy of conclusions. The conclusions should adequately reflect the outcomes of the Delphi study with a view to the scope and applicability of the resulting practice guidance.	21
Publication and dissemination. The resulting guidance on good practice in palliative care should be clearly identifiable from the publication, including recommendations for transfer into practice and implementation. If the publication does not allow for a detailed presentation of either the resulting practice guidance or the methodological features of the applied Delphi technique, or both, reference to a more detailed presentation elsewhere should be made (e.g. availability of the full guideline from the authors or online; publication of a separate paper reporting on methodological details and particularities of the process (e.g. persistent disagreement and controversy on certain issues)). A dissemination plan should include endorsement of the guidance by professional associations and health care authorities to facilitate implementation.	19 - 20

1. Jünger S, Payne SA, Brine J, Radbruch L, Brearley SG. Guidance on Conducting and REporting DElphi Studies (CREDES) in palliative care: Recommendations based on a methodological systematic review. Palliat Med. 2017;31: 684–706. doi:10.1177/0269216317690685

For peer review only - http://bmjopen.bmj.com/site/about/guidelines.xhtml

PRISMA Checklist ScR (Scoping Review)

Section and Topic	Item #	Checklist item	Location where item is reported
**TITLE**			
Title	1	Identify the report as a systematic review.	Supplementary file S1 Page 19
**ABSTRACT**			
Abstract	2	See the PRISMA 2020 for Abstracts checklist.	Main document page 2
**INTRODUCTION**			
Rationale	3	Describe the rationale for the review in the context of existing knowledge.	Main document page 5
Objectives	4	Provide an explicit statement of the objective(s) or question(s) the review addresses.	Main document page 5
**METHODS**			
Eligibility criteria	5	Specify the inclusion and exclusion criteria for the review and how studies were grouped for the syntheses.	Main document page 6; Supplementary file S1 Page 20
Information sources	6	Specify all databases, registers, websites, organisations, reference lists and other sources searched or consulted to identify studies. Specify the date when each source was last searched or consulted.	Main document page 6
Search strategy	7	Present the full search strategies for all databases, registers and websites, including any filters and limits used.	Supplementary file S2 page 43
Selection process	8	Specify the methods used to decide whether a study met the inclusion criteria of the review, including how many reviewers screened each record and each report retrieved, whether they worked independently, and if applicable, details of automation tools used in the process.	Main document page 6; Supplementary file S1 pages 21,22
Data collection process	9	Specify the methods used to collect data from reports, including how many reviewers collected data from each report, whether they worked independently, any processes for obtaining or confirming data from study investigators, and if applicable, details of automation tools used in the process.	Main document page 6; Supplementary file S1 page 23
Data items	10a	List and define all outcomes for which data were sought. Specify whether all results that were compatible with each outcome domain in each study were sought (e.g. for all measures, time points, analyses), and if not, the methods used to decide which results to collect.	Supplementary file S1 pages 22
	10b	List and define all other variables for which data were sought (e.g. participant and intervention characteristics, funding sources). Describe any assumptions made about any missing or unclear information.	Supplementary file S1 pages 22
Study risk of bias assessment	11	Specify the methods used to assess risk of bias in the included studies, including details of the tool(s) used, how many reviewers assessed each study and whether they worked independently, and if applicable, details of automation tools used in the process.	Main document page 6; Supplementary file S1 pages 21,22
Effect measures	12	Specify for each outcome the effect measure(s) (e.g. risk ratio, mean difference) used in the synthesis or presentation of results.	Not applicable to scoping reviews.
Synthesis methods	13a	Describe the processes used to decide which studies were eligible for each synthesis (e.g. tabulating the study intervention characteristics and comparing against the planned groups for each synthesis (item #5)).	Not applicable to scoping reviews.
	13b	Describe any methods required to prepare the data for presentation or synthesis, such as handling of missing summary statistics, or data conversions.	Not applicable to scoping reviews.
	13c	Describe any methods used to tabulate or visually display results of individual studies and syntheses.	Supplementary file S1 pages 23
	13d	Describe any methods used to synthesize results and provide a rationale for the choice(s). If meta-analysis was performed, describe the model(s), method(s) to identify the presence and extent of statistical heterogeneity, and software package(s) used.	Not applicable to scoping reviews.

For peer review only

PRISMA Checklist ScR (Scoping Review)

Section and Topic	Item #	Checklist item	Location where item is reported
	13e	Describe any methods used to explore possible causes of heterogeneity among study results (e.g. subgroup analysis, meta-regression).	Not applicable to scoping reviews.
	13f	Describe any sensitivity analyses conducted to assess robustness of the synthesized results.	Not applicable to scoping reviews.
Reporting bias assessment	14	Describe any methods used to assess risk of bias due to missing results in a synthesis (arising from reporting biases).	Supplementary file S1 page 22,23
Certainty assessment	15	Describe any methods used to assess certainty (or confidence) in the body of evidence for an outcome.	Not applicable to scoping reviews.
**RESULTS**			
Study selection	16a	Describe the results of the search and selection process, from the number of records identified in the search to the number of studies included in the review, ideally using a flow diagram.	Main document page 9; Supplementary file S1 pages 24
	16b	Cite studies that might appear to meet the inclusion criteria, but which were excluded, and explain why they were excluded.	Not reported
Study characteristics	17	Cite each included study and present its characteristics.	Supplementary file S1 pages 25,26, 31-33
Risk of bias in studies	18	Present assessments of risk of bias for each included study.	Supplementary file S1 pages 29,30
Results of individual studies	19	For all outcomes, present, for each study: (a) summary statistics for each group (where appropriate) and (b) an effect estimate and its precision (e.g. confidence/credible interval), ideally using structured tables or plots.	Supplementary tables pages 2-15
Results of syntheses	20a	For each synthesis, briefly summarise the characteristics and risk of bias among contributing studies.	Supplementary file S1 pages 14-16
	20b	Present results of all statistical syntheses conducted. If meta-analysis was done, present for each the summary estimate and its precision (e.g. confidence/credible interval) and measures of statistical heterogeneity. If comparing groups, describe the direction of the effect.	Not applicable to scoping reviews.
	20c	Present results of all investigations of possible causes of heterogeneity among study results.	Not applicable to scoping reviews.
	20d	Present results of all sensitivity analyses conducted to assess the robustness of the synthesized results.	Not applicable to scoping reviews.
Reporting biases	21	Present assessments of risk of bias due to missing results (arising from reporting biases) for each synthesis assessed.	Not applicable to scoping reviews.
Certainty of evidence	22	Present assessments of certainty (or confidence) in the body of evidence for each outcome assessed.	Not applicable to scoping reviews.
**DISCUSSION**			
Discussion	23a	Provide a general interpretation of the results in the context of other evidence.	Main document page 19
	23b	Discuss any limitations of the evidence included in the review.	Main document page 17,18
	23c	Discuss any limitations of the review processes used.	Main document page 17,18
	23d	Discuss implications of the results for practice, policy, and future research.	Main document page 20
**OTHER INFORMATION**			
Funding	24	Describe sources of financial or non-financial support for the review, and the role of the funders or sponsors in the review.	Main document page 20

For peer review only

PRISMA Checklist ScR (Scoping Review)

From: Page MJ, McKenzie JE, Bossuyt PM, Boutron I, Hoffmann TC, Mulrow CD, et al. The PRISMA 2020 statement: an updated guideline for reporting systematic reviews. BMJ 2021;372:n71. doi: 10.1136/bmj.n71

For more information, visit: http://www.prisma-statement.org/

For peer review only
